# Systematic production of human kidney organoids for transplantation in porcine kidneys during ex vivo machine perfusion

**DOI:** 10.1038/s41551-025-01542-1

**Published:** 2025-10-31

**Authors:** Elena Garreta, Daniel Moya-Rull, Alberto Centeno, Andrés Marco, Asier Ullate-Agote, Gaia Amato, Carlos J. Aranda, Roger Oria, Daniel Lozano-Ojalvo, Merel B. F. Pool, Tim L. Hamelink, Idoia Lucía Selfa, Federico González, Carolina Tarantino, Alejandro Montero Salinas, Patricia López San Martín, Priyanka Koshy, Aleix Gavaldà-Navarro, Amaia Vilas-Zornoza, Juan R. Rodríguez-Madoz, Antón Fernández García, Inmaculada Marquez-Leiva, Henri G. D. Leuvenink, Cristobal Belda-Iniesta, Maarten Naesens, Beatriz Dominguez-Gil, Marcelino González-Martín, Javier Rodríguez-Rivera, Jordi Ochando, Felipe Prosper, Cyril Moers, Nuria Montserrat

**Affiliations:** 1 Pluripotency for organ regeneration (PR Lab), Institute for Bioengineering of Catalonia, The Barcelona Institute of Science and Technology, Barcelona, Spain; 2 Department of Cell Biology, Physiology and Immunology, Faculty of Biology, University of Barcelona, Barcelona, Spain; 3 Instituto de Investigación Biomédica de A Coruña, Complexo Hospitalario Universitario A Coruña, Sergas, Universidad de A Coruña, A Coruña, Spain; 4 Regenerative Medicine and Hemato-Oncology Programs, Instituto de Investigación Sanitaria de Navarra, CIMA Universidad de Navarra, Pamplona, Spain; 5 Allergy Research Group, Instituto de Investigación Biomédica de Málaga y Plataforma en Nanomedicina–IBIMA Plataforma Bionand, Málaga, Spain; 6 Department of Surgery, University of California, San Francisco, San Francisco, CA USA; 7 Center for Bioengineering and Tissue Regeneration, University of California, San Francisco, San Francisco, CA USA; 8 Instituto de Investigación en Ciencias de la Alimentación, CSIC-UAM, Madrid, Spain; 9 Department of Surgery–Organ Donation and Transplantation, University Medical Center Groningen, University of Groningen, Groningen, the Netherlands; 10 Department of Morphology and Molecular Pathology, University Hospitals Leuven, Leuven, Belgium; 11 Departament de Bioquímica i Biomedicina Molecular, Institut de Biomedicina, Universitat de Barcelona and CIBER Fisiopatología de la Obesidad y Nutrición, Barcelona, Spain; 12 Transplant Immunology Unit, National Center of Microbiology, Instituto de Salud Carlos III, Madrid, Spain; 13 Instituto de investigación Sanitaria HM Hospitales, Madrid, Spain; 14 Department of Microbiology and Immunology, University of Leuven, Leuven, Belgium; 15 Department of Nephrology, University Hospitals Leuven, Leuven, Belgium; 16 Organización Nacional de Trasplantes, Madrid, Spain; 17 Centro de Investigación Biomédica en Red de Oncología and RICORS TERAV, Madrid, Spain; 18 Catalan Institution for Research and Advanced Studies, Barcelona, Spain

**Keywords:** Induced pluripotent stem cells, Differentiation

## Abstract

Organoids derived from human pluripotent stem (hPS) cells hold promise for therapeutic purposes. However, technological advances to overcome their massive production while ensuring differentiation fidelity are still lacking. Here we report a procedure sustaining the derivation of kidney organoids from hPS cells (hPSC-kidney organoids) using a scalable, reproducible and affordable approach that allows hPSC-kidney organoid differentiation into different renal cell types. Using single-cell RNA sequencing, confocal image analysis, metabolic assays and CRISPR–Cas9 engineering for generation of fluorescent reporters, we show that hPSC-kidney organoids exhibit transcriptional variety and cellular composition following cell-to-cell contact. We infuse human kidney organoids into ex vivo porcine kidneys using normothermic machine perfusion, and demonstrate in vivo engraftment of hPSC-kidney organoids. We further evaluate the immune response, confirming the feasibility and viability of the procedure. We identify cells of human origin after normothermic machine perfusion and in vivo transplantation by means of in situ hybridization, immunohistochemistry, confocal microscopy, image analysis and quantification, in vivo imaging, and flow cytometry. This work provides a foundation for using hPSC-kidney organoids for ex vivo cell-based therapies in clinical trials.

## Abstract (web summary)

Kidney organoids derived from human pluripotent stem cells are infused in a normothermic machine perfusion system to condition explanted porcine kidneys, and the feasibility and viability of their in vivo engraftment are demonstrated.

## Main

Organoid technology holds great promise for regenerative medicine applications. When developed from human pluripotent stem (hPS) cells, organoids have offered high fidelity at recapitulating important characteristics of human developing organs. In general, methodologies for organoid generation involve exploiting the inherent capacity of hPS cells to self-organize and respond to external biochemical stimuli, which ultimately promote their differentiation towards the desired organ. In the past years, other approaches have consisted in the introduction of physical constraints mimicking the embryonic milieu as an affordable methodology to externally guide hPS cell differentiation into organoids (for example, physiologically relevant conditions^1^ and stiffness^2^).

While the field has collectively demonstrated that hPS cell-derived organoids offer an unprecedented platform for modelling human development and disease, therapeutic studies aimed at translating this technology into clinically relevant applications remain largely elusive yet critically important. In part, this may be due to the difficulty of producing hPS cell-derived organoids under initial conditions that allow scalability, uniformity and reproducibility in a cost-effective manner, thus preventing the use of organoids in applications such as tissue regeneration and organ transplantation. Here, to explore the potential use of organoids in future clinical settings, we evaluated a differentiation toolkit to produce kidney organoids derived from hPS cells (hPSC-kidney organoids) in a reproducible manner by forcing cell-to-cell contact.

In this Article, we defined the specific conditions to derive uniform kidney organoids in a scalable manner, and we confirmed the results by means of confocal microscopy, metabolic analysis, image quantification and single-cell transcriptomics. We then demonstrated the utility of these organoids by validating the accuracy of our approach using pig kidneys subjected to ex vivo normothermic machine perfusion (NMP) with hPSC-kidney organoids, which were subsequently transplanted into the same pig for in vivo studies. These results validate the feasibility and the viability of hPSC-kidney organoids for ex vivo cell-based therapy in clinical trials.

## Results

### Generation of kidney organoids in free-floating culture

During mammalian development, the kidneys arise from the posterior intermediate mesoderm (PIM), which develops onto the metanephric mesenchyme (MM) whereas the anterior intermediate mesoderm (AIM) generates the ureteric bud (UB). While the MM will differentiate into nephron tubules, the glomerulus and the renal stroma owing to the presence of nephron progenitor cells (NPCs), the UB will give rise to the collecting duct and ureter. We previously demonstrated that increasing the time that hPS cell-derived PIM-committed cells are exposed to three-dimensional (3D) culture results in the generation of kidney organoids with higher features of differentiation. Here, we aimed to explore how forcing different numbers of cell-to-cell interactions would result in the generation of organoid derivatives exhibiting different extents of differentiation and cell composition. Such information would be relevant to identify initial culture conditions in which cell-fate determination can be predicted and conversely used to scale up robust organoid production.

**Fig. 1:**
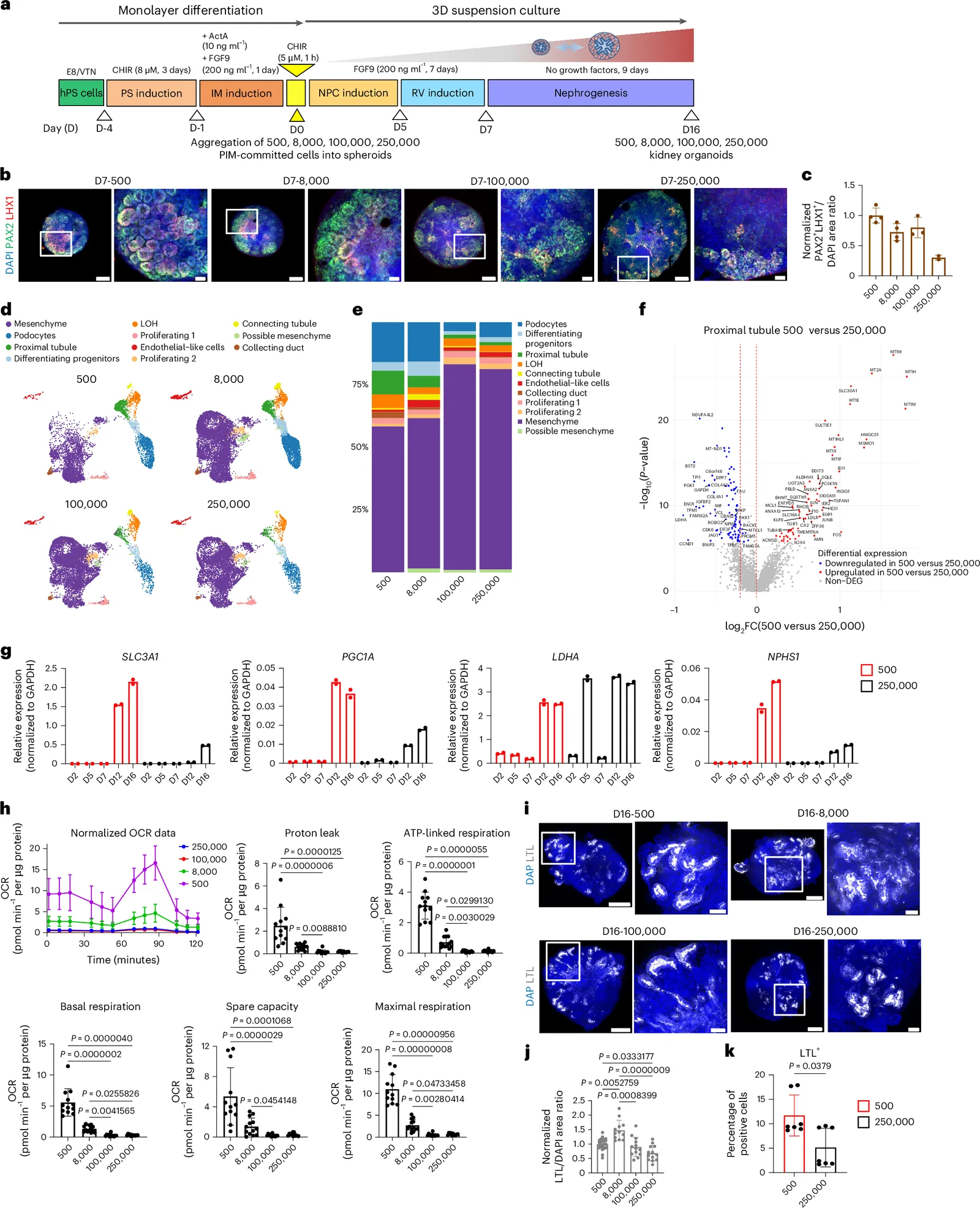
Generation of kidney organoids in suspension culture. **a**, Experimental scheme for the generation of human kidney organoids from hPS cells. **b**, Representative immunofluorescence staining for PAX2 (green), LHX1 (red) and DAPI (blue) in day (D) 7 RV-stage organoids derived from 500, 8,000, 100,000 and 250,000 IM-committed spheroids. Scale bars, 200 μm and 50 μm (magnified views). Images are representative of three independent experiments. **c**, Quantification of PAX2^+^LHX1^+^ area relative to DAPI in 500, 8,000, 100,000 and 250,000 organoids at D 7 of differentiation. Data are mean ± s.d. *n* = 4 (500), *n* = 4 (8,000), *n* = 3 (100,000), *n* = 2 (250,000) organoids. **d**, UMAP of kidney organoids split by conditions. Clusters are coloured by annotated cell types. LOH, loop of Henle. **e**, Cell type proportions in 500, 8,000, 100,000 and 250,000 kidney organoid samples at day 16 of differentiation. **f**, Differentially expressed genes (DEGs) in 500 organoids against 250,000 organoids considering only the proximal tubule cell type. In the volcano plot, the *x* axis indicates log_2_ fold change (FC) and the *y* axis indicates statistical significance with the −log_10_(*P* value) Genes with an adjusted *P* value <0.05 are considered upregulated (red) if the log_2_FC is >0.1 and downregulated (blue) if the log_2_FC is <−0.1. Non-DEGs are shown in grey. **g**, mRNA expression levels of *SLC3A1*, *SLC16A1*, *PGC1A*, *LDHA* and *NPHS1* during the time course of kidney organoid differentiation in 500 (red) compared with 250,000 (black) organoids. Data are mean ± s.d. (technical replicates). Each sample is a pool of 12 organoids per group with two technical replicates each. **h**, Seahorse analysis of 500, 8,000, 100,000 and 250,000 organoids at day 16 of differentiation. The OCR data are normalized to total protein. Inner mitochondrial membrane proton leak, cellular ATP production, basal respiration, spare respiratory capacity and maximal respiration are shown. Data are mean ± s.d. *n* = 12 (500), *n* = 13 (8,000), *n* = 12 (100,000), *n* = 11 (250,000) organoids from three independent experiments. Kruskal–Wallis (two-sided) followed by Dunn’s multiple-comparisons test. **i**, Representative immunofluorescence staining for LTL (greys) and DAPI (blue) in day 16 kidney organoids derived from 500, 8,000, 100,000 and 250,000 IM-committed spheroids. Scale bars, 200μm, 50μm (magnified views). Images are representative of three independent experiments. **j**, Quantification of LTL area relative to DAPI in 500, 8,000, 100,000 and 250,000 organoids at day 16 of differentiation. Data are mean ± s.d. *n* = 26 (500), *n* = 12 (8,000), *n* = 13 (100,000), *n* = 13 (250,000) organoids. Kruskal–Wallis (two-sided) followed by Dunn’s multiple-comparisons test. **k**, Quantification of the percentage of LTL^+^ proximal tubule cells in 500 and 250,000 kidney organoids by flow cytometry. Data are mean ± s.d. *n* = 7 biological replicates from a pool of 12 organoids each. Mann–Whitney test (two-sided). See also Extended Data Figs. 1–3. Source data

**Extended Data Fig. 1:**
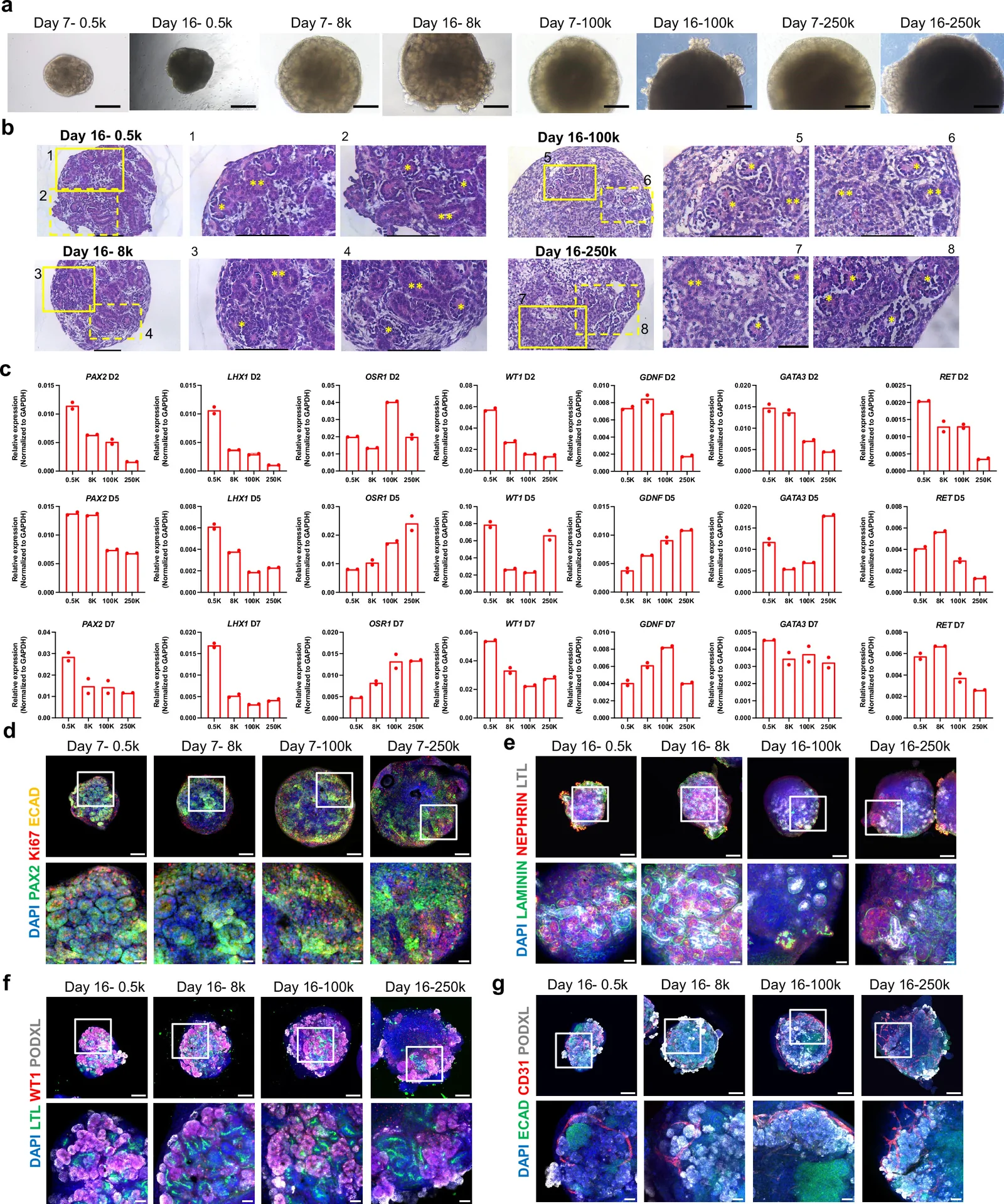
Characterization of kidney organoids derived from 0.5-, 8-, 100-, and 250-k PIM-committed spheroids. **a**, Representative bright-field images of day 7 and day 16 kidney organoids derived from 0.5-, 8-, 100-, and 250-k IM-committed spheroids. Scale bars, 250μm. **b**, Representative hematoxylin and eosin staining of day 16 kidney organoids derived from 0.5-, 8-, 100-, and 250-k IM-committed spheroids. Glomerular (*) and tubular (**) structures are shown. Scale bars, 100μm. **c**, mRNA expression levels of IM-, NPC- and UB-associated markers in day 2, 5 and 7 kidney organoids derived from the different aggregate conditions. Data are mean ± s.d. (technical replicates). Each sample is a pool of 12 organoids/group with two technical replicates each. **d**, Representative immunofluorescence staining for PAX2 (green), Ki67 (red), ECAD (grey) and DAPI (blue) in day 7 RV-stage organoids derived from 0.5-, 8-, 100-, and 250-k IM-committed spheroids. Scale bars, 200μm, 50μm (magnified views). **e,f,g**, Representative immunofluorescence staining in day 16 kidney organoids derived from 0.5-, 8-, 100-, and 250-k IM-committed spheroids for the detection of **e**, LAMININ (green), NEPHRIN (red), LTL (grey) and DAPI (blue). **f**, LTL (green), WT1 (red), PODXL (grays) and DAPI (blue). **g**, ECAD (green), CD31 (red), PODXL (grays) and DAPI (blue). Scale bars (e,f,g), 200μm, 50μm (magnified views). Images are representative of three independent experiments. Source data

**Extended Data Fig. 2:**
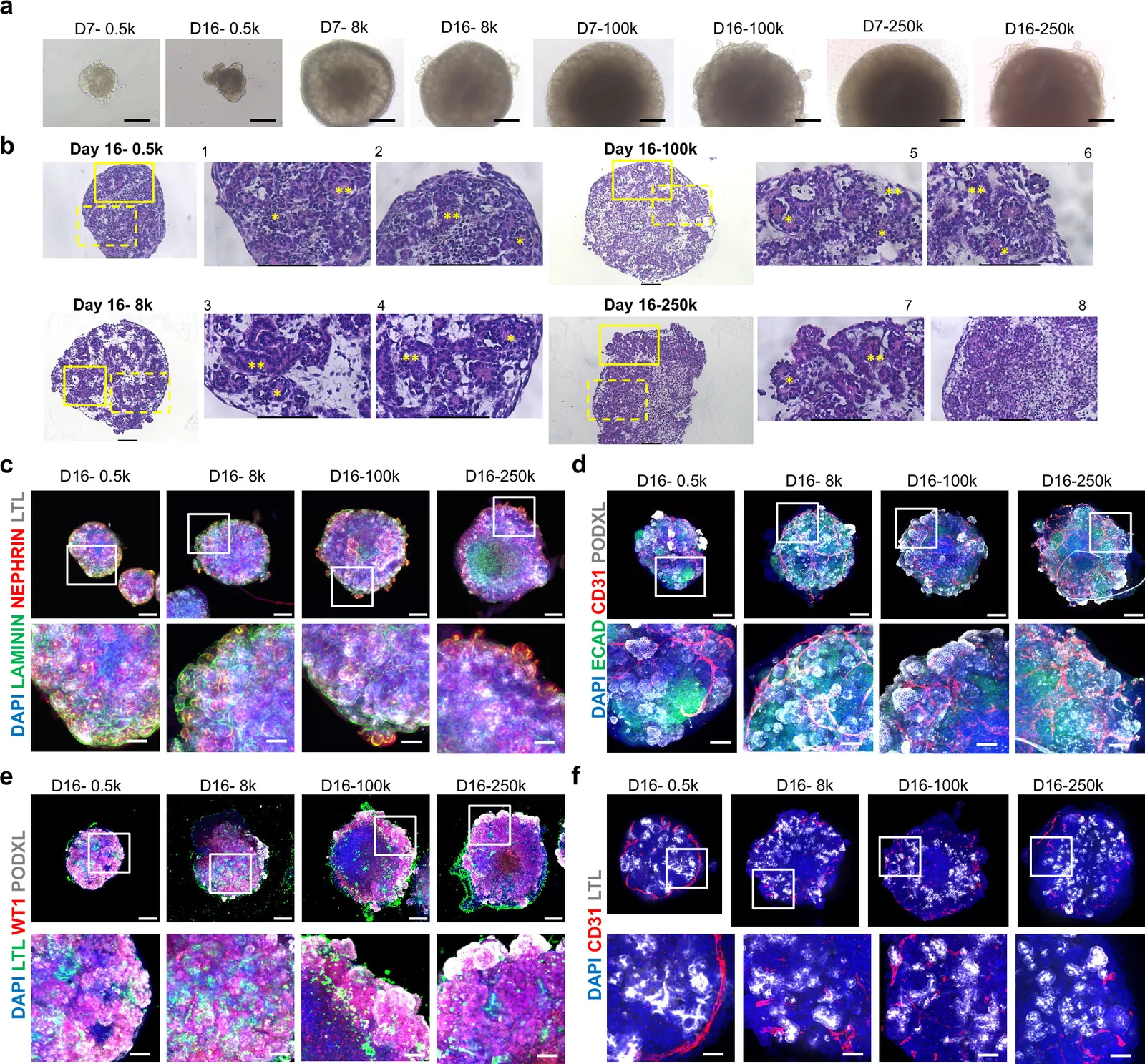
Generation of kidney organoids from hiPSC in free floating 3D culture conditions. **a**, Representative bright-field images of day 7 and day 16 hiPSC-derived kidney organoids generated from 0.5-, 8-, 100-, and 250-k IM-committed spheroids. Scale bars, 250 μm. **b**, Representative hematoxylin and eosin staining of day 16 hiPSC-derived kidney organoids generated from 0.5-, 8-, 100-, and 250-k IM-committed spheroids. Glomerular (*) and tubular (**) structures are shown. Scale bars, 100μm. **c-f**, Immunofluorescence analysis of day 16 hiPSC-derived kidney organoids from all aggregate conditions for the detection of the different nephron components and the endothelial cell compartment, including **c**, LAMININ (green), NEPHRIN (red), LTL (grays) and DAPI (blue). **d**, ECAD (green), CD31 (red), PODXL (grays) and DAPI (blue), **e**, LTL (green), WT1 (red), PODXL (grays) and DAPI (blue). **f**, CD31 (red), LTL (grays) and DAPI (blue). Scale bars (c-f), 200μm, 50μm (magnified views). Images are representative of three independent experiments.

Therefore, hPS cells were seeded at 1–2 × 10^5^ viable cells per well on vitronectin-coated 24-well plates and cultured in Essential 8 medium at 37 °C overnight. The next day (day −4), differentiation was induced by treating cell monolayer cultures with 8 μM CHIR99021 (CHIR) in Advanced RPMI 1640 basal medium for 3 consecutive days. On day −1, monolayer cultures were treated with 200 ng ml^−1^ FGF9, 1 μg ml^−1^ heparin and 10 ng ml^−1^ activin A for 24 h. On day 0, hPS-cell-PIM-committed cell monolayers were treated with CHIR (5 µM) for 1 h while maintaining FGF9 signalling and subsequently dissociated into single cells that were seeded in V-bottom 96-well plates to generate 3D spheroids by the self-aggregation of 500, 8,000, 100,000 and 250,000 PIM-committed cells (PIM-committed 3D spheroids) per well. The 3D spheroids were maintained in free-floating conditions until day 16 of differentiation (Fig. 1a; Methods) which led to the formation of kidney organoids (Extended Data Fig. 1a,b). FGF9 signalling was maintained from day 0 to day 7, promoting the formation of renal vesicles (RVs) (Fig. 1b; Methods). Further analyses were conducted to dissect the expression of markers of PIM, NPCs and RV by quantitative polymerase chain reaction (qPCR) at days 2, 5 and 7 (Extended Data Fig. 1c). In all the tested conditions, day 7 RV-stage organoids contained numerous RVs that expressed the RV-associated markers PAX2 and LHX1 as determined by immunofluorescence (Fig. 1b). Image quantification showed that day 7 organoids derived from 500 and 8,000 PIM-committed 3D spheroids developed more PAX2^+^LHX1^+^ RV structures (Fig. 1c), also exhibiting increased mRNA levels of *PAX2*, *LHX1* and *WT1* than those derived from 100,000 and 250,000 (Extended Data Fig. 1c). The presence of proliferative cells expressing Ki67 within the E-cadherin (ECAD)^+^PAX2^+^ RV-like structures was observed in all the conditions, as expected (Extended Data Fig. 1d). RV-stage organoids were further differentiated in the absence of growth factors (from day 7 to day 16) under free-floating culture conditions (Fig. 1a; Methods). For all the tested conditions (500, 8,000, 100,000 and 250,000), our approach led to the generation of kidney organoids with multiple nephron-like structures detected by morphological analysis using haematoxylin and eosin staining (Extended Data Fig. 1b). Immunofluorescence and confocal microscopy confirmed that the different structures were segmented into typical nephron components, including glomeruli with podocyte-like cells (PODXL^+^CD31^−^ECAD^−^/PODXL^+^WT1^+^LTL^−^/NEPHRIN^+^LTL^−^), proximal tubule-like (LTL^+^PODXL^−^WT1^−^), distal tubule-like and loop of Henle-like (ECAD^+^PODXL^−^CD31^−^) structures and endothelial-like cells (CD31^+^ECAD^−^PODXL^−^) (Extended Data Fig. 1e–g). Our methodology for the derivation of kidney organoids in free-floating culture conditions was robust and efficient and was extended to additional hPS cell lines (Extended Data Fig. 2a–f).

### Transcriptomic and phenotypic landscapes in kidney organoids

**Extended Data Fig. 3:**
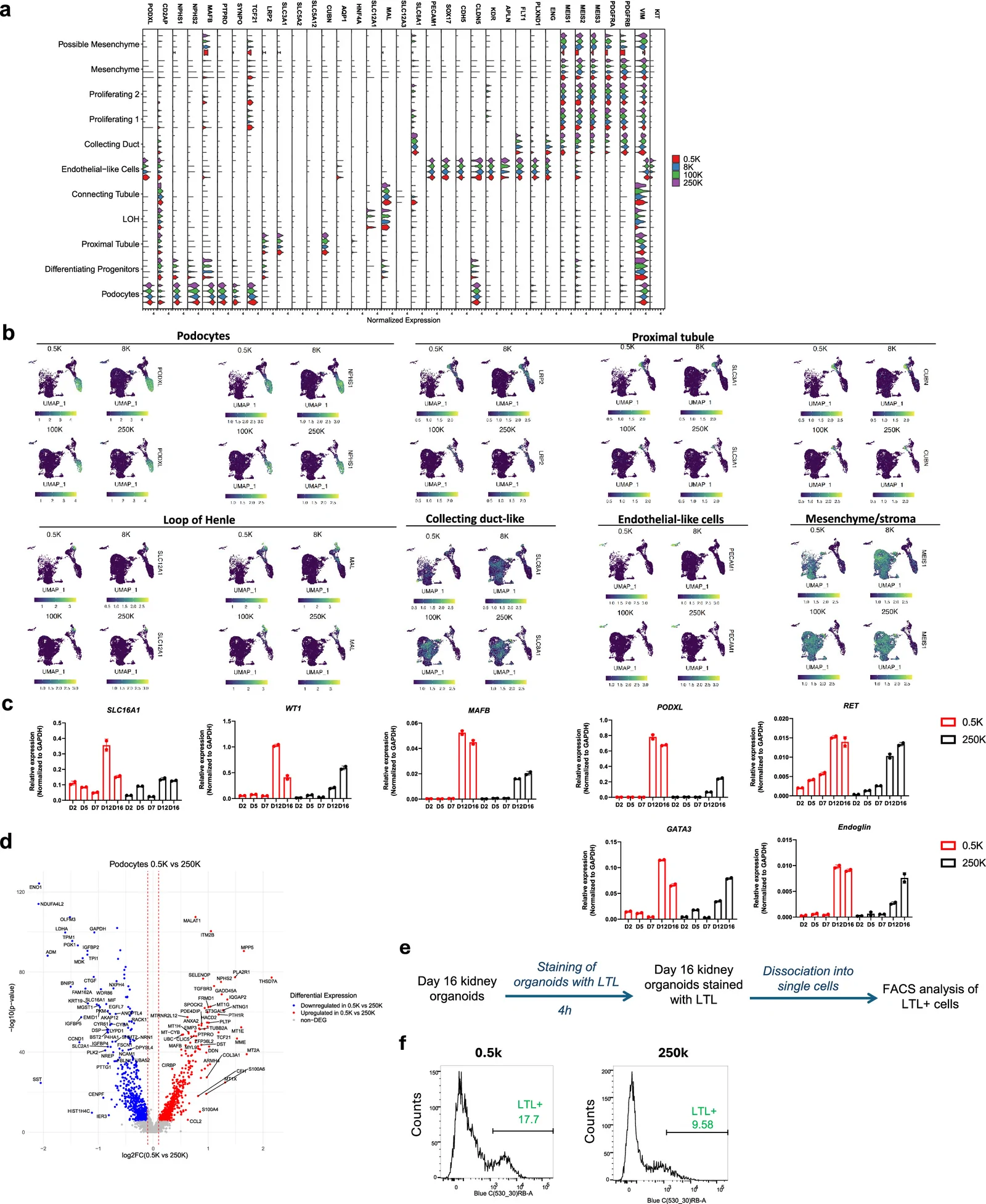
Single-cell RNA-seq profiling of kidney organoids derived from 0.5-, 8-, 100-, and 250-k PIM-committed spheroids. **a**, Violin plots representing log-normalized UMI gene expression levels for known renal lineage-associated genes (columns) for the different cell types indicated (rows), split by conditions. **b**, UMAPs indicating the log-normalized expression for selected nephron, collecting duct, endothelial and stromal key marker genes. Color intensity is scaled per gene. **c**, mRNA expression levels of *SLC16A1*, *WT1, MAFB, PODXL, RET, GATA3* and *ENDOGLIN* during the time course of kidney organoid differentiation in 0.5-k (red) compared to 250-k (black) organoids. Data are mean ± s.d. (technical replicates). Each sample is a pool of 12 organoids/group with two technical replicates each. **d**, Differentially expressed genes (DEGs) in 0.5-k organoids against 250-k organoids considering only the podocyte cell types. In the volcano plot, the x-axis indicates log2 fold change (FC) and the y-axis indicates statistical significance with the –log10(p-value) Genes with an adjusted p-value < 0.05 are considered upregulated (red) if the log2FC is > 0.1 and downregulated (blue) if the log2FC < is -0.1. Non DEG are shown in grey. **e**, Kidney organoids were stained with fluorescein-conjugated LTL (FL-1321, Vector Laboratories) to label proximal tubule cells, dissociated to single cells and analyzed by flow cytometry (FACS). **f**, Representative histograms representing the overlay of live cells and LTL positive cells from seven independent cell sorting experiments. Source data

To gain insight in the sequence of transcription-regulating events differentially expressed in response to cell-to-cell aggregation in PIM-committed spheroids, we performed single-cell RNA sequencing (scRNA-seq). Analysis was performed across four biological conditions (500, 8,000, 100,000 and 250,000 hPSC-kidney organoids at day 16). Cell types were assigned using unsupervised clustering after integrating all the assayed conditions, and the Uniform Manifold Approximation and Projection (UMAP) algorithm was used to visualize the scRNA-seq data (Fig. 1d). We retrieved renal endothelial-like, mesenchymal-like, proliferating, podocyte-like and tubule-like cell populations in all four experimental samples based on previous findings by us^3–5^ and others^6^. These observations indicate that starting seeding conditions did not alter the resulting cell composition across all four biological samples (Fig. 1d and Extended Data Fig. 3a). Importantly, we observed that the corresponding proportion for each cluster was discrepant in kidney organoids derived from 500 and 8,000 intermediate mesoderm (IM)-committed 3D spheroids that were composed of a large proportion of podocyte-, endothelial- and tubular-like cells, compared with those derived from organoids generated from 100,000 and 250,000 IM-committed 3D spheroids (Fig. 1e). Importantly, kidney organoids derived from 500 and 8,000 IM-committed 3D spheroids showed less abundancy of stromal-like cells than those derived from 100,000 and 250,000 (Fig. 1e), also highlighting a higher degree of differentiation as stromal cell diminution is associated with kidney maturation during development^7,8^. These observations correlated with changes in the expression of genes for the different kidney compartments (Extended Data Fig. 3a–c). In addition, all the assayed conditions led to the generation of AIM-derived cells, including collecting duct-like cells as highlighted by scRNA-seq analysis (Fig. 1d,e) and qPCR analysis (Extended Data Fig. 3c).

Differential gene expression analysis showed increased expression of metallothioneins in tubular-like cells of kidney organoids derived from 500 PIM-committed 3D spheroids compared with 250,000, which have been previously shown to maintain proximal tubule homeostasis during growth and development also offering protection against toxic metals and oxidative stress (Fig. 1f). These results highlight the overall applicability of the approach described here for investigating heavy-metal-induced nephrotoxicity responses in human kidney organoids—an area that remains largely unexplored as a functional assay in the field^9^. Our results also showed that podocyte-like cells (Extended Data Fig. 3d) exhibited higher expression levels of podocyte markers such as *TCF21*, *NPHS2* and *MAFB* as well as integral membrane protein 2B (ITM2B), a core component of the slit-diaphragm associated network. Furthermore, we observed an increase in the expression of *PGC1A*, a master regulator of mitochondrial biogenesis and oxidative phosphorylation (OXPHOS), in kidney organoids derived from 500 compared with 250,000 PIM-committed 3D spheroids (Fig. 1g). Conversely, a decrease in the expression of the glycolytic enzyme *LDHA* was also detected in the time course of organoid generation (Fig. 1g). Interestingly, we have previously shown that both fatty acid oxidation and OXPHOS drive proximal tubular differentiation in hPSC-kidney organoids^3^ and that forcing metabolic programming represents an affordable strategy to promote tubular differentiation^3^, as recently confirmed by others in the field^10^. In the present study, we observed that an increase in the mRNA levels of *PGC1A* also correlates with an elevated expression of proximal tubular cell markers (*SLC3A1* and *SLC16A1*) (Fig. 1g and Extended Data Fig. 3c). qPCR analysis furthermore highlighted that kidney organoids derived from 500 PIM-committed 3D spheroids exhibited a higher expression of markers for glomeruli (*WT1*, *NPHS1*, *MAFB* and *PODXL*), tubuli (*SLC16A1*) and endothelium (*ENDOGLIN*) compared with 250,000 during differentiation (Extended Data Fig. 3c). We have previously shown that promoting specific energy metabolism profiles during hPSC-kidney organoid differentiation enhances the differentiation of hPS cells into renal subtypes^2,3^. To further investigate the impact of cell-to-cell aggregation on the energetic metabolism of hPSC-kidney organoids, we performed Seahorse analysis on day 16 of differentiation. Our approach identified that day 16 hPSC-kidney organoids derived from 500 and 8,000 PIM-committed 3D spheroids exhibited an OXPHOS bioenergetic phenotype compared with 100,000 and 250,000 (Fig. 1h). The results provided here suggest that promoting cell-to-cell contact represents an affordable approach to induce an OXPHOS-mediated metabolic profile driving proximal tubular differentiation at the transcriptomic and phenotypic level. This is highlighted by the detection of more proximal tubular structures (Lotus tetragonolobus lectin (LTL)^+^) in kidney organoids derived from 500 and 8,000 PIM-committed 3D spheroids compared with 100,000 and 250,000 by image quantification (Fig. 1i,j) and fluorescence-activated cell sorting (FACS; Fig. 1k and Extended Data Fig. 3e,f).

### Scalable production of uniform kidney organoids by microaggregation

Previous studies have shown that the aggregation of patterned cells derived from hPS cells can be used in the production of organoids^11–15^. Based on our observations that the aggregation of 500 PIM-committed cells led to the generation of kidney organoids with higher transcriptomic and phenotypic traits of differentiation compared with the other assayed conditions, we explored suitable conditions to systematically generate PIM-committed spheroids.

**Fig. 2:**
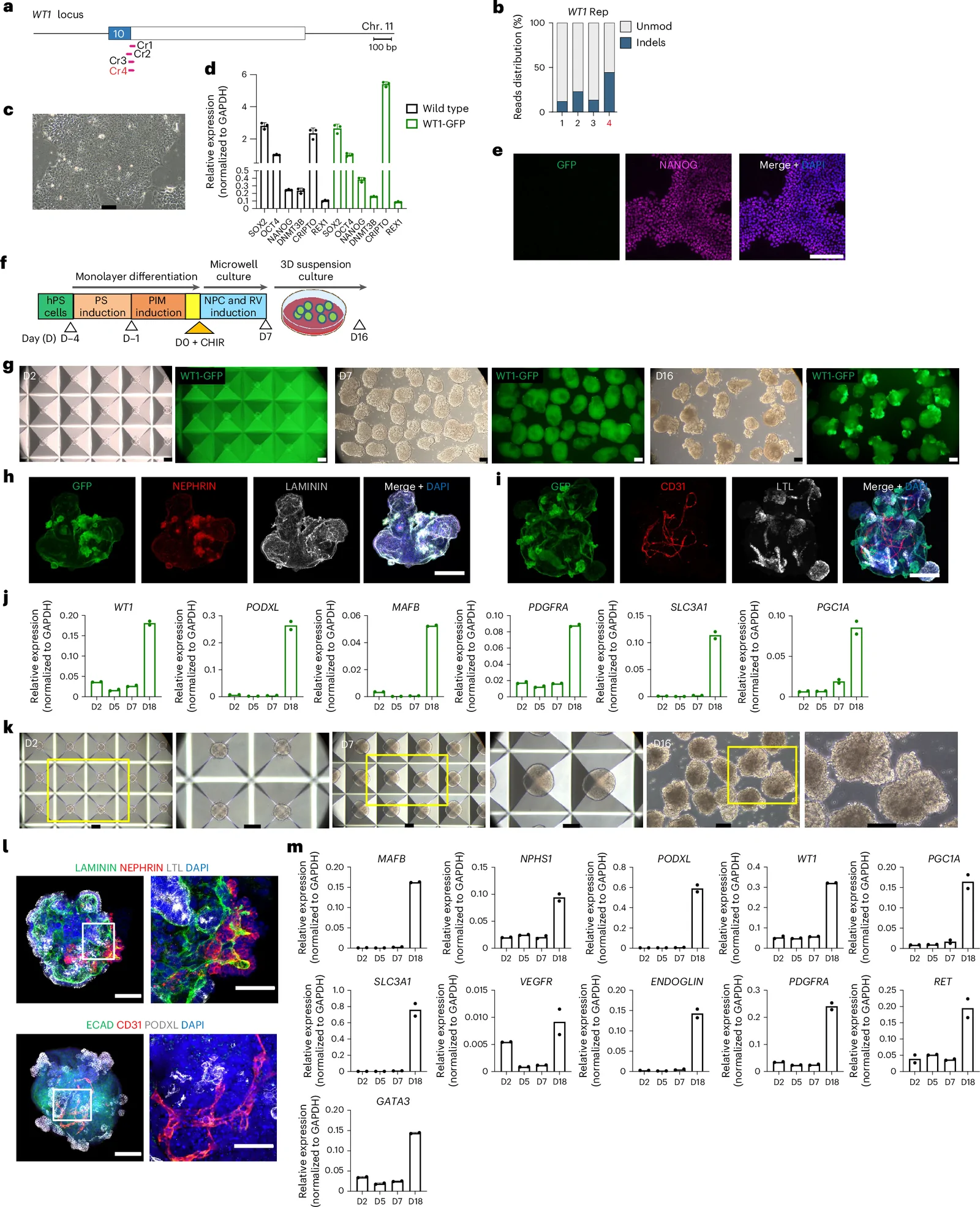
Scalable production of uniform kidney organoids by microaggregation. **a**, Schematic representation of the *WT1* gene locus where sgRNAs were designed. Four sgRNAs were targeted to sequences around the *WT1* stop codon at exon 10 and screened for editing efficiency by MiSeq. **b**, Quantification of editing efficiencies for each sgRNA is represented as a distribution of reads carrying Indels (% blue) or unmodified (Unmod) reads (% grey). The sgRNA leading to the highest efficiency of genome editing is highlighted in red. **c**, Representative bright field image of WT1-GFP reporter hPS cell line. Scale bar, 100 μm. Images are representative of three independent experiments. **d**, mRNA expression levels of *SOX2*, *OCT4*, *NANOG*, *DNMT3B*, *CRIPTO* and *REX1* in the WT1-GFP reporter hPS cell line (green) compared with the wild-type hPS cell line (black). Data are mean ± s.d. (technical replicates). Each sample is a pool of two wells of hPS cells with three technical replicates each. **e**, Representative immunofluorescence staining for GFP (green), NANOG (magenta) and DAPI (blue) in undifferentiated WT1-GFP reporter hPS cell colonies. Scale bar, 200 μm. Images are representative of three independent experiments. **f**, Experimental scheme for the generation of uniform 500 kidney organoids from the WT1-GFP reporter hPS cell line using the microwell culture system. **g**, Representative bright-field and fluorescent images of kidney organoids derived from the WT1-GFP reporter hPS cell line by the microwell culture system during the time course of differentiation (days are indicated). Images are representative of three independent experiments. **h**,**i**, Representative immunofluorescence staining in day 16 kidney organoids derived the WT1-GFP reporter hPS cell line using the microwell culture system for the detection of GFP (green), NEPHRIN (red), LAMININ (greys) and DAPI (blue) in **h**, and GFP (green), CD31 (red), LTL (greys) and DAPI (blue) in **i**. Scale bars, 200 μm (**h** and **i**). Images are representative of three independent experiments. **j**, mRNA expression levels of *WT1*, *PODXL*, *MAFB*, *PDGFRA*, *SLC3A1* and *PGC1A*, during the time course of kidney organoid differentiation in 500 organoids generated by the microwell culture system. Data are mean ± s.d. (technical replicates). Each sample is a pool of 12 organoids with two technical replicates each. **k**, Representative bright-field images of kidney organoids derived from hES cells by the microwell culture system on days 2, 7 and 16 of differentiation. Scale bars, 100 μm. Images are representative of three independent experiments. **l**, Representative immunofluorescence staining of day 16 kidney organoids derived from hES cells using the microwell culture system for the detection of LAMININ (green), NEPHRIN (red), LTL (greys) and DAPI (blue), and ECAD (green), CD31 (red), PODXL (greys) and DAPI (blue). Scale bars, 200 μm. Images are representative of three independent experiments. **m**, mRNA expression levels of renal differentiation markers during the time course of kidney organoid differentiation in 500 organoids generated by the microwell culture system. Data are mean ± s.d. (technical replicates). Each sample is a pool of 12 organoids with two technical replicates each. See also Extended Data Fig. 4. Source data

**Extended Data Fig. 4:**
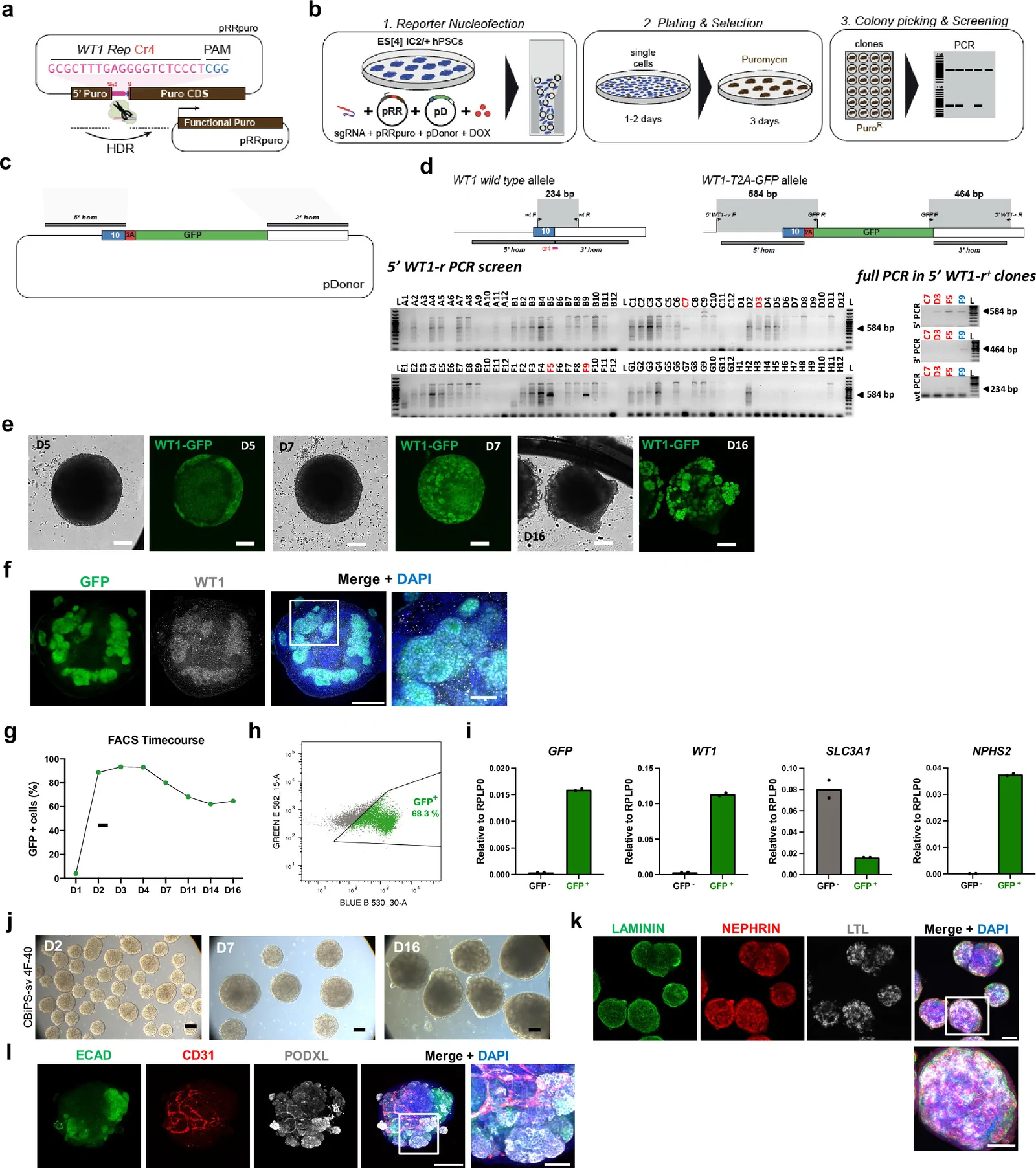
Generation of WT1-GFP reporter hPSC line using an inducible CRISPR/Cas9 system in hPSCs. **a**, Schematics for the pRR-Puro plasmid used as a selector of HDR-mediated insertions. This vector contains the WT1 KI-R Cr4 sgRNA protospacer sequence together with the corresponding PAM sequence flanked by a prematurely terminated 5’ end puromycin (puro)-resistance sequence and a complete puromycin coding sequence (CDS) with a stop codon (UAA) replacing the start codon. After the introduction of DSB by WT1 KI-R Cr4/Cas9 complexes, HDR between the homologous stop codon sequences results in the generation of functional puro-resistance expression. **b**, Workflow scheme for the generation of ES[4] WT1-T2A-GFP reporter cell line. First, the sgRNAs, the pDonor plasmid (pD) and the pRR-puro plasmid (pRR) were co-nucleofected in DOX- treated ES[4] iC2/+ 51. After nucleofection, cells are plated in the absence of puromycin as single cells to allow clonal colony growth. After 48 hours, cells are treated with puromycin to select the resistant targeted cells. Resistant colonies are manually picked based on morphology and genomic DNA is subsequently extracted for PCR colony screening. **c**, Schematics of the pDonor plasmid (WT1-T2A-GFP donor plasmid). Cloning into the pD empty vector was performed using restriction digestion followed by T4 ligation of relevant DNA fragments including PCR products from WT1 5’ and 3’ homology arms at each side of WT1 stop codon and a PCR product from a T2AGFP expression cassette. **d**, Representation of the PCR genotyping strategy to identify WT1 wild-type (wt) and WT1-T2A-GFP targeted alleles. The wt PCR primers were designed within the 5’ and 3’ homology arms (HA) of the WT1 locus, and the expected product was 234bp. The PCR primer pairs to identify the 5’ and 3’ ends of the WT1-T2A-GFP integrated sequence were designed outside the 5’ and 3’ homology arms (HA) and within the GFP sequence. The expected sizes for the 5’ and 3’ PCR products were 584bp and 464bp, respectively. After this first screening positive clones were subjected to a second PCR screening in which wt, 5’ and 3’ PCRs were performed. Only one clone, clone F9, was positive for both 5’ and 3’ PCR, indicating the correct integration of the donor plasmid. **e**, Live imaging of kidney organoids generated from the WT1-GFP reporter hPSC line at day 5, 7 and 16 of differentiation for the detection of the GFP reporter expression. Scale bars, 200μm. **f**, Representative immunofluorescence staining for GFP (green), WT1 (grays), and DAPI (blue) in day 16 kidney organoids derived from the WT1-GFP reporter hPSC line. Scale bars, 200μm, 50μm (magnified view). **g**, Quantification of GFP^+^ cells during the time course of kidney organoid differentiation from the WT1-GFP reporter hPSC line by FACS analysis. **h**, Gating strategy for cell sorting of GFP^+^ cells from day 16 kidney organoids derived from the WT1-GFP reporter hPSC line. Numbers in outlined area indicate percent GFP^+^ cells. **i**, mRNA expression levels of *GFP*, *WT1*, *SLC3A1* and *NPHS2* in the sorted GFP^+^ and GFP^−^ cell fractions from day 16 kidney organoids derived from the WT1-GFP reporter hPSC line. Data are mean ± s.d. (technical replicates). Each sample is a pool of 12 organoids/group with two technical replicates each. **j**, Representative bright field images of 0.5-Ks kidney organoids derived from hiPSC by the microwell culture system on day 2, 7 and 16 of differentiation. Scale bars, 100μm. **k,l**, Representative immunofluorescence staining of day 16 hiPSC-kidney organoids generated by the microwell culture system for the detection of LAMININ (green), NEPHRIN (red), LTL (grays) and DAPI (blue) in (k), and ECAD (green), CD31 (red), PODXL (grays) and DAPI (blue) in (l). Scale bars (k, l), 200μm, 100μm (magnified view in k), 50μm (magnified view in l). Images are representative of three independent experiments. Source data

For that, we explored the use of additional cell lines for the generation of kidney organoids in a scalable and robust manner by creating a fluorescent reporter cell line mirroring the endogenous expression of WT1, a marker of NPCs and nascent podocytes during kidney development. We took advantage of our previously described inducible CRISPR–Cas9 system in hPS cells^5,16,17^ (Fig. 2a,b and Extended Data Fig. 4a–d; Methods). We selected the targeted WT1-GFP clone based on PCR screen (Extended Data Fig. 4d; Methods). The established WT1-GFP reporter cell line exhibited the typical hPS cell-associated morphology (Fig. 2c) as well as the expression of major pluripotency-related markers as confirmed by qPCR analysis and immunofluorescence (Fig. 2d,e). The WT1-GFP reporter cell line conveyed WT1 expression dynamics as green fluorescent protein (GFP) expression was detected by live imaging (Extended Data Fig. 4e). Immunofluorescence analysis of day 16 kidney organoids showed that GFP expression mirrored endogenous WT1 expression in podocyte-like cells within the organoids (Extended Data Fig. 4f). In addition, flow cytometry analysis during kidney organoid generation confirmed the presence of GFP^+^ cells from day 1, corresponding to NPC induction, continuing its expression in day 7 RV-stage organoids until day 16 kidney organoids (Extended Data Fig. 4g). qPCR analysis quantifying mRNA expression of *GFP*, podocyte markers *WT1* and *NPHS2*, and the proximal tubule marker *SLC3A1* in GFP^+^ and GFP^−^ sorted cell populations from day 16 kidney organoids (Extended Data Fig. 4h,i) confirmed that GFP^+^ cells had a higher expression of *WT1* and *NPHS2* compared with GFP^−^ cells (Extended Data Fig. 4i). The latter showed increased expression of the proximal tubule marker *SLC3A1* (Extended Data Fig. 4i), overall indicating that GFP reporter expression correlated with endogenous WT1 expression in podocyte-like cells within day 16 kidney organoids. In light of these results, we established cell culture conditions to scale up kidney organoid production using commercially available AggreWell 400 Microwell plates containing 1,200 × 400 μm microwells per well of a 24-well plate (Fig. 2f; Methods). In our approach, PIM-committed cell monolayers (day 0) prepared from the WT1-GFP reporter cell line were first treated with 5 μM CHIR, 200 ng ml^−1^ of FGF9 and 1 μg ml^−1^ of heparin for 1 h. Next, cell monolayers were disaggregated into a single-cell suspension and plated at 6 × 10^5^ cells per well of a 24-well AggreWell 400 Microwell plate in AdvRPMI medium supplemented with 200 ng ml^−1^ of FGF9 and 1 μg ml^−1^ of heparin (corresponding to 5 × 10^2^ cells in each microwell). Following centrifugation and overnight culture, uniform cell aggregates with an approximate diameter of ∼100 μm were formed in each microwell (Fig. 2g). The generated microaggregates were then left to differentiate in the presence of FGF9 signalling from day 0 to day 7, when RV-like structures developed (Fig. 2g). Day 7 microorganoids were then transferred to low-adherent p10 plates and maintained in the absence of growth factors until day 16 when kidney organoids exhibited multiple nephron-like structures segmented into typical nephron components: glomeruli (NPHS1^+^GFP^+^LTL^−^), proximal tubuli (LTL^+^NPHS1^−^GFP^−^) and endothelial cells (CD31^+^GFP^−^LTL^−^), together with the presence of basement membrane protein LAMININ that was used to detect all the epithelial-, mesothelial- and endothelial-like structures (Fig. 2h,i). These findings were confirmed by qPCR analysis during the time course of kidney organoid generation (Fig. 2j). Moreover, this methodology was used for the generation of day 16 kidney organoids from two additional hPS cell lines, including human embryonic stem (hES) cells (Fig. 2k–m) and human induced pluripotent stem (iPS) cells (Extended Data Fig. 4j–l). Kidney organoids derived from both hPS cell lines contained segmented nephron-like structures at day 16 of differentiation, including glomeruli (NEPHRIN^+^LTL^−^/ECAD^−^PODXL^+^), proximal tubules (LTL^+^NEPHRIN^−^), distal tubules with loops of Henle (PODXL^−^ECAD^+^) and endothelial cells (CD31^+^ECAD^−^PODXL^−^) (Fig. 2l and Extended Data Fig. 4k,l), confirming that this approach was highly reproducible across hPS cell lines and allowed the production of kidney organoids in a scalable manner.

### Human kidney organoids engraft in porcine kidneys during ex vivo NMP

**Extended Data Fig. 5:**
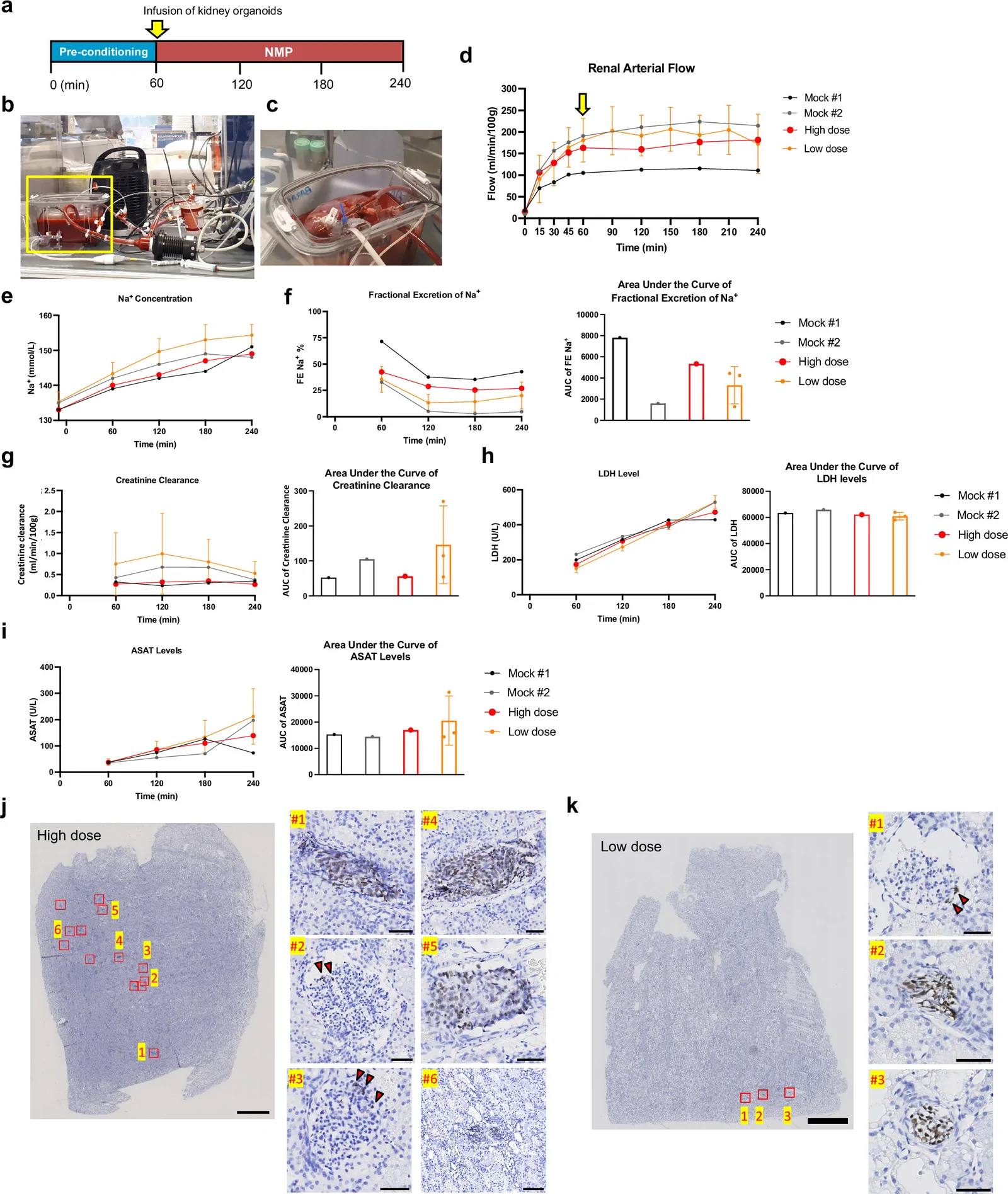
Infusion of intact kidney organoids during *ex vivo* NMP in porcine kidneys. **a**, Experimental scheme for infusion of kidney organoid during *ex vivo* NMP in porcine kidneys. **b**, Photograph of the homemade NMP setup used during porcine kidney perfusion. **c**, Photograph of a porcine kidney during NMP. **d**, Arterial flow (mL/min/100 g) of the three experimental groups during NMP (mean ± s.d.), mock *n=* 2 kidneys, high dose *n=* 1 kidney, low dose *n=* 3 kidneys. The yellow arrow indicates the time of organoid infusion. **e**, Sodium concentration (Na+) (mmol/L). **f**, Fractional excretion (FE) of sodium (FENa+) (%) and area-under-the curve (AUC). **g**, Creatinine clearance (mL/min/100g) and AUC. **h**, Lactate dehydrogenase (LDH) levels (U/L) and AUC. **i**, Aspartate aminotransferase (ASAT) levels (U/L) and AUC. In **d-i**, Data are mean ± s.d. mock *n=* 2 kidneys, high dose *n=* 1 kidney, low dose *n=* 3 kidneys. **j,k**, Detection of Alu repeat sequences of human DNA present in formalin fixed paraffin sections of porcine renal cortex by *in situ* hybridization. Images correspond to kidneys after infusion of intact kidney organoids during *ex vivo* NMP at high (j) and low (k) dose, respectively. n = 3 cortical punch biopsies were analyzed from one experiment at high dose and three independent experiments at low dose, with similar results. Arrowheads indicate Alu^+^ cells in glomerular structures. Scale bars, 2mm, 50μm (magnified views). Source data

**Extended Data Fig. 6:**
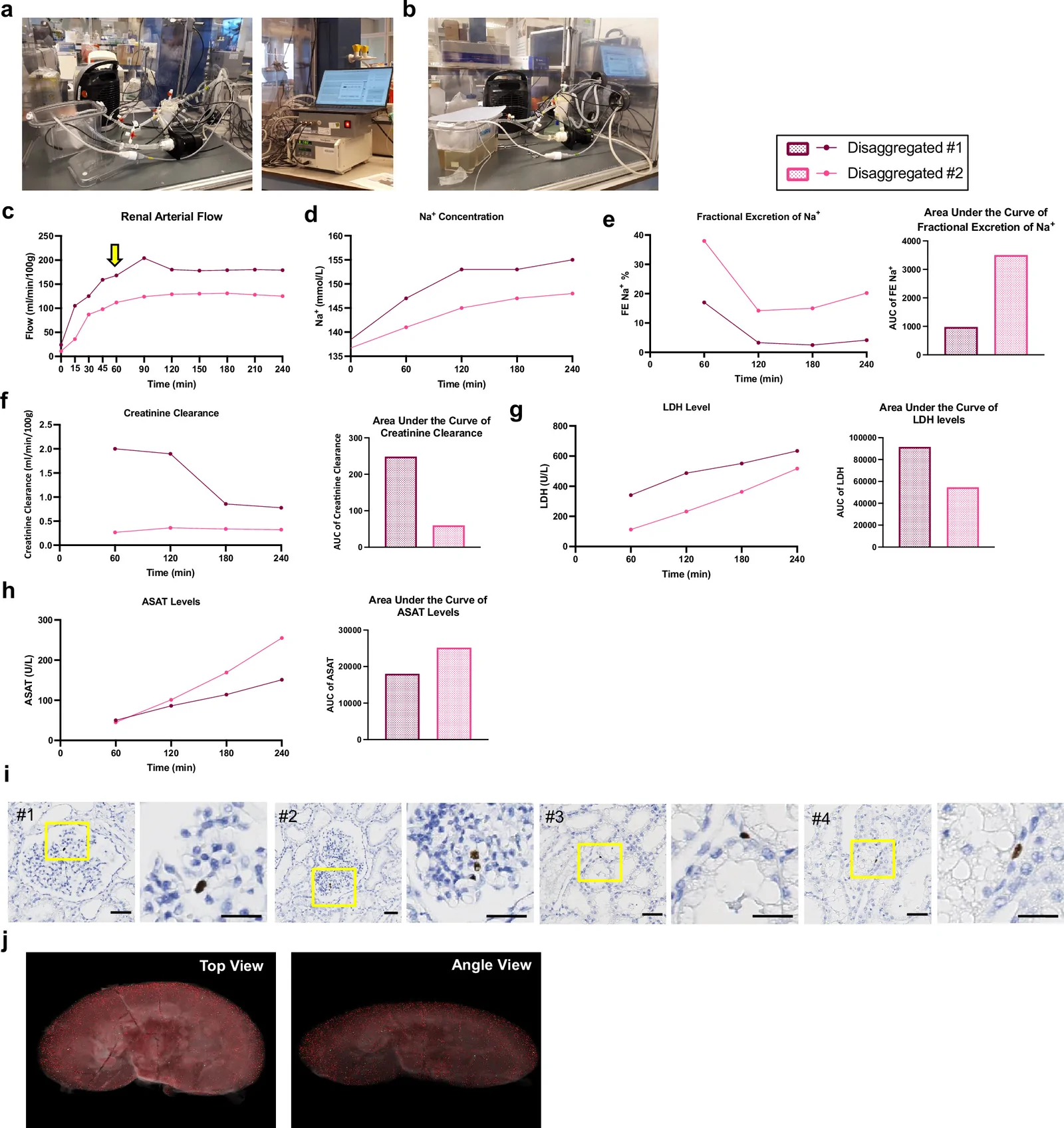
Infusion of kidney organoid-derived cells during *ex vivo* NMP in porcine kidneys. **a,b**, Photographs of the homemade NMP setup used during porcine kidney perfusion. **c**, Arterial flow (mL/min/100 g) of kidneys infused with organoid disaggregated cells during NMP. The yellow arrow indicates the time of organoid infusion. **d**, Sodium concentration (mmol/L)**. e**, Fractional excretion (FE) of sodium (FENa+) (%) and area-under-the curve (AUC). **f**, Creatinine clearance (mL/min/100g) and AUC. **g**, Lactate dehydrogenase (LDH) levels (U/L) and AUC. **h**, Aspartate aminotransferase (ASAT) levels (U/L) and AUC. In **c-h**, Data are mean ± s.d. *n=* 2. **i**, Detection of Alu repeat sequences of human DNA present in formalin fixed paraffin sections of porcine renal cortex by *in situ* hybridization. Images correspond to kidneys after infusion of disaggregated kidney organoid cells during *ex vivo* NMP. Scale bars, 50μm, 25μm (magnified views). n = 3 cortical punch biopsies were analyzed from two independent experiments, with similar results. **j**, Detected Qdot-positive cells (pseudocolored in red) and rendered along with brightfield volume of a 400 μm-thick slice from one kidney infused with dissociated kidney organoid cells. Source data

Infusion of hPS cell-derived NPCs or hPSC-kidney organoids in animal models have shown considerable improvements of organoid glomerular and tubular structures and the formation of endothelial networks^18–20^, as well as inducing different degrees of host vascularization and the diminution of off-target cells^2,20–22^. Despite these advances, the reliance on animal hosts or sophisticated bioengineering techniques to improve organoid vascularization and maturation limits their scalability. In light of the potential to scale up kidney organoid production using a microwell culture system that ensures reproducible differentiation, we next aimed to evaluate our differentiation pipeline for future therapeutic applications in kidney regeneration. To that end, we further leveraged the window of opportunity provided by NMP, during which the kidney can be continuously monitored and functionally assessed while potential therapies are administered ex vivo. We and others have previously assessed ex vivo hypothermic (1–8 °C) and normothermic (35–37 °C) machine perfusion to improve organ preservation and reduce ischaemia–reperfusion injury by either circulating a cold preservation fluid or a warm oxygenated blood-like perfusate through porcine kidneys before transplantation^23–29^. Because our differentiation methodology using a microwell culture system allows scalability in the generation of organoids, intact hPSC-kidney organoids were injected in the renal artery of viable porcine kidneys (sow; type: Topigs 20) from local slaughterhouse during NMP at different concentrations (Methods). Based on previous findings^23–29^, the organs were perfused with an oxygenated perfusate, containing red blood cells, albumin, electrolytes and nutrients at normal body temperature and a sinusoidal pulsatile (60 bpm) pressure of 110/70 mm Hg for up to 4 h to maintain a near-physiological microenvironment (Extended Data Fig. 5a–c; Methods). During the first 60 min of NMP, renal arterial flow increased in all the experimental groups, including the vehicle group (mock conditions represent the infusion of perfusate in the absence of organoids) (Extended Data Fig. 5d). Thereafter, flow rates mostly remained constant until the end of the experiment (240 min) with no substantial differences in flow progression, suggesting no major differences in vascular resistance over time between groups (Extended Data Fig. 5d). Notably, organoid infusion through the renal artery of the organ 60 min after NMP did not have a major effect on renal blood flow (Extended Data Fig. 5d). Metabolic and vasomotor activity of organoid-infused kidneys during NMP was preserved, as shown by maintained levels of total sodium (Extended Data Fig. 5e), fractional excretion of sodium (Extended Data Fig. 5f) and creatinine clearance (Extended Data Fig. 5g) in all groups. In addition, lactate dehydrogenase (LDH) and aspartate aminotransferase (ASAT) levels during NMP were analysed as markers for general cell injury and toxicity and showed similar levels between all groups (Extended Data Fig. 5h,i). To confirm delivery and structural integrity of hPSC-kidney organoids or organoid-derived cells, porcine kidney biopsies after NMP were analysed for the presence of Alu repeat sequences of human DNA by in situ hybridization, showing that Alu^+^ cells were present in both glomerular and blood vessel structures (Extended Data Fig. 5j,k). Notably, our experimental set-up succeeded in delivering both organoids (Extended Data Fig. 5a–k) and enzymatically dissociated organoid-derived cells into the renal artery (Extended Data Fig. 6a–j), the latter showing a homogeneous distribution of fluorescence Qdot-labelled infused cells in whole kidneys after NMP (Extended Data Fig. 6j). Our results provide the proof of principle that ex vivo perfused porcine kidneys can be used to ascertain engraftment of hPSC-kidney organoids and derived cells.

### Human kidney organoids persist in porcine kidneys after in vivo transplantation

The first report of organoid infusions during NMP was performed in human discarded livers using dissociated organoid-derived cells^30^. Here, we took the additional step towards the clinical applicability of hPSC-kidney organoids by transplanting the pig kidney after infusion of hPSC-kidney organoids into the same animal through vascular anastomosis, using a previously described porcine model^31,32^. Laboratory pigs (Hybrid Landrace/Large White crossbreed) were used (Methods).

**Extended Data Fig. 7:**
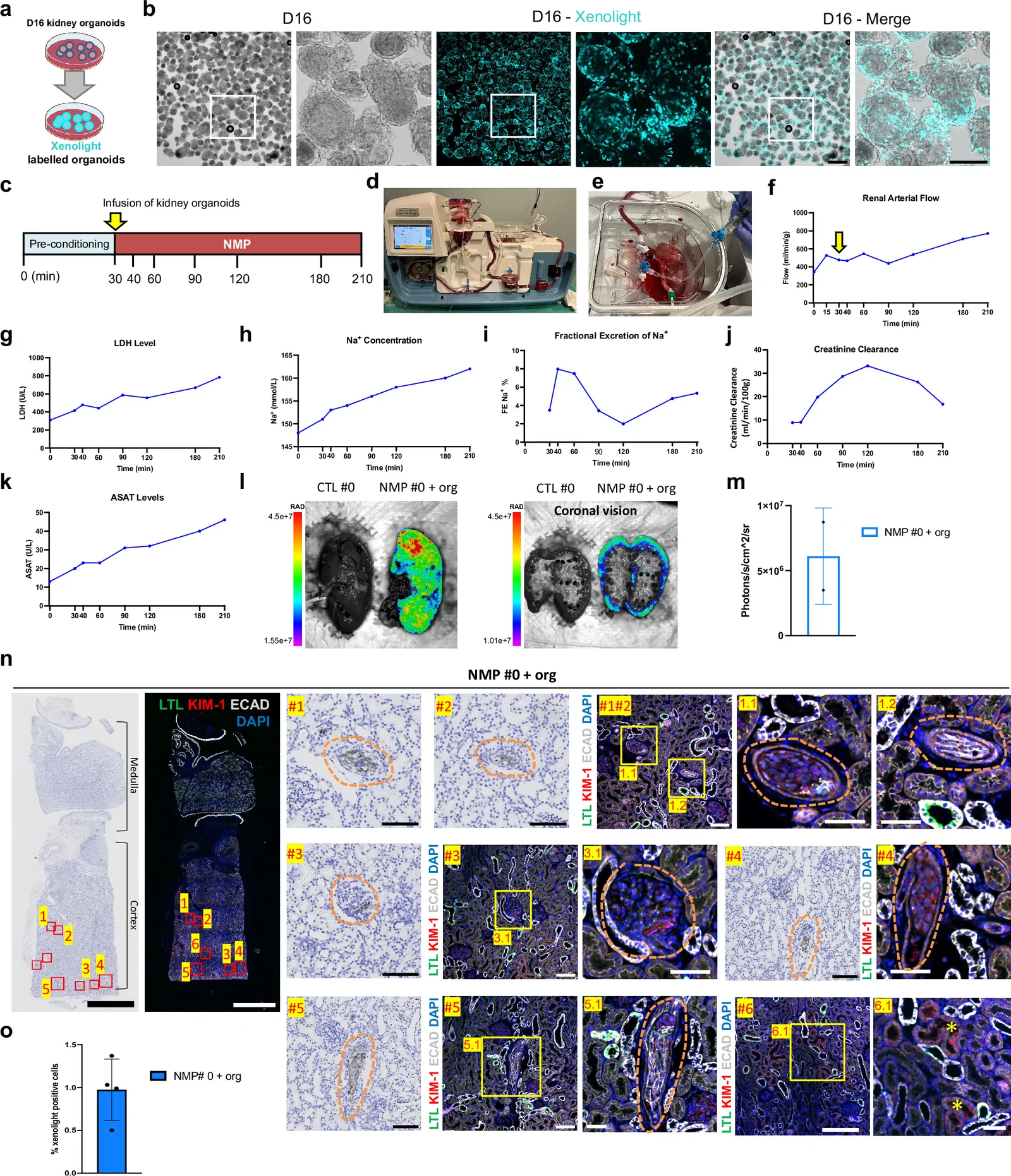
Engraftment of kidney organoids in porcine kidneys during *ex vivo* NMP. **a**, Kidney organoids were obtained by the microwell culture approach and day 16 intact organoids were labeled with a fluorescent dye, namely Xenolight, before infusion into porcine kidneys during *ex vivo* NMP. **b**, Representative bright field and confocal images of day 16 kidney organoids labelled with Xenolight. Scale bars, 500μm, 200μm (magnified views). Percentage of positive cells analyzed by flow cytometry in kidney organoids labelled with Xenolight was 62.5%, in front of other common cell membrane fluorescent dyes: Qtracker 655 (12.5% positive cells), Qtracker 800 (20.9% positive cells) and IVI Sense 680 (68.4% positive cells). Images are representative of three independent experiments. **c**, Experimental scheme for infusion of kidney organoids during *ex vivo* NMP in porcine kidneys. **d**, Photograph of the EBERS NMP setup used during porcine kidney perfusion. **e**, Photograph of a porcine kidney during NMP at the time of human kidney organoid infusion. **f**, Arterial flow (mL/min/100 g) of the kidney infused with organoids during NMP. The yellow arrow indicates the time of organoid infusion. **g**, Lactate dehydrogenase (LDH) levels (U/L) during NMP. **h**, Sodium concentration (mmol/L). **i**, Fractional excretion (FE) of sodium (FENa+) (%). **j**, Creatinine clearance (mL/min/100g). **k**, Aspartate aminotransferase (ASAT) levels (U/L). In **f-k**, *n=* 1 kidney infused with organoids during NMP (NMP #0 + org). **l**, IVIS fluorescent images of a kidney infused with Xenolight-labeled kidney organoids during *ex vivo* NMP (NMP#0 + org). Non-perfused contralateral kidney was used as control (CTL). Whole and coronal organ views are shown. **m**, Quantification of the average radiant efficiency (photons/s/cm^2^/sr) from the IVIS images in panel f (NMP #0 + org; *n=1*). Data are mean ± s.d. **n**, Detection of Alu repeat sequences of human DNA by *in situ* hybridization in formalin fixed, paraffin sections of porcine renal cortex from a kidney infused with kidney organoids during *ex vivo* NMP (NMP #0 + org) (*n= 1*). Scale bars, 2mm (whole slide image), 50μm (magnified views). n = 3 cortical punch biopsies from one kidney were analyzed with similar results. Immunofluorescence and confocal images in consecutive paraffin sections for the detection of ECAD (grays), LTL (green), KIM-1 (red) and DAPI (blue) are shown. Scale bars, 2mm (whole slide image), 100μm (magnified views #1to #5), 200μm (magnified view #6), 50μm (closer magnified views). n = 3 cortical punch biopsies from one kidney were analyzed with similar results. Dashed lines indicate areas containing cells of human origin. Yellow asterisks indicate KIM-1 positive tubuli. **o**, Quantification of the percentage of Xenolight positive cells by flow cytometry in renal cortical tissue from organoid-infused kidneys after NMP. Data are mean ± s.d. *n=* 4 biopsy samples from one kidney (NMP #0 + org) were analyzed. Source data

Before organoid delivery to the pig kidney, we labelled hPSC-kidney organoids with a near-infrared fluorescent dye (Xenolight) which showed a great efficiency (62.5% ± 11.4% of Xenolight-positive cells in stained organoids; *n* = 3 biological replicates) compared with other common cell membrane fluorescent dyes (Extended Data Fig. 7a,b), necessary to achieve high-sensitivity and reliable optical imaging data after NMP and in vivo transplantation. We then used a commercially available NMP system (Ebers ARK system) to perfuse porcine kidneys for up to 3 h, based on our previous results^23,25–27^ and the observations shown here. The infusion of Xenolight-labelled organoids was performed after 30 min of organ preconditioning (Extended Data Fig. 7c–e; Methods). Renal arterial flow increased after organoid infusion, remaining constant until the end of the experiment with no major differences in flow progression (Extended Data Fig. 7f). Metabolic, functional and vasomotor activity of organoid-infused kidneys during NMP was preserved (Extended Data Fig. 7g–k). Xenolight-labelled cells were detected in the cortex region of the kidney making use of the IVIS optical imaging system (PerkinElmer) (Extended Data Fig. 7l). Image analysis and quantification showed an average radiance of 6.10 × 10^6^ photons s^−1^ cm^−2^ sr^−1^ 3 h after organoid infusion during NMP (Extended Data Fig. 7m). Further analysis demonstrated the presence of Alu^+^ cells in both glomerular and blood vessel structures (Extended Data Fig. 7n). Consecutive paraffin tissue sections were stained for the tubule markers ECAD and LTL, and the marker of tubular injury kidney injury molecule-1 (KIM-1) as a surrogate of tubular damage during NMP^33^. Our analysis confirmed the presence of human cells in the renal cortex of pig kidneys after NMP, positive for ECAD and LTL. Our observations highlighted few tubular structures co-expressing KIM-1 (Extended Data Fig. 7n) in accordance with previous observations indicating the presence of tubular inflammation after NMP^33^.

**Fig. 3:**
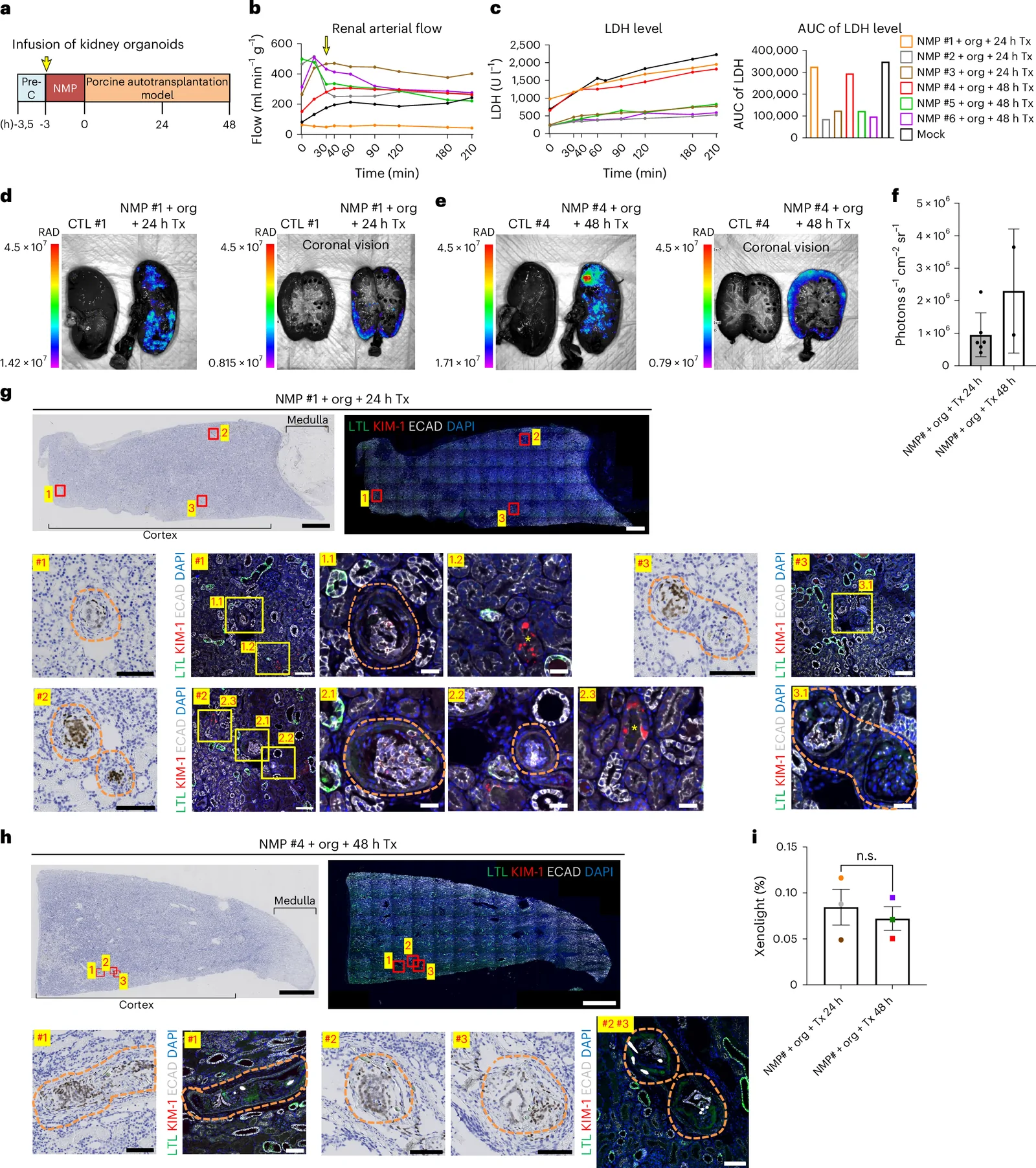
In vivo transplantation of porcine kidneys infused with human kidney organoids. **a**, Experimental scheme for infusion of kidney organoids in pig kidneys during NMP and further in vivo kidney transplantation into the same pig. In vivo transplanted organs and contralateral controls were collected 24 h (*n* = 3) and 48 h (*n* = 3) after transplantation. **b**, Arterial flow (ml min^−1^ per 100 g) of organoid-infused kidneys during NMP. The yellow arrow indicates the time of organoid infusion. **c**, LDH levels (U l^−1^) during NMP. In **b** and **c**, *n* = 6 organoid-infused kidneys, *n* = 1 kidney without organoids (Mock). **d**,**e**, IVIS fluorescent images of organoid-infused kidneys 24 h (NMP #1 + org + 24 h Tx) (*n* = 1) (**d**) and 48 h (NMP #4 + org + 48 h Tx) (*n* = 1) (**e**) after transplantation. org, organoids. Non-perfused contralateral kidneys are also shown (CTL). Whole and coronal organ views are shown. **f**, Quantification of the average radiant efficiency (photons s^−1^ cm^−^^2^ sr^−1^) from the IVIS images of organoid-infused kidneys 24 h and 48 h after transplantation. Data are mean ± s.d. *n* = 3 kidneys + 24 h Tx, *n* = 1 kidney + 48 h Tx. **g**, Left: detection of Alu repeat sequences of human DNA by in situ hybridization in formalin-fixed paraffin sections of porcine renal cortex from kidneys 24 h after transplantation. Scale bars, 2 mm and 50 μm (magnified views). Right: immunofluorescence and confocal images in consecutive paraffin sections for the detection of ECAD (greys), LTL (green), KIM-1 (red) and DAPI (blue) are shown. Scale bars, 2 mm (whole slide image), 100 μm (magnified views #1 to #3) and 25 μm (closer magnified views). *n* = 3 cortical punch biopsies were analysed from three independent experiments showing similar results. **h**, Left: detection of Alu repeat sequences of human DNA by in situ hybridization in formalin-fixed paraffin sections of porcine renal cortex from kidneys 48 h after transplantation. Scale bars, 2 mm and 50 μm (magnified views). Right: immunofluorescence and confocal images in consecutive paraffin sections for the detection of ECAD (greys), LTL (green), KIM-1 (red) and DAPI (blue) are shown. Scale bars, 2 mm (whole slide image) and 100 μm (magnified views #1 to #3). In **g** and **h**, dashed lines indicate areas containing cells of human origin. Asterisks indicate KIM-1 positive tubuli. *n* = 3 cortical punch biopsies were analysed from three independent experiments showing similar results. **i**, Quantification of the percentage of Xenolight-positive cells by flow cytometry in renal cortical tissue from organoid-infused kidneys 24 h and 48 h after in vivo transplantation. Data are mean ± s.d. *n* = 3 biological replicates per group. n.s., no statistical significance. Unpaired Student’s *t*-test (two-sided). See also Extended Data Figs. 5–10. AUC, area under the curve. Source data

**Extended Data Fig. 8:**
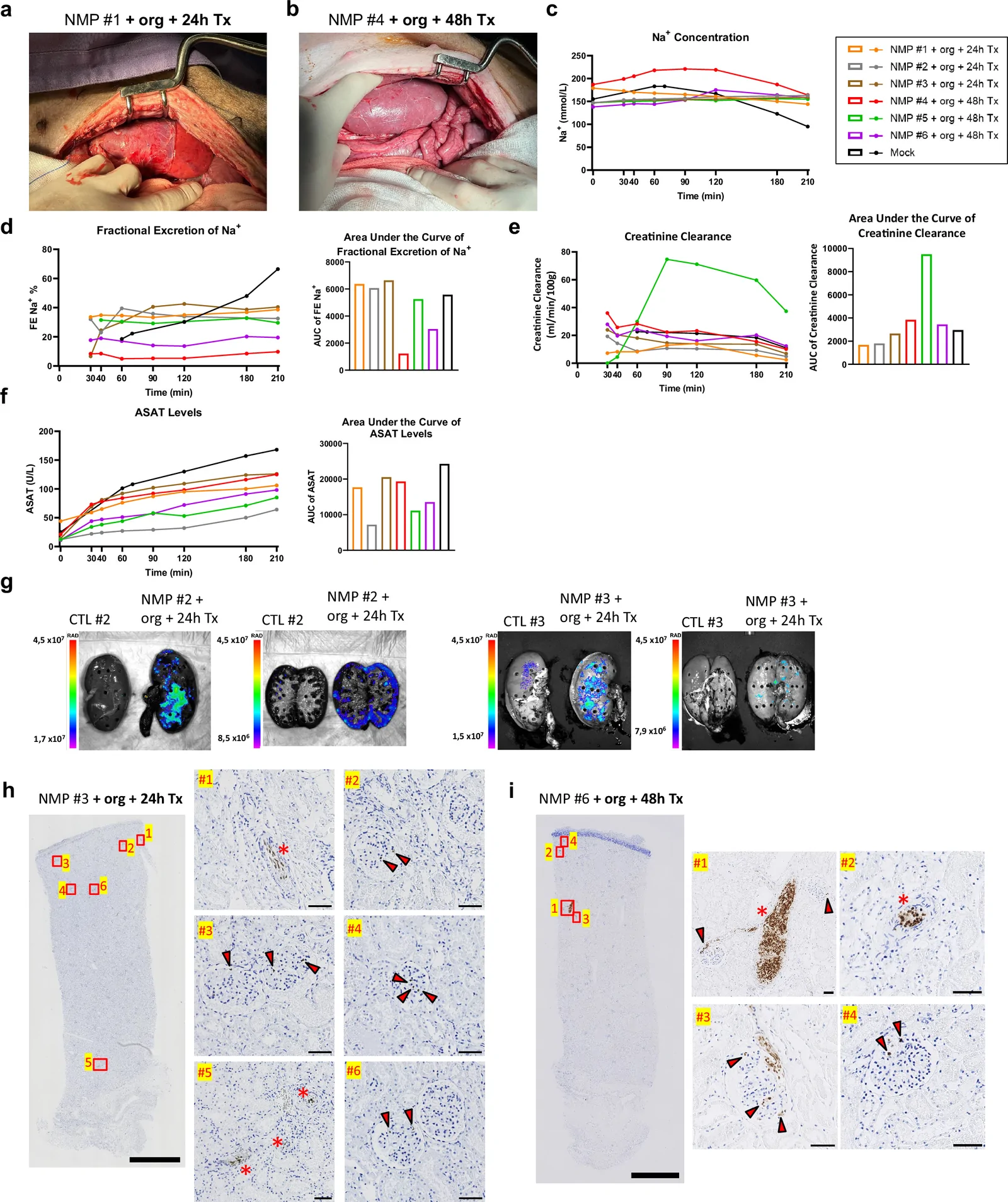
*In vivo* transplantation of porcine kidneys treated with organoids. **a**, Photograph of the kidney 24h post-transplantation (NMP #2 + org + 24h Tx). **b**, Photograph of the kidney 48h post-transplantation (NMP #2 + org + 48h Tx). **c**, Sodium concentration (mmol/L). **d**, FE of sodium (FENa+) (%) area-under-the curve (AUC). **e**, Creatinine clearance (mL/min/100g) and AUC. **f**, Aspartate aminotransferase (ASAT) levels (U/L) and AUC. In **c-f**, *n= 6* kidneys were infused with organoids (NMP #1, #2, #3 + org + 24h Tx and NMP #4, #5, #6 + org + 48h Tx) and *n= 1* kidney was not infused with organoids (Mock) during NMP. **g**, IVIS fluorescent images of kidneys infused with kidney organoids during *ex vivo* NMP following reimplantation in pigs for 24h. *n=* 2 (NMP #2, #3 + org + 24h Tx). Non-perfused contralateral kidneys are also shown (CTL). Whole and coronal organ views are shown. **h,i**, Detection of Alu repeat sequences of human DNA by *in situ* hybridization in formalin fixed paraffin sections of porcine renal cortex from kidneys 24h (h) and 48h (i) post-transplantation. n = 3 cortical punch biopsies were analysed from three independent experiments showing similar results. Arrowheads indicate Alu^+^ cells in glomerular structures. Asterisks indicate Alu^+^ cells in vascular structures. Scale bars, 2mm, 50μm (magnified views). Source data

**Extended Data Fig. 9:**
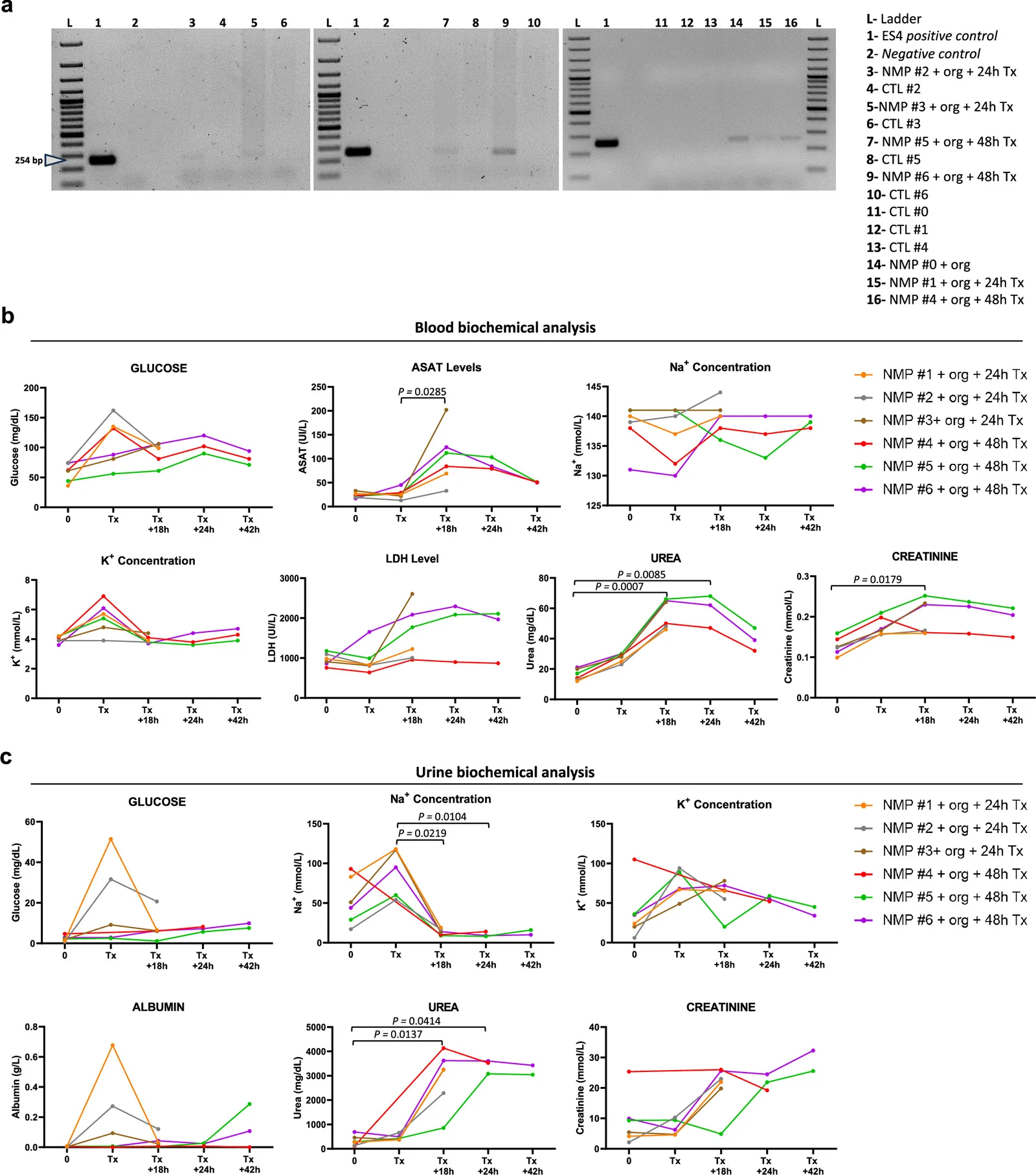
Human cell detection and biochemical analysis in reimplanted animals. **a**, Electrophoresed PCR products showing band sized 254bp for the detection of SRY (Y chromosome) in biopsy samples from organoid-infused kidneys before and after *in vivo* transplantation compared to the correspondent contralateral kidney biopsy samples. *n=* 3 NMP + org + 24h Tx (NMP #1, #2, #3 + org + 24h Tx) and *n=* 3 NMP + org + 48h Tx (NMP #4, #5, #6 + org + 48h Tx), and *n=* 1 NMP + org (NMP #0 + org) **b**, Blood and **c**, urine biochemical analysis performed in pigs before (t0, basal) and after reimplantation (Tx). *n=* 3 animals reimplanted with organoid-treated kidneys for 24h (NMP #1, #2, #3 + org + 24h Tx) and *n=* 3 animals reimplanted with organoid-treated kidneys for 48h (NMP #4, #5, #6 + org + 48h Tx). Kruskal-Wallis (two-sided) followed by Dunn’s multiple comparisons test. *p* values for statistically significant comparisons are shown. Source data

To assess the feasibility of our approach in a preclinical setting, organoid engraftment was evaluated by qualitative and quantitative techniques 24 or 48 h after ex vivo organoid infused kidneys were transplanted back into the same pig^31,32^(Fig. 3a; Methods). Importantly, no reperfusion syndrome was observed (Extended Data Fig. 8a,b), which has been previously described after transplantation of injured organs^34^. Renal arterial flow remained constant during NMP (Fig. 3b). Similarly, metabolic, functional and vasomotor activity of the organoid infused kidneys during NMP was preserved (Fig. 3c and Extended Data Fig. 8c–f). Xenolight-labelled cells were detected 24 h (Fig. 3d and Extended Data Fig. 8g) or 48 h (Fig. 3e) after transplantation in the cortex region of the kidneys. Image analysis and quantification showed average radiances of 0.95 × 10^6^ photons s^−1^ cm^−2^ sr^−1^ or 2.29 × 10^6^ photons s^−1^ cm^−2^ sr^−1^ 24 h and 48 h after transplantation, respectively, indicating that organoid-derived cells remained viable for at least 48 h after in vivo transplantation (Fig. 3f). To verify these results, we performed systematic microscopic analysis of paraffin sections prepared from kidney biopsies, identifying Alu^+^ cells in the renal cortex of in vivo transplanted kidneys (Fig. 3g,h and Extended Data Fig. 8h,i). Consecutive paraffin tissue sections were stained for the tubule markers ECAD and LTL, showing ECAD and LTL expression in cells of human origin 24 h (Fig. 3g) and 48 h (Fig. 3h) after transplantation, as well as KIM-1. The results confirm that hPSC-kidney organoids persist at both 24 h and 48 h after kidney reimplantation, albeit a certain degree of damage could be observed in both conditions (Fig. 3g and Extended Data Fig. 7n). In addition, FACS analysis revealed the detection of Xenolight-positive cells before (Extended Data Fig. 7o) and after (Fig. 3i) kidney reimplantation. Concomitantly, PCR analysis on genomic DNA detected the amplification of genetic material of human origin (Extended Data Fig. 9a). During postoperative follow-up, we performed biochemical analysis of blood and urine samples retrieved from transplanted pigs (Extended Data Fig. 9b,c and Supplementary Table 1). Blood glucose, sodium, potassium and LDH levels in animals reimplanted with organoid-infused kidneys remained mostly invariable during the whole study period with values close to basal levels (Extended Data Fig. 9b). Conversely, blood ASAT, urea and creatinine levels peaked at transplantation (Tx) + 18 h and subsequently approached basal levels at Tx + 42 h (Extended Data Fig. 9b and Supplementary Table 1). In urine, glucose, albumin, potassium and creatinine levels showed values comparable to basal levels (Extended Data Fig. 9c and Supplementary Table 1), whereas sodium and urea levels showed significant variations compared with baseline levels (Extended Data Fig. 9c). These variations might be attributed to factors related to the surgical procedure and the associated physiological response of the organism, as previously reported^34,35^. Overall these findings suggest that graft viability was mostly preserved during the study period evaluated here, overall showing the feasibility and viability of our approach.

### Human kidney organoids do not elicit major immune responses locally or systemically

**Fig. 4:**
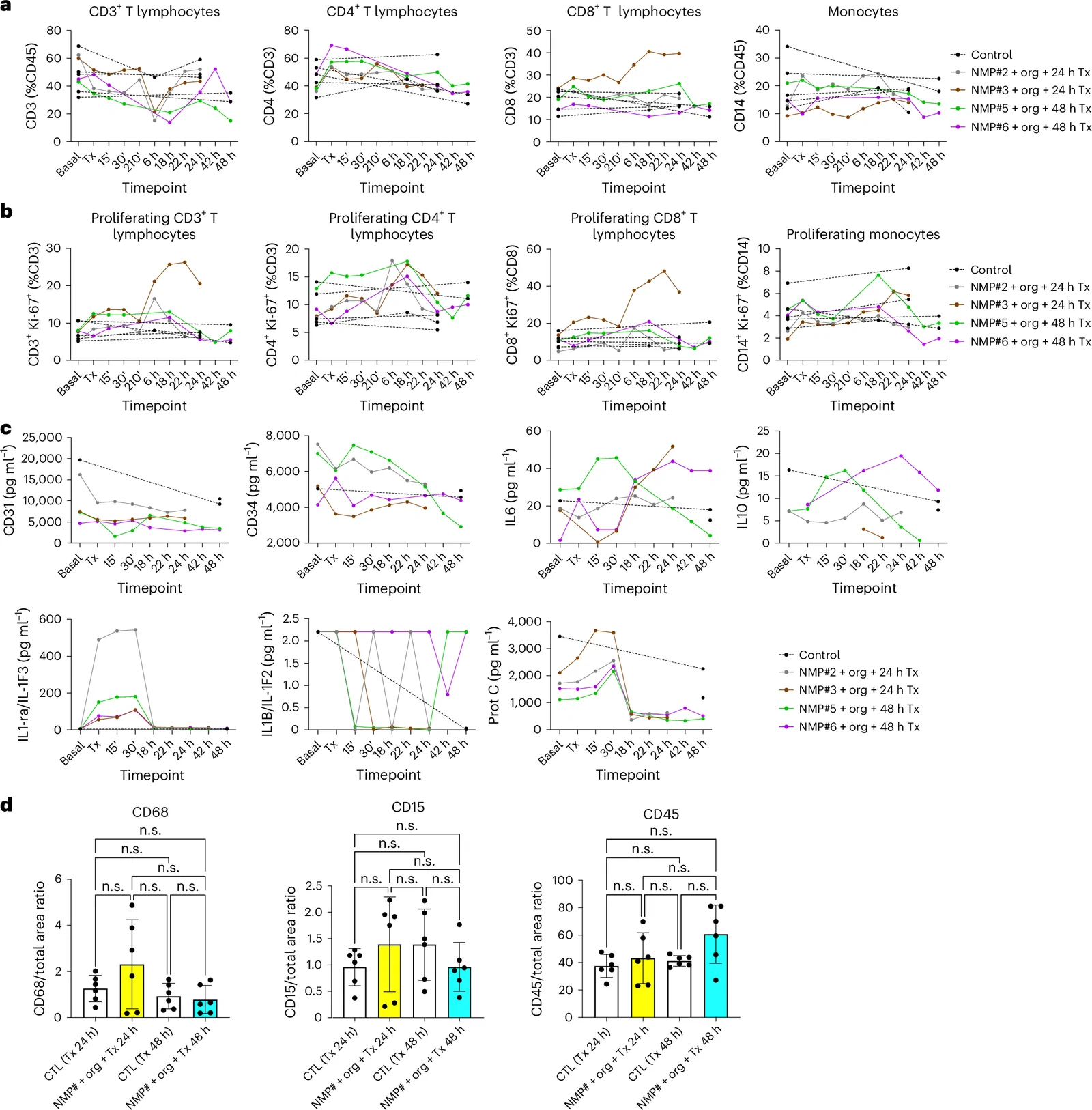
Analysis of potential systemic and local immune response after in vivo transplantation of porcine kidneys infused with human kidney organoids. PBMCs of pigs at different timepoints after in vivo transplantation were analysed by flow cytometry. **a**, Levels of total CD3^+^ lymphocytes, CD4, CD8 or monocytes as a percentage of total CD45^+^ cells were analysed. **b**, Proliferating levels of CD3^+^ lymphocytes, CD4, CD8 or monocytes by the expression of Ki-67. Each line represents a single pig. **c**, Serum levels of CD31, CD34, IL6, IL10, IL1-ra, IL1B and CRP measured by Luminex. In **a**–**c**, *n* = 5 control (non-transplanted animals), *n* = 4 transplanted animals. Mann–Whitney (two-sided) test was used to compare between the two experimental groups at 0, 24 and 48 h; no statistical differences were found. Paired Wilcoxon matched-pairs signed-rank (two-sided) test was used to analyse changes inside the same group at 0, 24 and 48 h; no statistical differences were found. *P* values less than 0.05 were considered statistically significant. **d**, Image quantification of CD68, CD15 and CD45 area relative to total tissue section area in renal cortical biopsies retrieved from kidneys 24 h and 48 h after transplantation. Data are mean ± s.d. *n* = 3 biological replicates per group with technical duplicates each. Kruskal–Wallis (two-sided) followed by Dunn’s multiple-comparisons test. See also Extended Data Fig. 10. Source data

**Extended Data Fig. 10:**
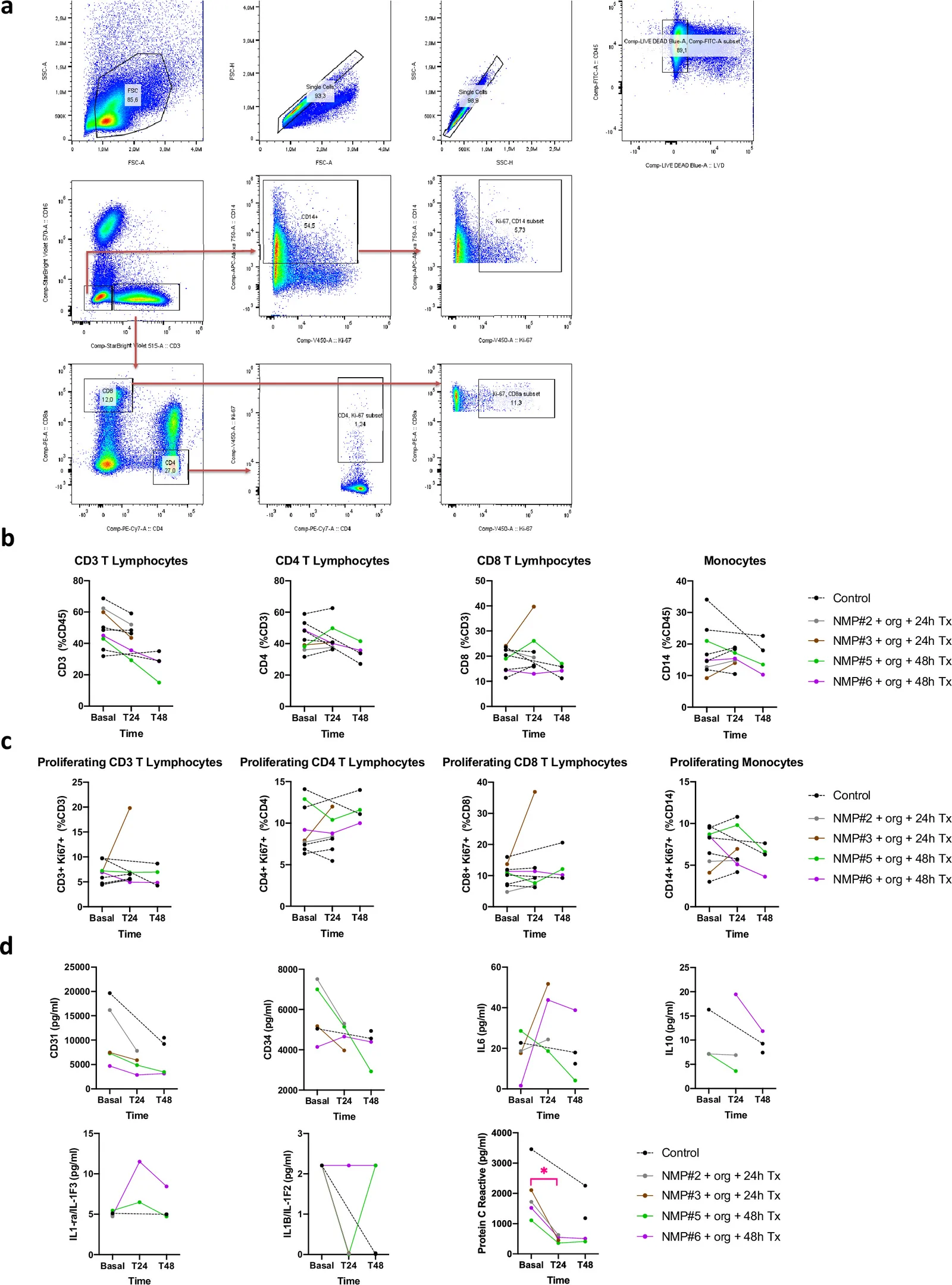
Systemic inmune response after *in vivo* transplantation of porcine kidneys infused with human kidney organoids. **a**, Flow cytometry gating strategy for analyzing peripheral blood mononuclear cells of pigs at different timepoints after *in vivo* transplantation (Tx) were analyzed by flow cytometry. **b**, Levels of total CD3 lymphocytes, CD4, CD8 or monocytes as percentage of total CD45 cells were analyzed at times 0, 24 and 48 hours. **c**, Proliferating levels of CD3 lymphocytes, CD4, CD8 or monocytes by the expression of Ki-67 at times 0, 24 and 48 hours. Each line represents a single pig. In **b,c**, *n=* 5 control (non-transplanted animals), *n=* 4 transplanted animals. Mann-Whitney (two-sided) test was used to compare between the 2 experimental groups at 0, 24 and 48 hours, no statistical differences were found. Paired Wilcoxon matched-pairs signed rank (two-sided) test was used to analyze changes inside the same group at 0, 24 and 48 hours, no statistical differences were found. P-values less than 0.05 were considered statistically significant. **d**, Serum levels of CD31, CD34, IL6, IL10, IL1-ra, IL1B and C reactive protein measured by Luminex. *n=* 1 control (non-transplanted animals), *n=* 4 transplanted animals. Paired Wilcoxon matched-pairs signed rank (two-sided) test was used to analyze changes inside the transplanted group at 0, 24 and 48 hours, no statistical differences were found. Unpaired Mann-Whitney test (two-sided) was also used to analyze changes inside the transplanted group at 0, 24 and 48 hours, *p= 0.0286 (0, 24h). *p*-values less than 0.05 were considered statistically significant. Source data

To evaluate the possible elicited xenogeneic pig-mediated immune response against human organoids, we characterized the host adaptive and innate immune system by flow cytometry and Luminex assays. First, we evaluated the cell percentages of myeloid cells and T cell lymphocytes in peripheral blood mononuclear cells (PBMCs) at different timepoints before and after transplantation (gating strategy is detailed in Extended Data Fig. 10a). PBMCs from non-transplanted animals (*n* = 5) were assessed as a control group. Figure 4a and Extended Data Fig. 10b show no significant differences in the percentages of total CD3^+^ T lymphocytes, CD4^+^ T helper and CD8^+^ T cytolytic cells between the transplanted and the control group at the indicated timepoints (*T* = 0 h, *T* = 24 h and *T* = 48 h). These longitudinal results suggest that human organoids do not elicit major adaptive immune responses when implanted into pig kidney autografts. To evaluate the proliferative state of T lymphocytes, the expression of Ki-67, a protein induced in dividing cells^36^, was also analysed. Figure 4b and Extended Data Fig. 10c show no significant differences in the percentage of CD3^+^, CD4^+^ and CD8^+^ T cell proliferation between groups or longitudinally within the same group. The response of the innate immune system was also monitored by evaluating the percentage of classical monocytes. Consistent with the T cell results, no significant differences in the percentage of monocytes or their proliferative status were observed between groups at the indicated timepoints (*T* = 0 h, *T* = 24 h and *T* = 48 h) were observed (Fig. 4a,b and Extended Data Fig. 10b,c). Overall, flow cytometric analysis showed no significant changes in the immunological response to human organoids implanted in xenogeneic pig kidney grafts. To further characterize the immune response, immunological parameters were also assessed by Luminex, including cytokines, growth factors and inflammatory proteins. Figure 4c and Extended Data Fig. 10d show no significant differences in the serum levels of CD31 and CD34, which have been previously used as markers of endothelial cell damage during kidney perfusion^37,38^. In line with these results, pro-inflammatory IL-6 and anti-inflammatory IL-10 show no significant differences between groups^39,40^. Similar results were observed when measuring plasma levels of pro-inflammatory IL-1B or antagonistic IL-1RA^41^. Serum levels of C-reactive protein (CRP), an independent risk factor for renal allograft loss that correlates with metabolic syndrome^42^, also showed no significant changes (Fig. 4c and Extended Data Fig. 10d). It has been previously demonstrated that xenogeneic immune responses occur within minutes or hours after transplantation, leading to hyperacute rejection and graft destruction within 24 h (ref. ^43^). We observed an early increase in IL-6 and IL-1RA in the plasma at reperfusion, which approached baseline levels at the experimental endpoint after 24 h (ref. ^44^). Here, we extended this endpoint to 48 h and confirmed that several immunological parameters are not significantly affected within the control nor transplanted groups. In light of our results, we conducted phenotypic analysis using a multimodal strategy that combined morphological evaluation and immunophenotyping (CD15, CD45 and CD68), along with image analysis and quantification, to further assess initial immune responses in pig kidney xenografts after transplantation. Phenotyping was conducted on paraffin sections of kidney samples retrieved 24 h or 48 h after transplantation (including control lateral kidneys) as well as non-transplanted kidneys after NMP in the presence of organoids. We confirmed the presence of innate immune cells (CD68^+^ and CD15^+^ cells) and leukocytes (CD45^+^ cells) in all the experimental conditions, but automated quantification demonstrated no significant differences among the experimental groups (Fig. 4d). Our study provides a detailed report on the localization and immunophenotypic composition of inflammatory cells after infusion of human kidney organoids during NMP and in vivo transplantation. Our observations highlight that CD68^+^ and CD15^+^ cells were predominantly located within the glomeruli and/or tubular compartments, whereas CD45^+^ cells were observed within the peritubular space (a link to the images is provided in Methods).

## Discussion

Organoids derived from hPS cells have emerged as one of the next-generation multicellular systems to model human development and disease. Their potential therapeutic applicability stems from their inherent properties, which capture the cellular composition, function and organization found in the native organ^45^. In relation to hPSC-kidney organoids, different laboratories have proved that these in vitro models exhibit disease-related phenotypes (using genome editing^46–48^ or using patient iPS cells as starting cell source^49,50^) also offering potential applications to model infectious disease^4,5,51–54^ and cell replacement therapies upon damage^55^. Besides these advances, studies using hPSC-kidney organoid implantation in vivo have mostly explored the beneficial impact of transplantation on kidney organoid graft maturation^2,18,19,21,22^, with no study so far aimed to further investigate the potential use of these model systems for potential transplant applications.

So far, strategies to produce kidney organoids from hPS cells rely on the specific induction of the MM lineage^56,57^ or the simultaneous induction of MM- and UB-like progenitors^58^, with only one study generating higher-order grade hPSC-kidney organoids through manual assembly of MM- and UB-derived hPS cells^59^. Similarly, bioengineering approaches that use microfluidics have started to provide plausible scenarios to mirror physiologically relevant conditions to promote vascularization^1^ and generate hPS cell-derived renal cell types^60^. Despite all these advances, variability of growth and patterning of organoids still results in highly heterogeneous end-point collections, which represents one of the major limitations of the organoid technology^61^. We have previously shown that increasing the time that hPS-cell-PIM-committed cells are exposed to 3D culture conditions results in the generation of kidney organoids with higher features of differentiation at transcriptomic, structural and morphological levels^45^. Here, we further explored the identification of culture conditions empowering kidney organoid differentiation into segmented nephron structures including multiple renal cell types. We reasoned that forcing different numbers of cell-to-cell interactions from the same pool of hPS-cell-PIM-committed cells (500, 8,000, 100,000 and 250,000) would help to identify the initial number of progenitor cells leading to kidney organoids with nephron-like structures and transcriptomic signatures resulting from cell sorting and self-organization processes. In support of this hypothesis, we have demonstrated that the aggregation of 500 enables the generation of kidney organoids that exhibit higher traits of differentiation compared with other assayed conditions in different hPS cell lines, including expression of nephron and endothelial markers at the scRNA-seq level, a substantial reduction in stromal-like cells, enhanced proximal tubule differentiation and production, and metabolic rewiring towards an OXPHOS state. It has been shown that undifferentiated stromal populations negatively interfere vascularization and differentiation upon transplantation^6,10^, thus opening other applications of the approach described here for future developments to boost vascularization of hPSC-kidney organoids. Further use of our differentiation procedure may also include coupling the culture conditions described here with interventions on specific metabolic routes fuelling energy metabolism and thus boost the generation of specific renal cells within kidney organoids as shown by us^3^ and more recently by others when promoting tubular differentiation^10^. We next investigated the scalability of our kidney organoid procedure in controlled conditions making use of microwell structures. First, we established a one-step selection-free knocking strategy through CRISPR–Cas9 engineering to generate WT1-GFP reporter hPS cells allowing real-time monitoring of WT1 gene expression as well as enrichment of specific glomerular-like populations during kidney organoid differentiation. Overall, this strategy allows to generate ~30,000 hPSC-kidney organoids per each 24-well plate with nearly 100% of the resulting organoids exhibiting segmented nephron-like structures positive for the expression of nephron and endothelial markers. Interestingly, the procedures described here present a series of advantages, such as short culture time, high fidelity and independence of extracellular matrix components for organoid generation and maintenance. This underpins the potential translation of our findings to other applications such as drug screening and disease modelling.

Ex vivo organ machine perfusion has the potential to serve as a technology for graft interventions including pharmacologic organ preconditioning, stem cell and organoid therapy and gene modulation^28,62,63^. However, the application of complex cellular systems, such as hPS cell-derived organoids, for graft pretreatment has been limited by the lack of methodologies to ensure the production of sufficient quantities of hPS cell-derived renal cells (including organoids and/or differentiated cell types) that have adequate molecular and cellular fidelity. To apply organoids obtained through our differentiation approach in a realistic graft pretreatment setting, we subjected viable slaughterhouse-derived porcine kidneys to several hours of normothermic ex vivo perfusion and intraarterial administration of intact hPSC-kidney organoids or isolated cells from hPSC-kidney organoids. Cell doses were tested in two different concentrations based on preclinical studies in pigs using mesenchymal stem cells during NMP from previous studies performed by our group^64^ and others^65^. Our experiments demonstrate that the addition of both hPSC-kidney organoids and isolated cells during NMP does not elicit alterations to perfusion dynamics. The approach undertaken here allowed cells of human origin to remain structurally intact and detectable as identified by in situ hybridization, in vivo optical imaging and FACS analysis. Seminal work demonstrated that cells dissociated from gallbladder organoids can be used for autologous cell-based therapy to repair human intrahepatic bile ducts during ex vivo NMP^30^. Whereas that study utilized fluoroscopic guidance to administer enzymatically dissociated cells from gallbladder organoids into the intrahepatic ducts of human donor livers, our approach demonstrates that intraarterial administration of kidney organoids or their derivatives similarly maximizes cell density, as indicated by confocal microscopy, image reconstruction analysis and in vivo optical imaging. In the context of kidney, the administration of neonatal kidney progenitor cells to human donor kidneys during NMP reactivates the expression of the stem cell transcription factor SIX2 in proximal tubular epithelial cells of the donor kidneys upon perfusion^66^, suggesting a possible endogenous regenerative mechanism while raising questions on potential side effect consequences of SIX2 activation in donor kidneys^66^.

So far, there is no clear consensus on the best NMP strategy for the kidney^67^. One initial clinical trial demonstrated that a short (1-h) period of NMP following hypothermic preservation yielded positive results in terms of perfusion characteristics and early transplantation outcomes^68^. More recently, the same group conducted a randomized controlled trial comparing outcomes of DCD (donation after circulatory death) kidney transplants preserved using conventional static cold storage alone versus static cold storage followed by 1-h NMP^68^. Two other groups, one in Europe and the other in Canada, have started clinical programmes of NMP using longer periods of NMP (2–6 h), but results are still pending^67^. In general, there is a limited amount of evidence for NMP in clinical kidney transplantation with few case series^67^. Here, we aimed to validate perfusion characteristics and biomarkers detection during hPSC-kidney organoid infusion in a pig model. Our analysis incorporates morphological and quantitative outputs to capture hPSC-kidney organoid engraftment during NMP and in vivo, as well as evaluation of potential immune responses, overall showing the feasibility and viability of the procedure.

In summary, we present a systematic approach to determine specific culture conditions and timings to generate kidney organoids from hPS cells in a cost-effective and robust manner. Our approach empowers hPS cell differentiation and scaling up to generate kidney organoids that contain nephron-like structures with different renal cell types, as demonstrated by single-cell transcriptomic analysis, confocal microscopy and metabolic assays. Collectively, transplant research still lacks in vitro models that closely mimic organ features at a functional, structural and transcriptomic level. Such advances depend on our capability to engineer robust and affordable (costly and timely) organotypic technologies linking cellular processes to organ-level function. The combination of organoid technology and ex vivo organ machine perfusion offers opportunities to perform cellular interventions in fully controlled conditions before organs are transplanted in the context of living kidney donation. Here, we provide the proof of principle that machine perfusion represents an accessible and safe method to administer nephron-stage kidney organoid-based therapies to donor kidneys in the future. Therefore, the tools developed here open the door to further studies aimed at understanding the potential functional benefit of hPSC-kidney organoids and favour the development of therapeutic approaches for renal cell regeneration in the clinic.

### Limitations of the study

Notwithstanding the strengths of our study proving the feasibility and viability of our approach, we are aware that important limitations exist. Our porcine model lacks important hallmarks typically observed clinically as well as a longer follow-up period upon transplantation. While the anatomical and physiological similarities between pigs and humans support the potential translational relevance of our findings, the biological interpretation of these results requires further investigation in future studies.

Because we focused the design of our study on monitoring early time periods (24 h and 48 h) during human organoid infusion into porcine kidneys ex vivo and subsequent reimplantation in vivo, we cannot make any predictions with respect to the long-term organoid xenograft viability, engraftment and potential long-term immunogenic or side effects. Further studies will evaluate these aspects as well as the extent and impact of acute damage at longer post-transplantation time periods.

This study has evaluated a sample size of *n* = 7 perfused kidneys using a commercially available NMP system, of which *n* = 1 was perfused but not reimplanted, *n* = 3 was perfused and transplanted in vivo for 24 h and *n* = 3 was perfused and transplanted in vivo for 48 h. All perfused kidneys performed well during NMP. Future investigations will increase sample size to reduce potential biological variability.

Human kidney organoids were infused within porcine kidneys during ex vivo NMP and subsequently reimplanted in animals without performing nephrectomy of the contralateral kidney. As such, any functional analysis in the in vivo model is limited because the native contralateral kidney remains intact and functioning. Future studies involving contralateral kidney nephrectomy or utilizing kidney injury models will enable investigation of the impact of hPSC-kidney organoid treatment on renal function following in vivo transplantation.

In this regard, extensive dose–response studies coupled to kidney functional readouts are needed to uncover the potential therapeutic mechanisms (direct or paracrine) of hPSC-kidney organoid-based treatments.

## Methods

### Cells

hPS cell lines were obtained after the approval of the Ethics Committee from the Clinical Translational Program for Regenerative Medicine in Catalonia (P-CMR [C]) and the Comisión de Seguimiento y Control de la Donación de Células y Tejidos Humanos del Instituto de Salud Carlos III (project numbers 0036/3897/2015, 0336/1123/2021, 0336/2723/2021, 336/165/2022 and 9067/487286/2022). hES cell ES[4] line and CBiPS1sv-4F-40 iPS cell line were obtained from the National Bank of Stem Cells (ISCIII, Madrid). Both hPS cell lines were maintained and grown in Essential 8 medium (A1517001, Thermo Fisher Scientific) in cell culture plates coated with 5 µg ml^−1^ vitronectin (A14700, Thermo Fisher Scientific) with 5% CO_2_ at 37 °C. Cells were passaged every 4–6 days by disaggregating hPS cell colonies into small cell clusters using 0.5 mM EDTA (15575-038, Thermo Fisher Scientific).

### Generation of genome-edited hPS cell lines

The *WT1* reporter cell line was generated using a hES cell line in which a doxycycline-inducible Cas9 cassette was introduced at the AAVS1 safe harbour locus as we described before elsewhere^16,17^. A first screening using in-house retrotranscribed single guide RNAs (sgRNAs) was performed by lipid-based transfection (Lipofectamine RNAiMAX, #13778075) into doxycycline-treated undifferentiated hPS cells and subsequent editing quantification by MiSeq analysis on genomic DNA from transfected cells. MiSeq analysis was performed on a locus-specific pooled PCR library that was generated through two rounds of PCR introducing Illumina adapters and specific indexes. In this way, pooled cells edited with different sgRNAs were differentially labelled (Supplementary Table 2). Indel frequency in edited pools was obtained by analysing trimmed FASTQ reads using CRISPResso2 software (v 2.2.7)^69^. Results indicated that WT1 KI-R Cr4 was the sgRNA associated with the highest frequency of indel mutations (Fig. 2b). The sgRNA WT1 KI Cr4 (Supplementary Table 2) was ordered and purchased as 2 nmol Alt-R CRISPR–Cas9 crRNAs (CRISPR RNAs) (Integrated DNA Technologies) and annealed with Alt-R CRISPR–Cas9 tracrRNA (trans-activating CRISPR RNA) (Integrated DNA Technologies) following the manufacturer’s guidelines. In parallel, a donor plasmid containing a sequence of the GFP and a T2A sequence flanked by homology arms corresponding to the targeted sequence at the end of the *WT1* was designed (Extended Data Fig. 4c). The strategy for generation of the WT1 reporter cell line included the co-nucleofection of the sgRNA and the donor plasmid together with a pRRpuro plasmid (Extended Data Fig. 4a). The pRRpuro plasmid contained a puromycin resistance sequence interrupted by the protospacer sequence of the WT1 KI-R Cr4 together with the protospacer adjacent motif. Plasmids and sgRNAs were introduced in by nucleofection using the Amaxa Nucleofector (Lonza, VCA-1003). The mix contained 164 µl Nucleofector solution and 36 µl supplement and 1× DNA mix consisting of 3 µg donor vector, 2 µg pRR-Puro vector and 1 µg of Alt-R crRNA/tracrRNA duplex. After the co-nucleofection of the sgRNA and both plasmids, cells were plated as single cells and grown for 2 days in the absence of puromycin. Subsequent antibiotic treatment for 3 days enabled the selection of individual colonies, which were then picked and plated in duplicate 96-well plates. One replica plate was kept as a frozen stock while DNA was extracted for genotyping. The genotyping strategy was based on PCR screening using primers flanking the 5′ homology arm within exon 10 of WT1, and primers flanking the 3′ homology arm within the 3′ untranslated region (Extended Data Fig. 4d and Supplementary Table 3). This strategy identified that clone F9 contained the T2A-GFP sequence correctly integrated at the end of *WT1* sequence. This clone was selected as a candidate WT1-T2A-GFP reporter cell line (WT1-GFP reporter cell line) and was used for the studies described here.

### Kidney organoid differentiation

Before differentiation (day −5), undifferentiated hPS cell colonies were dissociated into cell clumps using 0.5 mM EDTA (15575-038 Thermo Fisher Scientific) at 37 °C for 4 min. To obtain single-cell suspensions, samples were then incubated in Accumax (07921, Stem Cell Technologies) at 37 °C for 3 additional minutes. Cells were counted using the Countess Automated Cell Counter (Invitrogen) and seeded at 1–2 × 10^5^ viable cells per well on 24-well plates coated with 5 µg ml^−1^ vitronectin in the presence of Essential 8 medium (A1517001, Life Technologies) at 37 °C overnight. The next day (day −4), differentiation was initiated by treating monolayer cultures with 8 μM CHIR99021 (CHIR; SML1046, Merck) in Advanced RPMI 1640 basal medium (AdvRPMI medium; 12633020, Thermo Fisher Scientific) supplemented with 1% penicillin–streptomycin and 1% GlutaMAX (35050061, Thermo Fisher Scientific) for 3 days with daily medium changes. On day −1, monolayer cultures were treated with 200 ng ml^−1^ FGF9 (100-23, Peprotech), 1 μg ml^−1^ heparin (H3149, Merck) and 10 ng ml^−1^ activin A (Act A) (338-AC-050, Vitro) in AdvRPMI medium supplemented with 1% penicillin–streptomycin and 1% GlutaMAX for 24 h. On day 0, cell monolayers were treated with 5 μM CHIR, 200 ng ml^−1^ FGF9 and 1 μg ml^−1^ heparin in AdvRPMI medium supplemented with 1% penicillin–streptomycin and 1% GlutaMAX for 1 h. After this 1-h treatment, cell monolayers were dissociated using TrypLE Express Enzyme (1260402, Thermo Fisher Scientific) for 1 min, collected and counted. Next, 250,000 cells per well, 100,000 cells per well, 8,000 cells per well or 500 cells per well were dispensed on a V-shape 96-well plate (249935, Thermo Fisher Scientific) and centrifuged at 300*g* for 5 min. Cell spheroids were cultured in AdvRPMI medium supplemented with 1% penicillin–streptomycin and 1% GlutaMAX, 200 ng ml^−1^ FGF9 and 1 μg ml^−1^ heparin for 7 days with medium changes every other day. From day 7, organoids were maintained in AdvRPMI medium supplemented with 1% penicillin–streptomycin and 1% GlutaMAX. From that stage, the medium was changed every other day. For kidney organoid generation using the microwell culture system, IM-committed cell monolayers (day 0) were first treated with 5 μM CHIR, 200 ng ml^−1^ of FGF9 and 1 μg ml^−1^ of heparin in AdvRPMI medium supplemented with 1% penicillin–streptomycin and 1% GlutaMAX for 1 h, and subsequently disaggregated using TrypLE Express Enzyme (1260402, Thermo Fisher Scientific) for 1 min, collected and counted. A 24-well AggreWell 400 Microwell plate (34411, Stem Cell Technologies) was pretreated with anti-Adherence Rinsing Solution (07010, Stem Cell Technologies) by adding 0.5 ml of the solution in each well for 5 min and centrifuged at 300*g* for 5 min to remove bubbles. The resultant single-cell suspension was then plated at 6 × 10^5^ cells per well of the 24-well AggreWell 400 Microwell plate in 2 ml of AdvRPMI medium supplemented with 200 ng ml^−1^ of FGF9, 1 μg ml^−1^ of heparin, 1% penicillin–streptomycin and 1% GlutaMAX. The plate was then centrifuged at 300*g* for 5 min and incubated with 5% CO_2_ at 37 °C. After overnight culture, uniform IM-committed cell spheroids formed in each microwell. Cell spheroids were cultured in AdvRPMI medium supplemented with 1% penicillin–streptomycin and 1% GlutaMAX, 200 ng ml^−1^ FGF9 and 1 μg ml^−1^ heparin for 7 days with medium changes every other day. On day 7, microorganoids were transferred to low-adherent p10 plates (833902500, Sarstedt) pretreated with anti-Adherence Rinsing Solution and maintained in AdvRPMI medium supplemented with 1% penicillin–streptomycin and 1% GlutaMAX until day 16 with medium changes every other day.

### scRNA-seq

scRNA-seq was performed as described in our previous study^4^. Specifically, kidney organoids were collected and washed twice with phosphate-buffered saline (PBS), and further dissociated into a single-cell suspension by treating them with Accumax (07921, Stem Cell Technologies) for 15 min at 37 °C followed by trypsin–EDTA 0.25% (wt/vol) trypsin (25300-054, Life Technologies) for an additional 15 min at 37 °C. The reaction was deactivated by adding 10% foetal bovine serum (FBS). The cell suspension was then passed through a 40-μm cell strainer. After centrifugation at 300*g* for 5 min, cell numbers and viability were analysed using Countess Automated Cell Counter (Invitrogen). Then, cell suspensions were loaded onto a well of a 10x Chromium Single Cell instrument (10x Genomics). Barcoding and cDNA synthesis were performed according to the manufacturer’s instructions. Qualitative analysis was performed using the Agilent Bioanalyzer High Sensitivity assay. The cDNA libraries were constructed using the 10x Chromium Single Cell 3′ Library Kit v3 according to the manufacturer’s original protocol. Libraries were sequenced on Illumina NextSeq 500 using the following read length: 28-bp Read1 for cell barcode and unique molecular identifier (UMI), 8-bp I7 index for sample index and 55-bp Read2. Libraries were preprocessed using Cell Ranger (3.0.1) from 10x Genomics (http://10xgenomics.com). Reads from each sample were aligned to the reference human genome (GRch38) downloaded from the 10x Genomics website (version 3.0.0, corresponding to Ensembl v93 annotation). The median number of UMIs per cell was between 7,939 and 13,570, with a median of 3,014 and 4,129 genes detected per condition.

### scRNA-seq data analysis

The computational analysis of the resulting UMI count matrices was performed using the R package Seurat (v 4.0.5)^70^. Cells were subjected to a quality control step, excluding cells with <1,000 or >7,000–7,800 detected genes, >35,000–45,000 UMIs or >10% of UMIs assigned to mitochondrial genes. Thresholds for each sample were obtained from visual inspection of the UMI and detected genes distribution. In addition, cells labelled as doublets by scDblFinder (v1.9.4) were discarded, and genes expressed in fewer than three cells for a sample independently, or in fewer than five cells when the samples were merged, were removed from the analyses. Each dataset was separately subjected to normalization, identification of highly variable features and scaling using the SCTransform function with the ‘glmGamPoi’ method parameter, regressing out for the number of detected genes, the percentage of mitochondrial UMIs and cell cycle. All samples were integrated in Seurat using canonical correlation analysis to remove batch effects and enable downstream comparisons between the different organoid sizes.

Principal component analysis was performed, and the top 20 components were kept for further analysis in the integrated dataset. Unsupervised clustering was performed by setting the resolution parameter to 0.5 in the FindClusters function. Dimensionality reduction for data visualization was done using the RunUMAP function.

Cell markers in each cluster were identified using the FindConservedMarkers and FindAllMarkers functions in the non-integrated counts by using the Wilcoxon rank-sum test. Genes with a *P* value <0.05 (adjusted by Bonferroni’s correction) were retained. Clusters were labelled by comparing the expression of the identified markers with databases available at the Kidney Interactive Transcriptomics (KIT) webpage (http://humphreyslab.com/SingleCell/) and with markers from previous publications^71,72^. Differential expression analysis to identify changes across conditions were also performed using the Wilcoxon test, keeping as upregulated genes those present in a fraction of 0.1 of either population, with a log fold change greater than 0.1 and with an adjusted *P* value <0.05. To perform overrepresentation analysis between conditions, we used the R package enrichR (v3.0)^73^.

### Histology and immunocytochemistry of kidney organoids

Specimens were fixed at the indicated timepoints with 4% paraformaldehyde (153799, Aname) overnight at 4 °C. Samples were then washed twice with PBS, embedded in paraffin and sectioned into 5–10-µm samples. Sections were stained with haematoxylin and eosin. Images were captured using the AF7000 Leica microscope. For immunofluorescence, samples were fixed with 4% paraformaldehyde (153799, Aname) for 20 min at room temperature, washed three times with PBS and blocked using Tris-buffered saline containing 6% donkey serum (S30, Millipore) and 1% Triton X-100 (T8787, Sigma) for 1 h at room temperature. Samples were treated overnight at 4 °C with the primary antibodies indicated in Supplementary Table 4 diluted in antibody dilution buffer (Tris-buffered saline solution with 6% donkey serum and 0.5% Triton X-100). Samples were then washed three times with antibody dilution buffer and further incubated for 4 h at room temperature with fluorescent conjugated secondary antibodies indicated in Supplementary Table 5.

### RT–qPCR

RNA was isolated from cells and kidney organoids using TRIzol (Invitrogen). Two micrograms of RNA was reverse transcribed using the cDNA archival kit (Life Technology), and qPCR with reverse transcription (RT–qPCR) was run in the QuantStudio5 (Life Technologies) machine using SYBRGreen Master Mix (Applied Biosystem) and gene-specific primers. The data were normalized and analysed using the ∆∆Ct method. The primer sequences used are presented in Supplementary Table 6. Design and Analysis QuantStudio Real time PCR software (v 1.5.2) was used for qPCR data collection.

### OCR

The measurement of oxygen consumption rate (OCR) in kidney organoids was performed using an XFe24 extracellular flux analyser (Seahorse Bioscience) as previously described^2,3^. Specifically, day 16 kidney organoids were transferred into a Seahorse cell culture microplate in warm Seahorse XF Assay Medium supplemented with 5 mM glucose, 0.5 mM pyruvate and 2 mM glutamine (Seahorse Bioscience). After 1 h of incubation at 37 °C, plates were loaded into an XF24 respirometry machine (Seahorse Bioscience). Uncoupled and maximum OCR were assayed with oligomycin (10 μM) and FCCP (2 μM). To inhibit complex I- and III-dependent respiration, rotenone (5 μM) and antimycin A (15 μM) were used, respectively. OCR represents the oxygen tension and acidification of the medium as a function of time (pmol min^−1^). OCR was normalized to protein quantity in each well. Seahorse wave desktop software (v 2.6) was used for data collection and analysis.

### Labelling of kidney organoids and single-cell suspensions from kidney organoids

Before infusion of organoids in porcine kidneys during NMP, kidney organoids and single-cell suspensions derived from kidney organoids were labelled with Qtracker 655 (Cell Labelling Kits, Thermo Fisher Scientific) following the protocol provided by the manufacturer. A total number of 10,000 intact organoids were labelled with Qtracker 655 for 1 h at 37 °C with periodic shaking. For labelling kidney organoid-derived single cell suspensions, samples were prepared by washing kidney organoids twice with PBS and dissociating these organoids using Accumax (07921, Stem Cell Technologies) for 15 min at 37 °C and trypsin–EDTA 0.25% (wt/vol) trypsin (25300-054, Life Technologies) for additional 15 min at 37 °C. The reaction was deactivated by adding 10% FBS. Cell suspensions were then passed through a 40-μm cell strainer, and after centrifugation at 300*g* for 5 min, cell numbers and viability were analysed using Countess Automated Cell Counter (Invitrogen). A total of 14 million cells were labelled with Qtracker 655 for 1 h at 37 °C with periodic shaking, following the manufacturer’s instructions.

Kidney organoids were labelled making use of the commercially available lipophilic tracer 1,1-dioctadecyl-3,3,3,3-tetramethylindotricarbocyanine iodide (IVISense DiR 750 Fluorescent Cell Labeling Dye –XenoLight, 125964, PerkinElmer). Kidney organoids (pool of 7,200 organoids per tube) were labelled with 2.16 ml of Xenolight-labelling solution (50 μg ml^−1^ final concentration) for 30 min, washed one time with PBS and resuspended in medium. Similar procedures were done for Qtracker 800 (Cell Labelling Kits, Thermo Fisher Scientific) and IVISense 680 Fluorescent Cell Labeling Dye (NEV12001, PerkinElmer).

### Study design

For this preclinical study, we utilized porcine kidneys based on their high resemblance to human kidneys in size, anatomical and physiological characteristics. For experiments shown in Extended Data Figs. 5 and 6, viable porcine kidneys (sow; type: Topigs 20) and autologous, heparinized whole blood were obtained from a local slaughterhouse. Kidneys underwent 30 min of warm ischaemia, and then organs were flushed with cold UW-CS (Belzer UW cold storage solution, Bridge to Life) preservation solution. Storage and transport were done on melting ice (0–4 °C). Autologous red blood cell concentrates were obtained after leukocyte depletion by filtering, centrifuging and washing porcine blood. Kidneys were randomly assigned to four experimental groups. All the groups were subjected to a phase of organ conditioning (1 h). In the first group, kidneys underwent 3 h of NMP without organoid infusion (*n* = 2). In the second group, kidneys underwent 3 h of NMP with organoid infusion at low dose (36 organoids per gram of renal tissue; *n* = 3). In the third group, kidneys underwent 3 h of NMP with organoid infusion at high dose (154 organoids per gram of renal tissue; *n* = 1). In the fourth group, kidneys underwent 3 h of NMP with infusion of cells obtained by enzymatic dissociation of organoids (*n* = 2). In all four groups, NMP was performed using a custom-made perfusion set-up described elsewhere^25^. For experiments shown in Figs. 3 and 4 and Extended Data Figs. 7–10, kidneys from laboratory female pigs (Hybrid Landrace/Large White cross breed; 12–14 weeks of age; *n* = 7 animals) were randomly assigned to three experimental groups. All the groups were subjected to a phase of organ conditioning (30 min). In the non-transplanted group, the kidney underwent 3 h of NMP with organoid infusion (*n* = 1). In the second and third experimental groups, kidneys underwent 3 h of NMP with organoid infusion followed by autotransplantation and graft retrieval at 24 h (*n* = 3) or 48 h (*n* = 3) after transplantation, respectively. In all three groups, NMP was performed using the Ark kidney set-up from EBERS Medical Technology SL.

### Ethics and animals

Female laboratory pigs (Hybrid Landrace/Large White crossbreed; 12–14 weeks of age) weighing approximately 50 kg were used for autotransplantation. All animal care and procedures followed guidelines by the European Union (directive 2010/63/EU) and local regulations. The study was approved by the Ethical Committee of Animal Experimentation of the University Hospital Complex of A Coruña (reference number 15002/2023/04). All personnel involved had Federation of European Laboratory Animal Science Associations licences.

### Surgical procedure

The surgical procedure followed the method outlined by Kaths et al., except that contralateral nephrectomy was not performed^31^. Before the procedure, premedication for sedation was administered using ketamine (15 mg kg^−1^), midazolam (0.4 mg kg^−1^) and medetomidine (4 mg kg^−1^). The marginal vein of the ear was then cannulated, and anaesthesia induction was achieved with propofol. After orotracheal intubation, sevoflurane (2–3%) was introduced for inhalation anaesthesia. Continuous analgesia was maintained through the intravenous infusion of remifentanil (0.15 µg kg^−1^ min^−1^). Upon reaching the surgical anaesthetic plane, the animal was positioned in dorsal recumbency with a midline infraumbilical incision to expose the left kidney. Following heparinization, clamping was performed using clips in the following order: ureter, renal artery and, finally, the renal vein. The vessels were then incised, and the kidney was extracted. Following this, the kidney underwent perfusion with 250 ml of IGL-1 solution at 4 °C. The renal artery and ureter were cannulated to connect them to the normothermic preservation machine. After 3-h ex vivo perfusion, the kidneys undergoing NMP using organoids were disconnected from the device, flushed and cooled with 250 ml of IGL-1. Kidneys were then autotransplanted end-to-side to the infrarenal vena cava and aorta. This involved dissecting 5–8 cm of the vena cava and aorta above the iliac bifurcation. The ureter was cannulated with a double J catheter and anastomosed directly to the bladder. The midline incision was closed, and the pigs were returned to the stables for 24 or 48 h of observation. During postoperative follow-up, analgesia was administered (buprenorphine 50 µg kg^−1^ intravenous twice a day and meloxicam 0.4 mg kg^−1^ intramuscular once a day, along with prophylactic antibiotic therapy (enrofloxacin 2.5 mg kg^−1^ intravenous once a day). After 24 or 48 h, the pigs were anaesthetized, and blood and urine samples were collected. Subsequently, graft and control lateral nephrectomies were performed. The animal was then euthanized under general anaesthesia.

### Collection of allogeneic erythrocytes

Seven days before the autotransplantation schedule, under general anaesthesia, the femoral vein of the animal was cannulated, and 450 ml of blood was extracted using a quadruple bag CPD7SAGm blood filtration system with a lysosome cell death regulator filter (Fresenius blood bag systems, CQ32255), from which approximately 230 ml of red blood cell concentrate was obtained.

### Machine perfusion set-ups

Two different perfusion set-ups were used. On the one hand, for all the experiments shown in Extended Data Figs. 5 and 6, the perfusion circuit was the same as described elsewhere^25^. In brief, it consisted of a pump unit (Medos Deltastream Pumpdrive DP2) and a centrifugal pump (both Medos Medizintechnik AG), an oxygenator/heat exchanger (Dideco Kids D100 neonatal oxygenator, Sorin LivaNova Nederland NV, or Hilite 800 LT, Medos Medizintechnik AG) and a modified LifePort organ chamber with SealRing cannula (Organ Recovery Systems). During each experiment, perfusate temperature was kept at 37 °C. Oxygenation of the perfusate was achieved with carbogen (95% O_2_/ 5% CO_2_). Flow was monitored using an ultrasonic clamp-on flow probe (Transonic Systems). Pressure was measured directly at the SealRing cannula using a clinical-grade pressure sensor (TruWave disposable pressure transducer, Edwards Lifesciences). To obtain a physiological colloid concentration, the set-up was primed using 500 ml Williams’ Medium E (Gibco William’s Medium E + GlutaMAX, Life Technologies) supplemented with amoxicillin–clavulanate 1,000 mg/200 mg (Sandoz B.V.) and 40 g of albumin (bovine serum albumin fraction V, GE Healthcare Bio-Sciences). Then, 350 ml of pure red blood cells were added to the perfusate. Kidneys were perfused in a pressure-controlled, pulsatile (60 bpm) sinusoid fashion at an arterial pressure of 110/70 mm Hg during 4 h. Cold ischaemia time before NMP ranged from 3.5 to 5.0 h. For experiments shown in Figs. 3 and 4 and Extended Data Figs. 7–10, the perfusion circuit used was from EBERS Medical Technology SL (Ark Kidney).

### Tissue collection

Six-millimetre cortical punch biopsies from the upper, middle and lower kidney poles were collected at the end of NMP, and 24 or 48 h after transplantation of kidneys undergoing NMP with or without organoids. In addition, biopsies were collected from the contralateral kidneys. All tissue samples were stored at −80 °C for ulterior experiments for FACS analysis. Samples for DNA isolation were stored at −80 °C. For histological analysis, punch biopsies and tissue samples containing both cortex and medulla from the upper, middle and lower poles were collected and immediately fixed in 4% formalin.

### Immunohistochemistry

Immunohistochemistry analysis of kidney biopsies was performed in the Leica Bond RX automatic platform (Leica). Formalin-fixed paraffin-embedded sections were cut at a thickness of 3 μm, deparaffinized and subjected to antigen retrieval. After blocking endogenous peroxidases, samples were incubated with the primary antibody of interest. Primary antibodies for the detection of CD68, CD15 and CD45 are detailed in Supplementary Table 4. Next, a horseradish peroxidase-labelled secondary antibody and diaminobenzidine as chromogen were used to detect binding of the primary antibody. Finally, samples were counterstained with haematoxylin. Image acquisition of stained samples was performed using the NanoZoomer 2.0 HT C9600 digital scanner (Hamamatsu), equipped with a 20× objective. Whole slide images were imported into the NDP.view2 (U12388-01) Image viewing software^74^. All the immunohistochemistry images from which this analysis has been done are included at ref. ^75^.

### Image analysis and quantification

All raw confocal microscope images were processed with the Fiji ImageJ2 (version 2.3.0) software^76^ using our in-house macro scripts^77^. For optimal image visualization, *Z*-stacks were projected onto a tiff image where the background signal was removed, and brightness and contrast were enhanced. For fluorescent signal quantification, we used our in-house macro scripts^78^. In brief, *Z*-stacks were projected as a sum of all image pixel grey values across the stack, and then selection masks were created at each channel by the SetThreshold tool to measure area using the Measure tool. The measured areas for each indicated marker in kidney organoids were normalized to total organoid areas, which were demarked by the area of 4′,6-diamidino-2-phenylindole (DAPI). For quantification of CD68, CD15 and CD45 in paraffin sections of renal biopsies, immunohistochemistry images were analysed using a custom-built MATLAB code^79^. In brief, images were loaded, and channels were splitted. Images were median-filtered, and the channel of the haematoxylin was subtracted from the channel of the marker to avoid identification of nuclei. Images were Look-Up Table-inverted and subsequently thresholded either using im2binarize for CD45 markers or im2bw in an iterative process when the marker was less present. Based on the average size of the nuclei, a threshold of 30 pixels was introduced for the area detected by regionprops in MATLAB to remove noise and small nuclei, to ultimately retrieve a final accurate occupied area of the analysed markers.

### Blood and observations during NMP

Blood samples were collected at basal and indicated timepoints after transplantation following standard procedures, as previously described^32^. During NMP, renal blood flow was monitored.

### Imaging protocol for the detection of kidney organoids labelled with XenoLight during NMP and after transplantation

For the assessment of kidney organoid distribution ex vivo and after transplantation, kidneys were studied in IVIS Spectrum (PerkinElmer) using the 710 excitation/760 emission filter set to detect Xenolight fluorescence. The fluorescence signal intensities were analysed by Aura Imaging Software (v 4.0.8).

### Detection of human genomic DNA in kidney tissue

DNA was isolated from cortical tissue using a DNeasy Blood & Tissue Kit (Qiagen) according to the manufacturer’s protocol. In total, 10–25 mg of tissue was used. The Y chromosome was detected by RT–PCR using primers directed to the male-specific repeat located on the human Y chromosome (SRY gene loci), SRY F 5′-CATGAACGCATTCATCGTGTGGTC-3′ and SRY R 5′-CTGCGGGAAGCAAACTGCAATTCTT-3′, as published elsewhere^80^.

### Flow cytometry

Cryopreserved cortical kidney biopsies from the NMP experiments were enzymatically disrupted to single cells using collagenase type IV (17104019, Thermo Fisher Scientific) for 45 min at 37 °C. SpectroFlo software was used to acquire flow cytometry samples in the Cytek Aurora spectral cell analyser. FACS FlowJo software version 10 was used to analyse the data. Cryopreserved PBMCs were thawed in RPMI with 20% of FBS in the presence of DNAse (1 mg ml^−1^) (Roche) and washed twice in Dulbecco’s PBS (DPBS). Cells were suspended in DPBS for live dead staining using Live/Dead Blue (Thermo Fisher Scientific) diluted 1:4,000 for 15 min at room temperature. Then, 4–8 × 10^6^ cells were stained in staining buffer (DPBS containing 2% FBS, 4 mM EDTA and 0.02% of sodium azide) with extracellular antibodies from Supplementary Table 7 in ice in the dark for 40 min. After one wash with staining buffer, cells were fixed and permeabilized according to the manufacturer’s instructions using the eBioscience Foxp3/Transcription Factor Staining Buffer Set (Thermo Fisher Scientific). Cells were stained for Ki-67 antibodies for 40 min on ice in the dark, washed twice and resuspended in staining buffer for the acquisition. Samples were acquired on a Cytek five-laser Aurora (Cytek Biosciences) cytometer. Results were analysed using FlowJo software v10.10 (Tree Star).

### In situ hybridization

After NMP, renal cortex samples from porcine kidneys infused with kidney organoids and controls (non-infused) were collected and fixed with 4% paraformaldehyde. Samples were embedded in paraffin following the standard procedure, and 3-μm-thickness sections were prepared using a rotary microtome. Tissue sections were deparaffinized in an automated tissue processor following the standard procedure. Hybridization was performed using a Ventana discovery XT (Roche). For antigen retrieval samples were treated with Cell Conditioning 2 buffer under mild conditions (5279798001, Roche) followed by protease 3 incubation for 20 min at 37 °C (5266718001, Roche). Thereafter, hybridization of the probe Alu-FITC (05278694001, Roche) was performed for 4 min at 37 °C, 8 min at 85 °C and 64 min at 47 °C. Then, samples were washed for 4 min at 45 °C with 2× SSC buffer (05353947001, Roche). Subsequently, samples were incubated with the polyclonal rabbit anti-FITC/HRP (1:500) for 60 min (P5100, Dako – Agilent). Antigen–antibody complexes were detected with Liquid DAB + (K3468, Dako – Agilent). Samples were counterstained with haematoxylin (CS700, Dako – Agilent) and mounted with Mounting Medium, Toluene-Free (CS705, Agilent) using a Dako CoverStainer (Agilent). Images were acquired with a NanoZoomer-2.0 HT C9600 digital scanner (Hamamatsu) equipped with a 20× objective. All images were visualized with a gamma correction set at 1.8 in the image control panel of the NDP.view 2 U12388-01 software (Hamamatsu, Photonics).

### Luminex assay

Serum samples were prepared from whole blood collected from animals before transplantation (basal) and at different timepoints after transplantation. Serum samples were used to measure cytokines, growth factors and inflammatory proteins concentrations using Porcine Luminex Discovery Assays (R&D Systems, cat. no. LXSAPM-01 and LXSAPM-07). Assayed markers included CD31, CD34, interleukin (IL)-6, IL-10, IL-1-RA, IL-1F3, IL-1B, IL-1F2 and CRP. In brief, serum samples were thawed on ice, vortexed and cleared at 1,000*g* for 15 min at 4 °C. Samples were used both undiluted and diluted 1:2 with the diluent provided in the kit. Next, 50 µl of each sample or standard were added to a 96-well assay plate and incubated with 50 µl of antibody-conjugated microparticle cocktail for 2 h at room temperature on a microplate shaker at 800 rpm. Next, three wash steps were performed using a magnetic plate washer. Captured analytes were then probed with 50 µl of the biotin-antibody cocktail for 1 h at room temperature under shaking conditions (800 rpm) and washed three times before treatment with streptavidin–phycoerythrin (PE) for 30 min. The plate underwent a final series of washes after which the microparticles were resuspended in a reading buffer. Concentrations of markers were measured on the Luminex MAGPIX platform with xPONENT software (v 4.3).

### Statistics and reproducibility

All data are presented as mean ± s.d., and other details such as the number of replicates and the level of significance are mentioned in the figure legends. Statistical analysis was done in GraphPad Prism 9 (GraphPad software)^81^. Kruskal–Wallis followed by Dunn’s multiple-comparisons test was used to analyse intergroup differences. *P* values less than 0.05 were considered statistically significant. All data were analysed from at least three individual experiments unless stated otherwise.

### Reporting summary

Further information on research design is available in the Nature Portfolio Reporting Summary linked to this article.

## Supplementary information

Reporting Summary

Peer Review File

Supplementary Tables 1–7 Supplementary Tables 1–7.

## Source data

Source Data Fig. 1 Statistical source data for the immunofluorescence quantifications shown in panels c and k, RT–qPCR analysis shown in panel g, seahorse analysis shown in panel h and flow cytometry quantification shown in panel k.

Source Data Fig. 2 Statistical source data for MiSeq quantification shown in panel b, and RT–qPCR analysis shown in panels d, j and m.

Source Data Fig. 3 Statistical source data for renal arterial flow analysis shown in panel b, LDH level quantification in panel c, IVIS image quantification in panel f and flow cytometry quantification in panel i.

Source Data Fig. 4 Statistical source data for quantification of PBMCs shown in panels a and b, analysis of protein serum levels shown in panel c and image quantification shown in panel d.

Source Data Extended Data Fig. 1 Statistical source data for RT–qPCR analysis shown in panel c.

Source Data Extended Data Fig. 3 Statistical source data for RT–qPCR analysis shown in panel c and flow cytometry quantification shown in panel f.

Source Data Extended Data Fig. 4 Statistical source data for FACS experiments shown in panels g and i.

Source Data Extended Data Fig. 4 Unprocessed gels shown in panel d.

Source Data Extended Data Fig. 5 Statistical source data for renal arterial flow analysis shown in panel d, and quantification of NMP parameters shown in panels e–i.

Source Data Extended Data Fig. 6 Statistical source data for renal arterial flow analysis shown in panel c, and quantification of NMP parameters shown in panels d–h.

Source Data Extended Data Fig. 7 Statistical source data for renal arterial flow analysis shown in panel f, quantification of NMP parameters shown in panels g–k, IVIS image quantification shown in panel m and flow cytometry quantification shown in panel o.

Source Data Extended Data Fig. 8 Statistical source data for quantification of NMP parameters shown in panels c–f.

Source Data Extended Data Fig. 9 Statistical source data for blood biochemical analysis shown in panel b, and urine biochemical analysis shown in panel c.

Source Data Extended Data Fig. 9 Unprocessed gels shown in panel a.

Source Data Extended Data Fig. 10 Statistical source data for quantification of PBMCs shown in panels b and c, and analysis of protein serum levels shown in panel d.

## Data Availability

The authors declare that all data supporting the findings of this study are available within the article and its extended data figures and supplementary tables. scRNA-seq data are publicly available via Gene Expression Omnibus (GEO) at http://www.ncbi.nlm.nih.gov/geo under the accession number GSE229433. For kidney organoids generated from 8,000 and 100,000 IM-committed spheroids, raw sequencing data were previously deposited in GEO under the accession numbers GSM4447249 (8,000 condition)^4^ and GSM4625992 (100,000 condition)^3^. Reference scRNA-seq data were obtained via Kidney Interactive Transcriptomics (KIT) webpage at http://humphreyslab.com/SingleCell/ and from previous publications as detailed in Methods. An OSF link to the immunohistochemistry images for the detection of CD68, CD15 and CD45, from which quantitative analysis was done, is included (https://osf.io/n7xk4/). Source data are provided with this paper.

## Code availability

The custom code for image analysis and quantification is available via Zenodo at https://doi.org/10.5281/zenodo.7689048 (ref. ^77^) and https://doi.org/10.5281/zenodo.7689082 (ref. ^78^) and via GitHub at https://github.com/Roger-Oria (ref. ^79^).
