# Supplementary material for: Systematic production of human kidney organoids for transplantation in porcine kidneys during ex vivo machine perfusion
Source: Nat Biomed Eng. 2025 Oct 31;10(10):2230–47. doi: 10.1038/s41551-025-01542-1 (PMC13672120; doi:10.1038/s41551-025-01542-1)
Supplement: Supplementary file 1 [file 41551_2025_1542_MOESM11_ESM.pdf]

Extended Data Fig.4d

Left gels

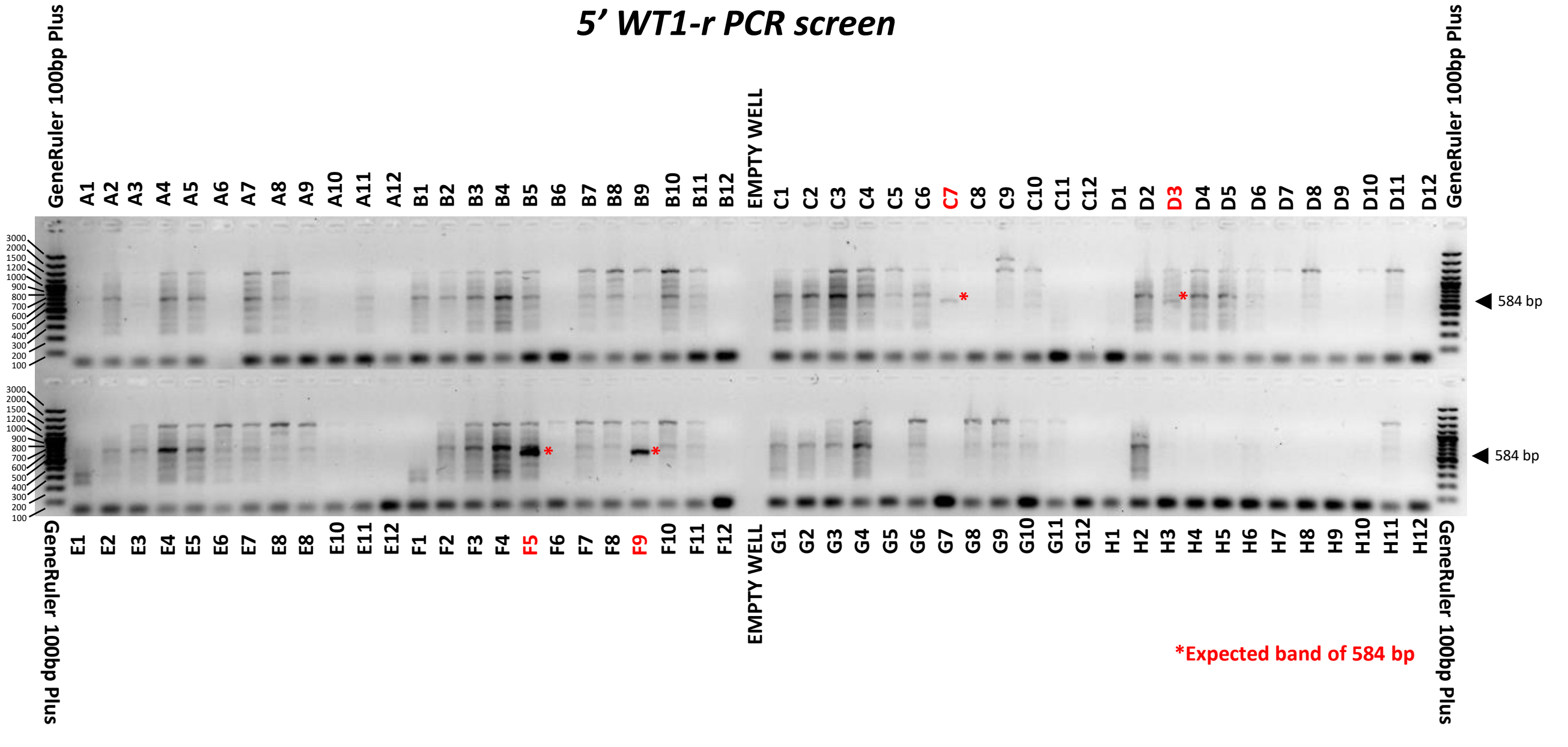

Extended Data Fig.4d

Right gels

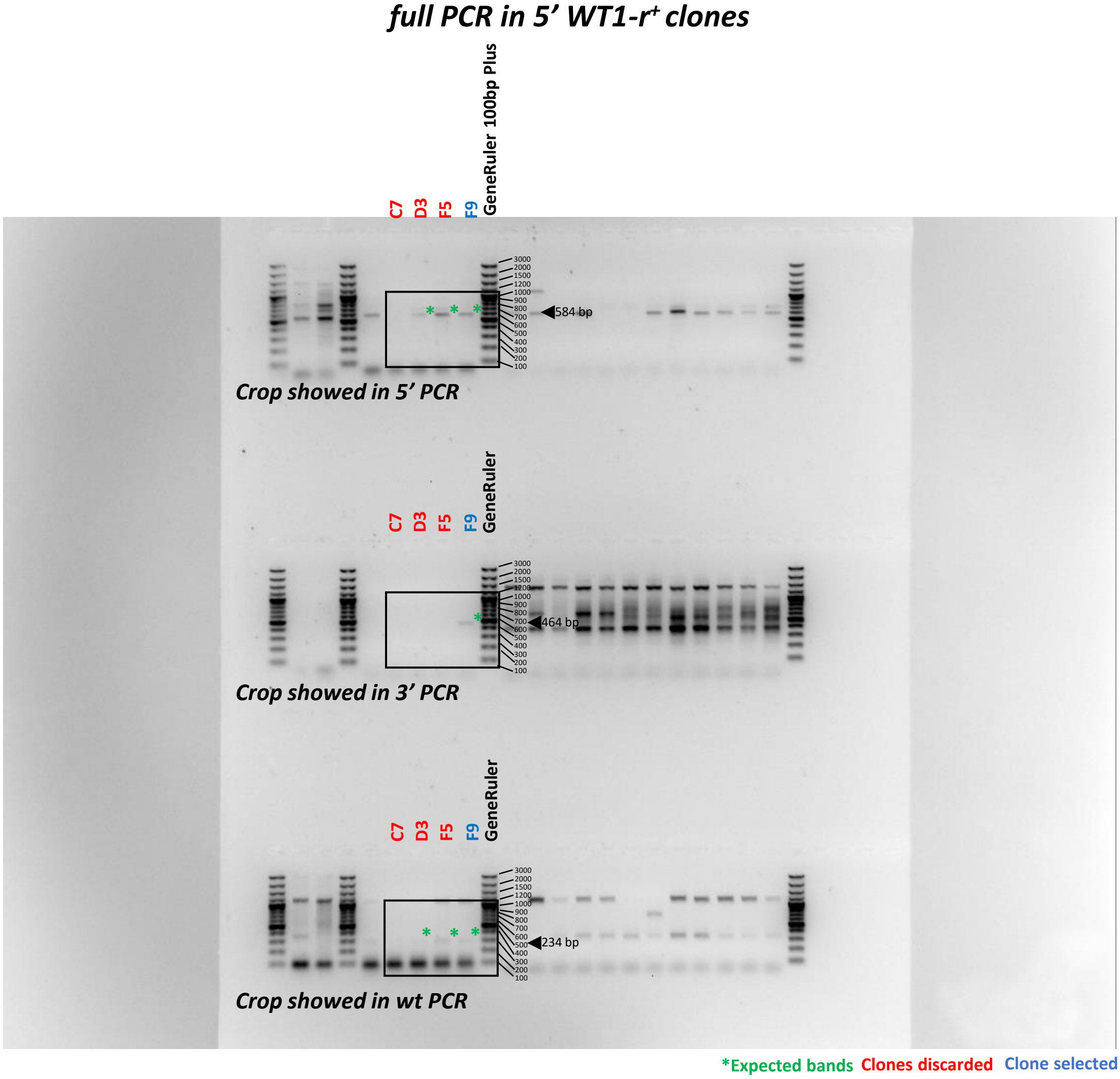

Rest of PCRs runned in the gel are unrelated to the data shown in the manuscript
