# Supplementary material for: Systematic production of human kidney organoids for transplantation in porcine kidneys during ex vivo machine perfusion
Source: Nat Biomed Eng. 2025 Oct 31;10(10):2230–47. doi: 10.1038/s41551-025-01542-1 (PMC13672120; doi:10.1038/s41551-025-01542-1)

Extended Data Fig. 9a

Left gel

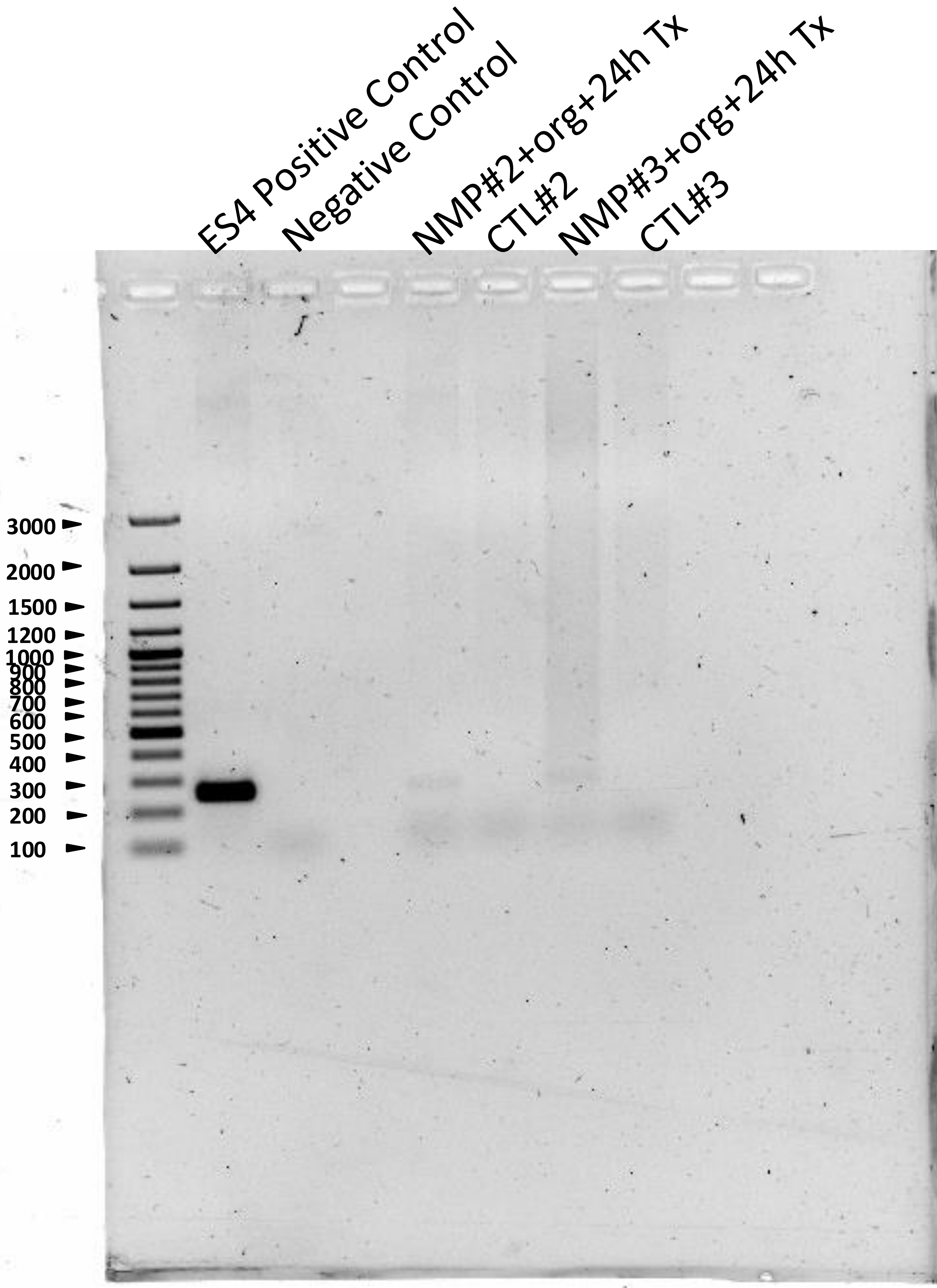

Extended Data Fig. 9a

Middle gel

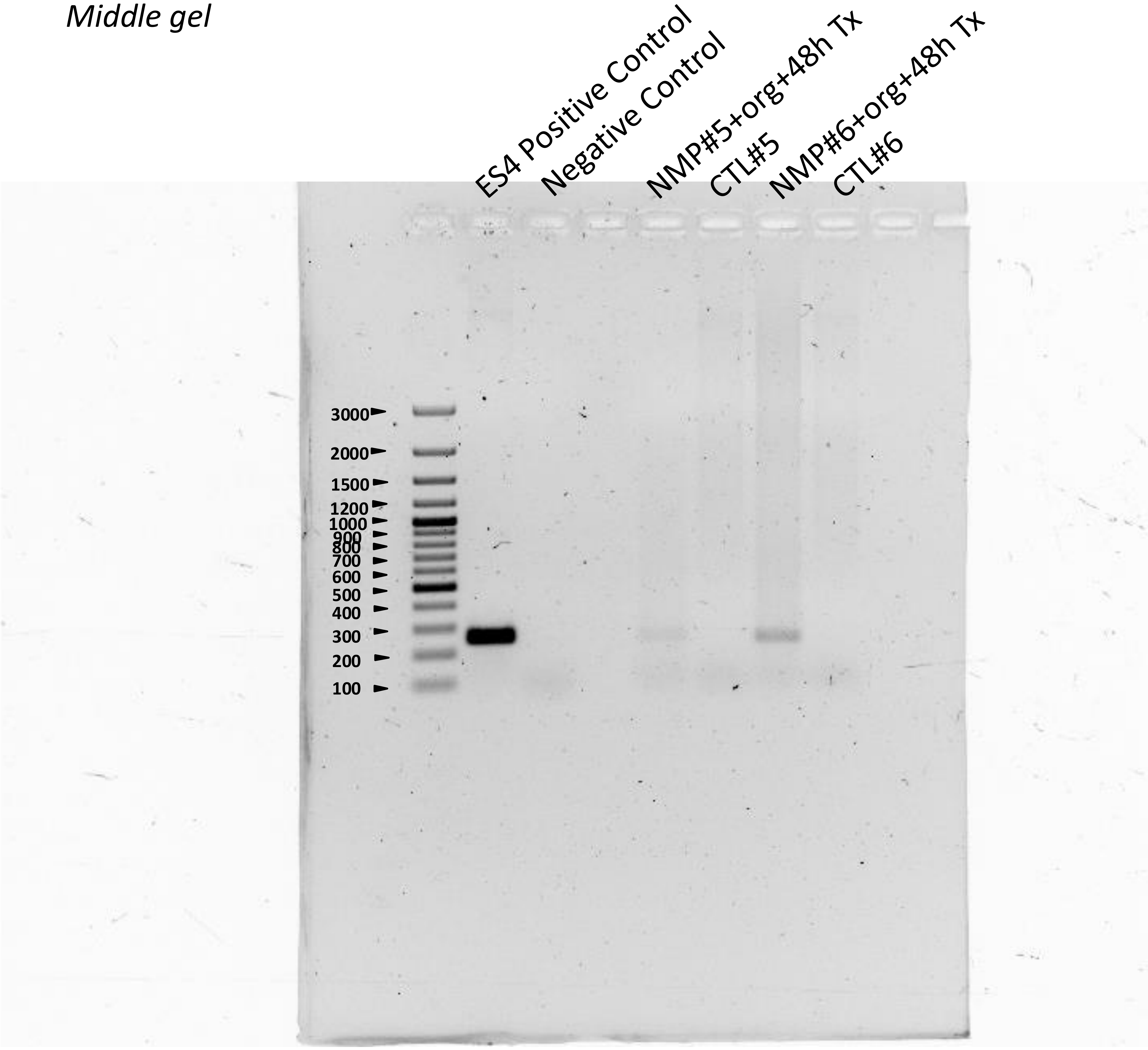

Extended Data Fig. 9a

Right gel

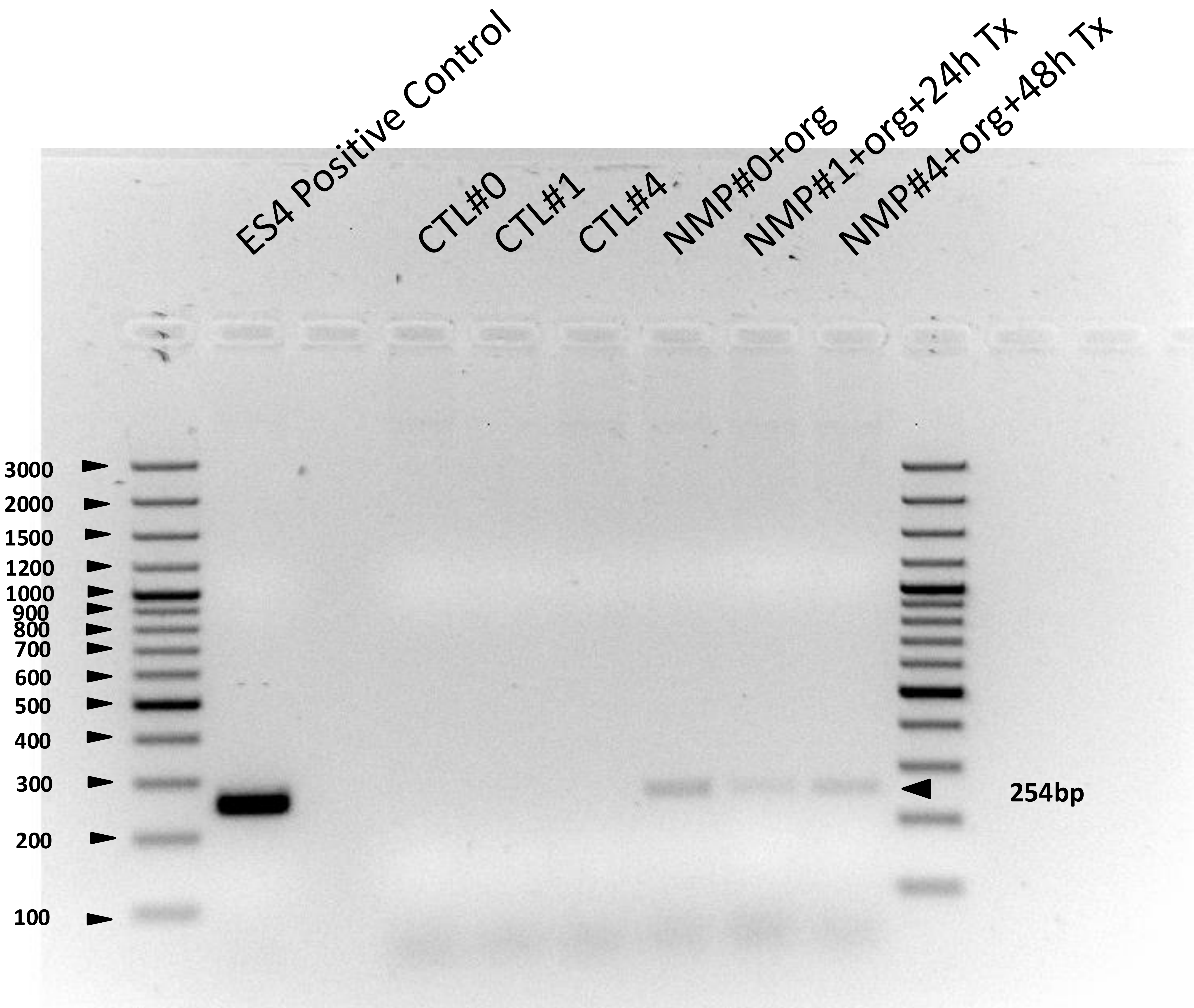

Supplement: Supplementary file 2 [file 41551_2025_1542_MOESM17_ESM.pdf]
