# Supplementary material for: Systematic production of human kidney organoids for transplantation in porcine kidneys during ex vivo machine perfusion
Source: Nat Biomed Eng. 2025 Oct 31;10(10):2230–47. doi: 10.1038/s41551-025-01542-1 (PMC13672120; doi:10.1038/s41551-025-01542-1)
Supplement: Supplementary file 3 [file 41551_2025_1542_MOESM1_ESM.pdf]

nature portfolio

Corresponding author(s): Nuria Montserrat

Last updated by author(s): Jul 15, 2025

Reporting Summary

Nature Portfolio wishes to improve the reproducibility of the work that we publish. This form provides structure for consistency and transparency in reporting. For further information on Nature Portfolio policies, see our Editorial Policies and the Editorial Policy Checklist.

Statistics

For all statistical analyses, confirm that the following items are present in the figure legend, table legend, main text, or Methods section.

n/a	Confirmed	
☐	☒	The exact sample size (*n*) for each experimental group/condition, given as a discrete number and unit of measurement
☐	☒	A statement on whether measurements were taken from distinct samples or whether the same sample was measured repeatedly
☐	☒	The statistical test(s) used AND whether they are one- or two-sided<br>*Only common tests should be described solely by name; describe more complex techniques in the Methods section.*
☐	☒	A description of all covariates tested
☐	☒	A description of any assumptions or corrections, such as tests of normality and adjustment for multiple comparisons
☐	☒	A full description of the statistical parameters including central tendency (e.g. means) or other basic estimates (e.g. regression coefficient) AND variation (e.g. standard deviation) or associated estimates of uncertainty (e.g. confidence intervals)
☐	☒	For null hypothesis testing, the test statistic (e.g. *F*, *t*, *r*) with confidence intervals, effect sizes, degrees of freedom and *P* value noted<br>*Give P values as exact values whenever suitable.*
☒	☐	For Bayesian analysis, information on the choice of priors and Markov chain Monte Carlo settings
☒	☐	For hierarchical and complex designs, identification of the appropriate level for tests and full reporting of outcomes
☒	☐	Estimates of effect sizes (e.g. Cohen's *d*, Pearson's *r*), indicating how they were calculated

Our web collection on statistics for biologists contains articles on many of the points above.

Software and code

Policy information about availability of computer code

Data collection	SpectroFlo® software was used for flow citometry. NanoZoomer-2.0 HT C9600 digital scanner (Hamamatsu) equipped with a 20X objective was used for histology image acquisition. Cell^D (v 3.2) and LAS EZ (v 3.4.0) were used for optical microscopy. Design nand Analysis QuantStudio Real time PCR software (v 1.5.2) was used for qPCR data collection. NiS elements (v 5.42.04) and ZEN Black (v 2.3) were used for confocal microscopy. IVIS Spectrum (Perkin Elmer) using the 710 ex/760 em filter was used to detect Xenolight fluorescence.
Data analysis	FlowJoTM (v 10.10) was used for flow cytometry data analysis.<br>Seahorse wave desktop software (v 2.6) was used for OCR data collection and analysis.<br>The NDP.view 2 U12388-01 software (Hamamatsu, Photonics, France) was used for histological analysis after immunohistochemistry and FISH.<br>Microsoft excel (v 16.81 or greater) was used for qPCR and NMP data analysis.<br>Graphpad Prism (v. 9.4.1) was used for graphing and statistical analysis.<br>Indel frequency in hPSC edited pools was obtained by analyzing trimmed FASTQ reads using CRISPResso2 (v 2.2.7) software.<br>Cell Ranger (v 3.0.1), R package Seurat (v 4.0.5), scDblFinder (v1.9.4), R package enrichR (v3.0) were used for scRNA-seq data analysis as described in the Methods. cDNA libraries were pre-processed using Cell Ranger (3.0.1) from 10X Genomics (http://10xgenomics.com). Reads from each sample were aligned to the reference human genome (GRch38) downloaded from the 10X Genomics website (version 3.0.0, corresponding to Ensembl v93 annotation).<br>IVIS image analysis was performed using Aura Imaging Software® (v 4.0.8).<br>Luminex MAGPIX platform with xPONENT® software (v 4.3) was used for the milliplex assay.<br>All raw confocal microscope images were processed with the Fiji ImageJ2 (version 2.3.0) software (https://imagej.net/software/fiji/) using our in-house macro scripts (https://doi.org/10.5281/zenodo.7689048). For optimal image visualization, Z-stacks were projected onto a tiff image where the background signal was removed, and brightness and contrast were enhanced. For fluorescent signal quantification we used our in-house macro scripts (https://doi.org/10.5281/zenodo.7689082). A description of the procedures is available in Methods section of the

manuscript.
For quantification of CD68, CD15 and CD45 in paraffin sections of renal biopsies, immunohistochemistry images were analyzed using a custom-build Matlab code (https://github.com/Roger-Oria).

For manuscripts utilizing custom algorithms or software that are central to the research but not yet described in published literature, software must be made available to editors and reviewers. We strongly encourage code deposition in a community repository (e.g. GitHub). See the Nature Portfolio guidelines for submitting code & software for further information.

Data

Policy information about availability of data

All manuscripts must include a data availability statement. This statement should provide the following information, where applicable:

- Accession codes, unique identifiers, or web links for publicly available datasets
- A description of any restrictions on data availability
- For clinical datasets or third party data, please ensure that the statement adheres to our policy

The authors declare that all data supporting the findings of this study are available within the Article and its Extended Data figures and Supplementary tables. Source data are provided with this paper. scRNA-Seq data are publicy available in Gene Expression Omnibus (GEO, http://www.ncbi.nlm.nih.gov/geo) under the accession number GSE229433. For kidney organoids generated from 8-Ks and 100-Ks IM-committed spheroids, raw sequencing data were previously deposited in GEO under the accession numbers GSM4447249 (8-Ks condition) and GSM4625992 (100-Ks condition). Reference scRNA-seq data was obtained from the KIT (Kidney Interactive Transcriptomics) webpage [http://humphreyslab.com/SingleCell/] and from previous publications as detailed in Methods section in this study. An OSF link to the immunohistochemistry images for the detection of CD68, CD15 and CD45, from which quantitative analysis was done is included (https://osf.io/n7xk4/).

Research involving human participants, their data, or biological material

Policy information about studies with human participants or human data. See also policy information about sex, gender (identity/presentation), and sexual orientation and race, ethnicity and racism.

Reporting on sex and gender	Not applicable
Reporting on race, ethnicity, or other socially relevant groupings	Not applicable
Population characteristics	Not applicable
Recruitment	Not applicable
Ethics oversight	Not applicable

Note that full information on the approval of the study protocol must also be provided in the manuscript.

Field-specific reporting

Please select the one below that is the best fit for your research. If you are not sure, read the appropriate sections before making your selection.

☒ Life sciences ☐ Behavioural & social sciences ☐ Ecological, evolutionary & environmental sciences

For a reference copy of the document with all sections, see nature.com/documents/nr-reporting-summary-flat.pdf

Life sciences study design

All studies must disclose on these points even when the disclosure is negative.

Sample size

No statistical methods were used to determine sample size. All sample sizes, statistical tests and P values (when applicable) are indicated in the figure legends. All data were analysed from at least 3 individual experiments unless stated otherwise.
For in vitro experiments, pools of 12 organoids were analyzed at the time points and conditions indicated in the manuscript for each experiment. Sample size was determined based on previous studies in the field.
Relevant citations include:
- Garreta E, Prado P, Tarantino C, Oria R, Fanlo L, Martí E, Zalvidea D, Trepat X, Roca-Cusachs P, Gavaldà-Navarro A, Cozzuto L, Campistol JM, Izpisúa Belmonte JC, Hurtado Del Pozo C, Montserrat N. Fine tuning the extracellular environment accelerates the derivation of kidney organoids from human pluripotent stem cells. Nat Mater. 2019 Apr;18(4):397-405. doi: 10.1038/s41563-019-0287-6. Epub 2019 Feb 18. PMID: 30778227; PMCID: PMC9845070.
- Garreta E, Prado P, Stanifer ML, Monteil V, Marco A, Ullate-Agote A, Moya-Rull D, Vilas-Zornoza A, Tarantino C, Romero JP, Jonsson G, Oria R, Leopoldi A, Hagelkruys A, Gallo M, González F, Domingo-Pedrol P, Gavaldà A, Del Pozo CH, Hasan Ali O, Ventura-Aguiar P, Campistol JM, Prosper F, Mirazimi A, Boulant S, Penninger JM, Montserrat N. A diabetic milieu increases ACE2 expression and cellular susceptibility to SARS-CoV-2 infections in human kidney organoids and patient cells. Cell Metab. 2022 Jun 7;34(6):857-873.e9. doi: 10.1016/j.cmet.2022.04.009. Epub 2022 May 12. PMID: 35561674; PMCID: PMC9097013.

For ex vivo NMP: For experiments shown in Extended Data Fig. 5 and Extended Data Fig. 6, in the first group kidneys underwent 3h of NMP without organoid infusion (n= 2). In the second group kidneys underwent 3h of NMP with organoid infusion at low dose (36 organoids/gr of renal tissue; n= 3). In the third group kidneys underwent 3h of NMP with organoid infusion at high dose (154 organoids/gr of renal tissue; n= 1). In the fourth group kidneys underwent 3h of NMP with infusion of cells obtained by enzymatic dissociation of organoids (n= 2). Punch biposies (n=3) and tissue samples containing both medulla and cortex (n= 3) were analyzed per kidney. Sample size was determined based on previous studies in the field. For in vivo transplantation experiments, shown in Fig. 3, and Extended Data Fig. 7 to Extended data Fig. 10, kidneys from female laboratory pigs (Hybrid Landrace/Large White crossbreed; 12 to 14 weeks of age; n= 7 animals) were randomly assigned to three experimental groups. All the groups were subjected to a phase of organ conditioning (30 min). In the non-transplanted group, a kidney underwent 3h of NMP with organoid infusion (n= 1). In the second and third experimental groups kidneys underwent 3h of NMP with organoid infusion followed by autotransplantation and graft retrieval at 24 h (n=3) or 48 h (n=3) after transplantation, respectively. Punch biopsies (n=3) and tissue samples containing both medulla and cortex (n= 3) were analyzed per kidney. Sample size was determined based on previous studies in the field.
Relevant citations include:
- Clavien, P.-A. et al. Transplantation of a human liver following 3 days of ex situ normothermic preservation. Nat Biotechnol 40, 1610–1616 (2022).
- Pool, M. B. F., Hartveld, L., Leuvenink, H. G. D. & Moers, C. Normothermic machine perfusion of ischaemically damaged porcine kidneys with autologous, allogeneic porcine and human red blood cells. PLoS One 15, (2020).
- Lohmann, S. et al. A Pilot Study of Postoperative Animal Welfare as a Guidance Tool in the Development of a Kidney Autotransplantation Model with Extended Warm Ischemia. Transplant Direct 5, (2019).
- de Haan, M. J. A. et al. A cell-free nutrient-supplemented perfusate allows four-day ex vivo metabolic preservation of human kidneys. Nat Commun 15, 3818 (2024).

Data exclusions: No data were excluded from the analyses.

Replication: In vitro experimental findings were reproduced independently at least two times. All attempts at replication were successful. To ensure the reproducibility of our methodology for generating kidney organoids, we used a human embryonic stem cell (hESC) line and a human induced pluripotent stem cell line. Ex vivo NMP and in vivo experimental findings were reproduced independently at least two times.

Randomization: Cells/organoids were chosen at random for measurements within each condition. For in vivo transplantation experiments, animals were allocated randomly into experimental groups.

Blinding: Blinding was not used during data collection and analysis in experiments that did not involve direct comparisons between groups. Blinding was used for quantitative measurements comparing different conditions or for the detection of Alu positive cells, in which data analysis was carried out using a custom-made code (Fig. 1c,j; Fig. 3g,h; Fig. 4d; Extended Data Fig. 5j,k; Extended Data Fig. 6i; Extended Data Fig. 7n; Extended Data Fig. 8h,i).

Reporting for specific materials, systems and methods

We require information from authors about some types of materials, experimental systems and methods used in many studies. Here, indicate whether each material, system or method listed is relevant to your study. If you are not sure if a list item applies to your research, read the appropriate section before selecting a response.

Materials & experimental systems

n/a	Involved in the study	
☐	☒	Antibodies
☐	☒	Eukaryotic cell lines
☒	☐	Palaeontology and archaeology
☐	☒	Animals and other organisms
☒	☐	Clinical data
☒	☐	Dual use research of concern
☒	☐	Plants

Methods

n/a	Involved in the study	
☒	☐	ChIP-seq
☐	☒	Flow cytometry
☒	☐	MRI-based neuroimaging

Antibodies

Antibodies used: Antibodies and other staining reagents used for immunocytochemistry/ immunohistochemistry:
PAX2 (R&D Systems, Cat# AF3364, polyclonal, Lot# XOT0215072, Dilution 1:20);
LHX1 (R&D Systems, Cat# MAB2725 RRID:AB_2135636, # 320416, Lot# XMP0321121, Dilution 1:9);
WT1 (Abcam, Cat# ab89901, clone CAN-R9(IHC)-56-2, Lot# GR177328-54, Dilution 1:100);
ECAD (BD Bioscience, Cat# 610181, clone 36/E-cadherine, Lot# 7187865, Dilution 1:50);
PODXL (R&D Systems, Cat# BAF1658, polyclonal, Lot# JLV0112111, Dilution 1:25);
NEPHRIN (R&D Systems, Cat# AF4269, polyclonal, Lot# ZMU0114031, Dilution 1:300);
CD31 (Abcam, Cat# ab28364, polyclonal, Lot# GR31176844-16, Dilution 1:50);
Laminin (Sigma, Cat# L9393, polyclonal, Lot# 028M4890V, Dilution 1:50);GATA3 (Cell Signalling, Cat# 5852S RRID:AB_10835690,D13C9, Lot# 6, Dilution 1:300);
GFP (Aves, Cat# GFP-1020 RRID:AB_10000240, polyclonal, Lot# GFP3717982, Dilution 1:250);
Ki67 (Abcam, Cat# ab15580 RRID:AB_443209, polyclonal, Lot# GR3445754-1, Dilution 1:500);
NANOG (R&D Systems, Cat# AF1997 RRID:AB_355097, polyclonal, Lot# KKJ0922031, Dilution 1:25);

TIM-1/KIM-1/HAVCR R&D AF 1750 RRID:AB_2795238 Polyclonal 1:300
CD68 Agilent M081401-2 RRID:AB_2750584 , cloe KP1 1:100
CD15 Agilent GA06261-2 Carb-3 Ready to use
CD45 Bio-RAD MCA1222GA RRID:AB_3100021 , clone K252.1E4 1:100
Biotinylated Lotus Tetragonolobus Lectin (LTL) (Vector Laboratories, Cat# B-1325, Lot# ZC2428, Dilution 1:200)
Fluorescein labeled LTL (Vector Laboratories, Cat# FL-1321, Lot# ZC0914, Dilution: 1:500)

Secondary Antibodies:
Anti-Goat Alexa Fluor 488-conjugated (Jackson ImmunoResearch, Cat# 705-545-147; RRID:AB_2336933, Dilution 1:200);
Anti-Goat IgG Alexa Fluor 555-conjugated (Fisher Scientific, Cat# A-21432; RRID:AB_2535853 , Dilution 1:200;
Anti-rabbit IgG Alexa fluor 488-conjugated (Fisher Scientific, Cat# A21206; RRID:AB_2535792, Dilution 1:200);
Anti-rabbit IgG Alexa fluor 555-conjugated (Fisher Scientific, Cat# A-31572; RRID:AB_162543, Dilution 1:200;
Anti-Mouse IgG CyTM3-conjugated (Jackson ImmunoResearch , Cat# 715-165-151; RRID:AB_2315777, Dilution 1:200);
Anti-Sheep IgG Alexa Fluor 555-conjugated (Fisher Scientific, Cat# A-21436; RRID:AB_2535857, Dilution 1:200;
Dylight 649 Streptavidin (Vector Labs, Cat# SA-5649; RRID:AB_2336421, Dilution 1:200);
Alexa Fluor 488-conjugated streptavidin (Vector Laboratories, Cat# SA5488, Lot# ZD0313, Dilution: 1:50);

Antibodies and other staining reagents used for flow cytometry:
IVISense DiR 750 Fluorescent Cell Labeling Dye-XenoLight, 125964, Perkin Elmer.
IVISense 680 Fluorescent Cell Labeling Dye (NEV12001, Perkin Elmer).
Qtracker 655® (Cell Labelling kits, Thermo Fisher Scientific, Landsmeer, Netherlands)
Qtracker 800® (Cell Labelling kits, Thermo Fisher Scientific, Landsmeer, Netherlands).
DAPI (Invitrogen, D1306).
CD45, clone K252-1E4, FITC, Bio-Rad ,dilution 400
CD3, clone PPT3, StarBright Violet 515, Bio-RAD , dilution 25
CD4, clone 74-12-4 , PE-Cy7, BD Biosciences, dilution 400
CD8a, clone 76-2-11, PE, BD , dilution 200
CD14, clone TUK4, APC-Fluor 750 , Thermo Fisher , dilution 24
CD16, clone G7, StarBright Violet 570 Bio-RAD, dilution 50
Ki-67, V450 , dilution 400

Antibodies and other staining reagents used for in situ hybridization:
Alu-FITC (05278694001, Roche)
Polyclonal Rabbit Anti-FITC/HRP (1:500) (P5100, Dako – Agilent).

Validation

Antibody validations for the species and assay used were performed by antibody suppliers as described in the manufacture's web page, or were published in previous studies. Relevant articles are:
- Garreta E, Prado P, Tarantino C, Oria R, Fanlo L, Martí E, Zalvidea D, Trepat X, Roca-Cusachs P, Gavaldà-Navarro A, Cozzuto L, Campistol JM, Izpisúa Belmonte JC, Hurtado Del Pozo C, Montserrat N. Fine tuning the extracellular environment accelerates the derivation of kidney organoids from human pluripotent stem cells. Nat Mater. 2019 Apr;18(4):397-405. doi: 10.1038/s41563-019-0287-6. Epub 2019 Feb 18. PMID: 30778227; PMCID: PMC9845070.
- Garreta E, Prado P, Stanifer ML, Monteil V, Marco A, Ullate-Agote A, Moya-Rull D, Vilas-Zornoza A, Tarantino C, Romero JP, Jonsson G, Oria R, Leopoldi A, Hagelkruys A, Gallo M, González F, Domingo-Pedrol P, Gavaldà A, Del Pozo CH, Hasan Ali O, Ventura-Aguiar P, Campistol JM, Prosper F, Mirazimi A, Boulant S, Penninger JM, Montserrat N. A diabetic milieu increases ACE2 expression and cellular susceptibility to SARS-CoV-2 infections in human kidney organoids and patient cells. Cell Metab. 2022 Jun 7;34(6):857-873.e9. doi: 10.1016/j.cmet.2022.04.009. Epub 2022 May 12. PMID: 35561674; PMCID: PMC9097013.
- Garreta E, Moya-Rull D, Stanifer ML, Monteil V, Prado P, Marco A, Tarantino C, Gallo M, Jonsson G, Hagelkruys A, Mirazimi A, Boulant S, Penninger JM, Montserrat N. Protocol for SARS-CoV-2 infection of kidney organoids derived from human pluripotent stem cells. STAR Protoc. 2022 Dec 16;3(4):101872. doi: 10.1016/j.xpro.2022.101872. Epub 2022 Nov 7. PMID: 36595951; PMCID: PMC9637521.
- Kalchenko et al., Use of lipophilic near-infrared dye in whole-body optical imaging of hematopoietic cell homing. Journal of Biomedical Optics, September/October 2006, Vol 11(5).

Eukaryotic cell lines

Policy information about cell lines and Sex and Gender in Research

Cell line source(s)

Human pluripotent stem cell lines:
All the human pluripotent stem cell lines used in this study were obtained after the approval of Ethics Committee from the Clinical Translational Program for Regenerative Medicine in Catalonia (P-CMR [C]) and the Comisión de Seguimiento y Control de la Donación de Células y Tejidos Humanos del Instituto de Salud Carlos III (project numbers: 0036/3897/2015; 0336/1123/2021; 0336/2723/2021; 336/165/2022; 9067/487286/2022). ES[4] hESC and CBiPSsv-4F-40 were obtained from The National Bank of Stem Cells (ISCIII, Madrid). Information on cell line sources used in this study is also provided in the Methods section of the manuscript.

Authentication

Human pluripotent stem cell lines:
Human embryonic stem cells (ES[4] hESC line; https://www.isciii.es/QueHacemos/Servicios/BIOBANCOS/BNLC/Lists/Lneas%20embrionarias/Attachments/41/Caracteristicas%20-%20ES4.pdf) and human induced pluripotent stem cells (CBiPSsv-4F-40 line; https://hpscreg.eu/cell-line/ESi007-A) were authenticated in their lab of origin through the expression of pluripotency-associated markers, and in vitro and in vivo differentiation assays towards lineages of the three embryonic layers. The WT1

	reporter cell line was established in the ES[4] hESC background. The line has been characterized in this study.
Mycoplasma contamination	All cell lines tested negative for mycoplasma contamination.
Commonly misidentified lines (See ICLAC register)	No commonly misidentified cell lines were used.

Animals and other research organisms

Policy information about studies involving animals; ARRIVE guidelines recommended for reporting animal research, and Sex and Gender in Research

Laboratory animals	Female laboratory pigs (Hybrid Landrace/Large White crossbreed; 12 to 14 weeks of age) weighing approximately 50 kg were used for autotransplantation.
Wild animals	The study does not involve wild animals.
Reporting on sex	Female laboratory pigs were used for autotransplantation.
Field-collected samples	The study does not involve samples collected from the field.
Ethics oversight	Female laboratory pigs (Hybrid Landrace/Large White crossbreed; 12 to 14 weeks of age) weighing approximately 50 kg were used for autotransplantation. All animal care and procedures followed guidelines by the European Union (directive 2010/63/EU) and local regulations. The study was approved by the Ethical Committee of Animal Experimentation of the University Hospital Complex of A Coruña (reference-number 15002/2023/04). All personnel involved had Federation of European Laboratory Animal Science Associations licenses.

Note that full information on the approval of the study protocol must also be provided in the manuscript.

Plants

Seed stocks	Not applicable
Novel plant genotypes	Not applicable
Authentication	Not applicable

Flow Cytometry

Plots

Confirm that:

☒ The axis labels state the marker and fluorochrome used (e.g. CD4-FITC).

☒ The axis scales are clearly visible. Include numbers along axes only for bottom left plot of group (a 'group' is an analysis of identical markers).

☒ All plots are contour plots with outliers or pseudocolor plots.

☒ A numerical value for number of cells or percentage (with statistics) is provided.

Methodology

Sample preparation	Fig. 1k:<br>Kidney organoids were stained with fluorescein-conjugated LTL (FL-1321, Vector Laboratories) for 4 hours. Kidney organoids were then dissociated to single cells using accumax (07921, Stem Cell Technologies) for 15 min followed by 0.25% (wt/vol) trypsin (25300-054, Life Technologies) for 15 min at 37ºC.<br>Fig. 3i and Extended Data Fig. 7o:<br>Kidney organoids were stained using 50 µg/ml of Xenolight 750 for 30 min according to the methodology described in the Methods section of the manuscript. Kidney organoids were then dissociated to single cells using Accumax (07921, Stem Cell Technologies) for 15 min at 37ºC and Trypsin-EDTA 0.25% (wt/vol) trypsin (25300-054, Life Technologies) for additional 15 min at 37ºC. Cryopreserved cortical kidney biopsies from the NMP experiments were enzymatically disrupted to single cells using collagenase type IV (17104019, Thermo Fisher) for 45 min at 37 °C.<br>Figure 4 and Extended Data Fig.10:<br>Cryopreserved PBMCs were thawed in RPMI with 20% of FBS in the presence of DNAse (1mg/ml) (Roche) and washed twice

in DPBS. Cells were suspended in DPBS for live dead staining using Live/Dead Blue (Thermo Fisher Scientific) diluted 1:4000 for 15 minutes at room temperature. Then 4-8·10^6 cells were stained in staining buffer (DPBS containing 2% FBS, 4mM EDTA and 0.02% of sodium azide) with extracellular antibodies in ice in the dark for 40 min. After one wash with staining buffer cells were fix and permeabilized according to manufacturer instructions using the eBioscience™ Foxp3 / Transcription Factor Staining Buffer Set (Thermo Fisher Scientific). Cells were stained for Ki-67 antibodies for 40 minutes in ice in the dark, washed twice and resuspended in staining buffer for the acquisition. Samples were acquired on a Cytek™ 5-laser Aurora (Cytek Biosciences) cytometer. Results were analyzed using FlowJo software v10.10 (Tree Star Inc).

Instrument

Cytek® Aurora spectral cell analyzer was used to acquire flow cytometry samples (Fig. 3i; Extended Data Fig. 7o).
Cytek™ 5-laser Aurora (Cytek Biosciences) cytometer was used to acquire flow cytometry samples in Figure 4 and Extended Data Fig. 10.

Software

SpectroFlo® software was used to acquire flow cytometry samples in the Cytek® Aurora spectral cell analyzer. FlowJo software version 10 was used to analyze these data.

Cell population abundance

The abundance of the Xenolight 750 positive cell population was also confirmed by the detection of Xenolight 750 positive cells by confocal microscopy.

Gating strategy

Fig. 1k:
For analysis of LTL-FITC positive and negative cell fractions, cells were first gated on a SSC-A versus
FSC-A plot, the polulation from which was then gated on singlets (FSC-W versus FSC-A plot), the population from which was then gated on the LIVE/DAD negative population (LIVE/DEAD versus FSC-A plot), the population from which was then analyzed on a plot of autofluorescence (AU) versus LTL-FITC.
Fig. 3i and Extended Data Fig. 7o:
For assessment of Xenolight 750 staining, cells were first gated on an SSC-A versus FSC-A plot, the population from which was then gated on the LIVE/DEAD negative population (LIVE/DEAD versus FSC-A plot), the population from which was then analyzed on a plot of SSC-A versus Xenolight 750.
Figure 4 and Extended Data Fig. 10:
Flow cytometry gating strategy for analyzing peripheral blood mononuclear cells of pigs at different timepoints after in vivo engraftment is shown in Extended Data Fig. 10a.

☒ Tick this box to confirm that a figure exemplifying the gating strategy is provided in the Supplementary Information.
