# Supplementary material for: Systematic production of human kidney organoids for transplantation in porcine kidneys during ex vivo machine perfusion
Source: Nat Biomed Eng. 2025 Oct 31;10(10):2230–47. doi: 10.1038/s41551-025-01542-1 (PMC13672120; doi:10.1038/s41551-025-01542-1)
Supplement: Supplementary file 4 [file 41551_2025_1542_MOESM2_ESM.pdf]

nature portfolio

Peer Review File

Systematic production of human kidney organoids for transplantation in porcine kidneys during ex vivo machine perfusion

Corresponding Author: Prof Nuria Montserrat

A version of this paper was originally rejected for publication by Nature Biomedical Engineering, however that decision was reconsidered after appeal by the authors.

Version 0:

Decision Letter:

Dear Prof Montserrat,

Thank you for submitting to *Nature Biomedical Engineering* your manuscript, "Systematic production of human kidney organoids to empower differentiation and transplantation in porcine kidneys during ex vivo perfusion". I regret that we are unable to consider it for external peer review.

As you may know, we screen manuscripts against our editorial criteria, and decline a substantial proportion of them without input from external referees (pertinent journal-level statistics are available). In such cases, even if reviewers were to certify the manuscript as technically correct, we feel that it would not be of outstanding interest to merit publication in *Nature Biomedical Engineering*. These editorial judgements are based on considerations of the degree of advance, broad implications, and breadth and depth of the work reported in the manuscript.

The topic of the manuscript is within the remit of the journal. However, in view of your own published work (being the most relevant ref. 2), we feel that optimization of seeding conditions, improving of yield of human kidney organoid and the development of a lineage tracing strategy do not reach the high threshold in degree of advances we look for in manuscripts that we consider for external peer review. Shall you provide evidence of the applicability of your organoids in translationally relevant models (as it appears to be in line with current ongoing efforts in your lab), I would be happy to reassess your study.

We appreciate that rejections are unwelcome. To make them easier to understand, we wrote about how we decline manuscripts.

Best wishes,

Valeria

__
Dr Valeria Caprettini
Associate Editor, *Nature Biomedical Engineering*

* The manuscript may be appropriate for another journal in the Nature Portfolio. If you wish to explore the journals and transfer your manuscript, please use our manuscript transfer portal. You will not have to re-supply manuscript metadata and files. This link can only be used once and remains active until used. For more information, please see our manuscript transfer FAQ page.

Note that any decision to opt in to *In Review* at the original journal is not sent to the receiving journal on transfer. You can opt in to *In Review* at receiving journals that support this service by choosing to modify your manuscript on transfer. *In Review* is available for primary research

manuscript types only.

Version 1:

Decision Letter:

Dear Prof Montserrat,

Thank you again for submitting to *Nature Biomedical Engineering* your manuscript, "Systematic production of human kidney organoids to empower differentiation for transplantation in porcine kidneys during ex vivo machine perfusion" and please accept our sincere apologies for the time taken in reaching a decision, which is related to the team being short of staff, attendance to pre-organised conferences together with an extremely high workload. The manuscript has been seen by 2 experts, whose reports you will find at the end of this message. I was trying really hard to secure an additional expert but I wasn't lucky, and I decided to move forward with these two reports.

You will see that the reviewers appreciate the work. However, they express concerns about the degree of support for the claims, and provide useful suggestions for improvement. We hope that with substantial further work you can address the criticisms and convince the reviewers of the merits of the study.
In particular, we would expect that a revised version of the manuscript provides:

* Description of the elicited immune response and discussion about the potential implications of it

* Thorough methodological reporting, with emphasis on the protocol chosen for obtaining the infused organoids

* Additional controls as per the reviewers' comments

When you are ready to resubmit your manuscript, please upload the revised files, a point-by-point rebuttal to the comments from all reviewers, the reporting summary, and a cover letter that explains the main improvements included in the revision and responds to any points highlighted in this decision.

Please follow the following recommendations:

* Clearly highlight any amendments to the text and figures to help the reviewers and editors find and understand the changes (yet keep in mind that excessive marking can hinder readability).

* If you and your co-authors disagree with a criticism, provide the arguments to the reviewer (optionally, indicate the relevant points in the cover letter).

* If a criticism or suggestion is not addressed, please indicate so in the rebuttal to the reviewer comments and explain the reason(s).

* Consider including responses to any criticisms raised by more than one reviewer at the beginning of the rebuttal, in a section addressed to all reviewers.

* The rebuttal should include the reviewer comments in point-by-point format (please note that we provide all reviewers will the reports as they appear at the end of this message).

* Provide the rebuttal to the reviewer comments and the cover letter as separate files.

We hope that you will be able to resubmit the manuscript within <u>20 weeks</u> from the receipt of this message. If this is the case, you will be protected against potential scooping. Otherwise, we will be happy to consider a revised manuscript as long as the significance of the work is not compromised by work published elsewhere or accepted for publication at *Nature Biomedical Engineering*.

We hope that you will find the referee reports helpful when revising the work. Please do not hesitate to contact me should you have any questions.

Best wishes,

Valeria

___
Dr Valeria Caprettini
Associate Editor, *Nature Biomedical Engineering*

____
Reviewer #1 (Report for the authors (Required)):

The study is exceptionally interesting and innovative. The description of the organoid generation is very impressive and demonstrates the exceptional experience of the CIBER-BBN group. The authors are to be congratulated to advance the implementation of organoid-based regeneration into a porcine kidney perfusion model.
I have several suggestions:

1) The authors could better describe the concept of introducing organoids into the kidney through to advance and scale production and/or test the integration and function of such organoids. One would expect embolization and necrosis of the respective functional unit through the infusion of organoids in an otherwise healthy kidney. The cited work from Sampaziotis introduced the organoids into the biliary tree and blocked the respective site for a period in order to enable engraftment. How are the organoids expected to regenerate kidney tissue in the real world application?
2) The transplantation of the kidneys carrying humanoid organoids represent xenografts. The authors should describe the immune response. Was the immune response the reason to choose a 48 hours observation period?
3) It would be great to advance the testing of hPSC-derived kidney organoids tested in a discarded human kidney model.
4) The number of xenolight positive cells is decreasing after transplantation. Was is the supposed mechanism for the loss?
5) It is difficult to find the number and conditions of the experiments. This would be helpful to estimate experiments -related variations.
6) The conclusion that „This work paves the way to the 45 utilization of hPSC-kidney organoids for ex vivo cell-based therapies to improve graft function before transplantation" is overstating the content of the study.
7) I am not qualified to assess the methodology of organoid generation.

Reviewer #2 (Report for the authors (Required)):

The authors have extensively evaluated the effect of cell density of posterior intermediate mesoderm (PIM)-committed cells on kidney organoid composition using various techniques including, but not limited to, immunocytochemistry, labeling of WT1 precursors and single cell mRNA sequencing. They then infuse a population of organoids using normothermic machine perfusion of porcine kidneys and autotransplant the perfused kidneys into the donor pig. They find that they can detect human organoid cells in the pig kidney. They confirm that there is little effect on a number of physiological parameters. There are, however, a number of issues with the manuscript.

1.The data are interesting in making the point that the density of PIM-committed cells upon transition from one to two-dimensional culture determines the number of nephron segments and metabolic characteristics of the developing organoid. It seems, however, that there is not a very good connection between these data and the intraarterial organoid infusion to the pig. It is not clear how they decided which protocol to use to obtain organoids (different densities of PIM-committed cells) on infusion and why they chose a normothermic perfusion temperature. There is no indication of any function of the organoids that have been infused, and no information about vascularization etc, so it is difficult to see how important the pig data are beyond the fact that all the organoid cells do not die after injection. We do not have an indication of what percentage of the cells survive the transplantation. Recognizing that one cannot count all the cells in the transplanted organ perhaps there can be some estimate of survival range by sampling regions. A more interesting comparison in terms of organ regeneration would be to induce injury/kidney disease and evaluate whether organoids or dissociated cells into the host animal had any effect on kidney function.

2.It is unclear whether the authors utilized the micro-organoids produced by their protocols for their downstream transplant studies. Authors should conclusively state whether or not their micro-organoids were used in any pig studies. (Authors do briefly state micro-well organoids were used in the figure legend of supp. Fig 7… But not in the Results or any main body figure legends).

3.Much of the key data in the manuscript are in the Supplemental figures. It is not clear which this distribution of Figures (main manuscript/supplement) was chosen by author.

4.A major conclusion of the work is that the density of PIM-committed cells is key to differentiation and metabolism with the higher densities resulting in fewer proximal tubules and less oxidative metabolism. If one looks at the various panels in Supplemental figures 1 it is clear that the more PIM-committed cells that are combined the bigger the organoid is. The changes in outcome may relate to the fact that there is more hypoxia in the center of the larger organoids and this would be expected to drive glycolytic metabolism and fewer segments such as proximal tubule segments that were dependent upon oxidative phosphorylation. There are no controls for cell size.

5.Fig. 1: The authors come to the conclusion that "promoting cell-cell contact induces OXPHOS, which drives PT differentiation." but they do not present data on cell-cell contact to be able to reach that conclusion? They infer from the fact that higher cell density organoids have less differentiation… However, the larger organoids could have just as much cell-cell contact as the smaller organoids?

6.Many published organoid protocols employ 3D aggregation to stimulate differentiation. What appears to be novel is their

modified protocol and techniques to mass produce micro-organoids. This protocol can be scaled up and employed for organoid production for use in disease modeling or prior to engraftment into kidneys or scaffolds to produce renal replacement options. The authors, however, do not comment on the scale-up potential.

7.Line 85: The authors start with hPSC-PIM-committed cell monolayers but they do not indicate what they did to commit the cells to PIM? It would be important for the authors to state how the hPSCs were maintained in culture and how many cells were placed in monolayer. The authors should talk about the monolayer differentiation phase so that the Results section can be more clearly related to Figure 1a and b.

8.Line 133 and the associated paragraph: The authors comment on the increased levels of metallothioneins in tubular-like cells of kidney organoids derived from 0.5-PIM compared to 250-Ks and in podocyte-like cells. It is not stated what the significance of this is?

9.Suppl figure 1, panel c : The authors state that there is staining with ureteric bud markers without stating which of the markers they are referring to. GATA3 is a ureteric bud marker but does not indicate ureteric bud derivation. It is expressed in the mesangial cell and distal tubules of healthy human kidneys (Grigorieva et al JASN 2019) which are not derived from ureteric bud. Do the authors have indication that there are any anterior intermediate mesoderm markers along with PIM during organoid development using their protocols?

10.Suppl Figure 1, panels e and f. It appears that nephrin is highly expressed at day 16 in 0.5k and 8k conditions. This is not reflected in the quantitation in panel c. The authors should add quantitation on nephrin. In panel f, it is not possible to evaluate the grey PODXL staining.

11.Suppl Figure 2. Cannot distinguish the LTL or PODXL depicted in grey.

12.Suppl Figure 3a. I am surprised to see little variation in expression of proximal tubule genes for the 4 conditions. This implies that the genes are either expressed or not without any influence of the cell density at the start. Clearly there are a number of proximal tubule genes that are not expressed in any of the 4 conditions. If this is related to their stage of differentiation then it suggests that the 4 densities do not influence differentiation state of the proximal tubules but rather only the number of them.

13.Suppl Figure 3d. Not sure what can conclude from GATA3 staining other than there is no difference?

14.Figure 2f,h and Suppl Figure 7d and f. Fractional excretion of Na and creatinine should be a %. Not clear how to interpret these plots.

15.Suppl Fig 7p & q would seem to be important data for the main body figures since they data highlight renal function before and after transplantation of kidneys perfused with organoids. However, only 2 animals were used. One experiment ended at 24h and one ended at 48h, with urine and blood collections at various timepoints. Why were both animals not monitored for at least 48h? The values of the 2 animals seem similar. The biochemical analyses performed on these healthy animals prior to surgery and post-transplant doesn’t clarify the effects of organoid engraftment in the transplanted kidney. Why is the animal blood urea increased 4-fold within 18 hr of transplantation? Why does serum creatinine increase by 50% with the transplant? Would be good to have data comparing kidneys+organoids vs kidneys-organoids post-transplant?

16.Figure 2k. It appears that most of the Alu repeats in the porcine cortex are in glomerular regions which is predictable since the glomerular capillaries would trap them. What were the size of the organoids infused?

17.Lines 231-235 discuss the studies demonstrating vascularization of organoids by host tissues, and how they aimed to “challenge our differentiation pipeline for therapeutic translation in kidney regeneration”; however, the authors provide no data to assess kidney regeneration or vascularization in the transplantation experiments?
Minor
18. Suppl Figure 5f. Do not see the 100 m scale bars

19. line 203: “In” rather than “Into”

20. Suppl Figure 5h,k. It states in the figure legend: “Scale bars (l, m), 200 μm”. I believe the authors mean panels (h,k).

Version 2:

Decision Letter:

Dear Prof Montserrat,

Thank you for your revised manuscript, "Systematic production of human kidney organoids for transplantation in porcine kidneys during ex vivo machine perfusion", which has been seen by the original reviewers. In their reports, which you will find at the end of this message, you will see that the reviewers acknowledge the improvements to the work and raise a few additional technical criticisms that we hope you will be able to address. In particular, we would expect that the next version of the manuscript provides additional discussion of the limitations of the work, and a clearer explanation of how hPSC-derived organoids could be incorporated within a kidney graft intervention.

As before, when you are ready to resubmit your manuscript, please upload the revised files, a point-by-point rebuttal to the comments from all reviewers, the reporting summary, and a cover letter that explains the main improvements included in the revision and responds to any points highlighted in this decision.

As a reminder, please follow the following recommendations:

* Clearly highlight any amendments to the text and figures to help the reviewers and editors find and understand the changes (yet keep in mind that excessive marking can hinder readability).

* If you and your co-authors disagree with a criticism, provide the arguments to the reviewer (optionally, indicate the relevant points in the cover letter).

* If a criticism or suggestion is not addressed, please indicate so in the rebuttal to the reviewer comments and explain the reason(s).

* Consider including responses to any criticisms raised by more than one reviewer at the beginning of the rebuttal, in a section addressed to all reviewers.

* The rebuttal should include the reviewer comments in point-by-point format (please note that we provide all reviewers will the reports as they appear at the end of this message).

* Provide the rebuttal to the reviewer comments and the cover letter as separate files.

We expect that you will be able to resubmit the manuscript within 12 weeks of receiving this message. If this is the case, you will be protected against potential scooping. Otherwise, we will be happy to consider a revised manuscript as long as the significance of the work is not compromised by work published elsewhere or accepted for publication at *Nature Biomedical Engineering*.

We look forward to receive a further revised version of the work. Please do not hesitate to contact me should you have any questions.

Best wishes,

Valeria

__
Dr Valeria Caprettini
Senior Editor, *Nature Biomedical Engineering*

____
Reviewer #1 (Report for the authors (Required)):

Thanks to the authors for providing such comprehensive additional information in response to the suggestions provided in the first review. All in all, both the ambitious response and the extensive efforts to add more supporting information and substance to the study demonstrate the capability of the group of authors.

One aspect that I suggest to clarify is the hypothesis of the organoid NMP/infusion experiments. It remains unclear, how "hPSC-derived organoids as an intervention for
graft pre-treatment" should work. Is the idea to produce kidneys within kidneys? To add functional units within kidneys? How would the organoids integrate and add to the function of kidneys? Shall they extend an repair mechanism? Or - as xenografts in pig kidneys - modify the immune response?

Further to this, it should be emphasized in the limitations of the study, that because the contralateral kidney was not removed in the transplant experiments, any functional analysis in the transplant model are limited because the native kidney remains intact and functioning.
Despite this fact, the dynamics of blood and urine analysis are surprising and difficult to understand. The display of each

experiment individually instead of grouped outcomes, makes it hard to read and follow. Could you please clarify why you believe there is an increase in e.g. urea and creatinine. Also, data for the 24 hour experiment are shown for the 18 hour time point and not 24 hours.

Reviewer #2 (Report for the authors (Required)):

The authors have responded to each of my concerns. The effects on the functional aspects of the transplanted kidney related to organoid infusion remain limited.

Version 3:

Decision Letter:

Dear Prof Montserrat,

Thank you for your revised manuscript, "Systematic production of human kidney organoids for transplantation in porcine kidneys during ex vivo machine perfusion", which has been seen by Reviewers #1. In their reports, which you will find at the end of this message, you will see that the reviewers acknowledge the improvements to the work and raise a few additional technical criticisms that we hope you will be able to address.

As before, when you are ready to resubmit your manuscript, please upload the revised files, a point-by-point rebuttal to the comments from all reviewers, the reporting summary, and a cover letter that explains the main improvements included in the revision and responds to any points highlighted in this decision.

As a reminder, please follow the following recommendations:

* Clearly highlight any amendments to the text and figures to help the reviewers and editors find and understand the changes (yet keep in mind that excessive marking can hinder readability).

* If you and your co-authors disagree with a criticism, provide the arguments to the reviewer (optionally, indicate the relevant points in the cover letter).

* If a criticism or suggestion is not addressed, please indicate so in the rebuttal to the reviewer comments and explain the reason(s).

* Consider including responses to any criticisms raised by more than one reviewer at the beginning of the rebuttal, in a section addressed to all reviewers.

* The rebuttal should include the reviewer comments in point-by-point format (please note that we provide all reviewers will the reports as they appear at the end of this message).

* Provide the rebuttal to the reviewer comments and the cover letter as separate files.

We expect that you will be able to resubmit the manuscript within 8 weeks of receiving this message. If this is the case, you will be protected against potential scooping. Otherwise, we will be happy to consider a revised manuscript as long as the significance of the work is not compromised by work published elsewhere or accepted for publication at *Nature Biomedical Engineering*.

We look forward to receive a further revised version of the work. Please do not hesitate to contact me should you have any questions.

Best wishes,

Valeria

__
Dr Valeria Caprettini
Senior Editor, *Nature Biomedical Engineering*

Reviewer #1 (Report for the authors (Required)):

Thank again for the meaningful edits to the manuscript. The authors clearly put a lot of efforts into further improving the study.

Some minor remarks:

• In the revised version of the abstract, the authors stat that the organoid transfer into kidneys is done “with the aim to improve kidney function during organ failure”. This is a legit aim, but none of the data supports this claim. I would suggest to remove this part of the sentence and any such speculations throughout the manuscript.

• In the chapter demonstrating organoid engraftment, the authors mention that they are using slaughterhouse pigs for these experiments. In the next chapter, they describe that they are reimplanting the kidneys into the donor animals. I assume this is a different model. Please clarify.

• Terms such as “in vivo engrafted kidneys” is confusing. Maybe use retransplanted or transplanted instead.

• The authors state that “These results (of the transplant experiments) suggest that graft viability was mostly preserved during the study period evaluated here, overall showing the feasibility and safety of our approach.” I agree with safety and feasibility with some reservation. SFig. 9. Fig. 9 does not show a control, so strong conclusions are difficult. Also, the second, native kidney seems to be in place, but urea, crea and tissue injury markers increase during the early phase. What is shown is that despite of the interventions, the kidneys look macroscopically viable. However, a histological readout is missing. I would suggest to add a histological readout showing and grading the degree of damage to support the conclusion

• Rebuttal letter Fig 1a displays the results of 1 kidney transplanted without organoids. In this procedure, the contralateral kidney was removed. In the intervention group it was

Version 4:

Decision Letter:

Dear Prof Montserrat,

Thank you for your revised manuscript, "Systematic production of human kidney organoids for transplantation in porcine kidneys during ex vivo machine perfusion". Having consulted with Reviewers #1 (whose comment you will find at the end of this message), I am pleased to write that we shall be happy to publish the manuscript in *Nature Biomedical Engineering*.

We will be performing detailed checks on your manuscript, and in due course will send you a checklist detailing our editorial and formatting requirements. You will need to follow these instructions before you upload the final manuscript files.

Please do not hesitate to contact me if you have any questions.

Best wishes,

Valeria

__
Dr Valeria Caprettini
Senior Editor, *Nature Biomedical Engineering*

____
Reviewer #1 (Report for the authors (Required)):

No further objections. Thanks for the efforts you put into the revisions. Great work.

Version 5:

Decision Letter:

Dear Prof Montserrat,

I am happy to inform you that your manuscript, "Systematic production of human kidney organoids for transplantation in porcine kidneys during ex vivo machine perfusion", has now been accepted for publication in *Nature Biomedical Engineering*.

Over the next few weeks, the figures will be checked for production quality, the text edited to ensure that it conforms to house style, and the manuscript typeset.

Our Articles are published about 40 days after the acceptance date (we recommend that you inform your institutional press office of this timeframe), and you will be notified of the actual publication date a few days in advance. Articles can be published any working day of the week, and are pushed live shortly after 10 am London time.

Publishing agreement. You will be asked to digitally sign a publishing agreement (grant of rights). After the signed publishing agreement has been received, the proofs of the article will be sent to you for review. If you have any queries during the production process, or you cannot meet the requested deadline for returning the proofs, please contact rjsproduction@springernature.com.

Nature Biomedical Engineering is a Transformative Journal. Authors may publish their research with us through the traditional subscription access route, or make their paper immediately open access through payment of an article-processing charge. More information about publication options is available.

You may need to take specific actions to comply with funder and institutional open-access mandates. If the work described in the accepted manuscript is supported by a funder that requires immediate open access (as outlined, for example, by Plan S) and your manuscript was originally submitted on or after January 1st 2021, then you should select the gold OA route. Authors selecting subscription publication will need to accept our standard licensing terms (including our self-archiving policies), and these will supersede any other terms that the author or any third party may assert apply to any version of the manuscript.

Acceptance of your manuscript is conditional on agreement, by all authors, with both our media embargo and confidentiality and pre-publicity policies. In particular, you may arrange your own publicity of the Article (for instance, through your institutional press office), as long as you ensure that journalists strictly adhere to the media embargo.

To assist you in disseminating the work, as soon as the Article is published you will be able to take advantage of the Springer Nature SharedIt initiative to generate a unique shareable link to the Article that will allow anyone (with or without a subscription) to read it. Recipients of the link who are subscribers will also be able to download and print the PDF.

Thank you for having submitted this work to *Nature Biomedical Engineering*.

Best wishes,

Valeria

___
Dr Valeria Caprettini
Senior Editor, *Nature Biomedical Engineering*

Open Access This Peer Review File is licensed under a Creative Commons Attribution 4.0 International License, which permits use, sharing, adaptation, distribution and reproduction in any medium or format, as long as you give appropriate credit to the original author(s) and the source, provide a link to the Creative Commons license, and indicate if changes were made.
In cases where reviewers are anonymous, credit should be given to 'Anonymous Referee' and the source.
The images or other third party material in this Peer Review File are included in the article's Creative Commons license, unless indicated otherwise in a credit line to the material. If material is not included in the article's Creative Commons license and your intended use is not permitted by statutory regulation or exceeds the permitted use, you will need to obtain permission directly from the copyright holder.
To view a copy of this license, visit https://creativecommons.org/licenses/by/4.0/

REBUTTAL LETTER TO REVIEWERS

Reviewer (R) #1:

The study is exceptionally interesting and innovative. The description of the organoid generation is very impressive and demonstrates the exceptional experience of the CIBER-BBN group. The authors are to be congratulated to advance the implementation of organoid-based regeneration into a porcine kidney perfusion model.

I have several suggestions:

R#1: 1) *The authors could better describe the concept of introducing organoids into the kidney through to advance and scale production and/or test the integration and function of such organoids. One would expect embolization and necrosis of the respective functional unit through the infusion of organoids in an otherwise healthy kidney.*
The cited work from Sampaziotis introduced the organoids into the biliary tree and blocked the respective site for a period in order to enable engraftment. How are the organoids expected to regenerate kidney tissue in the real world application?

Authors (A): We appreciate the reviewer's invaluable feedback and the very positive evaluation of our work. We divide here the comments of the expert and answer to them:

R#1: 1.1) *The authors could better describe the concept of introducing organoids into the kidney through to advance and scale production and/or test the integration and function of such organoids.*

A: We appreciate the reviewer's invaluable feedback.
In the revised version of our work, we now carefully describe the concept of the introduction of organoids through the kidney renal artery in the corresponding section (Methods). Further details on the description of the novelty of our approach to scale up kidney organoids from hPSCs has been also included in the corresponding section (Methods) and by adding this information in the main text and in the new **Figure 2 of the manuscript** which details the generation WT1 reporter cell line to establish the methodology for organoid generation in a scalable model (***Figure 1 a-j*** **in this rebuttal letter** and **new Figure 2 a-j of the manuscript**). We also include the results about the validation of the approach in another cell line (***Figure 1 k-m*** **in this rebuttal letter** and **new Figure 1 k-m of the manuscript**).

>Figure redacted<

<u>Figure 1</u>. Production of uniform kidney organoids by micro-aggregation. ***a***, *Schematic representation of the WT1 gene locus where sgRNAs were designed. 4 sgRNAs were targeted to sequences around the WT1 stop codon at exon 10 and screened for editing efficiency by MiSeq.* ***b***, *Quantification of editing efficiencies for each sgRNA is represented as a distribution of reads carrying Indels (% blue) or unmodified reads (% gray). The sgRNA leading to highest efficiency of genome editing is highlighted in red.* ***c***, *Representative bright field image of WT1-GFP reporter hPSC line. Scale bar, 100 µm.* ***d,*** *mRNA expression levels of SOX2, OCT4, NANOG, DNMT3B, CRIPTO and REX1 in WT1-GFP reporter hPSC line (green) compared to wild-type hPSC line (black). Data are mean ± s.d. (technical replicates).* ***e***, *Representative immunofluorescence staining for GFP (green), NANOG (magenta) and DAPI (blue) in undifferentiated WT1-GFP reporter hPSC colonies. Scale bar, 200 µm.* ***f,*** *Experimental scheme for the generation of uniform kidney organoids from the WT1-GFP reporter hPSC line using the microwell culture system.* ***g,*** *Representative bright field and fluorescent images of kidney organoids derived from the WT1-GFP reporter hPSC line by the microwell culture system during the time course of differentiation (days are indicated).*

h*,*i *Representative immunofluorescence staining in day 16 kidney organoids derived the WT1-GFP reporter hPSC line using the microwell culture system for the detection of GFP (green), NEPHRIN (red), LAMININ (grays) and DAPI (blue) in (c), and GFP (green), CD31 (red), LTL (grays) and DAPI (blue) in (d). Scale bars (c,d), 200 µm.* ***j,*** *mRNA expression levels of WT1, PODXL, MAFB, PDGFRA, SLC3A1 and PGC1A, during the time course of kidney organoid differentiation in 0.5-Ks organoids generated by the microwell culture system. Data are mean ± s.d. (technical replicates). Each sample is a pool of 1200 organoids with two technical replicates each.* ***k,*** *Representative bright field images of kidney organoids derived from hESC by the microwell culture system on day 2, 7 and 16 of differentiation. Scale bars, 100 µm.* ***l,*** *Representative immunofluorescence staining of day 16 kidney organoids derived from hESC using the microwell culture system for the detection of LAMININ (green), NEPHRIN (red), LTL (grays) and DAPI (blue), and ECAD (green), CD31 (red), PODXL (grays) and DAPI (blue). Scale bars, 200 µm.* ***m,*** *mRNA expression levels of renal differentiation markers during the time course of kidney organoid differentiation in 0.5-Ks organoids generated by the microwell culture system. Data are mean ± s.d. (technical replicates). Each sample is a pool of 1200 organoids with two technical replicates each. organoids with two technical replicates each.* ***i,*** *Representative bright field images of kidney organoids derived from hiPSC by the microwell culture system on day 2, 7 and 16 of differentiation. Scale bars, 100 µm.*

To provide further experimental evidence about *the function of such organoids* (**R#1. 2**) we have conducted very preliminary experiments described **in this rebuttal letter**. Specifically, we have exposed kidney organoids in front of a very well-known nephrotoxic agent called Cisplatin for 24h as an assay to assess potential responses of kidney organoid tubular-like cells (demarked with *Lotus Tetraglobus Lectin*-LTL) through the detection of kidney-specific injury molecule KIM1 in comparison to control counterparts (without cisplatin) (***Figure 2*** **in this rebuttal letter**). Furthermore, we have assessed the impact of cisplatin treatment by RT-qPCR. Our results suggest that 0.5 Ks kidney organoids respond to cisplatin and open the door for the realization of other assays in vitro. For the present time we have not explored more functional assays as we believe this is out of the scope of the manuscript. We would like to acknowledge the expert comment, and we will work on the realization of these new experiments for the realization of other manuscript focused on the characterization of our new differentiation pipeline.

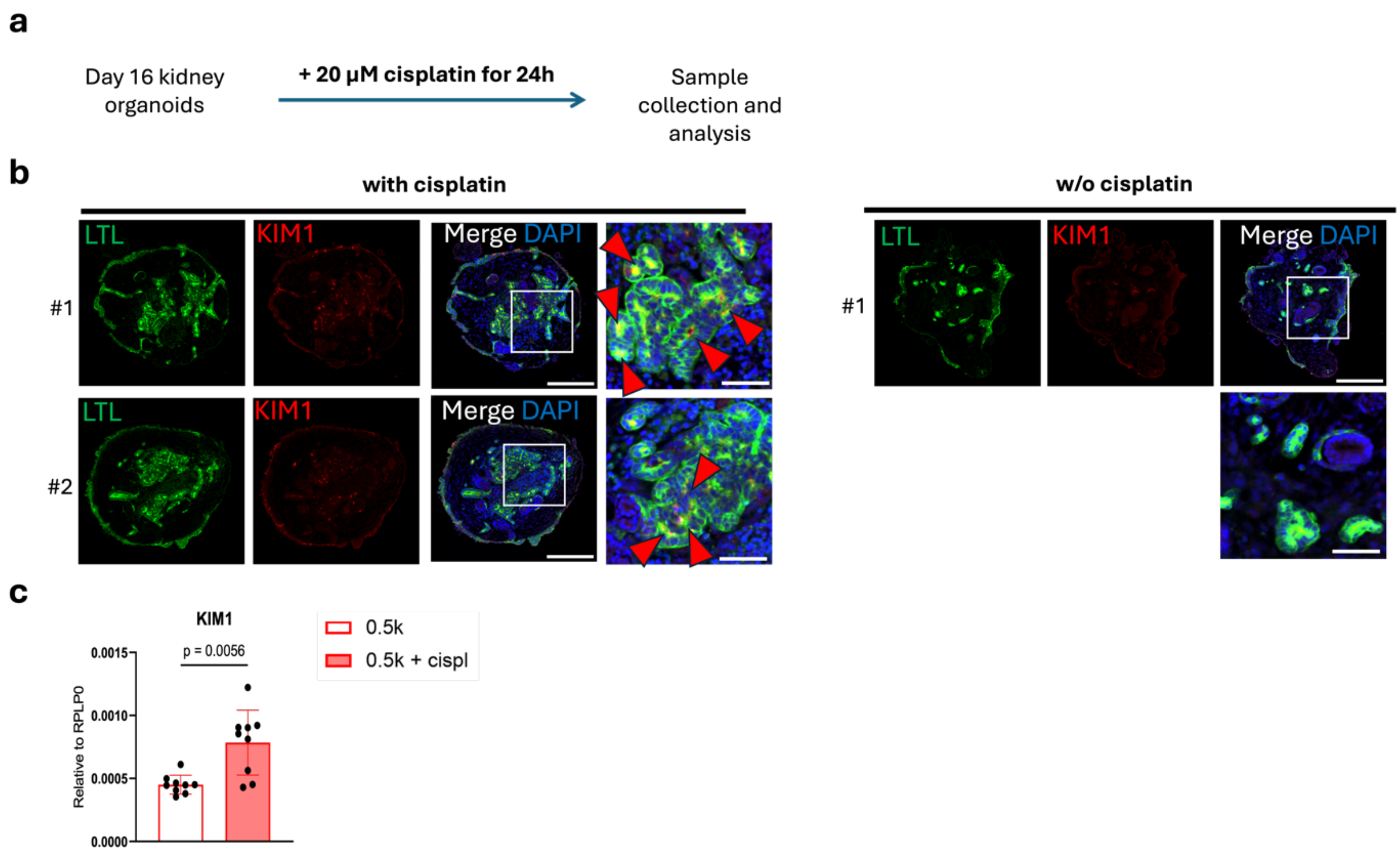

***Figure 2**. **a)** Day 16 kidney organoids were treated with 20 micromolar cisplatin for 24h after which samples were collected for immunofluorescence and qPCR analysis. **b)** Immunofluorescence analysis for the detection of KIM1 (red) and LTL (green) staining and DAPI (blue) in kidney organoids after 24h treatment with the nephrotoxicant Cisplatin. Red arrows highlight double positive cells for LTL and KIM-1 detection. Scale bars, 200 microns, 50 microns (Magnified views). **c**) mRNA expression levels of KIM-1 in the absence or presence of cisplatin during 24 h in 0.5-Ks. Data are mean ± s.d. n= 3 biological samples per group. Each sample is a pool of 12 organoids/group with three technical replicates each.*

To provide feedback to the expert on evidences of *integration* we have conducted an analysis for the detection of ECAD and LTL positive cells, as well as for the tubule injury marker KIM-1, in three biological experiments corresponding to kidney biopsies of specimens undergoing organoid infusion during NMP (**Supplementary Fig. 7n in the manuscript**) and further autotransplantation for 24 and 48 hours (**Figure 3g,h in the manuscript**). Immunofluorescence analyses were conducted in consecutive paraffin sections showing positivity for Alu. In this manner, we aim to show the integration of human cells within the porcine kidney tissues. Part of these new results are shown also here in ***Figure 3*** **in this rebuttal letter.**

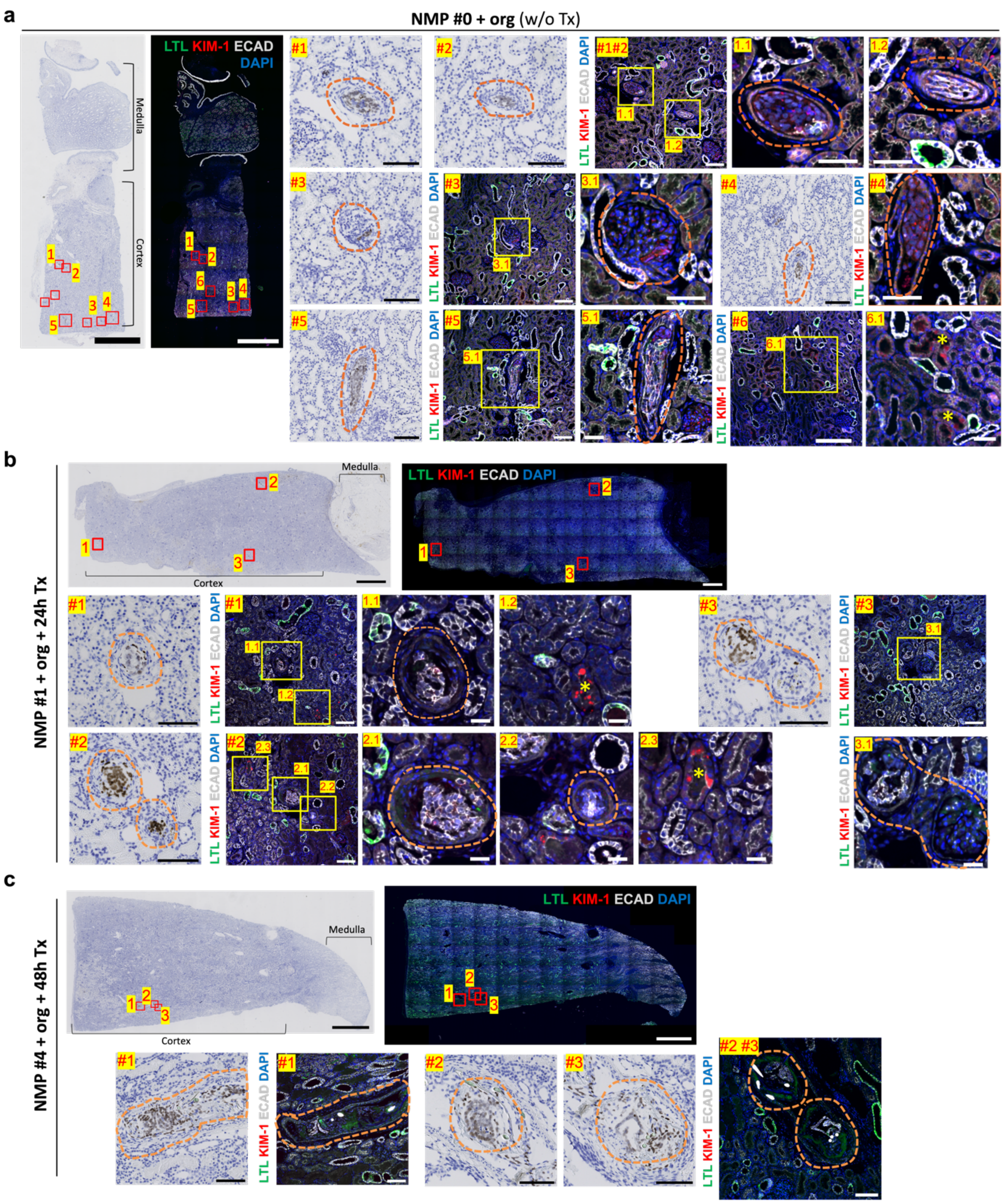

Figure 3. *Detection of E-Cadherin (ECAD; white), Lotus Tetraglobus Lectin (LTL; green) and KIM-1 (red) in consecutive paraffin sections positive for the detection of Alu repeat sequences of human DNA (red boxes highlighted with dashed numbers). Images correspond to kidneys after infusion of intact kidney organoids during ex vivo NMP (**a**) and autotransplantation during 24 h (**b**) and 48 h (**c**), respectively. Scale bars, 2 mm, 100 µm (magnified views), 50 µm (closer magnified views). Dashed orange lines indicate areas containing cells of human origin. Yellow asterisks indicate KIM-1 positive tubuli.*

With regards to this last comment, we would like to add that experiments to test integration would need a different experimental set up to the one conducted in our manuscript. For example, and as highlighted by the expert in her/his remark ***R#1.3***, kidney organoids may be applied by fluoroscopic

guidance and further block the respective site of application may be done to assess function ex vivo during NMP. This approach was the one used by Sampiazotis and co-workers who administer enzymatically dissociated cells from gallbladder organoids and applied NMP during 100 hours to further assess cell engraftment. Besides the appropriateness of this approach to answer the expert comment we have conducted the series of analysis highlighted in the previous paragraph which now represent new data for the manuscript. We hope that the new results will be of interest for the expert and we would like to thank her/his comment to improve our work.

R#1: 1.2) *One would expect embolization and necrosis of the respective functional unit through the infusion of organoids in an otherwise healthy kidney.*

A: We thank the expert comment and acknowledge that because NMP is a promising technique, evaluation of injury during perfusion is needed, especially in the context of different NMP protocols that are currently used, which might affect this injury. To date, there is no consensus on rather basic aspects of machine perfusion for kidneys such as perfusate composition or pressure settings. Similarly, recent studies have evaluated the amount of endothelial injury during NMP in porcine kidneys (PMID: 37456589) and whether different pressures and hematocrits affect this injury (PMID: 33979315; PMID: 24811916; PMID: 16673356). Interestingly, previous studies have compared structural cell injury in isolated porcine kidneys during NMP for seven hours with autologous, allogeneic porcine, or human red blood cells (RBC) observing glomerular and tubular damage in all experimental groups with signs of pathological hyperfiltration and microvascular injury in the human RBC group (PMID: 32155167).

Here, in response to the expert comments, we have performed a qualitative evaluation of damage in our experimental set up through hematoxylin eosin staining and pathology assessment (***Table 1*** **in this rebuttal letter**). The biopsies were examined, and a damage scoring system was developed, based on previously reported scoring systems (PMID: 30951018) and validated by an experienced renal pathologist (University of Leuven; participating as co-authors in the revised manuscript). The sections were scored on a scale of 0–3 for the severity of acute tubular injury (ATI), tubular necrosis, tubulitis, interstitial inflammation, Glomerulitis (g), Peritubular capillaritis (ptc), Endarteritis (v), interstitial fibrosis and tubular atrophy (IFTA).

Specimen	ATI	Severity of ATI	Tubular necrosis	Tubulitis	Interstitial inflammation	Type of inflammatory cells	Glomerulitis	Peritubular capillaritis	Endarteritis	IFTA
MOCK CTL 4h U_5_HE	1	1	0	0	0	mononuclear cell infiltrates	0	0	0	5%
CTL#0 U 20 - 2023-12-04	1	1	0	0	0	mononuclear cell infiltrates	0	0	0	0
CTL#0 U_5_HE - 2024-05-04	1	1	0	0	0	nil	0	0	0	0
CTL#1 U 20 - 2023-12-04	1	1	0	0	0	nil	0	0	0	0
CTL#1 U_5_HE - 2024-05-04	1	1	0	0	1	mononuclear cell infiltrates	0	0	0	0
CTL#2 C(1)_10_HE - 2024-06-20	1	2	0	0	0	nil	0	0	0	0
CTL#3 C(1)_10_HE - 2024-06-20	1	1	0	0	0	nil	0	0	0	0
CTL#4 U 20 - 2023-12-04	1	2	0	0	0	nil	0	0	0	0
CTL#4 U_5_HE - 2024-05-04	1	1	0	0	0	nil	0	0	0	0
CTL#5 C (1)_HE - 2024-05-17	1	1	0	0	0	nil	0	0	0	0
CTL#5 C (2)_HE - 2024-05-17	1	1	0	0	0	mononuclear cell infiltrates	0	0	0	0
CTL#5 L (1)_HE - 2024-05-17	1	1	0	0	0	nil	0	0	0	0
CTL#5 L (2)_HE - 2024-05-17	1	1	0	0	0	nil	0	0	0	0
CTL#5 U (1)_HE - 2024-05-17	1	1	0	0	0	nil	0	0	0	0
CTL#5 U (2)_HE - 2024-05-17	1	1	0	0	0	mononuclear cell infiltrates	0	0	0	0
CTL#6 C (1)_HE - 2024-05-17	1	1	0	0	0	nil	0	0	0	0
CTL#6 C (2)_HE - 2024-05-17	1	1	0	0	0	nil	0	0	0	0
CTL#6 L (1)_HE - 2024-05-17	1	1	0	0	0	nil	0	0	0	0
CTL#6 L (2)_HE - 2024-05-17	1	1	0	0	0	nil	0	0	0	0
CTL#6 U (1)_HE - 2024-05-17	1	1	0	0	0	nil	0	0	0	0
CTL#6 U (2)_HE - 2024-05-17	1	1	0	0	0	nil	0	0	0	0
MOCK +24 H tx U_5_HE	1	3	0	0	0	mononuclear cell infiltrates	0	0	0	0
NMP#0+org C 20 - 2023-12-04	1	1	0	0	0	nil	0	0	0	0
NMP#0+org L 5 - 2023-12-04	1	2	0	0	1	mononuclear cell infiltrates	0	0	0	0
NMP#0+org L 20 - 2023-12-04	1	2	0	0	0	mononuclear cell infiltrates	0	0	0	0
NMP#0+org U 20 - 2023-12-04	1	1	0	0	0	nil	0	0	?1	0
NMP#0+org U_5_HE - 2024-05-04	1	1	0	0	0	nil	0	0	0	0
NMP#1+org+24h Tx C 5 - 2023-12-04	1	2	1	0	1	mononuclear cell infiltrates	0	0	0	0
NMP#1+org+24h Tx C 20 -2023-12-04	1	2	1	0	1	mononuclear cell infiltrates	0	0	0	0
NMP#1+org+24h Tx L 5 - 2023-12-04	1	3	1	0	1	mixed - L, P, N	0	0	0	0
NMP#1+org+24h Tx L 20 - 2023-12-04	1	3	1	0	1	mixed - L, P, N	0	0	0	0
NMP#1+org+24h Tx U 5 - 2023-12-05	1	2	1	0	1	mixed - L, P, N	0	0	0	0
NMP#1+org+24h TxU_5_HE - 2024-05-04	1	2	1	0	1	mixed - L, P, N	0	0	0	0
NMP#2+org+24h Tx C (2)_10_HE - 2024-06-20	1	2	0	0	0	nil	0	0	0	0
NMP#2+org+24h Tx C (1)_10_HE - 2024-06-20	1	2	0	0	0	nil	0	0	0	0
NMP#3+org+24h TxC(1)_10_HE - 2024-06-20	1	3	1	0	0	nil	0	0	0	0
NMP#3+org+24h Tx C(2)_10_HE - 2024-06-20	1	3	1	0	0	nil	0	0	0	0
NMP#4+org+48h Tx C 5 - 2023-12-05	0	0	0	0	0	nil	0	0	0	0
NMP#4+org+48h Tx L 5 - 2023-12-05	1	1	1	0	0	mononuclear cell infiltrates	0	0	0	0
NMP#4+org+48h Tx L 20 - 2023-12-05	1	1	1	0	0	mononuclear cell infiltrates	0	0	0	0
NMP#4+org+48h Tx U 5 - 2023-12-05	1	1	1	0	0	mononuclear cell infiltrates	0	0	0	0
NMP#4+org+48h Tx U 20 - 2023-12-05	1	1	1	0	0	mononuclear cell infiltrates	0	0	0	0
NMP#4+org+48h Tx U_5_HE - 2024-05-04	1	1	1	0	0	mononuclear cell infiltrates	0	0	0	0
NMP#5+org+48h Tx C (1)_HE - 20-05-17	1	3	1	0	2	Mixed - L, P, E	0	0	0	0
NMP#5+org+48h Tx C (2)_HE - 20-05-17	1	3	1	0	1	mononuclear cell infiltrates	0	0	0	0
NMP#5+org+48h Tx L (1)_HE - 2024-05-17	1	3	1	0	1	mononuclear cell infiltrates	0	0	0	0
NMP#5+org+48h Tx L (2)_HE - 2024-05-17	1	3	1	0	1	mononuclear cell infiltrates	0	0	0	0
NMP#5+org+48h Tx U (1)_HE - 2024-05-17	1	3	1	0	1	mononuclear cell infiltrates	0	0	0	0
NMP#5+org+48h Tx U (2)_HE - 2024-05-17	1	3	1	0	1	mononuclear cell infiltrates	0	0	0	0
NMP#6+org+48h Tx C (1)_HE - 2024-05-17	1	3	1	0	0	mononuclear cell infiltrates	0	0	0	0
NMP#6+org+48h Tx C (2)_HE - 2024-05-17	1	3	1	0	0	mononuclear cell infiltrates	0	0	0	0
NMP#6+org+48h Tx L (1)_HE - 2024-05-17	1	3	1	0	0	mononuclear cell infiltrates	0	0	0	0
NMP#6+org+48h TxL (2)_HE - 2024-05-17	1	3	1	0	0	mononuclear cell infiltrates	0	0	0	0
NMP#6+org+48h Tx U (1)_HE - 2024-05-17	1	3	1	0	0	mononuclear cell infiltrates	0	0	0	0
NMP#6+org+48h Tx U (2)_HE - 2024-05-17	1	3	1	0	0	mononuclear cell infiltrates	0	0	0	0

Table 1. *Qualitative evaluation of damage in all the samples of the study through hematoxylin eosin (H&E) staining and pathology assessment. Formalin fixed paraffin embedded biopsies of the upper (U), lateral (L) and central (C) renal cortex were stained with HE to assess changes in tissue morphology. The sections were scored on a scale of 0–. ATI: acute tubular injury; IFTA: interstitial fibrosis and tubular atrophy (IFTA). ATI severity was also scored on a scale of 0-3. One specimen undergoing NMP in the absence of organoids and further autotransplantation with nephrectomy during 24 h (MOCK+24 h Tx) was included in the study. CTL refers to control lateral specimens. NMP#0+org refers to one specimen undergoing NMP for 3 hours after organoid infusion. NMP#1-3+org+24h Tx refers to specimens (#1, #2 and #3) undergoing NMP for 3 hours after organoid infusion and further autotransplantation for 24h. NMP#4-6+org+24h Tx refers to specimens (#4, #5 and #6) undergoing NMP for 3 hours after organoid infusion and further autotransplantation for 48h.*

Our results show that all the analyzed samples, including control lateral kidneys, exhibit ATI. No signs of tubular necrosis, tubulitis, interstitial inflammation, Glomerulitis (g), Peritubular capillaritis (ptc), or endarteritis were found. We would like to highlight that the level of severity of ATI ranges from 1 to 3 in all the experimental conditions. Importantly, we encountered that NMP+org conditions and NMP+org+Tx conditions led to a low (score 1) to severe (score 3) extent of ATI whereas a kidney specimen undergoing NMP in the absence of organoids and Tx with nephrectomy for 24h led to a severity of ATI with score 3. This observation suggests that NMP alone, in the absence of organoids, together with the surgery procedure, already induces a certain degree of acute damage in the tubuli and that the level of damage is comparable to that found in NMP+org+Tx conditions.

In case the expert considers that this information is of interest we would be extremely in favor of adding these results in the manuscript.
For the moment we attach the table **only in this rebuttal letter** as well as a link to all the images from which this analysis was done (https://tinyurl.com/yc6pzd2m). Images can be visualized with the NDP.view2 Image viewing free software (https://www.hamamatsu.com/jp/en/product/life-science-and-medical-systems/digital-slide-scanner/U12388-01.html) or ImageJ2 (version 2.3.0) software (https://imagej.net/software/fiji/).
Similarly, a link to ***Table 1*** presented **in this rebuttal** letter can be also found here https://tinyurl.com/yc6pzd2m.

R#1: 1.3) *The cited work from Sampaziotis introduced the organoids into the biliary tree and blocked the respective site for a period in order to enable engraftment. How are the organoids expected to regenerate kidney tissue in the real world application?*

A: We agree with the expert comments and understand her/his worries on the real-world application of our approach. Soon we aim to conduct the experiments and conditions set up here in human discarded kidneys for implantation in kidney transplant candidates. The path for this real-world application is long and requires the type of experiments conducted here making use of a large preclinical animal model allowing to test parameters/conditions as we show here, briefly:
-organoid production in a scalable manner, for the first time, while guaranteeing that organoids exhibit features of differentiation and cellular composition qualitatively and quantitatively as well as indications of function (*see our response* to comment ***R#1.1***).
-single cell and organoid dosage during NMP by the realization of experiments making use of disaggregated cells from organoids and intact organoids as shown here.
-safety of the procedure of organoid infusion during NMP by qualitative and quantitative analysis, including: morphological changes in kidney tissues after NMP or after NMP+Tx, biochemical analysis in urine and plasma, possible elicited immune response systemically and on renal tissues (*see our response* to comment ***R#1.2***).

At the present time we are working in coordination with the Spanish Organization of Organ Transplantation (ONT) to further start a multicentric collaboration for the realization of a project entitled "Normothermic perfusion of renal organoids in discarded kidneys for implantation in kidney transplant candidates". This manuscript represents an important step for this next project, and we would like to thank the comments and criticisms raised by the experts and the editor of the manuscript to improve our work and increase the level of accuracy in our experiments and measurements.

R#1: 2a) *The transplantation of the kidneys carrying humanoid organoids represent xenografts. The authors should describe the immune response.*

A: To describe the elicited xenogeneic pig-mediated immune response against human organoids we have characterized the adaptive and innate immune system employing phenotyping and quantitative techniques including flow cytometry and Milliplex assays.
First, we evaluated the cell percentages of myeloid cells and T cell lymphocytes in peripheral blood mononuclear cells (PBMCs) at different time points before and after transplantation (***Figure 4a*** **in this rebuttal lette**r and **New Figure 4 in the manuscript**; further information of the gating strategy in ***Figure 5*** **in this rebuttal letter** and as **New Supplementary Figure 11a in the manuscript**). ***Figure 4b*** **in this rebuttal letter** shows no significant differences in the percentages of total $CD3^+$ T lymphocytes, $CD4^+$ T helper and $CD8^+$ T cytolytic cells between the transplanted and the control groups at the indicated time points (T=0h, T=24h and T=48h).

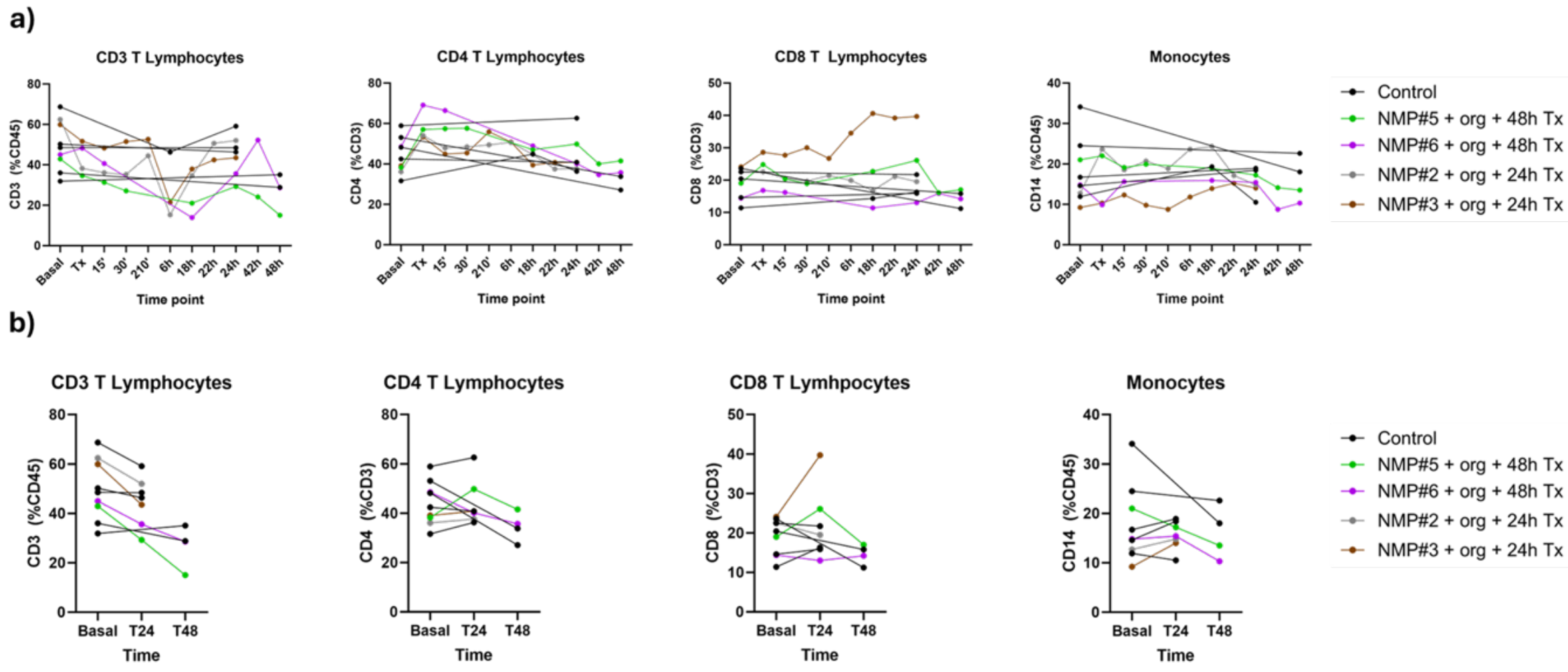

Figure 4. *Peripheral blood mononuclear cells of pigs at different timepoint after the perfusion and transplantation were analyzed by flow cytometry.* ***a****, Levels of total CD3 lymphocytes, CD4, CD8 or monocytes as percentage of total CD45 cells were analyzed.* ***b****, Levels of total CD3 lymphocytes, CD4, CD8 or monocytes as percentage of total CD45 cells were analyzed at times 0, 24 and 48 hours.*

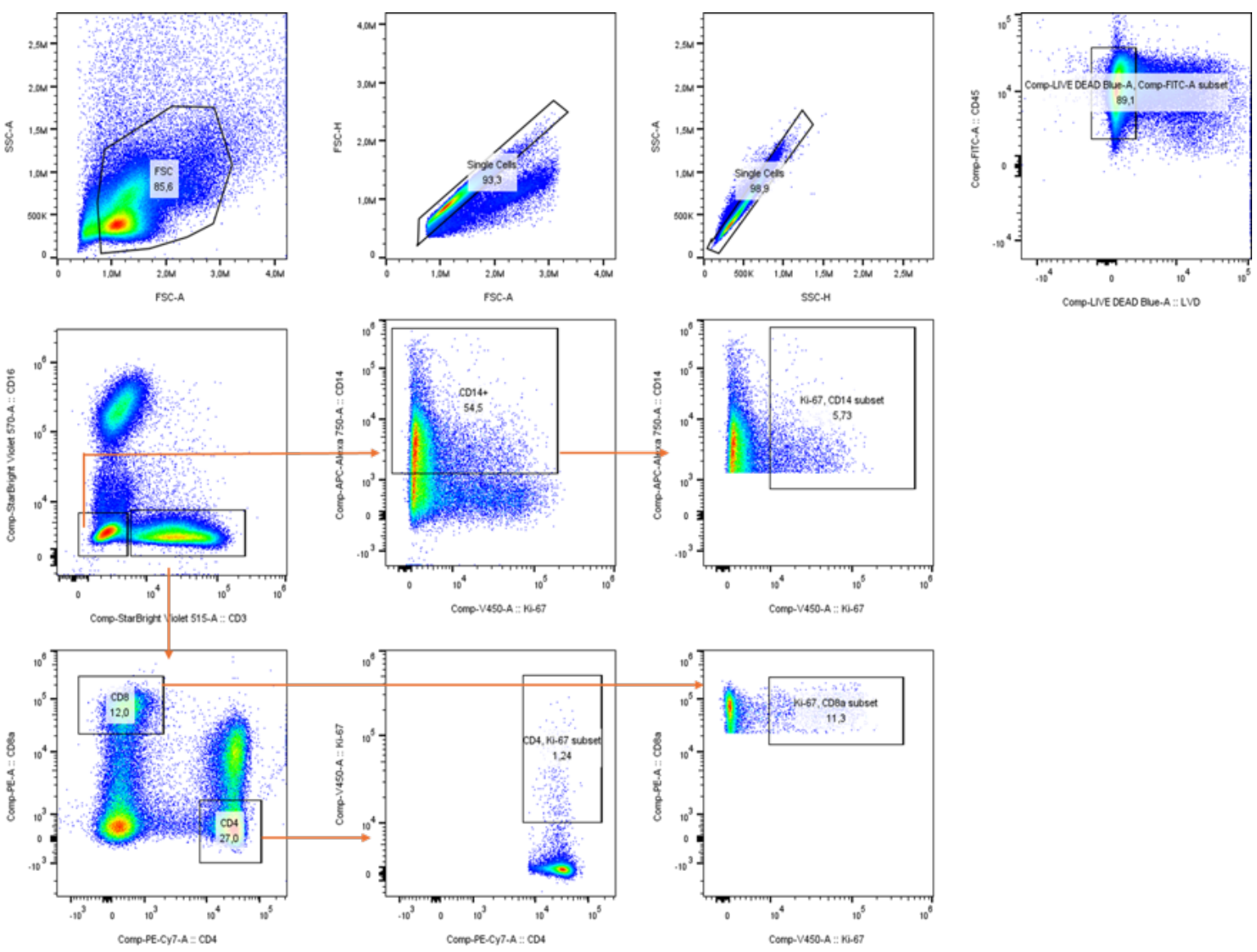

Figure 5*. Flow cytometry gating strategy for analyzing peripheral blood mononuclear cells of pigs at different timepoint after the perfusion and transplantation were analyzed by flow cytometry.*

Furthermore, our analysis indicates no significant differences within the control or transplanted groups longitudinally, overall suggesting that human organoids may not elicit major adaptive immune responses when implanted into pig kidney grafts in our experimental set up. Next, we measured the expression of *Ki-67*, a protein that is expressed only by dividing cells (PMID: 6206131) as an additional maker to evaluate the potential proliferative state of T lymphocytes prior and after transplant. ***Figure 6a,b*** **in this rebuttal letter** (**New Figure 3b and new Supplementary Figure 11c in the manuscript)** show no significant differences in the percentage of CD3, CD4 and CD8 T cell proliferation between groups or longitudinally within the same group. In addition, we have monitored the response of the

innate immune system by evaluating the percentage of classical monocytes (right panel ***Figure 4a,b*** **in this rebuttal letter,** and **New Figure 4a** and **Supplementary Figure 11b in the manuscript**). Consistent with the T cell immune response described above, our results indicate no significant differences in the percentage of monocytes or their proliferative status between groups at the indicated time points (T=0h, T=24h and T=48h). Overall, flow cytometric analysis shows no significant changes in the immunological response to human organoids implanted in xenogeneic pig kidney grafts.

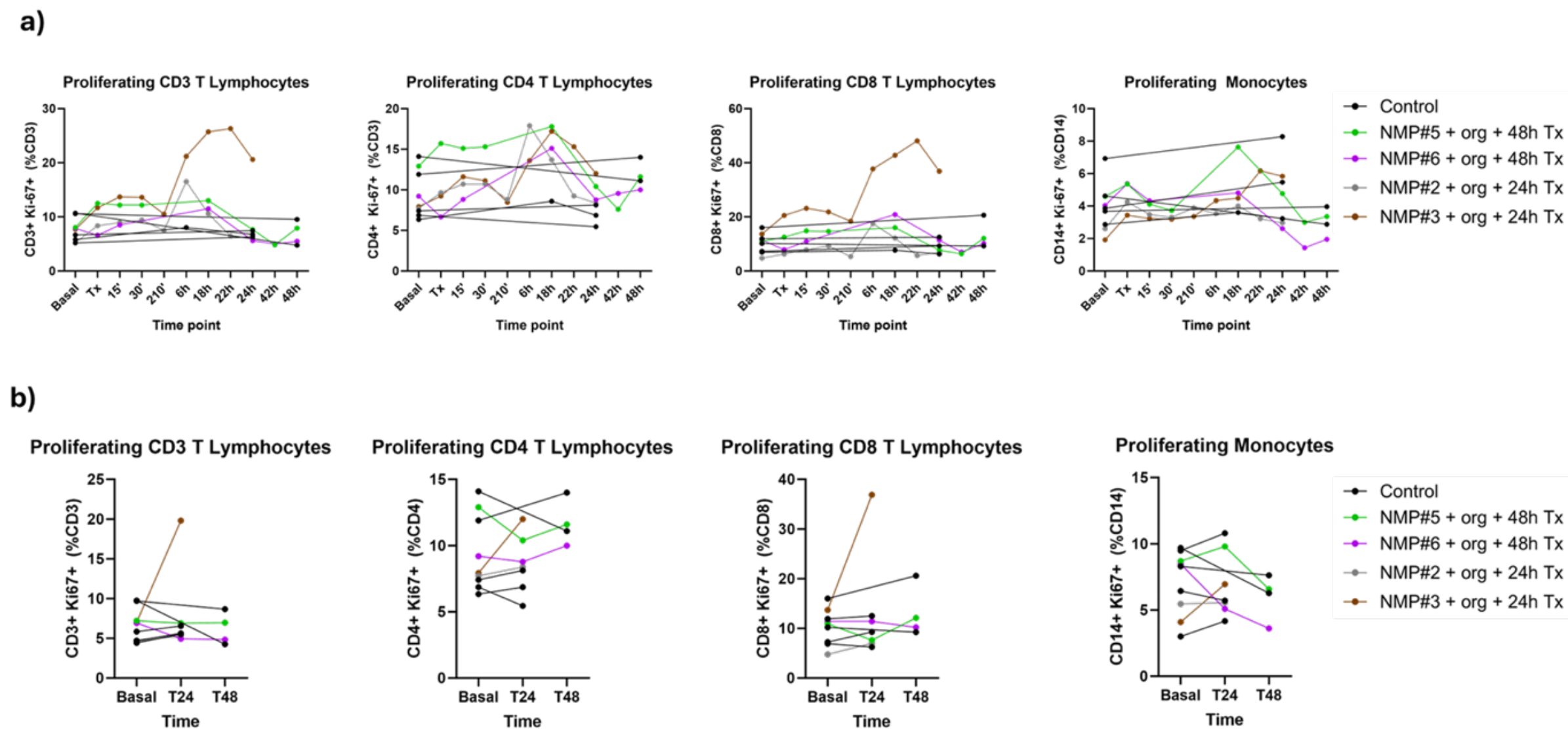

Figure 6. ***a***, *Proliferating levels of CD3 lymphocytes, CD4, CD8 or monocytes by the expression of Ki-67. Each line represents a single pig.* ***b***, *Proliferating levels of CD3 lymphocytes, CD4, CD8 or monocytes by the expression of Ki-67 at times 0, 24 and 48 hours. Each line represents a single pig.*

To further describe the immune response of kidney transplant recipients receiving human organoids we measured different immunological parameters in the serum *using MILLIPLEX®*, including cytokines, growth factors and inflammatory proteins. ***Figure 7*** **in this rebuttal letter** (**New Figure 4c in the manuscript**) shows no significant difference in the serum levels of CD31 and CD34 through the experimental time points. Importantly, CD31 and CD34 have been already used as markers of endothelial cell damage during kidney perfusion (PMID: 33903694 and 38807604). We next measured the levels of pro-inflammatory IL-6 and anti-inflammatory IL-10 (PMID: 32235253 and 24392313) and the results indicate no significant differences between groups. Similar results were observed when measuring plasma levels of pro-inflammatory IL-1B or antagonistic IL-1RA (PMID: 30995499). Serum levels of C-reactive protein (CRP), an independent risk factor for renal allograft loss that correlates with metabolic syndrome (PMID: 17524855), indicates no significant changes.

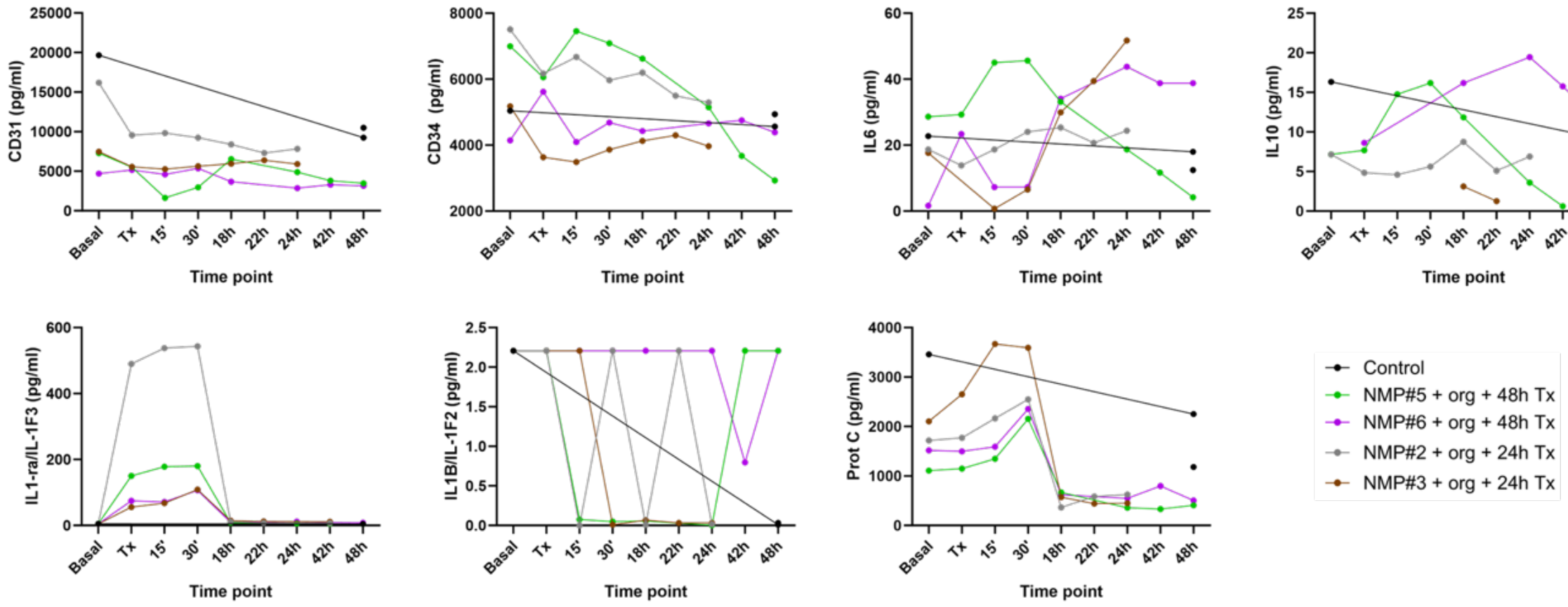

Figure 7. *Serum levels of CD31, CD34, IL6, IL10, IL1-ra, IL1B and C reactive protein measured by MILLIPLEX®. Mann-Whitney test was used to compared between the 2 experimental groups at 0, 24 and 48 hours, no statistical differences were found. Paired Wilcoxon matched-pairs signed rank test was used to analyze changes inside the same group at 0, 24 and 48 hours. P-values less than 0.05 were considered statistically significant.*

R#1: 2b) *Was the immune response the reason to choose a 48 hours observation period?*

A: Xenogeneic immune responses have been shown to occur within minutes or hours after transplantation and lead to hyperacute rejection and graft destruction within 24 hours (PMID: 35967435). In this same line, it has been previously shown that early after xenogeneic cross-circulation there is an increase in IL-6 and the IL-1 receptor antagonist (IL-1RA) in the plasma at reperfusion but approached baseline levels at the experimental end point after 24h (PMID: 37000867). We extended this endpoint to 48h and confirmed that immunological parameters are not significantly affected throughout the study (***Figure 8*** **in this rebuttal letter** and **New Supplementary Figure 11d in the manuscript**).

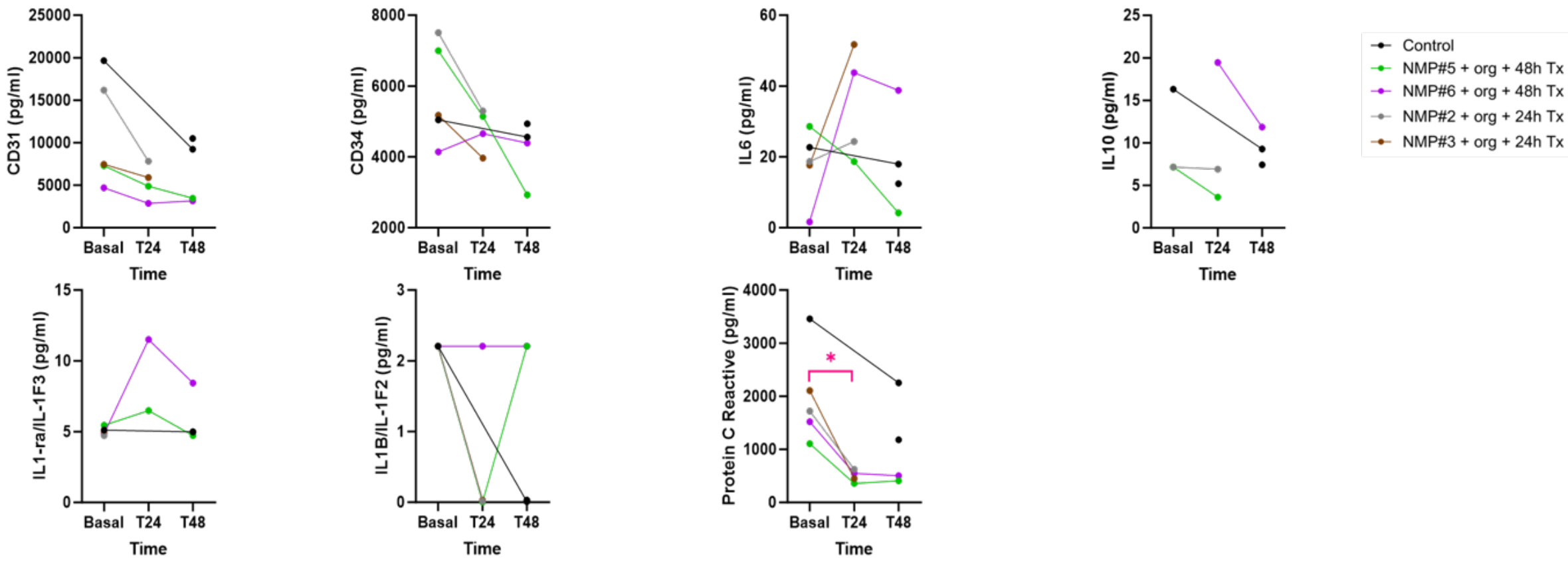

Figure 8. *Serum levels of CD31, CD34, IL6, IL10, IL1-ra, IL1B and C reactive protein measured by MILLIPLEX®. Mann-Whitney test was used to compared between the 2 experimental groups at 0, 24 and 48 hours, no statistical differences were found. Paired Wilcoxon matched-pairs signed rank test was used to analyze changes inside the same group at 0, 24 and 48 hours. P-values less than 0.05 were considered statistically significant.*

Into the light of our results, we conducted phenotyping analysis making use of a multimodal strategy combining morphological evaluation and immunophenotyping (CD68, CD15 and CD45) coupled to image quantification analysis to further ascertain initial immune responses in pig kidney xenografts after autotransplantation. Phenotyping was conducted on paraffin sections of kidneys samples

retrieved 24 or 48 h post-transplantation (including control lateral kidneys) as well as non-transplanted kidneys after NMP in the presence of organoids (**Figure. 4d in the manuscript and *Figure 9* in this rebuttal letter**). We confirmed the presence of innate immune cells (CD68+ and CD15+ cells, ***Figure 9 a,b*** in this rebuttal letter) and leukocytes (CD45+ cells, ***Figure 9c*** in this rebuttal letter) in all the experimental conditions. Results are shown quantifying the presence of the marker of interest for a total area and compared to control lateral counterparts (CTL). Details on the automated quantification are described in **Methods section in the manuscript** demonstrating no significant differences among the experimental groups.
We attach **in this rebuttal letter** a link to all the immunohistochemistry images from which this analysis was done (https://tinyurl.com/29k6d52d). Images can be visualized with the NDP.view2 Image viewing free software (https://www.hamamatsu.com/jp/en/product/life-science-and-medical-systems/digital-slide-scanner/U12388-01.html) or ImageJ2 (version 2.3.0) software (https://imagej.net/software/fiji/).

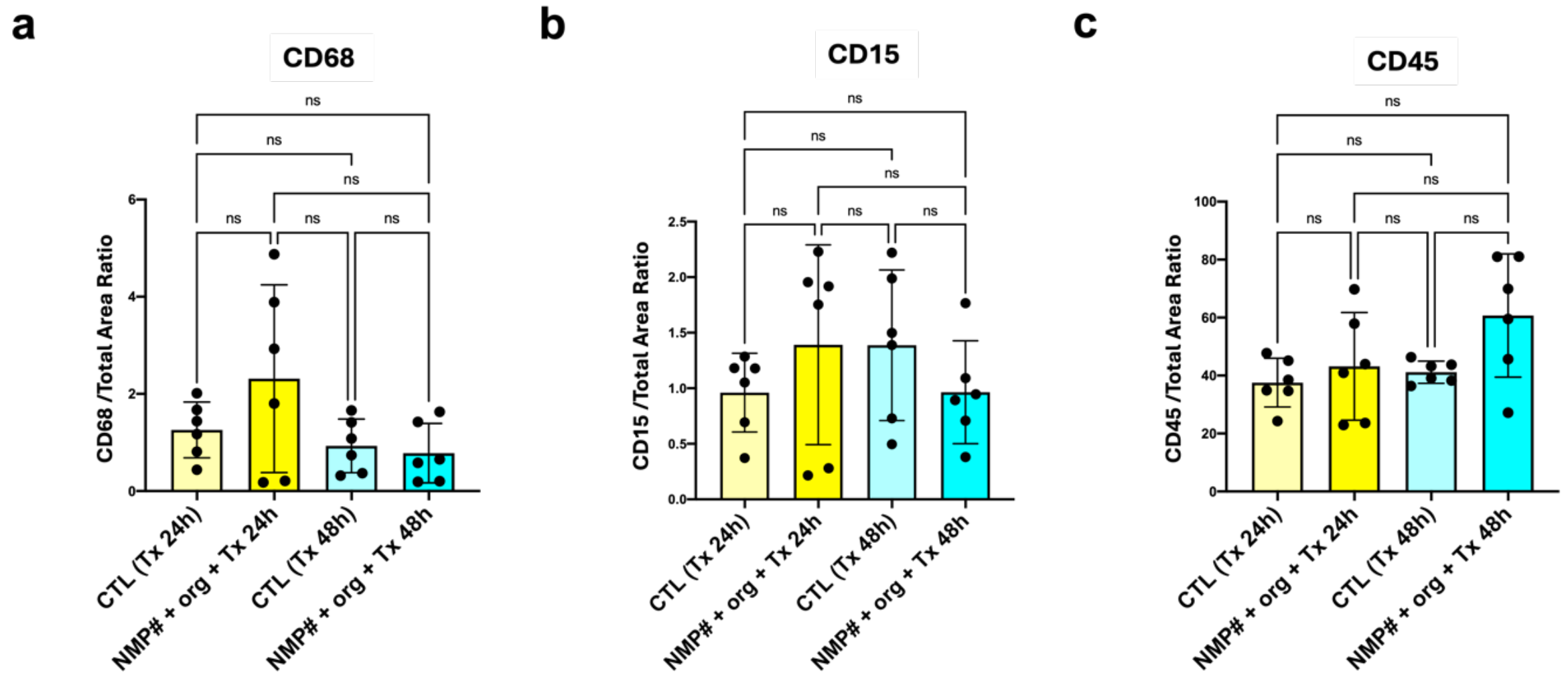

Figure 9. *Image quantification of CD68, CD15 and CD45 area relative to total tissue section area in renal cortical biopsies retrieved from kidneys 24 h and 48 h post-transplantation. Data are mean ± s.d. n = 3 kidneys/group with technical duplicates each. Kruskal-Wallis (two-sided) followed by Dunn's multiple comparisons test. ns, no statistical significance.*

R#1: 3) *It would be great to advance the testing of hPSC-derived kidney organoids tested in a discarded human kidney model.*

A: We agree with the expert for this insightful comment. We are at the present time setting up a collaboration with the Spanish Organization of Organ Transplantation (ONT) to further organize a multicentric collaboration for the realization of a project entitled "*Normothermic perfusion of renal organoids in discarded kidneys for implantation in kidney transplant candidates*". At the present time, and thanks to the coordination of the ONT we have identified three clinical sites which will participate in the study: University Hospital of La Coruña (already participating in the project, co-author of the study), The Marqués de Valdecilla University Hospital (HUMV) and The Ramón y Cajal University Hospital. The inclusion of the different sites has been done based on their capacity in kidney transplantation and the number of discarded human kidneys for implantation in kidney transplant candidates in the last 5 years. This analysis has been carefully done by the ONT (see this inform for 2023 as example: https://www.ont.es/wp-content/uploads/2024/01/BALANCE_2023_ONT_17012024.pdf). We are now setting up the normalized procedures for their presentation in the different committees and we expect to start the

project by October 2024. As the realization of these experiments are out of the scope of the current manuscript we would like to stress that the work conducted during this revision process has been key to address caveats and identify pitfalls when preparing the project in human kidneys, so we overall thank the unvaluable feedback from both experts to increase the quality of our work in the preclinical setting.

R#1: 4) *The number of xenolight positive cells is decreasing after transplantation. Was is the supposed mechanism for the loss?*

A: We would like to thank the reviewer for this observation. In the first submission the results corresponded to measures from one biological experiment from each experimental condition. During the revision process we have increased the number of biological replicates which now show that there is no statistically significant decrease in the number of Xenolight positive cells between the experimental groups. The results are shown in ***Figure 10*** **in this rebuttal letter** and as a **new panel in Figure 3 in the manuscript**.

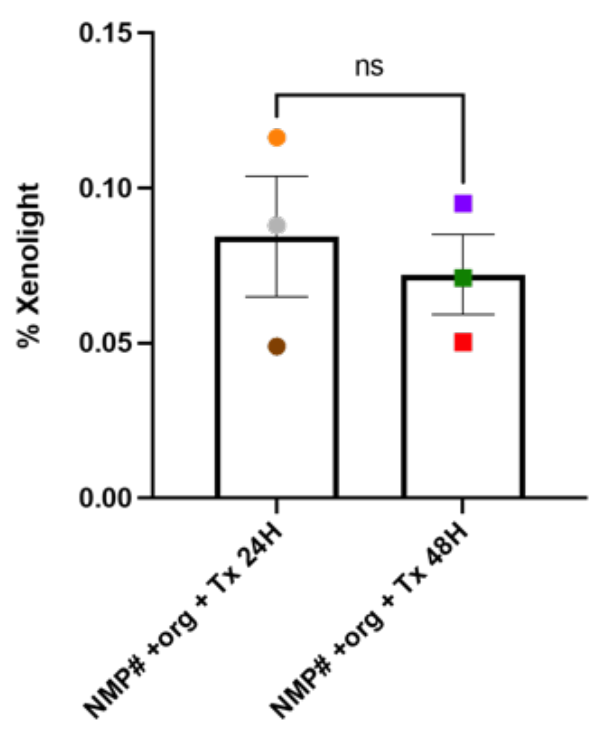

Figure 10*. Quantification of the percentage of Xenolight positive cells by flow cytometry in renal cortical tissue from organoid-infused kidneys 24 h and 48 h after in vivo transplantation in comparison with contralateral kidneys. Data are mean ± s.d. n = 3 biological replicates/group. ns, no statistical significance. Unpaired Student's t test.*

R#1: 5) *It is difficult to find the number and conditions of the experiments. This would be helpful to estimate experiments -related variations.*

A: We would like to apologize for this mistake. During the revision period we have increased the number of biological replicates and technical replicates.

In the previous version of our manuscript, we showed results on the use of dissociated kidney organoids and their infusion during NMP in 3 independent experiments. We also included results from a total of 5 independent experiments *ex vivo* corresponding to the infusion of intact kidney organoids during NMP (including 3 different experiments testing different organoid doses). We also included 2 different experiments on the infusion of kidney organoids during NMP for three hours and further autotransplantation for 24 h and 48 h (n=1 for each experimental group).

We also included 2 additional controls corresponding to mock experimental conditions (porcine kidneys infused with perfusate in normothermia for 3 hours).

Now we have performed 4 additional different experiments on the infusion of kidney organoids during NMP for three hours and further autotransplantation for 24 h and 48 h (n=2 for each experimental group). We have also added additional information corresponding to a mock experimental condition (one single experiment in which the porcine kidney was infused with perfusate for 3 hours and further autotransplanted after nephrectomy and recovered after 24h). This control condition has been used

for the new analysis conducted during the revision process following reviewer 1 and 2 comments, including: morphological analysis (H&E), biochemical analysis in urine and plasma.

Overall, we have made use of a total of 16 animals for the realization of this experiments:
Infusion of disaggregated cells from organoids during NMP (n=2)
Infusion of intact organoids during NMP (n=5)
Infusion of perfusate during NMP (n=2)
Infusion of intact organoids during NMP and auto-TX for 24 h (n=3)
Infusion of intact organoids during NMP and auto-TX for 48 h (n=3)
Infusion of perfusate during NMP and auto-TX with nephrectomy for 24 h (n=1)

Table 2 **in this rebuttal letter** summarizes all the experiments conducted in the manuscript.

ANIMAL NAME	INTERNAL SAMPLE NAME	EXPERIMENTAL CONDITION	SAMPLE CODING (before Tx)	SAMPLE CODING (after Tx)	INCLUDED IN THE FIRST SUBMISSION	INCLUDED IN THE SECOND SUBMISSION	FIGURES CONTATINING THIS EXPERIMENT
ANIMAL A	E250	Perfused with single cells	Disaggregatted 1	N/A	YES	YES	**Suppl. Fig. 6**
ANIMAL B	E256	Perfused with single cells	Disaggregatted 2	N/A	YES	YES	**Suppl. Fig. 6**
ANIMAL C	E251	PERFUSED with intact organoids low dose	Low dose	N/A	YES	YES	**Suppl. Fig.5**
ANIMAL D	E257	PERFUSED with intact organoids low dose	Low dose	N/A	YES	YES	**Supp. Fig. 5**
ANIMAL E	E999	PERFUSED with intact organoids low dose	Low dose	N/A	YES	YES	**Suppl. Fig. 5**
ANIMAL F	E998	PERFUSED with intact organoids high dose	High dose	N/A	YES	YES	**Suppl. Fig. 5**
ANIMAL G	E996	Perfused with Perfusate alone	Mock	N/A	YES	YES	**Suppl. Fig. 5**
ANIMAL H	E997	Perfused with Perfusate alone	Mock	N/A	YES	YES	**Suppl. Fig. 5**
ANIMAL 1	Kidney 1	**PERFUSED without organoids and TX with Nephrectomy**	MOCK +24H Tx	MOCK +24H Tx	NO	YES	**Suppl. Figs 8, 9, 10**
ANIMAL 1	Kidney 2	**CTRL of Kidney 1**		MOCK Ctl 4H	NO	YES	**Supp.Figs. 8, 9, 10**
ANIMAL 2	Kidney 3	**PERFUSED with intact organoids and Tx 48 H**	NMP#4+org	NMP#4+org+48h Tx	NO	YES	**Fig. 3, 4, Suppl. Figs. 8, 9, 10**

ANIMAL 2	Kidney 4	**CTRL of Kidney 3**	N/A	CTL#4	NO	YES	**Figs.3, 4 Suppl.Figs.8, 9, 10**
ANIMAL 3	Kidney 5	**PERFUSED with intact organoids and Tx 24 H**	NMP#1+org	NMP#1+org+24h Tx	NO	YES	**Figs. 3,4 Suppl. Figs. 8, 9, 10**
ANIMAL 3	Kidney 6	**CTRL of Kidney 3**	N/A	CTL#1	NO	YES	**Figs. 3,4 Suppl. Fig. 9**
ANIMAL 5	Kidney 9	**PERFUSED with intact organoids**	NMP#0+org	N/A	NO	YES	**Suppl. Figs. 7, 9**
ANIMAL 5	Kidney 10	**CTRL of Kidney 9**	CTL#0	N/A	NO	YES	**Suppl. Figs. 7, 9**
**ANIMAL 6**	**Kidney 11**	**PERFUSED with intact organoids and Tx 48 H**	**NMP#5+org**	**NMP#5+org+48h Tx**	NO	YES	**Figs. 3, 4 Suppl. Figs. 8,9,10,11**
**ANIMAL 6**	**Kidney 12**	**CTRL of Kidney 11**	**N/A**	**CTL#5**	NO	YES	**Fig. 4 , Suppl. Fig. 9**
**ANIMAL 7**	**Kidney 13**	**PERFUSED with intact organoids and Tx 48 H**	**NMP#6+org**	**NMP#6+org+48h Tx**	NO	YES	**Figs. 3, 4 Suppl. Figs. 8, 9, 10, 11**
**ANIMAL 7**	**Kidney 14**	**CTRL of Kidney 13**	**N/A**	**CTL#6**	NO	YES	**Fig. 4 Suppl. Fig. 9**
ANIMAL 8	Kidney 15	**PERFUSED with intact organoids and** Tx 24 H	NMP#2+org	NMP#2+org+24h Tx	NO	YES	**Fig. 4 Suppl. Figs. 8, 9, 10, 11**
ANIMAL 8	Kidney 16	CTRL of Kidney 15	N/A	CTL#2	NO	YES	**Fig. 4 Suppl. Figs. 8,9**
**ANIMAL 9**	**Kidney 17**	**PERFUSED with intact organoids and Tx 24 H**	**NMP#3+org**	**NMP#3+org+24h Tx**	NO	YES	**Figs. 3, 4 Suppl. Figs. 8, 9, 10, 11**
**ANIMAL 9**	**Kidney 18**	**CTRL of Kidney 17**	**N/A**	**CTL#3**	NO	YES	**Suppl. Figs. 8,9**

Table 2. *List of experiments conducted within the lifetime of the project. CTL: control; N/A: not applicable.*

R#1: 6) *The conclusion that "This work paves the way to the utilization of hPSC-kidney organoids for ex vivo cell-based therapies to improve graft function before transplantation" is overstating the content of the study.*

A: We would like to thank the expert for this important remark. We have modified this sentence from the current version of the manuscript.

R#1: 7) *I am not qualified to assess the methodology of organoid generation.*

A: We would like to thank all the comments and intense revision performed by the expert which overall has increased the level of accuracy and detail when presenting the organoid data and results.

Reviewer (R) #2:

The authors have extensively evaluated the effect of cell density of posterior intermediate mesoderm (PIM)-committed cells on kidney organoid composition using various techniques including, but not limited to, immunocytochemistry, labeling of WT1 precursors and single cell mRNA sequencing. They then infuse a population of organoids using normothermic machine perfusion of porcine kidneys and autotransplant the perfused kidneys into the donor pig. They find that they can detect human organoid cells in the pig kidney. They confirm that there is little effect on a number of physiological parameters. There are, however, a number of issues with the manuscript.

R#2: 1) *The data are interesting in making the point that the density of PIM-committed cells upon transition from one to two-dimensional culture determines the number of nephron segments and metabolic characteristics of the developing organoid. It seems, however, that there is not a very good connection between these data and the intraarterial organoid infusion to the pig. It is not clear how they decided which protocol to use to obtain organoids (different densities of PIM-committed cells) on infusion and why they chose a normothermic perfusion temperature. There is no indication of any function of the organoids that have been infused, and no information about vascularization etc, so it is difficult to see how important the pig data are beyond the fact that all the organoid cells do not die after injection. We do not have an indication of what percentage of the cells survive the transplantation. Recognizing that one cannot count all the cells in the transplanted organ perhaps there can be some estimate of survival range by sampling regions. A more interesting comparison in terms of organ regeneration would be to induce injury/kidney disease and evaluate whether organoids or dissociated cells into the host animal had any effect on kidney function.*

Authors (A): We appreciate the reviewer's important feedback and the very positive evaluation of our work. We divide here the comments of the expert and answer to them:

R#2: 1.1) *The data are interesting in making the point that the density of PIM-committed cells upon transition from one to two-dimensional culture determines the number of nephron segments and metabolic characteristics of the developing organoid. It seems, however, that there is not a very good connection between these data and the intraarterial organoid infusion to the pig. It is not clear how they decided which protocol to use to obtain organoids (different densities of PIM-committed cells) on infusion and why they chose a normothermic perfusion temperature.*

A: We would like to thank the expert for this important remark. The rational beyond our strategy was to dissect the effect of different densities of PIM-committed cells to identify those conditions leading to the generation of organoids exhibiting higher traits of differentiation qualitatively and quantitatively. Moreover, the conditions of choice should fulfil an important criterion which related to further interventions ensuring scalability. In this regard, our findings revealed that 0.5 K kidney organoids apart from guaranteeing fidelity (in terms of differentiation) and scalability also allow for their application through the renal artery (compared to organoids of bigger sizes). On the other hand, we would like to highlight that the experimental conditions used for the infusion of kidney organoids during NMP largely benefited from previous findings on the use of mesenchymal stem cells in porcine kidneys as described in our manuscript. We have now briefly stressed the connection between the different sections of the manuscript hoping to convince the expert about the relevance of our findings.

<u>R#2: 1.2)</u> *There is no indication of any function of the organoids that have been infused, and no information about vascularization etc, so it is difficult to see how important the pig data are beyond the fact that all the organoid cells do not die after injection. We do not have an indication of what percentage of the cells survive the transplantation. Recognizing that one cannot count all the cells in the transplanted organ perhaps there can be some estimate of survival range by sampling regions.*

A: We thank the reviewer for this important comment. During this revision process we have explored potential functional responses of the produced organoids. We have provided further experimental evidence about *the function of kidney organoids by* conducting very preliminary experiments described **in this rebuttal letter** as detailed in our response to Reviewer 1 (**R#1**; see our response to **R#1.2** in this letter), in the ***<u>Figure 2</u>* in this rebuttal letter**. Specifically, we have exposed kidney organoids in front of a very well-known nephrotoxic agent called Cisplatin for 24h as an assay to assess potential responses of kidney organoid tubular-like cells (demarked with *Lotus Tetraglobus Lectin*-LTL) through the detection of kidney-specific injury molecule KIM1 in comparison to control counterparts (without cisplatin). Furthermore, we have assessed the impact of cisplatin treatment by RT-qPCR. Our results suggest that 0.5 Ks organoids may represent a new differentiation pipeline to assess functional responses which we aim to explore in the future in another manuscript

To provide some feedback to the expert on evidences of *integration* (see our response **R#1.3** in this rebuttal letter, ***<u>Figure 3</u>* in this rebuttal letter** and new **panels g,h in Figure 3 of the manuscript** and **panel n in Supplementary Fig. 7 in the manuscript**) we have conducted an analysis for the detection of ECAD and LTL positive cells, as well as for the tubule injury marker KIM-1, in three biological experiments corresponding to kidney biopsies of specimens undergoing organoid infusion during NMP (**Supplementary Fig. 7n in the manuscript**) and further autotransplantation for 24 and 48 hours (**Figure 3g,h in the manuscript**). Immunofluorescence analyses were conducted in consecutive paraffin sections showing positivity for Alu. In this manner, we aim to show the integration of human cells within the porcine kidney tissues. These results are shown in ***<u>Figure 3</u>* in this rebuttal letter**.
All in all, we hope to convince the expert of the relevance of our work while also acknowledging her/his comments to improve our investigation. We also hope to convince the expert about the importance of our work besides showing that organoid cells do not die after injection. Importantly and in the light of her/his important observation we have also estimated the number of total cells that survive after the transplantation. In this regard, we have prepared ***<u>Figure 11</u>* in this rebuttal letter** which briefly explains the experimental procedure that we have used for the estimation of cell survival rate. Briefly, renal biopsies are thaw and mechanically and enzymatically digested for FACS analysis (***<u>Figure 11</u>* in this rebuttal letter**). In ***<u>Figure 11 b</u>*** we show the gating strategy for the detection of live cells (DAPI negative) taking as an example a control sample (CTL) which corresponds to a control lateral kidney after 24 h [CTL (24h Tx)]. This example highlgiths the gating strategy for the accurate detection of Xenolight positive cells (Xenolight+) 24 h and 48 h post Tx (***<u>Figure 11 c,d</u>* in this rebuttal letter**). In ***<u>Figure 11 e</u>* in this rebuttal letter,** we show the calculations performed which consider the initial weight of the infused kidneys (the average weight of a kidney is 100g) and the organoid dosage used in our experimental set up to further estimate the number of Xenolight positive cells (Xenolight+) per gram of renal tissue (assuming that each organoid has 1500 cells and that 62.5% of cells are labelled positive for Xenolight) (***<u>Figure 11 e</u>* in this rebuttal letter**). Finally, an in order to answer to the expert, we have calculated the detection rate (%) of human organoid-derived cells present in porcine kidneys after transplantation by dividing i) the average Xenolight+ organoid derived-cells per gram of renal tissue that are detected by FACS (average value of 350-400 Xenolight+ cells/g) by ii) the estimated average value of Xenolight+ organoid-derived cells per gram of renal tissue that are initially infused into the kidney during NMP (144491 Xenolight+ organoid-derived cells per gram). Overall, these calculations highlight a detection rate of 0.25 % of human organoid-derived cells in porcine kidneys after transplantation (***<u>Figure 11 f</u>* in this rebuttal letter**).

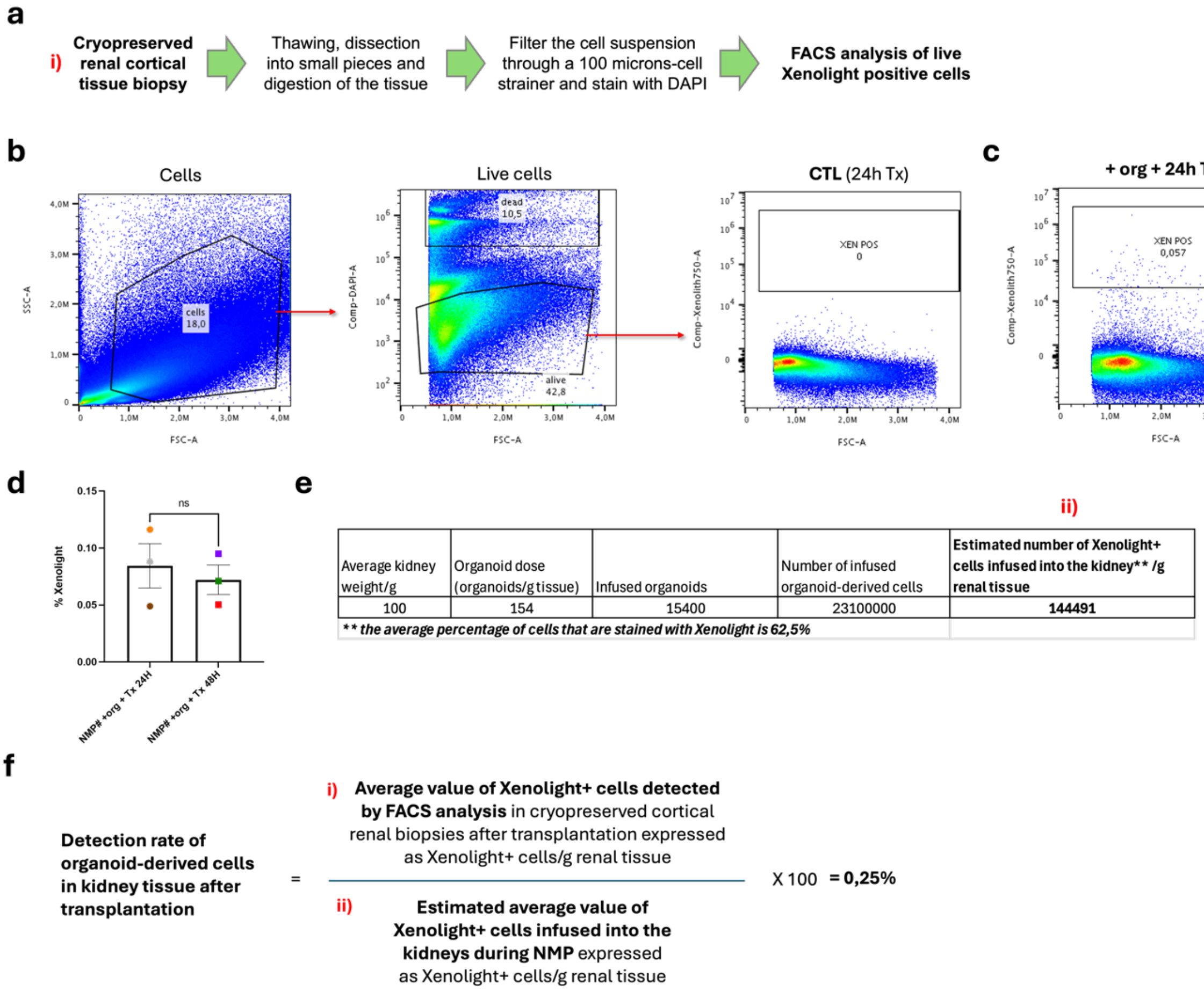

***<u>Figure 11</u>. a**, Six-millimeter cortical punch biopsies from kidney cortex were collected 24 or 48h after transplantation of kidneys undergoing NMP with Xenolight labelled organoids and cryopreserved for further analysis. To assess the presence of organoid cells by flow cytometry, frozen biopsies were thawed, dissected and submitted to a process of tissue digestion using collagenase type IV (17104019, Thermo Fisher) for 45 min at 37 °C. The resultant cell suspension was filtered through a 100micron-cell strainer. DAPI was added to determine cell viability. Next, cell suspensions were analyzed by FACS for the detection of live Xenolight+ cells. **b,c,** Flow cytometry gating strategy for analyzing live Xenolight+ cells in kidney biopsies collected 24 or 48h after transplantation of kidneys. Representative FACS plots for samples from contralateral (CTL) and transplanted (+ org + 24h Tx) kidneys are shown, respectively. **d**, Quantification of the percentage of Xenolight+ cells in renal cortical tissue from organoid-infused kidneys 24 h and 48 h after in vivo transplantation by flow cytometry. Data are mean ± s.d. n = 3 biological replicates/group. ns, no statistical significance. Unpaired Student's t test. **e**, Estimated number of Xenolight+ cells derived from human kidney organoids that are initially infused into porcine kidneys undergoing NMP. **f**, The detection rate (%) of human organoid-derived cells present in kidneys after transplantation is estimated by dividing i) the average Xenolight+ organoid derived-cells per gram of renal tissue that are detected by FACS by ii) the estimated average value of Xenolight+ organoid-derived cells per gram of renal tissue that are initially infused into the kidney during NMP.*

<u>R#2: 1.3)</u> *A more interesting comparison in terms of organ regeneration would be to induce injury/kidney disease and evaluate whether organoids or dissociated cells into the host animal had any effect on kidney function.*

A: We thank the expert for another important remark. The objective of our study is to pave the way for the overarching goal to employ kidney organoids during NMP for ulterior regenerative applications in vivo. The work presented here has been focused on the generation of novel procedures allowing for the generation of kidney organoids in a scalable manner while guaranteeing fidelity (in terms of

differentiation extent and cellular composition). Of equal importance we have established conditions for the infusion of intact organoids or dissociated cells during NMP and studied cell engraftment via qualitative and quantitative techniques. Those conditions have allowed for the definition of in vivo autotransplantation experiments to further evaluate the safety of our approach. Thanks to this revision process and into the light of the expert comments and suggestions we have now also worked on the quantitively evaluation of the possible elicited immune response systemically and in pig renal tissues. These results are included in this rebuttal letter (see response to reviewer 1 **R#1.2**, ***Figure 4, Figure 5, Figure 6, Figure 7*** and ***Figure 8*** in this rebuttal letter), and as main **Figure 4** and **Supplementary Figure 11 in the new version of the manuscript**. Furthermore, we have also started to analyze cell engraftment by the detection of genetic material of human origin and cells via confocal microscopy analysis. These results are shown in this rebuttal letter (***Figure 3*** in this rebuttal letter) and also as main **Figures 3g,h** and **Supplementary Figure 7n** in the new version of the manuscript. We believe that this step-by-step approach should be done before the use of organoids for regenerative purposes. In this line, and as cited in our response to reviewer 1 **R#1.3** we have set up a collaboration with the Spanish Organization of Organ Transplantation (ONT) to further organize a multicentric collaboration for the realization of a project entitled "Normothermic perfusion of renal organoids in discarded kidneys for implantation in kidney transplant candidates". Within the next months we will be able to start the new project involving the participation of different transplantation sites and further apply the knowledge generated here in the human context allowing to finally validate the use of human kidney organoids during NMP in human discarded kidneys presenting clinical indications and thus test for effect of kidney organoids *ex vivo*.

R#2: 2) *It is unclear whether the authors utilized the micro-organoids produced by their protocols for their downstream transplant studies. Authors should conclusively state whether or not their micro-organoids were used in any pig studies. (Authors do briefly state micro-well organoids were used in the figure legend of supp. Fig 7… But not in the Results or any main body figure legends).*

A: We thank the reviewer's comment. In this work we report a novel procedure for the generation of kidney micro-organoids from hPSCs and their infusion into porcine kidneys during NMP and autotransplant into donor pigs. This is the first time that kidney micro-organoids produced by our procedure are used in a pig study. We apologize if this has not been clearly stated in the manuscript. We have now briefly rephrased this part in the revised manuscript by adding the word "novel" or "new" in the corresponding sections (Results and Discussion).

R#2: 3) *Much of the key data in the manuscript are in the Supplemental figures. It is not clear which this distribution of Figures (main manuscript/supplement) was chosen by author.*

A: We appreciate the reviewer's remark. In the revised manuscript we have now reorganized the figures in the manuscript as follows: **Figure 1 and 2** and **Supplementary figures 1 to 4** now show key results on the hPSC-derived kidney organoid generation methodology and characterization, including the scale-up production of micro-organoids via the generation of a reporter cell line. **Figure 3 and 4** and **Supplementary figures 5 to 11** show key results on the infusion of intact organoids into porcine kidneys during NMP and after their transplantation into pig recipients for 24h and 48h, including additional transplantation experiments and new data to evaluate potential immune response (systemic and tissue) after transplantation (**new Figure 3g,h**, **new Figure 4 and Supplementary Figure 7n, Supplementary Figure 11 in the manuscript,** and ***Figures 3 to 9*** **in this rebuttal letter**). Multiple parameters have also been evaluated during NMP (in perfusate, urea and blood and urine and blood from the animals) as well as during early reperfusion times and at the end of transplantation (urine, plasma and blood).

R#2: 4) *A major conclusion of the work is that the density of PIM-committed cells is key to differentiation and metabolism with the higher densities resulting in fewer proximal tubules and less oxidative metabolism. If one looks at the various panels in Supplemental figures 1 it is clear that the more PIM-committed cells that are combined the bigger the organoid is. The changes in outcome may relate to the fact that there is more hypoxia in the center of the larger organoids and this would be expected to drive glycolytic metabolism and fewer segments such as proximal tubule segments that were dependent upon oxidative phosphorylation. There are no controls for cell size.*

A: We again would like to thank the expert for this important comment. To respond to her/his remark we have proceed to semi-quantitative measure mitochondrial mass and activity at the cellular level in 0.5 K and 250 K kidney organoids using Mito Tracker Red. In ***Figure 12*** **in this rebuttal letter** we show the detection of Mito Tracker Red positive cells via confocal microscopy in 0.5 K and 250 K kidney organoids (***Fig. 12 a***) and image analysis quantification (***Fig. 12 b***) highlighting the detection of higher abundancy of Mitotracker Red positive cells in 0.5 K kidney organoids compared to 250 K kidney organoids as also confirmed by FACS (***Fig. 12 c***). Further analysis by RT-qPCR showed the detection of glycolitic (LDHA) and fibrotic markers (FN1 and PDFGRa) at higher levels in 250 Ks kidney organoids compared to 0.5 K counterparts. These observations together with our previous results included in the first version of the manuscript revealing that 0.5- and 8-Ks-PIM-committed 3D spheroids exhibited an OXPHOS bioenergetic phenotype compared to 100- and 250- Ks (***Fig. 1h*** **in the manuscript**) overall suggest that promoting cell-to-cell contact represents an affordable approach to induce an OXPHOS-mediated metabolic profile driving proximal tubular differentiation at the transcriptomic and phenotypic level. These conclusions were done into the light of the detection of prominent proximal tubular structures (LTL^{+}) qualitatively (as shown in the previous version of our manuscript by confocal microscopy analysis and image quantification, RT-qPCR and scRNAseq in **Figure 1d-g,i-j in the manuscript**). During the revision process we have also performed quantitative assays, including the detection of **LTL+** cells by FACS which also highlight the derivation of more LTL+ in 0.5 Ks compared to 250 Ks kidney organoids (*please see* our new results in ***Figure 18*** **in this rebuttal letter** which are now also presented in **Figure 1k in the manuscript**).

We would like to highlight that the results presented here cannot be linked to the presence of more necrotic cells in the core of 250 Ks compared to 0.5 Ks kidney organoids as suggested after our analysis by confocal microscopy analysis and image analysis quantification of Caspase 3 positive cells (*please see* ***Figure 13*** **in this rebuttal letter**).

As for the question on "*controls for cell size*" we would like to highlight that all image analysis quantification shown in this rebuttal letter or in the manuscript is done based on the detection of the marker of interest in the same area for a given experimental condition (e.g., 0.5 K vs 250 K). We were not sure about the comment on "*control size*" and we hope that we have understood the question. In case the expert needs further clarification, we would be extremely honored to provide more information regarding this query.

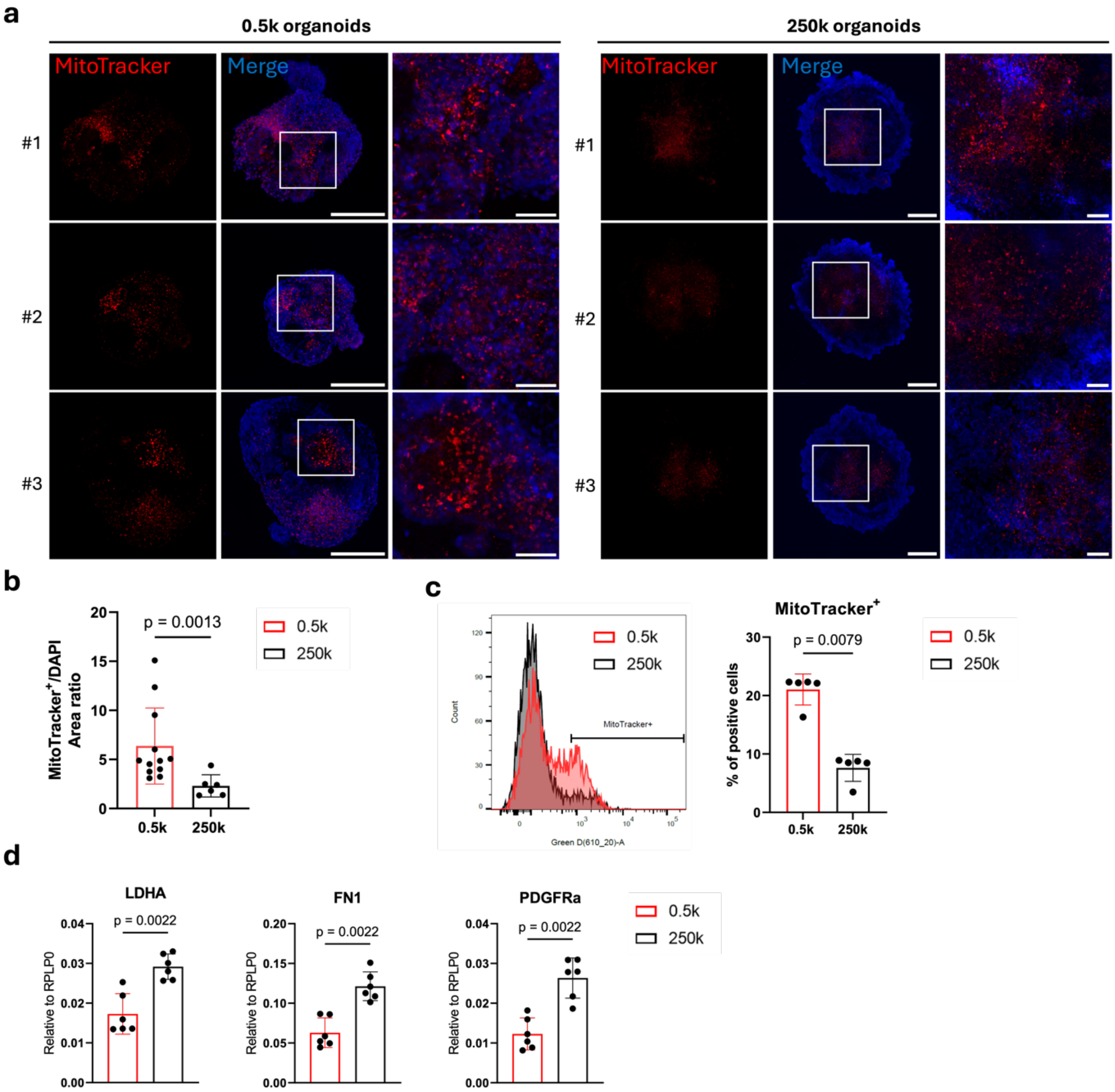

Figure 12*.** ***a, *Representative confocal images of 0.5k and 250k kidney organoids at day 16 of differentiation stained for MitoTracker™ Red CMXRos (Mito Tracker +, red) that labels active mitochondria and DAPI (blue). Scale bars, 200 µm, 50 µm (magnified views).* ***b***, *Image quantification of Mito Tracker + area relative to DAPI total area in 0.5 K (red) and 250 K (black) kidney organoids. Data are mean ± s.d. n = 12 (0.5k organoids), n = 6 (250k organoids). Mann-Whitney test.* ***c***, *Representative histograms representing Mito Tracker Red + (Mito Tracker +) cells from 5 independent cell sorting experiments.* ***c***, *Quantification of the % of LTL+ Mito Tracker + in 0.5k and 250k kidney organoids by FACS analysis. Data are mean ± s.d. n = 5 biological replicates from a pool of 12 organoids each. Mann-Whitney test.* ***d***, *RT-qPCR analysis on 0.5k (red) and 250k (black) kidney organoids at day 16 of differentiation. Dataare mean ± s.d. n= 3 biological samples per group. Each sample is a pool of 12 organoids/group with three technical replicates each. Mann-Whitney test.*

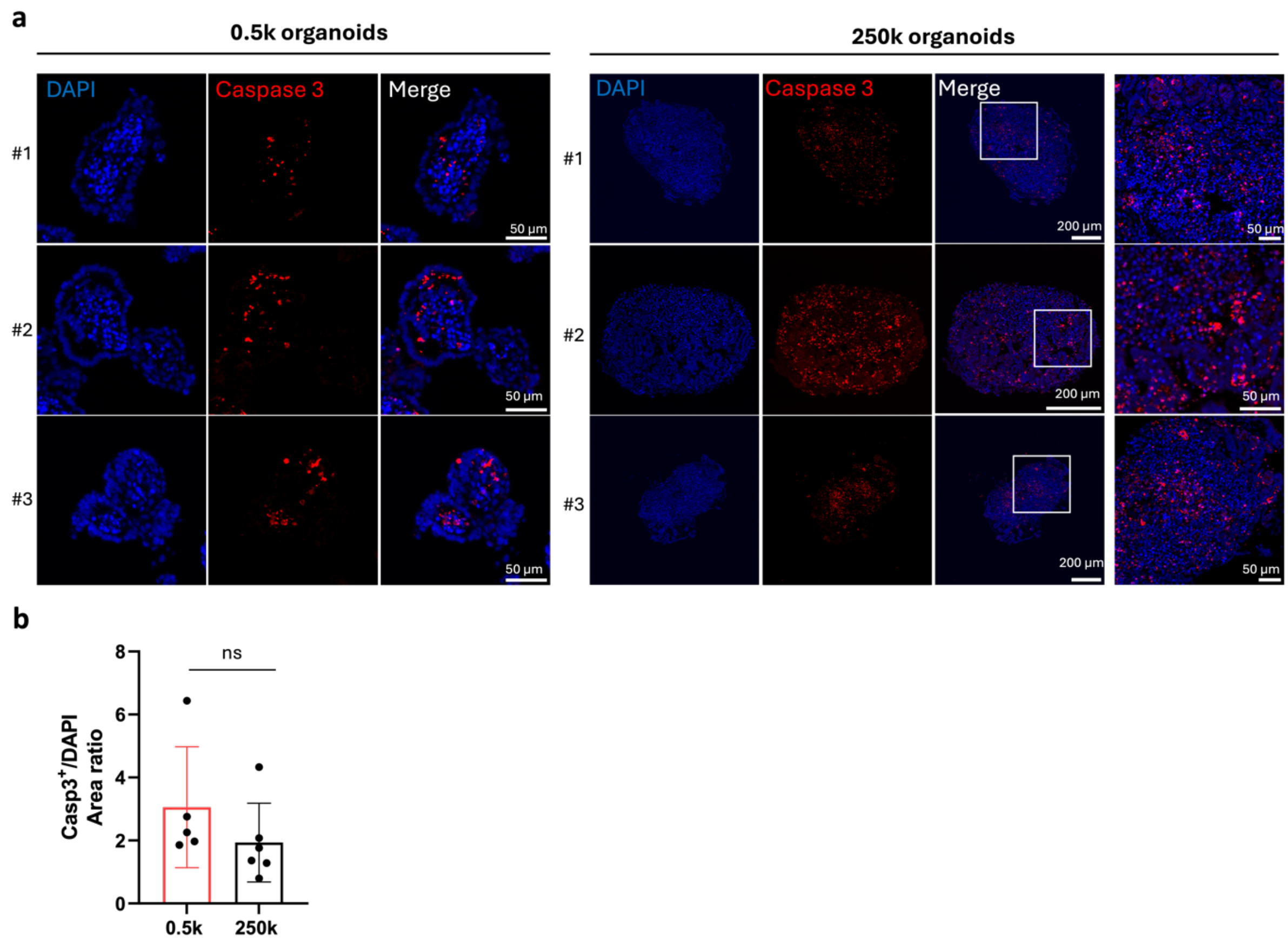

Figure 13. ***a***, *Representative confocal images of 0.5k and 250k kidney organoids at day 16 of differentiation stained for Caspase 3 (red) that labels death cells and DAPI (blue). Scale bars, 200 µm, 50 µm (magnified views).* ***b***, *Image quantification of Caspase 3 area relative to DAPI area in 0.5 K and 250 K kidney organoids. Data are mean ± s.d. n = 5 (0.5k organoids), n = 6 (250k organoids). ns, no statistical significance. Mann-Whitney test.*

R#2: 5) *Fig. 1: The authors come to the conclusion that "promoting cell-cell contact induces OXPHOS, which drives PT differentiation." but they do not present data on cell-cell contact to be able to reach that conclusion? They infer from the fact that higher cell density organoids have less differentiation… However, the larger organoids could have just as much cell-cell contact as the smaller organoids?*

A: We would like to thank the reviewer for yet another important comment. To answer this question, we have performed additional bioinformatic analysis on our scRNA seq data. Specifically, we selected gene sets that included the term "Adhesion" from the GO Biological Processes database from MSigDB and obtained scores for each of them using the AddModuleScore_Ucell function from the UCell R package to provide further information on possible adhesion-realted gene networks/processes differentially expressed comparing 0.5 K vs 250 K kidney organoids In general terms, our analysis shows that the resulting scores were similar between the 250K and 0.5K organoids for the gene sets of interest, though in most cases they seemed slightly higher in mesenchyme for the 0.5K condition (***Figure 14*** **in this rebuttal letter)**. As the question raised by the expert is of extremely relevance we aim to conduct proteomic analysis soon to be able to provide further information about how cell-to-cell contact is differentially regulated in our experimental conditions. At the present time we cannot present this new analysis in the revised manuscript, which is beyond the scope of our work. Again, we would like to thank the expert feedback to improve our work and to open new questions which will be explored in the context of a new manuscript.

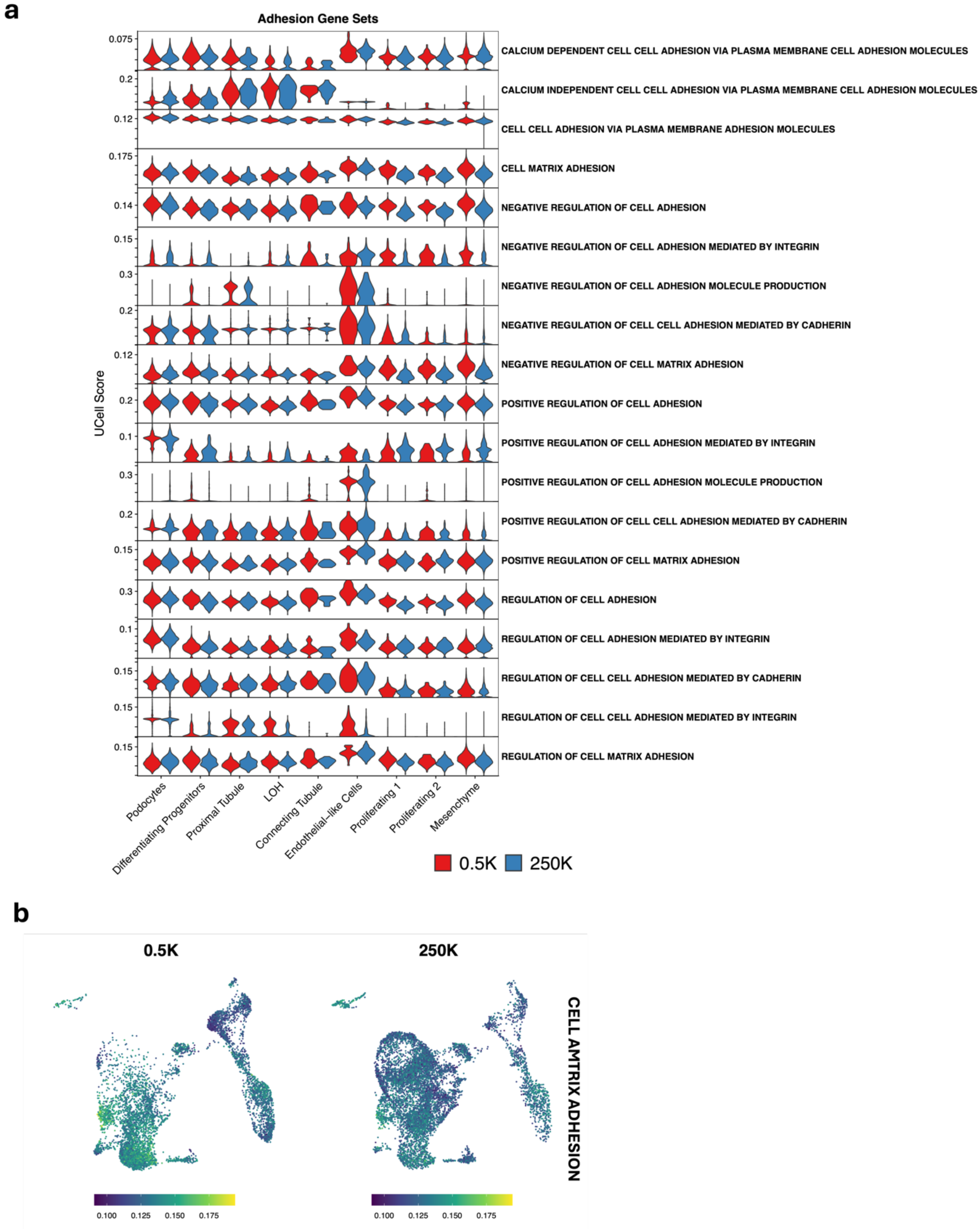

***Figure 14**. a,* *Violin plots representing UCell scores for GO Biological Processes related to cell adhesion for the different cell types split by condition. **b,** Uniform manifold approximation and projection (UMAP) representing the UCell scores for selected GO Biological Processes related to cell adhesion. Colour intensity goes from minimum to maximum score for each gene set.*

<u>R#2: 6)</u> *Many published organoid protocols employ 3D aggregation to stimulate differentiation. What appears to be novel is their modified protocol and techniques to mass produce micro-organoids. This protocol can be scaled up and employed for organoid production for use in disease modeling or prior to engraftment into kidneys or scaffolds to produce renal replacement options. The authors, however, do not comment on the scale-up potential.*

A: We agree with the reviewer's remark that our micro-organoid procedure can be easily scaled up to produce large numbers of organoids in a reproducible and robust manner opening the door to the use of this organoid technology for regenerative medicine applications that have not been realized yet such as transplantation into large animal models. In the revised manuscript we have now better described the scale up potential of our methodology.

<u>R#2: 7)</u> *Line 85: The authors start with hPSC-PIM-committed cell monolayers but they do not indicate what they did to commit the cells to PIM? It would be important for the authors to state how the hPSCs were maintained in culture and how many cells were placed in monolayer. The authors should talk about the monolayer differentiation phase so that the Results section can be more clearly related to Figure 1a and b.*

A: We thank the expert for yet another insightful remark. We now include further detail how hPSCs-PIM cells are derived in the main text as well as the cell density used for the generation of hPSC-PIM committed cells. This information was included in the Method section in the previous version of the manuscript. We totally agree that adding this information better reflects how experiments were assessed so that the Results section can be more clearly related to the corresponding **Figure 1a,b.**

<u>R#2: 8)</u> *Line 133 and the associated paragraph: The authors comment on the increased levels of metallothioneins in tubular-like cells of kidney organoids derived from 0.5-PIM compared to 250-Ks and in podocyte-like cells. It is not stated what the significance of this is?*

A: We thank the reviewer for this remark. Metallothioneins (MTs) are ubiquitous and highly conserved, cysteine-rich, low molecular weight metal-binding proteins. In the kidney, immunocytochemical studies have demonstrated MT are confined exclusively to the proximal tubules. MTs are the major protein thiol within the cell and with a crucial role in the metabolism, transport, homeostasis and toxicity or detoxification of certain metals. It has also been demonstrated that MT induction can offer cytoprotection against toxic metals and oxidative stress. Into the light of our findings that PT from 0.5 K organoids exhibit higher amounts of proximal-like tubular cells (LTL+) we now stress this finding and discuss about the biological significance of it as further refinement of our differentiation pipeline may represent a good alternative when aiming to develop functional assays for the tubular compartment. In this regard, it has been largely discussed that the embryonic nature of kidney organoids derived from human pluripotent stem cells largely impacts on the immaturity of the resulting nephron-like structures, which lack expression of important markers of the different tubular segments and thus hamper their use for functional testing.
Similarly, and following the reviewer comment we now offer a further discussion on the identified genes differentially expressed in podocyte-like cells comparing 0.5 vs 250 K kidney organoids. In this regard, we now briefly comment on the biological significance to detect higher expression of Podocin (NPHS2) together with other core components of the slit-diaphragm-associated network in podocyte-like cells of 0.5 vs 250 K kidney organoids, as the integral membrane protein 2B (ITM2B).

R#2: 9) *Suppl figure 1, panel c: The authors state that there is staining with ureteric bud markers without stating which of the markers they are referring to. GATA3 is a ureteric bud marker but does not indicate ureteric bud derivation. It is expressed in the mesangial cell and distal tubules of healthy human kidneys (Grigorieva et al JASN 2019) which are not derived from ureteric bud. Do the authors have indication that there are any anterior intermediate mesoderm markers along with PIM during organoid development using their protocols?*

A: We thank the reviewer for this remark. We fully agree with her/his comment that GATA3 expression does not indicate ureteric bud derivation and apologize for our statement that has been corrected by removing the sentence in the main text as well as the corresponding panel in Supplementary Figure 3 showing GATA3 expression in D16 kidney organoids. In relation to the second part of the comment of the expert, we would like to add that our previous work has shown that our differentiation pipeline sustains for the generation of anterior intermediate mesoderm (AIM) cells in a transient manner (as expected since our differentiation protocol sustains for the generation of PIM). Here we have also shown that all tested conditions led to the expression of markers of AIM by RT-qPCR (PAX2 and LHX1 at D4; GDNF and RET at D2, D5). These results were already shown in the previous version of the manuscript (**Supplementary Figure 1c in the manuscript**). We would like to share findings from the laboratory (non-published data; manuscript under preparation) which indicates that our differentiation pipeline sustains for the generation of GATA3 positive cells via the generation of a reporter cell line (***Figure 15 a-e*** **in this rebuttal letter)** which upon differentiation towards kidney organoids allows to monitor mOrange expression as a surrogate of GATA3 expression (***Figure 15 d,e*** **in this rebuttal letter**).

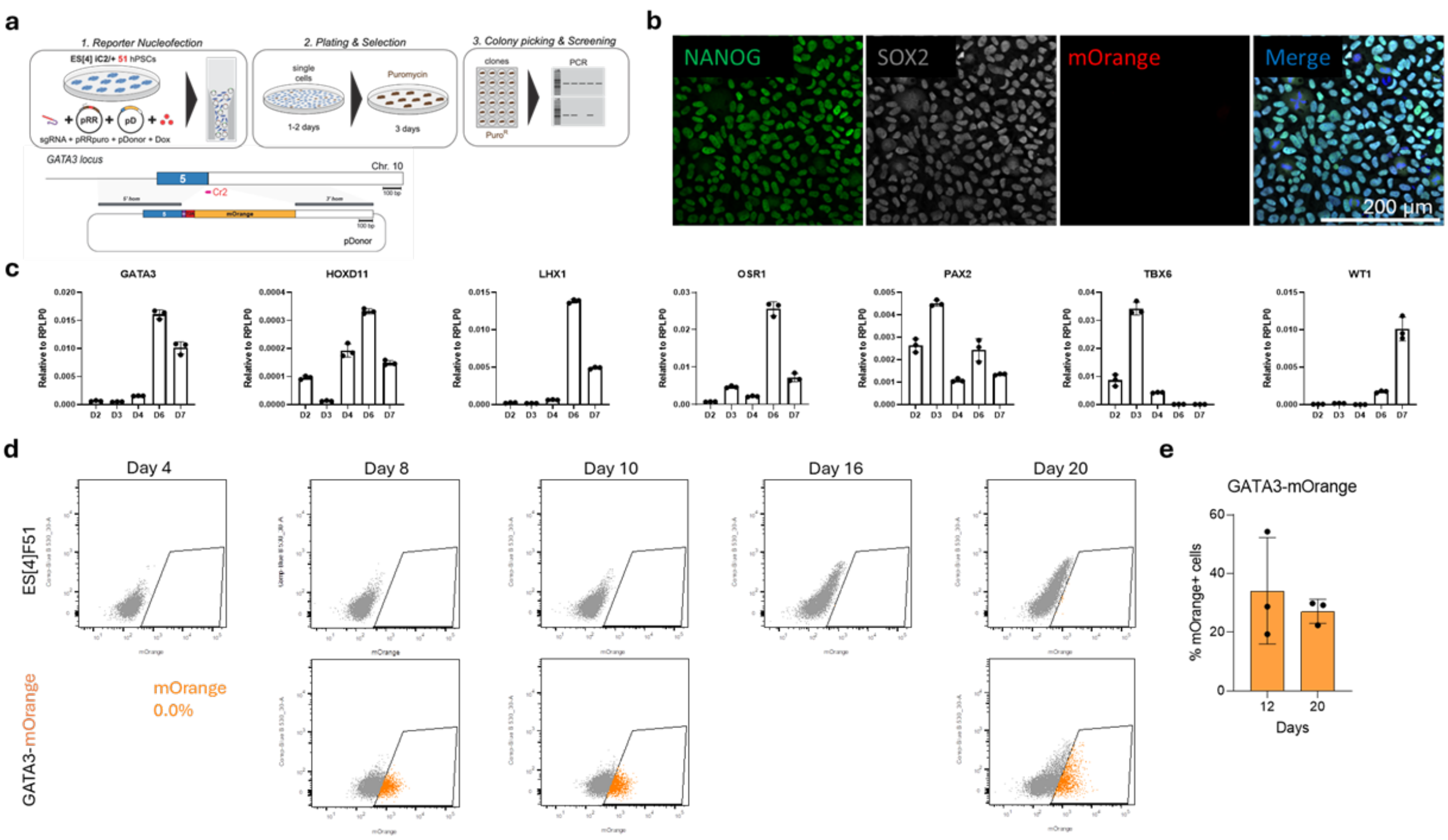

Figure 15. *Generation and validation of ES[4] F51 iC2 GATA3-mOrange reporter cell line.* ***a.*** *On the top, the schematic workflow used for the generation of the reporter cell line. On the bottom, the representation of GATA3 gene locus and the pDonor plasmid used to introduce the coding sequence of the fluorophore mOrange.* ***b.*** *Staining with the pluripotency markers NANOG and SOX2 and the reporter marker mOrange. Scale bar of 200 µm. Images are representative of n=3 technical replicates.* ***c.*** *RT-qPCR of the first 2-7 days of differentiation with*

GATA3-mOrange reporter cell line. The graphs are representative of n= 1 experiments with three technical replicates. ***d.*** *FACS timecourse of the endogenous expression of mOrange fluorophore (on the bottom) compared to the ES[4] F51 control cell line (on the top). Data were obtained with a pool of n= 12 (100 - Ks) organoids and the experiment was repeated n= 2 times.* ***e.*** *Quantification of the cells positive for mOrange at day 12 and 20 of differentiation after FACS sorting. Data are mean ± s.d. n= 12 (100 - Ks) organoids and the experiment was repeated n= 3 times.*

R#2: 10) *Suppl Figure 1, panels e and f. It appears that nephrin is highly expressed at day 16 in 0.5k and 8k conditions. This is not reflected in the quantitation in panel c. The authors should add quantitation on nephrin. In panel f, it is not possible to evaluate the grey PODXL staining.*

A: We would like to thank the expert for another important remark. We have now quantified the images relative to NEPHRIN detection in Supplementary Figure 1 (***Figure 16*** **in this rebuttal letter**). Although the statistical analysis shows no significant differences across the different experimental conditions there is a tendency of a higher NEPHRIN abundance in 0.5 K and 8 K kidney organoids compared to 100K and 250K counterparts. We have also performed a quantification of PODXL abundance observing a significant statistical difference across experimental conditions being more abundant in 0.5K compared to 100k kidney organoids for one of the hPSCs lines used in the manuscript. Due to the scarcity of biological replicates for the realization of these quantifications and since these experiments were included in **Supplementary Figure 1e-g** and **Supplementary Figure 2c-e in the manuscript** we present the results **in this rebuttal letter** but not in the revised manuscript.

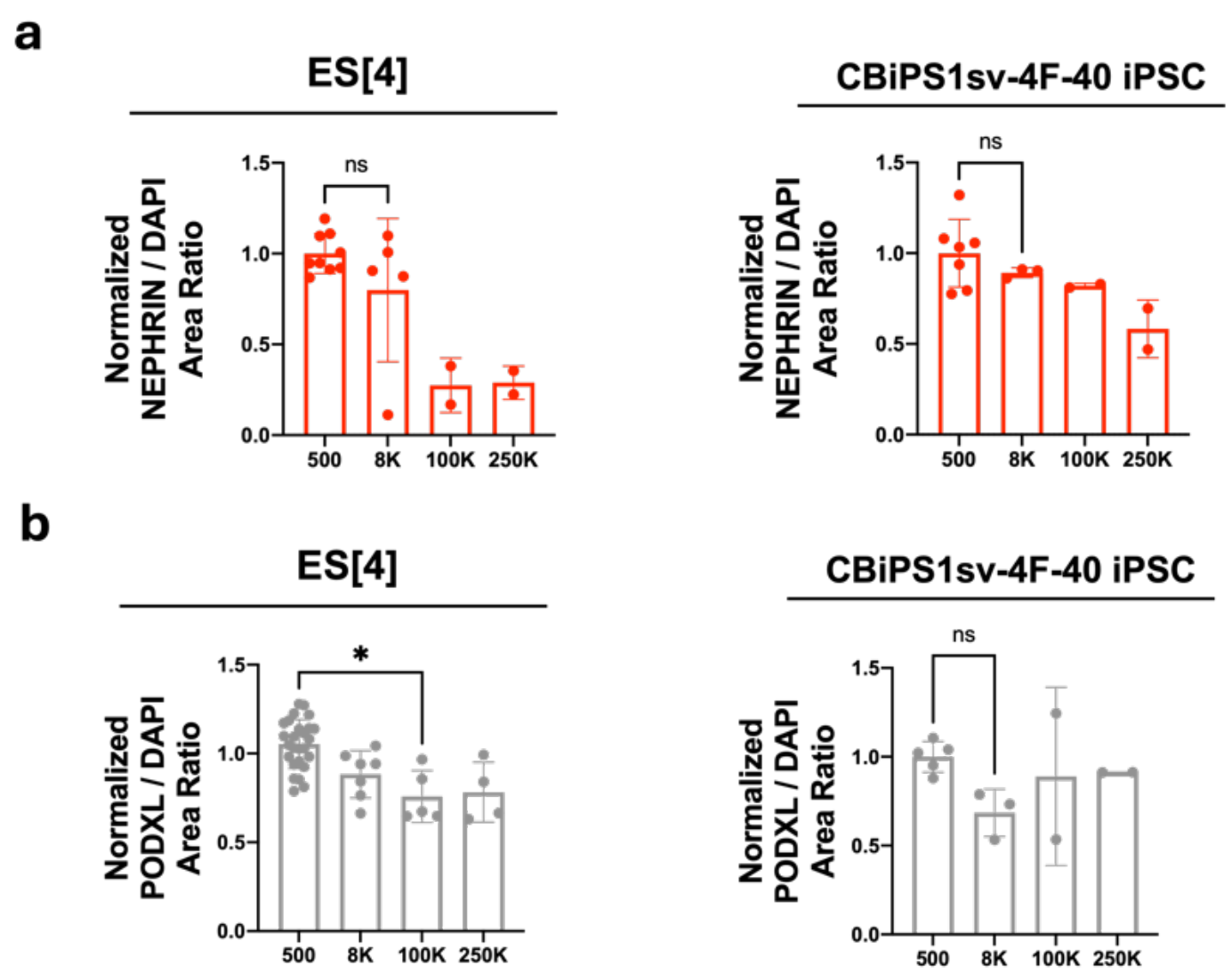

Figure 16. ***a***, *Quantification of normalized NEPHRIN area relative to DAPI in 0.5-, 8-, 100-, 250-Ks organoids at day 16 of differentiation. Data are mean ± s.d. n = 9 (0.5-Ks), n = 5 (8-Ks), n = 2 (100-Ks), n = 2 (250-Ks) for organoids derived from the ES[4] line. Data are mean ± s.d. n = 7 (0.5-Ks), n = 3 (8-Ks), n = 2 (100-Ks), n = 2 (250-Ks) for organoids derived from the CBiPS1sv-4F-40 iPSC line. ns, no statistical significance. Kruskal-Wallis (two-sided) followed by Dunn's comparison test.* ***b***, *Quantification of normalized PODXL area relative to DAPI in 0.5-, 8-, 100-, 250-Ks organoids at day 16 of differentiation. Data are mean ± s.d. n = 29 (0.5-Ks), n = 7 (8-Ks), n = 5 (100-Ks), n = 5 (250-Ks) organoids derived from the ES[4] line. Data are mean ± s.d. n = 5 (0.5-Ks), n = 3 (8-Ks), n = 2 (100-Ks), n = 2 (250-Ks) for organoids derived from the CBiPS1sv-4F-40 iPSC line. Kruskal-Wallis (two-sided) followed by Dunn's multiple comparisons test.*

R#2: 11) *Suppl Figure 2. Cannot distinguish the LTL or PODXL depicted in grey.*

A: We thank the expert comment. We would like to highlight that the decision on which colors to use when realizing confocal microscopy analysis for the detection of three different markers is based on previously established analysis pipelines in the laboratory. When combining red and green colors for the visualization of 2 out of the 3 markers, we have always used white or grey for the third marker of interest to ascertain the proper visualization of the three markers of interest in the same image (shown as merge by combining the three different channels in one single image). We present here an alternative color combination with green, red and yellow (color combination #2) instead of using grays (color combination #1), so that we can convince the expert that using grays represents the best option to depict this marker combination (***Figure 17*** **of this rebuttal letter**).

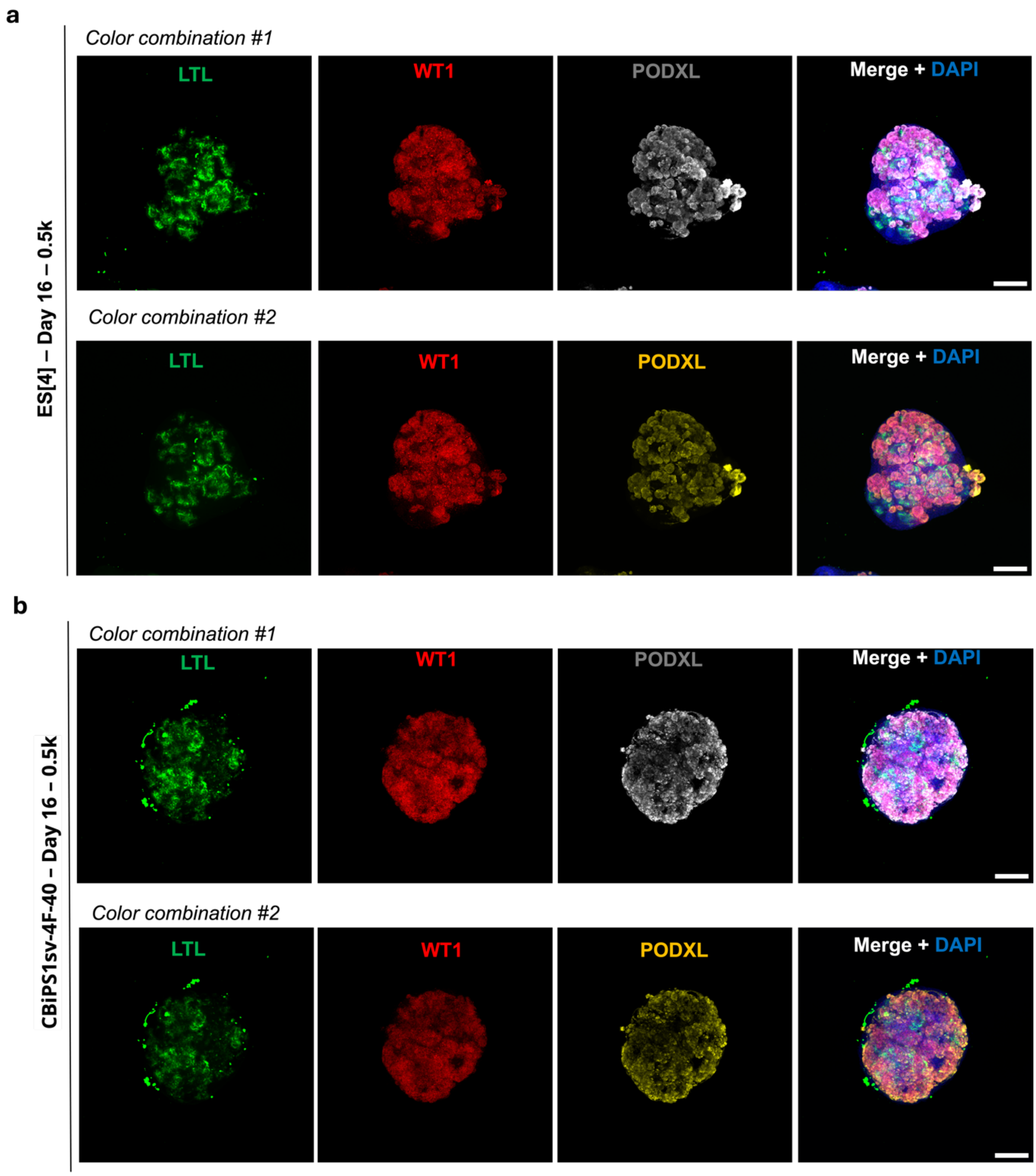

Figure 17. *Representative immunofluorescence staining for LTL (green), WT1 (red), PODXL (yellow) in day 16 kidney organoids derived from 0.5 Ks PIM-committed spheroids derived from ES[4] and CBiPS1sv-4F-40 hPSC lines. Rigth panels correspond to the merge of all the individual images and DAPI (blue). Scale bars, 200 µm.*

R#2: 12) *Suppl Figure 3a. I am surprised to see little variation in expression of proximal tubule genes for the 4 conditions. This implies that the genes are either expressed or not without any influence of the cell density at the start. Clearly there are a number of proximal tubule genes that are not expressed in any of the 4 conditions. If this is related to their stage of differentiation then it suggests that the 4 densities do not influence differentiation state of the proximal tubules but rather only the number of them.*

A: We thank the expert's comment. Indeed this observation is very relevant, as many other laboratories have shown on the possibility to generate kidney organoids by assembling 250 K or 500 K nephron progenitor-like cells [overall, the different works, including seminal works for the generation of kidney organoids from human pluripotent stem cells, have shown on the possibility to generate nephron progenitor-like cells in 2D cell culture setting through the exposure of undifferentiated hPSCs to Wnt agonists (CHIR, BMPs), RA agonists (Activin A), and FGF9 for 7 to 10 days] and using different cell culture formats (transwell system, Matrigel sandwich system, free floating conditions, among others). Our findings agree with those previous works and with our own previous published data showing that hPSCs-derived PIM cells after 4 days in culture can be assembled onto 8 K and 100 K spheroids to derive kidney organoids contain tubular-like cells, as expected (PMID: 32333836; PMID:35561674; PMID: 33301705).

Here, we show that 0.5 K kidney organoids exhibit a higher number of tubular-like cells (LTL+) compared to 250 K kidney organoids counterparts making use of confocal microscopy analysis and image analysis quantification (**Fig. 1i,j in the manuscript**). During this revision period and to reply to the expert question we have also evaluated the abundancy of LTL+ by FACS. Our results show that 0.5 K kidney organoids contain more LTL+ than 250 K kidney organoid counterparts (***Figure 18*** **in this rebuttal letter** and **new panel k in Figure 1** and **Supplementary Figure 3e,f in the new version of the manuscript,** respectively).

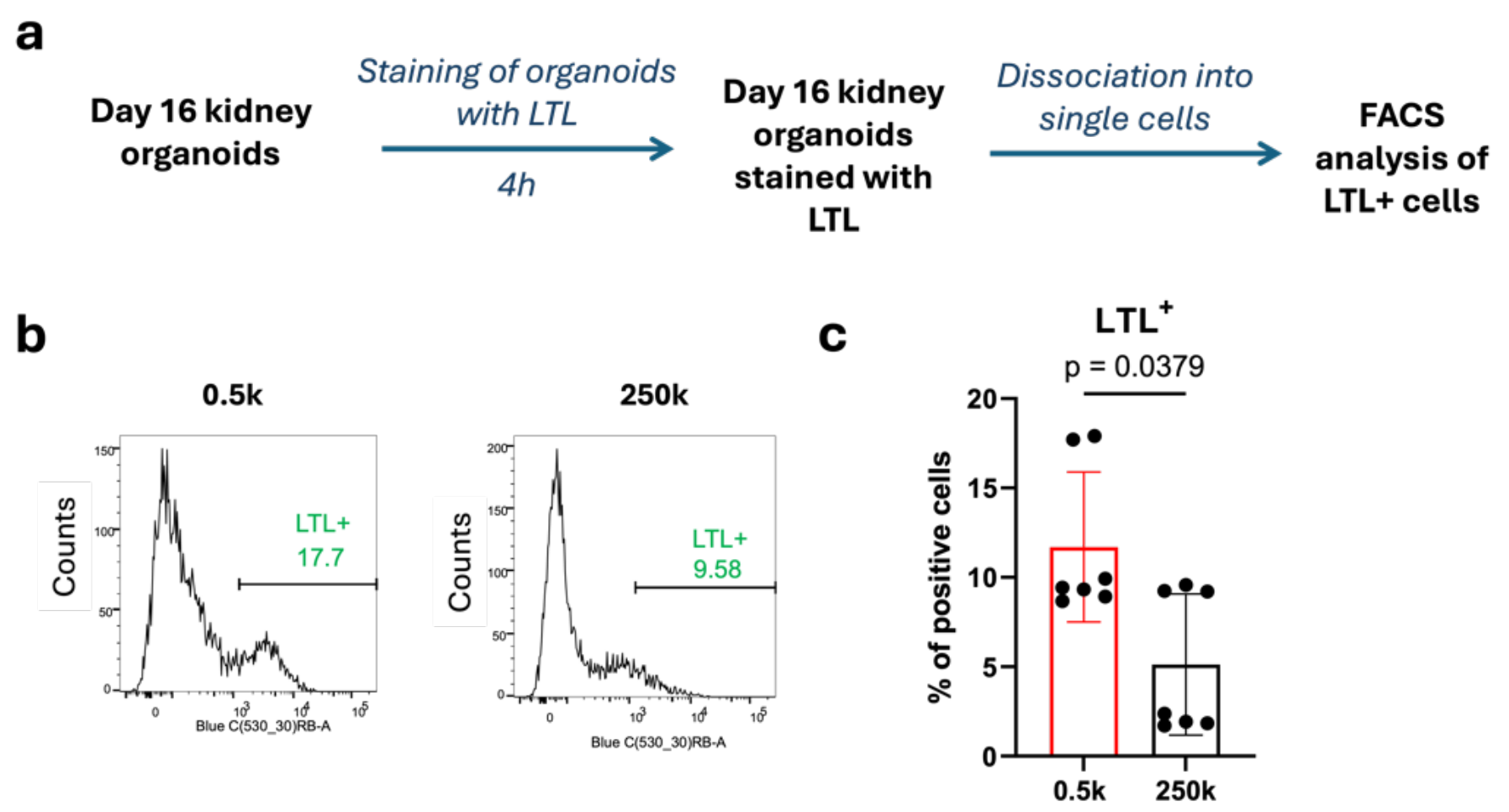

Figure 18. ***a***, *Kidney organoids were stained with LTL (fluorescein-conjugated LTL, FL-1321, Vector Laboratories) to label putative proximal tubule cells, dissociated to single cells and analyzed by flow cytometry (FACS).* ***b***, *Representative histograms representing the overlay of live cells and LTL+ cells from seven independent cell sorting experiments.* ***c***, *Quantification of the % of LTL+ proximal tubule cells in 0.5k and 250k kidney organoids by FACS analysis. Data are mean ± s.d. n = 7 biological replicates from a pool of 12 organoids each. Mann-Whitney test.*

<u>R#2: 13)</u> *Suppl Figure 3d. Not sure what can conclude from GATA3 staining other than there is no difference?*

A: We thank the reviewer for this remark. Following comment **R#2.9** from the expert we have removed this panel from the current version of the manuscript.

<u>R#2: 14)</u> *Figure 2 f,h and Suppl Figure 7d and f. Fractional excretion of Na and creatinine should be a %. Not clear how to interpret these plots.*

A: We thank the reviewer for this remark. We show in ***<u>Figure 19</u>*** **in this rebuttal** letter the correct representation of the requested parameters. We made a mistake when representing Fractional excretion of Na because we did use creatinine values in mmol/L and not in micromol/L. We now have represented the calculation as %. We apologize for this and thank the expert for this important remark. With respect to creatinine determinations, we have made a mistake when naming the plots. The plots that were named Fractional excretion of creatinine are in fact Creatinine clearance relative to 100g of renal weight (ml/min/100g). This mistake has been corrected in the **revised manuscript** and corrected in the following figures: **Supplementary Fig. 5g, Supplementary Fig. 6f, Supplementary Fig. 7j** and **Supplementary Fig. 8e**. We apologize for this and thank the expert for this important remark.

a

$$\text{FE Na}^{+}\,(\%) = \left(\frac{U_{Na}\, x\, P_{creatinine}}{P_{Na}\, x\, U_{creatinine}}\right) x\ 100$$

U_{Na} : urinary sodium (mmol/L)
$P_{creatinine}$: perfusate creatinine (μmol/L)
P_{Na} : perfusate sodium (mmol/L)
$U_{creatinine}$: urinary creatinine (μmol/L)

b

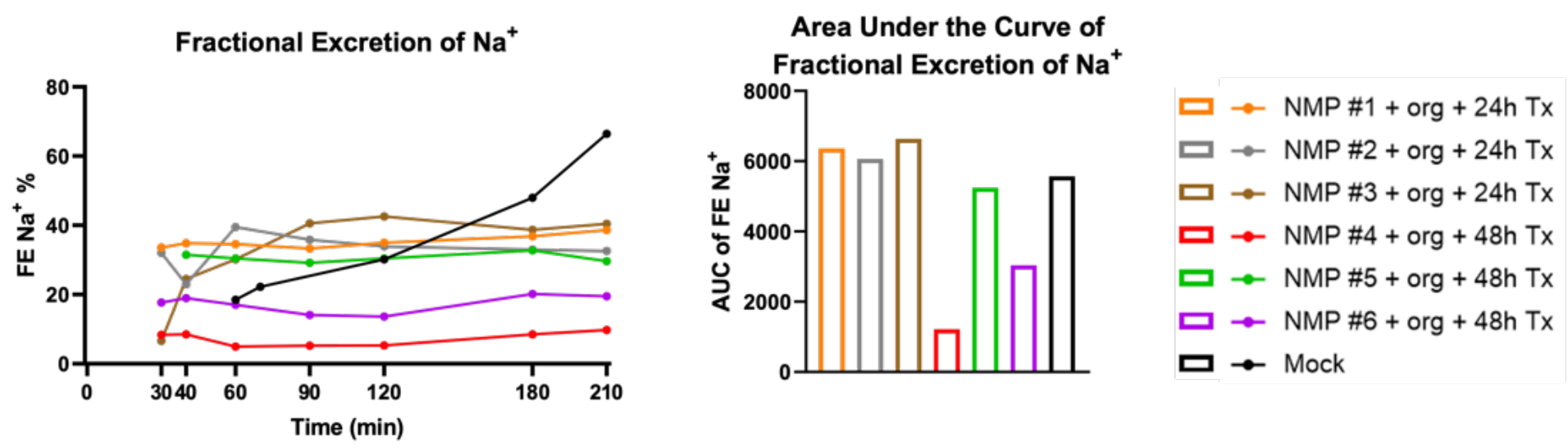

***<u>Figure 19</u>**. **a**, Formulation for the calculation of Fractional excretion of Na (FE Na^+). **b**, Representation of FE Na^+ (%) and area under the curve of FE Na^+ in all the experimental conditions.*

<u>R#2: 15)</u> *Suppl Fig 7p & q would seem to be important data for the main body figures since they data highlight renal function before and after transplantation of kidneys perfused with organoids. However, only 2 animals were used. One experiment ended at 24h and one ended at 48h, with urine and blood collections at various timepoints. Why were both animals not monitored for at least 48h? The values of the 2 animals seem similar. The biochemical analyses performed on these healthy animals prior to surgery and post-transplant doesn't clarify the effects of organoid engraftment in the transplanted kidney.*

A: We thank the expert for this important remark. We do also agree that **Suppl Fig 7-9** capture important information for our work. We have however organized the Figures in this new version of the manuscript also following the Editor recommendation to highlight the procedure for organoid generation. Importantly, and as also suggested by the expert we now include results from experiments

performed during the revision process including experiments on the infusion of kidney organoids during NMP and autotransplantation during 24 h and 48 h. Also following the expert comment and the considerations from **R#1** and the Editor we have made use of these new experiments to explore on potential immune responses until 48 h post-transplant. The reason to choose this period is due to several factors: i) the ethical clearance of our project was subjected to experiments performed within a time of 48 h; ii) xenogeneic immune responses have been shown to occur within minutes or hours after transplantation and lead to hyperacute rejection and graft destruction within 24 hours (PMID: 35967435).

With regards to the comment of the expert on the lack of clarity of the biochemical analysis when exploring potential effects related to engraftment, we are also in agreement with this comment. For this reason, we have made use of different techniques to further explore on potential side effects of cell engraftment such as the detection of markers related to local immune response through immunohistochemistry and image analysis quantification for CD15, CD68 and CD45. These analyses are now shown in the new **Figure 4 in the manuscript** and on ***Figure 9*** **of this rebuttal letter** (*please see* our response **R#1.2b)**.

R#2: 15a) *Why is the animal blood urea increased 4-fold within 18 hr of transplantation? Why does serum creatinine increase by 50% with the transplant?*

The increase in urea and creatinine after renal autotransplantation in pigs, as in other animal models and in humans, can be attributed to several factors related to the surgical procedure and the physiological response of the organism. Some of the factors are listed here:

1.Ischemia-Reperfusion:it has been largely described that during transplantation, the kidney is deprived of blood supply, causing ischemia damaging kidney cells. Also, that during reperfusion the restoration of blood flow can cause additional damage due to the production of free radicals and an inflammatory response. Overall, the ischemic-reperfusion damage compromises kidney function and leads to increased blood creatinine and urea.

2.Inflammatory Response: surgery and tissue damage can trigger an inflammatory response that can impair kidney function. The release of inflammatory cytokines and other mediators can contribute to kidney dysfunction.

3. Acute Tubular Necrosis: damage caused by ischemia-reperfusion can lead to acute tubular necrosis (ATN), a condition where renal tubular cells die, affecting the kidney's ability to filter waste efficiently. ATN is indeed a common cause of postoperative acute kidney failure.

4. Hemodynamic Effects: changes in blood pressure and renal blood flow during and after surgery can negatively impact kidney function. Intraoperative or postoperative hypotension can reduce renal blood flow and glomerular filtration.

5. Surgical Injury: manipulation of the kidney during surgery can cause direct damage to kidney tissue. Sutures and blood vessel anastomoses can cause microtraumas that affect kidney function.

During the revision process we have scrutinized for some of the factors cited above and we at the present time cannot disregard potential causes leading to the observed increase in urea and creatinine, as for example: acute tubular necrosis or hemodynamic effects or surgical injury.

R#2: 15b) *Would be good to have data comparing kidneys+organoids vs kidneys-organoids post-transplant?*

A: We thank the expert for this important remark. In ***Figure 20*** **in this rebuttal letter,** we present the requested data. The results highlight similar values in both experimental conditions and suggest no relationship between the observed values for creatinine and urine and the infusion of kidney organoids during NMP and autotransplantation.

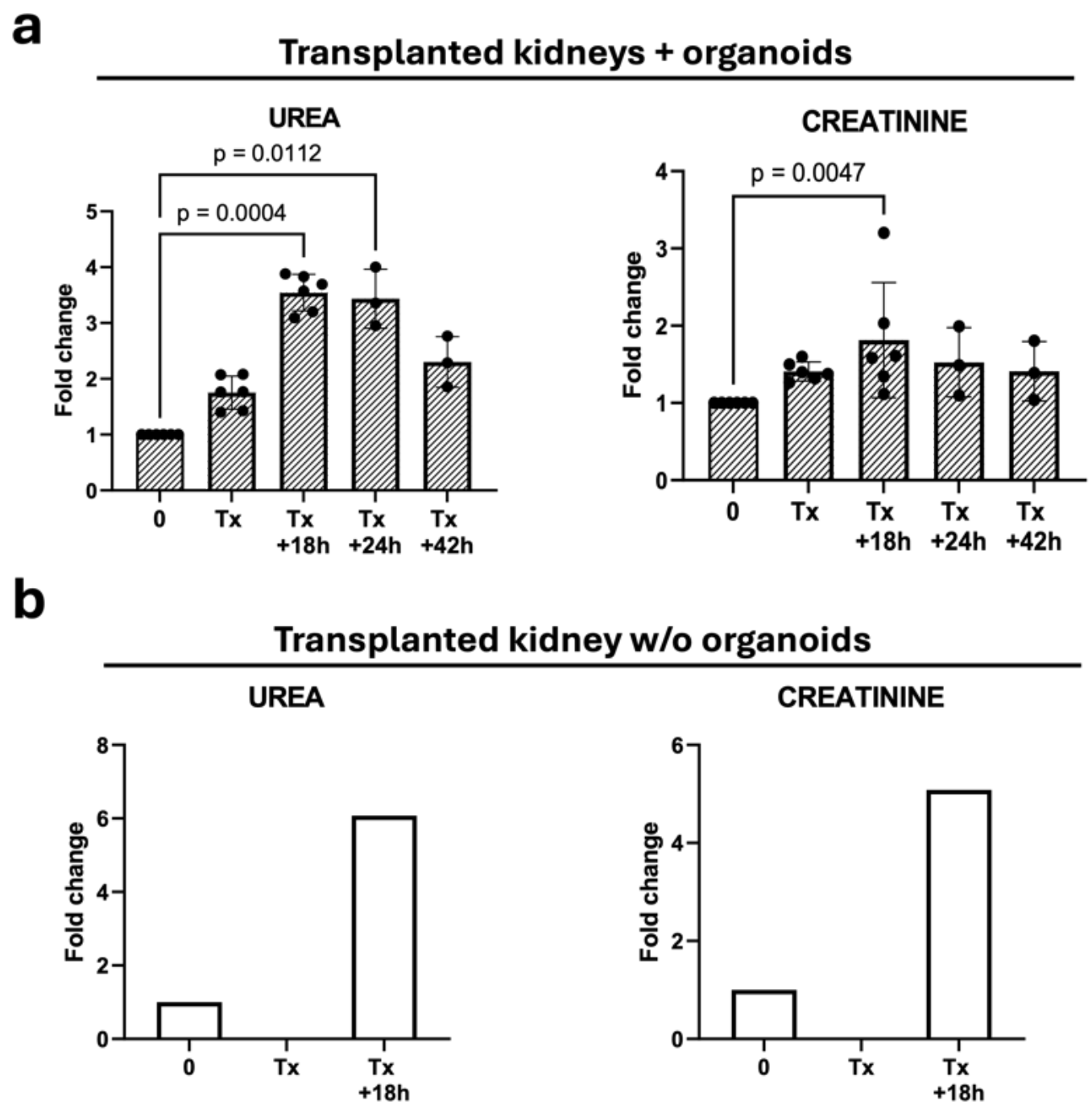

***Figure 20**. **a,** Urea and creatinine analysis performed in serum samples from recipient animals before (t0, basal) and after transplantation (Tx). Data are represented as fold change mean± s.d. n= 3 animals transplanted with organoid-treated kidneys for 24 h and n= 3 animals transplanted with organoid-treated kidneys for 48 h. Kruskal-Wallis (two-sided) followed by Dunn's multiple comparisons test. P values for statistical significant comparisons are shown. **b,** Urea and creatinine analysis performed in serum samples from recipient animals before (t0, basal) and after transplantation (Tx). Data are represented as fold change mean± s.d. n= 1 animal transplanted with a kidney without organoids (Mock) for 24 h.*

R#2: 16) *Figure 2k. It appears that most of the Alu repeats in the porcine cortex are in glomerular regions which is predictable since the glomerular capillaries would trap them. What were the size of the organoids infused?*

A: We would like to thank the reviewer for this comment. As highlighted by the expert, most of the Alu positive cells are encountered in glomerular regions. We would like to highlight that Alu positive cells are also found with less abundancy on tubular structures. With regards to the expert question, we would like to add that the overall size of the infused organoids is about 200 to 400 μm. We have prepared a single figure in **this rebuttal letter** (***Figure 21*** **in this rebuttal letter**) to show the different sizes of the generated organoids in two different cellular backgrounds. The individual panels used to prepare this figure are shown in **Supplementary Figures 1 and 2 in the manuscript.**

Importantly, we would like to highlight that experiments were conducted using either the infusion of intact organoids (**Figure 3** and **Supplementary Figure 7 in the manuscript)** as well as disaggregated organoids (**Supplementary Figure 6 in the manuscript**), and in both cases Alu positive cells were encountered in glomerular and tubular regions.

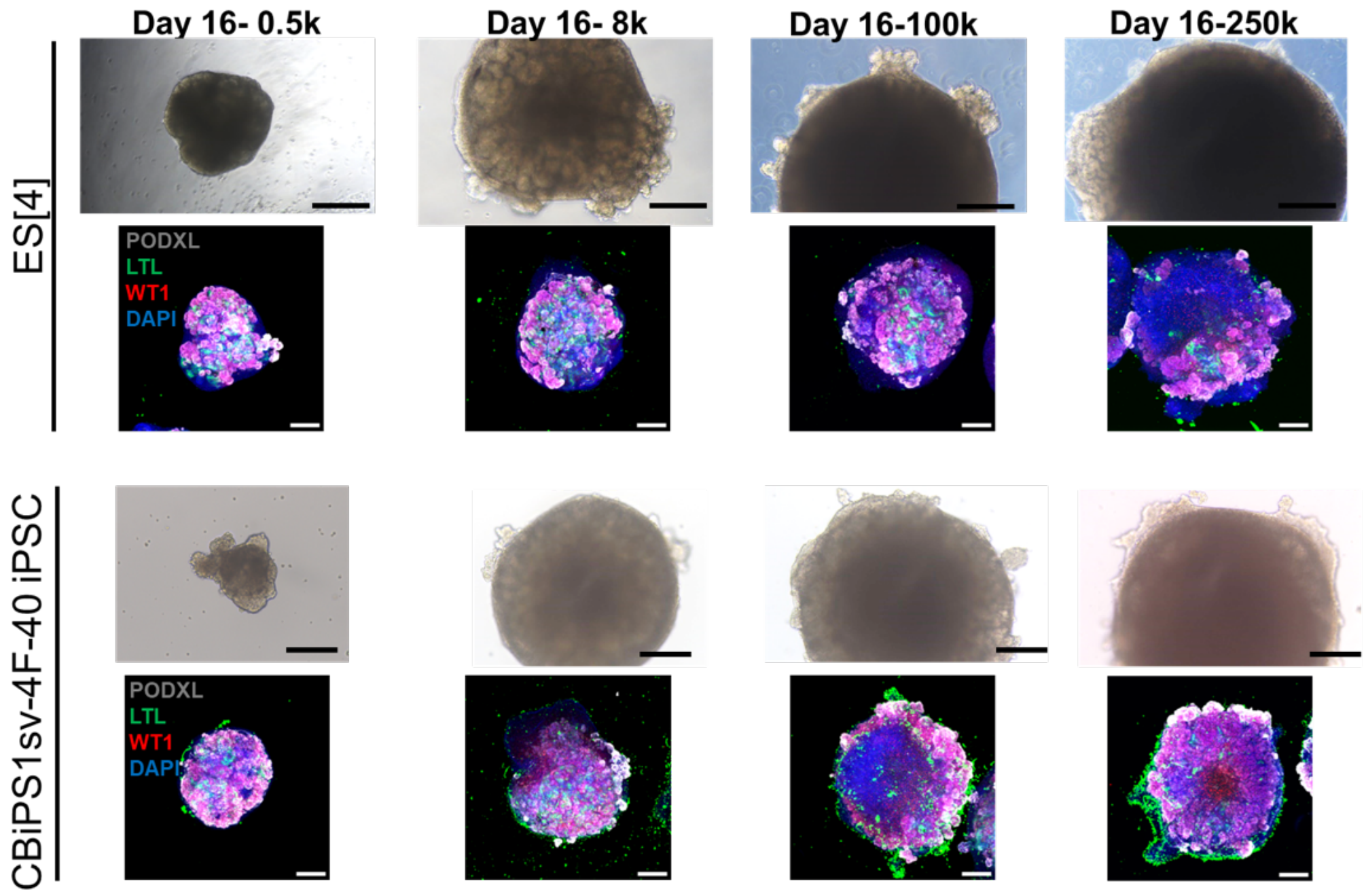

***Figure 21**. **a)** Representative bright-field images of day 16 kidney organoids derived from 0.5-, 8-, 100-, and 250-Ks IM-committed spheroids derived from the ES[4] line. Scale bars, 250 µm. **b)** Representative bright-field images of day 7 and day 16 CBiPS1sv-4F40 iPSC-derived kidney organoids generated from 0.5-, 8-, 100-, and 250-Ks IM-committed spheroids. Scale bars, 250 µm. **a,b** Bottom panels show representative immunofluorescence staining in day 16 kidney organoids derived from 0.5-, 8-, 100-, and 250-Ks IM-committed spheroids for the detection of LTL (green), WT1 (red), PODXL (grays) and DAPI (blue).*

R#2: 17) *Lines 231-235 discuss the studies demonstrating vascularization of organoids by host tissues, and how they aimed to "challenge our differentiation pipeline for therapeutic translation in kidney regeneration"; however, the authors provide no data to assess kidney regeneration or vascularization in the transplantation experiments?*

A: We would like to thank the reviewer for this observation. We have omitted this sentence in the corresponding section and only refer to the work performed in the manuscript.

Minor comments:

R#2: 18) *Suppl Figure 5f. Do not see the 100mm scale bars*

A: We would like to thank the reviewer for yet another remark. The scale bars were included in the center of the images, and they were difficult to distinguish. We now have included the scale bars on the right side of the image. Due to the inclusion of new data in the revised manuscript those panels are now found in **New Figure 2 in the manuscript**(**panel k**).

R#2: 19) *line 203: "In" rather than "Into"*

A: We would like to thank the reviewer for yet another remark. We have changed the text accordingly.

<u>R#2: 20)</u> *Suppl Figure 5h,k. It states in the figure legend: "Scale bars (l, m), 200 µm". I believe the authors mean panels (h,k).*

A: We would like to thank the reviewer for yet another remark. We have changed the text accordingly. Due to the inclusion of new data in the revised manuscript those panels are now found in **New Supplementary Fig. 4 (k,l) in the manuscript**.

Point-by-point response to the Reviewers' Comments:

Reviewer #1 (R#1)

R#1: 1) *Thanks to the authors for providing such comprehensive additional information in response to the suggestions provided in the first review. All in all, both the ambitious response and the extensive efforts to add more supporting information and substance to the study demonstrate the capability of the group of authors.*

Authors (A): We thank the reviewer for the very positive evaluation of our work and his/her constructive comments, which have allowed us to improve our manuscript during the revision process. We also would like to thank the expert for his/her insightful comments during the revision process which clearly helped us to improve our first submission resulting in the incorporation of new data and supportive information that now are incorporated as main figures in the final version of our work.

At the end of this rebuttal letter, we also include an **Annex** section that summarizes all the new data that has been generated during the review process which is now included in the revised version of the manuscript. The **Annex** also contains a summary of the experiments performed as well as all the additional analyses developed during this revision process.

R#1: 2) *One aspect that I suggest to clarify is the hypothesis of the organoid NMP/infusion experiments. It remains unclear, how "hPSC-derived organoids as an intervention for graft pre-treatment" should work. Is the idea to produce kidneys within kidneys? To add functional units within kidneys? How would the organoids integrate and add to the function of kidneys? Shall they extend an repair mechanism? Or - as xenografts in pig kidneys - modify the immune response?*

A: We appreciate the expert insightful comment and we agree that the potential use of organoid NMP/infusion needed further clarifications. In the revised version of the manuscript, we have emphasized the rationale behind organoid infusion during NMP in porcine kidneys and the potential applicability of this approach.
We here provide a more detailed explanation. In any case, we would agree to include this information in the manuscript under the expert's consideration.

In the near future, hPSC-kidney organoids could be produced on-demand in the clinical setting and used to (1) attempt to **repair and/or regain functional characteristics of damaged kidney cells** in three different settings.

1.A) **In patients with chronic kidney disease (CKD),** organoids could be introduced directly into diseased kidneys by catheterization. In vivo delivered organoids in mouse models has been shown to engraft into the host and differentiate into tubular and glomerular-like structures with maturity markers (PMID: **29429961**, **26586691**, **31303547**). Our therapeutic approach may be able to prolong the functional properties of end of stage kidneys and postpone/reduce replacement therapy with hemodialysis while waiting for a transplant.

1.B) **Before kidney transplantation**, it is possible that hPSC-kidney organoids may be able to add functional characteristics of donor kidneys that are normally discarded for organ transplantation. Biopsy findings of glomerulosclerosis is the most reported reason for discard (PMID: **29735310**)

and kidney organoids have been recently reported to ameliorate glomerulosclerosis measured by reduced proteinuria in a phase II clinical study. Therefore, our methodological approach may contribute to expanding the pool of organs available for transplantation.

1.C) **After kidney transplantation**, hPSC-kidney organoids could be used to repair damaged tissue during chronic allograft rejection. Chronic kidney rejection remains a major unresolved problem in the clinic and involves damage to both the graft parenchyma and blood vessels. Since replacement of damaged kidney tissue can be achieved with organoids, it is possible that our methodology may be able to extend the lifetime of the transplanted kidney (PMID: **35750295**).

In addition, hPSC-kidney organoids could be produced on-demand from a patient waiting for a transplant under living kidney donation to (2) **produce immune-compatible organotypic structures** in the donor kidney and reduce the recognition of foreign HLA molecules, which initiate the immune response during graft rejection (PMID: **39398250**).

Besides the importance of these potential applications, several technical considerations needed to be addressed, including the optimization of differentiation methods to generate *bona fide* hPSC-kidney organoids and evaluation of their in vivo safety in clinically relevant scenarios.
Our work is conceived for this purpose and to this, we first here establish a new procedure that defines a cost-effective method to reproducibly and scalable produce kidney organoids from hPSCs in large quantities with the advantage to contain different renal cell types. Then, we have developed a new procedure to infuse hPSCs kidney organoids during NMP into porcine kidneys ex vivo and proved the feasibility and the safety of this approach in vivo. In summary, we provide the first proof-of-principle that machine perfusion represents an accessible and safe platform to administer nephron-stage kidney organoid-based therapies in the future. Therefore, the tools developed here open the door to further studies aimed at understanding the potential functional benefit of hPSC-kidney organoids and favor the development of new therapeutic approaches for renal cell regeneration in the clinic.

In addition, we have taken the liberty to prepare a figure for a possible "*graphical abstract*" presenting the highlights of our study, including the *ex vivo* and *in vivo* platforms and the main readouts (**Graphical abstract figure in this rebuttal letter**). We will be happy to include this figure in the manuscript under the expert and editorial board consideration.

>Figure redacted<

<u>Graphical abstract figure</u>. We have defined a novel approach to differentiate human pluripotent stem cells (hPSCs) into hPSC-kidney organoids in a scalable and reproducible manner. Quantitative and qualitative analysis show that hPSC-kidney organoids exhibit transcriptional variety and cellular composition. To prove the therapeutic application of this new technology, hPSCs-kidney organoids have been infused into porcine kidneys *ex vivo* using a normothermic machine perfusion (NMP) and further validated *in vivo*. The safety of this new procedure has been demonstrated through constant monitoring and functional evaluation *ex vivo* and *in vivo*. Sample analysis shows kidney organoid engraftment after NMP *ex vivo* and *in vivo* and the absence of xenogeneic pig-mediated immune response against human organoids *in vivo*.

R#1: 3) *Further to this, it should be emphasized in the limitations of the study, that because the contralateral kidney was not removed in the transplant experiments, any functional analysis in the transplant model are limited because the native kidney remains intact and functioning.*

A: We appreciate the reviewers' comment and the limitations of the potential use of organoid NMP/infusion are now discussed in detail in the subsection "Limitations of the study".

R#1: 4) *Despite this fact, the dynamics of blood and urine analysis are surprising and difficult to understand. The display of each experiment individually instead of grouped outcomes, makes it*

hard to read and follow. Could you please clarify why you believe there is an increase in e.g. urea and creatinine

A: As suggested by the reviewer, we have now represented the blood and urine results in a grouped manner to provide a more comprehensive organization of the data (new **Supplementary Fig. 9b,c in the manuscript**). Specifically, we present the results regarding blood urea and creatinine levels in pigs reimplanted with organoid-treated kidneys (NMP + org) before engraftment (t0, basal) and during the postoperative follow up (**Figure 1a in this rebuttal letter** and **Supplementary Fig. 9b in the manuscript**), showing that blood urea and creatinine levels peaked at Tx+18h and subsequently approached basal levels at Tx+42h (**Figure 1a in this rebuttal letter**). In addition, we here present a comparison between blood urea and creatinine levels from pigs reimplanted with organoid-treated kidneys (n= 6) and those levels from an animal reimplanted with mock-treated kidney (without organoids) and contralateral nephrectomy (n=1) during the first 18 hours of postoperative follow up (**Figure 1b in this rebuttal letter**). This comparison shows that the observed fold change increase in urea and creatinine levels in animals with organoid treated-kidneys (NMP + org) is comparable to those with the mock-treated kidney (mock with nephrectomy; *n*= 1) (**Figure 1b in this rebuttal letter**), suggesting no relationship between the observed creatinine and urea values and the presence of kidney organoids.

The observed changes in the blood and urea results may be attributed to several factors related to the surgical procedure and the physiological response of the organism during the first hours of postoperative follow up (PMID: **35641829**). Some of these factors include: (1) ischemia-reperfusion injury, which leads to increased blood creatinine and urea; (2) surgical Injury, which leads to inflammation and secretion of DAMPs (damage-associated molecular patterns); and (3) hemodynamic effects due to Intraoperative or postoperative hypotension.

We have now included the description for these results together with the modified graphs representing the blood and urine biochemical data in a grouped manner (**Figure 1a** in this rebuttal letter and **Supplementary Fig. 9b,c in the manuscript**).

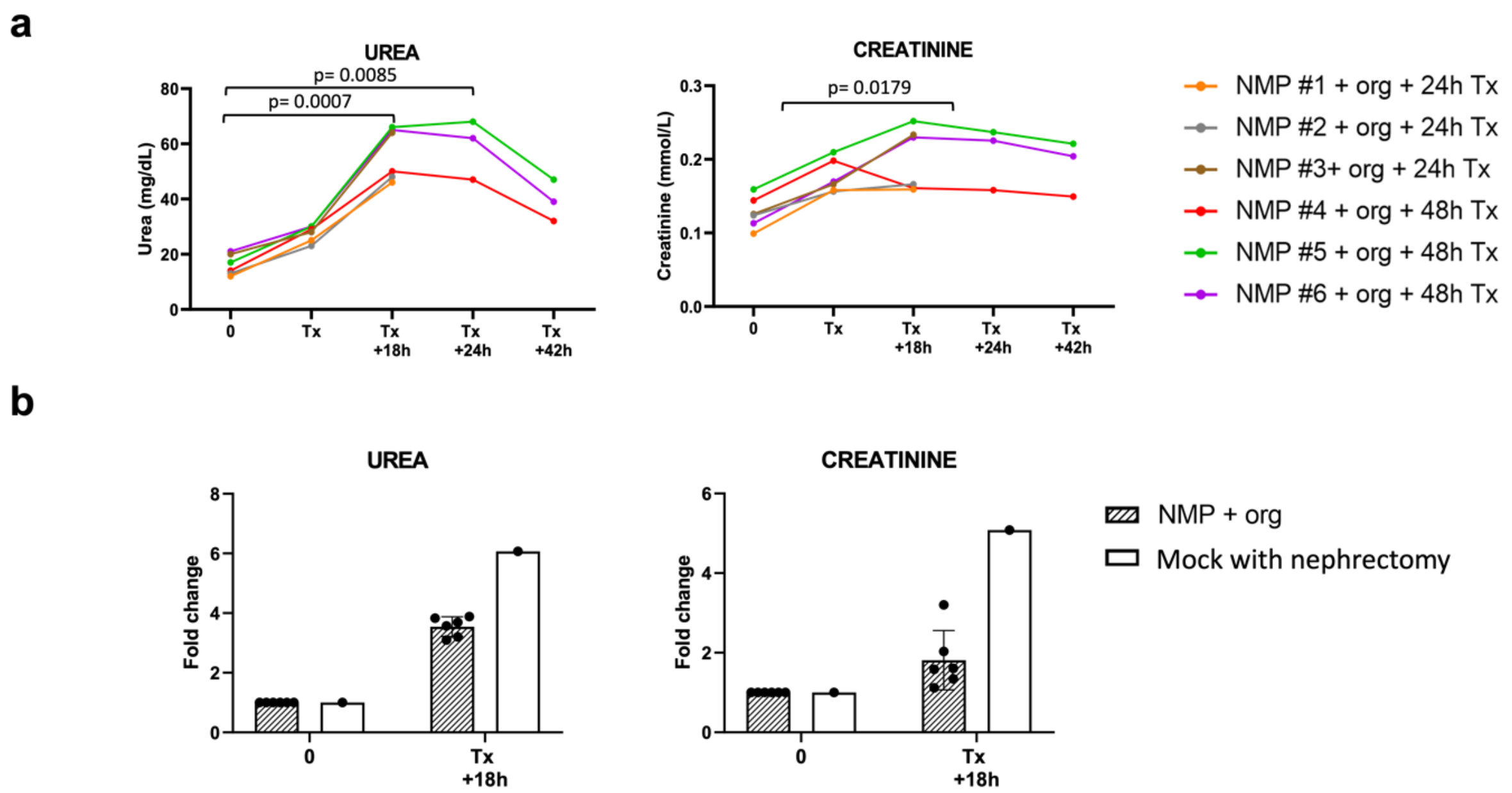

Figure 1. a) Blood urea and creatinine analysis performed in recipient animals before (t0, basal) and after transplantation (Tx). Data are mean ± s.d. *n*= 3 animals transplanted with organoid-treated kidneys for 24h (NMP #1, #2, #3 + org + 24h Tx) and *n*= 3 animals transplanted with organoid-treated kidneys for 48h (NMP

#4, #5, #6 + org + 48h Tx). Kruskal-Wallis (two-sided) followed by Dunn's multiple comparisons test. *p* values for statistically significant comparisons are shown. **b)** Blood urea and creatinine levels in recipient animals at Tx+18h of postoperative follow up relative to basal values (t0). Data are mean ± s.d. *n*= 3 animals transplanted with organoid-treated kidneys for 24h (NMP #1, #2, #3 + org + 24h Tx) compared to *n*= 1 animal transplanted with a kidney without organoids (Mock kidney with nephrectomy of the contralateral kidney).

R#1: 5) *Also, data for the 24 hour experiment are shown for the 18 hour time point and not 24 hours.*

A: We appreciate the expert remark. The time point of 18h corresponds to the precise time at which blood and urine samples were retrieved from animals during the postoperative follow up and before performing surgery to collect the kidney grafts in the 24h experiments. Similarly, in the 48h experiments, the time point of 42h corresponds to the precise time at which blood and urine samples were retrieved from animals during the postoperative follow up and before performing surgery to collect the kidney grafts.

Reviewer #2 (R#2)

R#2: 1) *The authors have responded to each of my concerns. The effects on the functional aspects of the transplanted kidney related to organoid infusion remain limited*.

A: We thank the reviewer for his /her positive evaluation of our work.

In the present study we evaluated the feasibility and safety of hPSC-kidney organoid infusion during NMP and after *in vivo* engraftment.

Our results demonstrate that the delivery of hPSC-kidney organoids during NMP in porcine kidneys do not have major effects on systemic metabolic parameters or macroscopic morphology of kidneys during NMP or early *in vivo* engraftment (24 or 48h), highlighting that the procedure established here is feasible and safe. We show that during the assayed time window human organoids engraft in the host renal tissue, remain viable, and do not elicit an immunogenic response. Importantly, the conceptual insights and technological developments provided here describe a technological platform, which opens the door to further investigations aimed at understanding the therapeutic potential of hPSC-derived kidney organoids in clinical studies. We now provide additional discussion of the limitations of this study in the revised version of the manuscript.

At the end of this rebuttal letter, we also include an **Annex** section that summarizes all the new data that has been generated during the review process which is now included in the revised version of the manuscript. The **Annex** also contains a summary of the experiments performed as well as all the additional analyses developed during this revision process.

ANNEX

Contents

1. ***New panels included in the main Figures of the manuscript.***
2. ***New panels included in the Supplementary figures of the manuscript.***
3. ***Summary of all the NMP and in vivo experiments conducted in the manuscript (Table 1).***
4. ***Qualitative assessment of potential tissue damage in biopsies from NMP and in vivo experiments by hematoxylin-eosin staining and pathological evaluation (Table 2).***

1. New panels included in the main Figures of the manuscript

1.1. Panels **a,b,c** presented in ***(Annex) Figure 1*** are included in **Figure 1k and Supplementary Fig.3e,f in the manuscript**:

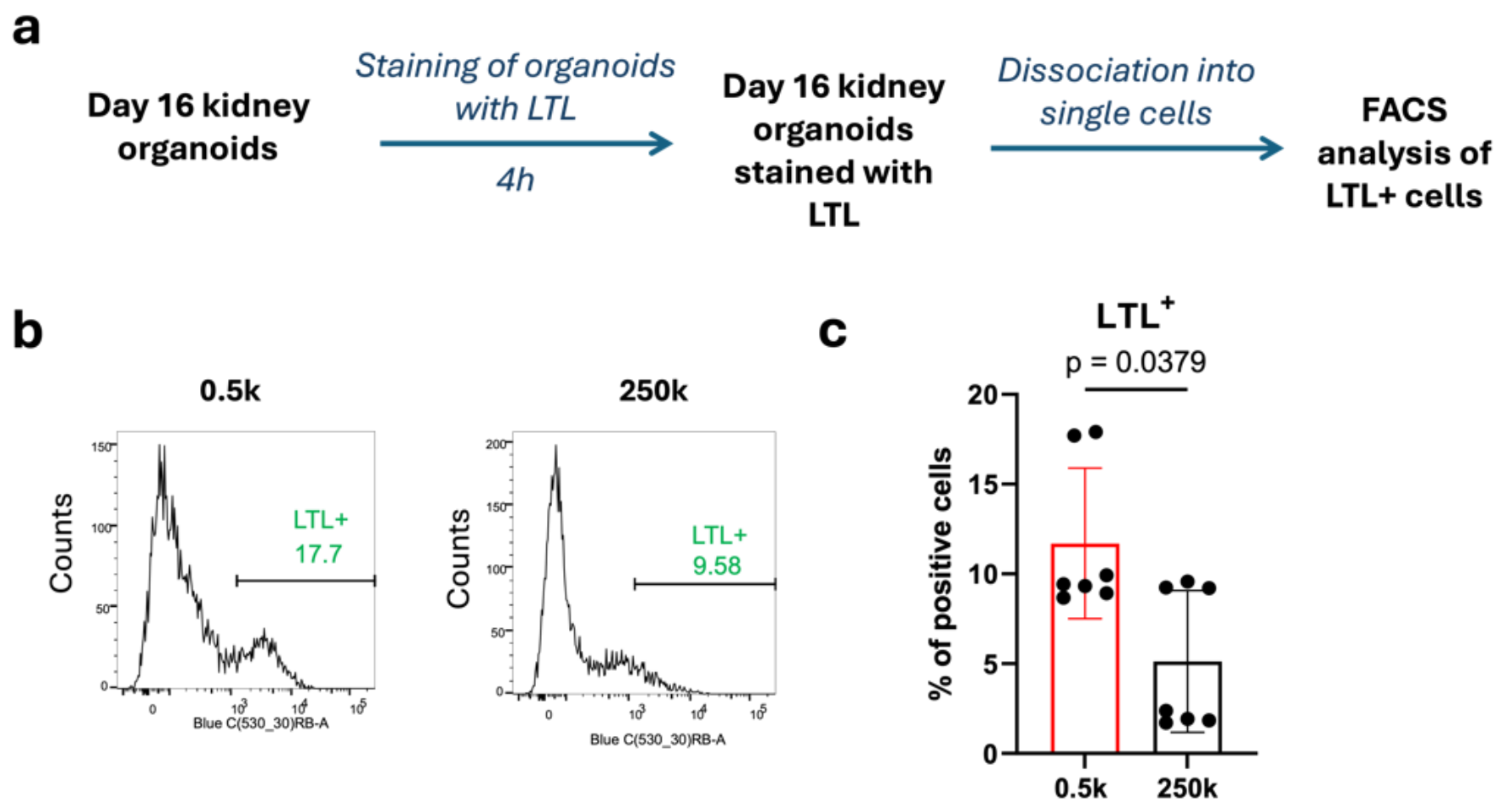

(Annex) Figure 1. ***a,*** *Kidney organoids were stained with LTL (fluorescein-conjugated LTL, FL-1321, Vector Laboratories) to label putative proximal tubule cells, dissociated to single cells and analyzed by flow cytometry (FACS).* ***b,*** *Representative histograms representing the overlay of live cells and LTL+ cells from seven independent cell sorting experiments.* ***c,*** *Quantification of the % of LTL+ proximal tubule cells in 0.5k and 250k kidney organoids by FACS analysis. Data are mean ± s.d. n = 7 biological replicates from a pool of 12 organoids each. Mann-Whitney test.*

1.2. Panels **b,c,f,g,h,i** that are shown in ***(Annex) Figure 2*** correspond to the new data included in **Figure 3 of the manuscript:**

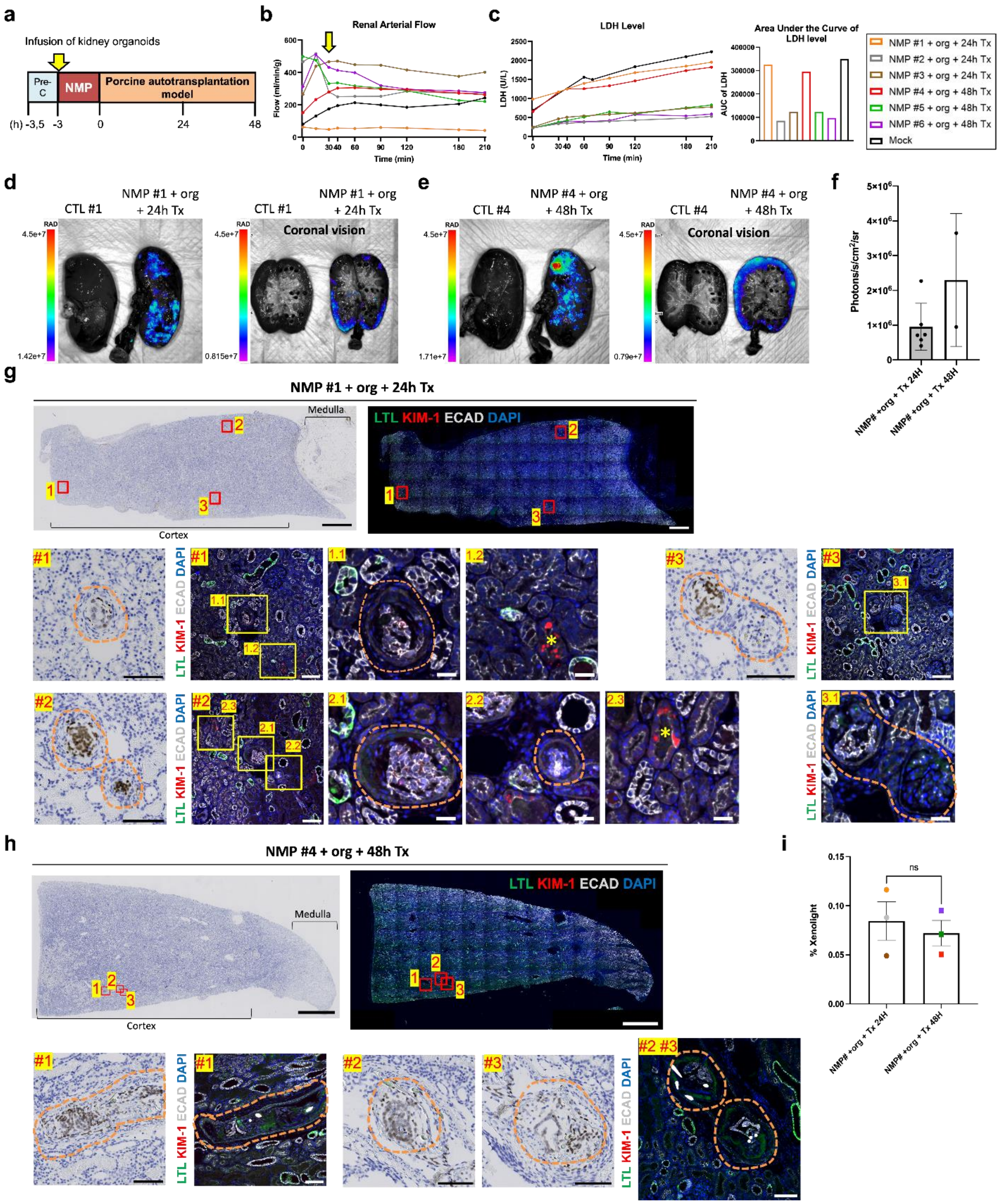

(Annex) Figure 2. ***In vivo engraftment of porcine kidneys infused with human kidney organoids. a,*** *Experimental scheme for infusion of Kidney organoids in pig kidneys during NMP and further kidney engraftment into the same pig in vivo. After NMP, organoid-infused porcine kidneys were transplanted into porcine recipients. Transplanted organs and contralateral controls were harvested 24h (n= 3) and 48h (n= 3) after transplantation.* ***b,*** *Arterial flow (mL/min/100g) of organoid-infused kidneys during NMP. The yellow arrow indicates the time of organoid infusion.* ***c,*** *Lactate dehydrogenase (LDH) levels (U/L) during NMP. In b,c, n= 6 organoid-infused kidneys, n= 1 kidney without organoids (Mock).* ***d,e,*** *IVIS fluorescent images of organoid-infused kidneys 24h (NMP #1 + org + 24h Tx) (n= 1) (d) and 48h (NMP*

#4 + org + 48h Tx) (n= 1) (e) after transplantation. Non-perfused contralateral kidneys are also shown (CTL). Whole and coronal organ views are shown. ***f,*** *Quantification of the average radiant efficiency (photons/s/cm^2/sr) from the IVIS images of organoid-infused kidneys 24h and 48h post-transplantation. Data are mean ± s.d. n= 3 kidneys + 24h Tx, n= 1 kidney + 48h Tx.* ***g,*** *Detection of Alu repeat sequences of human DNA by in situ hybridization in formalin fixed paraffin sections of porcine renal cortex from transplanted kidneys 24 h after transplantation (left panels). Scale bars, 2mm, 50µm (magnified views). Immunofluorescence and confocal images in consecutive paraffin sections for the detection of ECAD (grays), LTL (green), KIM-1 (red) and DAPI (blue) are shown (right panels). Scale bars, 2mm (whole slide image), 100µm (magnified views #1 to #3), 25µm (closer magnified views).* ***h,*** *Detection of Alu repeat sequences of human DNA by in situ hybridization in formalin fixed paraffin sections of porcine renal cortex from transplanted kidneys 48 h after transplantation (left panels). Scale bars, 2mm, 50µm (magnified views). Immunofluorescence and confocal images in consecutive paraffin sections for the detection of ECAD (grays), LTL (green), KIM-1 (red) and DAPI (blue) are shown (right panels). Scale bars, 2mm (whole slide image), 100µm (magnified views #1 to #3). In (g,h) dashed orange lines indicate areas containing cells of human origin. Yellow asterisks indicate KIM-1 positive tubuli.* ***i,*** *Quantification of the percentage of Xenolight positive cells by flow cytometry in renal cortical tissue from organoid-infused kidneys 24h and 48h after in vivo transplantation. Data are mean ± s.d. n = 3 biological replicates/group. ns, no statistical significance. Unpaired Student's t test.*

1.3. Panels **a-d** that are shown in ***(Annex) Figure 3*** correspond to the new data included in **<u>Figure 4 of the manuscript</u>:**

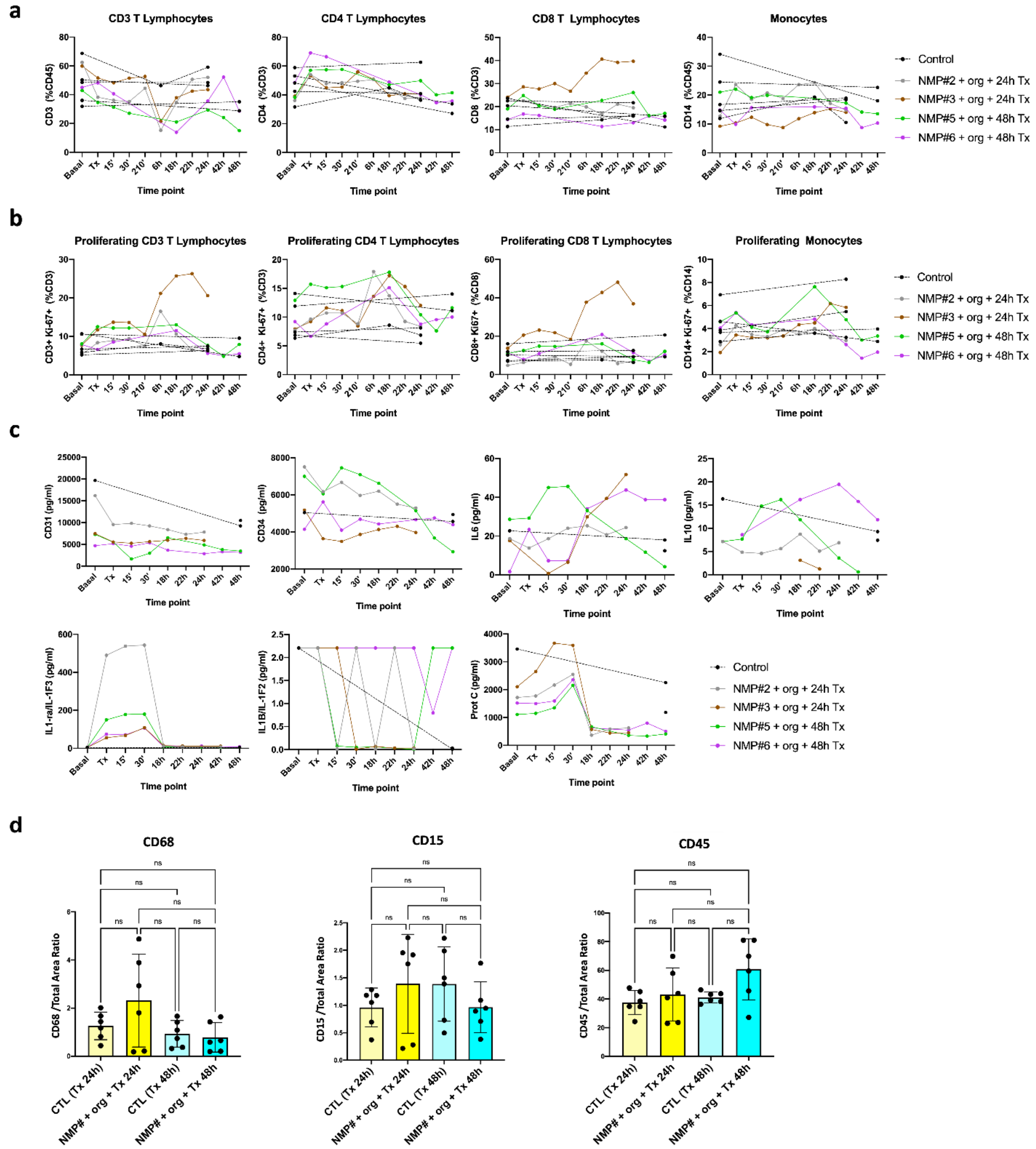

(Annex) Figure 3. ***Analysis of potential systemic and local immune response after in vivo engraftment of porcine kidneys infused with human kidney organoids.*** *Peripheral blood mononuclear cells of pigs at different timepoint after the perfusion and engraftment were analyzed by flow cytometry.* ***a****, Levels of total CD3 lymphocytes, CD4, CD8 or monocytes as percentage of total CD45 cells were analyzed.* ***b****, Proliferating levels of CD3 lymphocytes, CD4, CD8 or monocytes by the expression of Ki-67. Each line represents a single pig.* ***c****, Serum levels of CD31, CD34, IL6, IL10, IL1-ra, IL1B and C reactive protein measured by MILLIPLEX®. Mann-Whitney test was used to compared between the 2 experimental groups at 0, 24 and 48 hours, no statistical differences were found. Paired Wilcoxon matched-pairs signed rank test was used to analyze changes inside the same group at 0, 24 and 48 hours. P-values less than 0.05 were considered statistically significant.* ***d****, Image quantification of CD68, CD15 and CD45 area relative to total tissue section area in renal cortical biopsies retrieved from kidneys 24h and 48h post-engraftment. Data are mean ± s.d. n = 3 biological replicates/group with technical duplicates each. ns, no statistical significance. Kruskal-Wallis (two-sided) followed by Dunn's multiple comparisons test.*

We attach **in this Annex** a link to all the immunohistochemistry images from which the analysis in ***(Annex) Figure 3 panel d*** was done (https://tinyurl.com/29k6d52d). Images can be visualized with the NDP.view2 Image viewing free software (https://www.hamamatsu.com/jp/en/product/life-science-and-medical-systems/digital-slide-scanner/U12388-01.html) or ImageJ2 (version 2.3.0) software (https://imagej.net/software/fiji/).

2. New panels included in the Supplementary figures of the manuscript

2.1. Panel **a** that is shown in ***(Annex) Figure 4*** correspond to the new data included in **Supplementary Fig. 7n of the manuscript:**

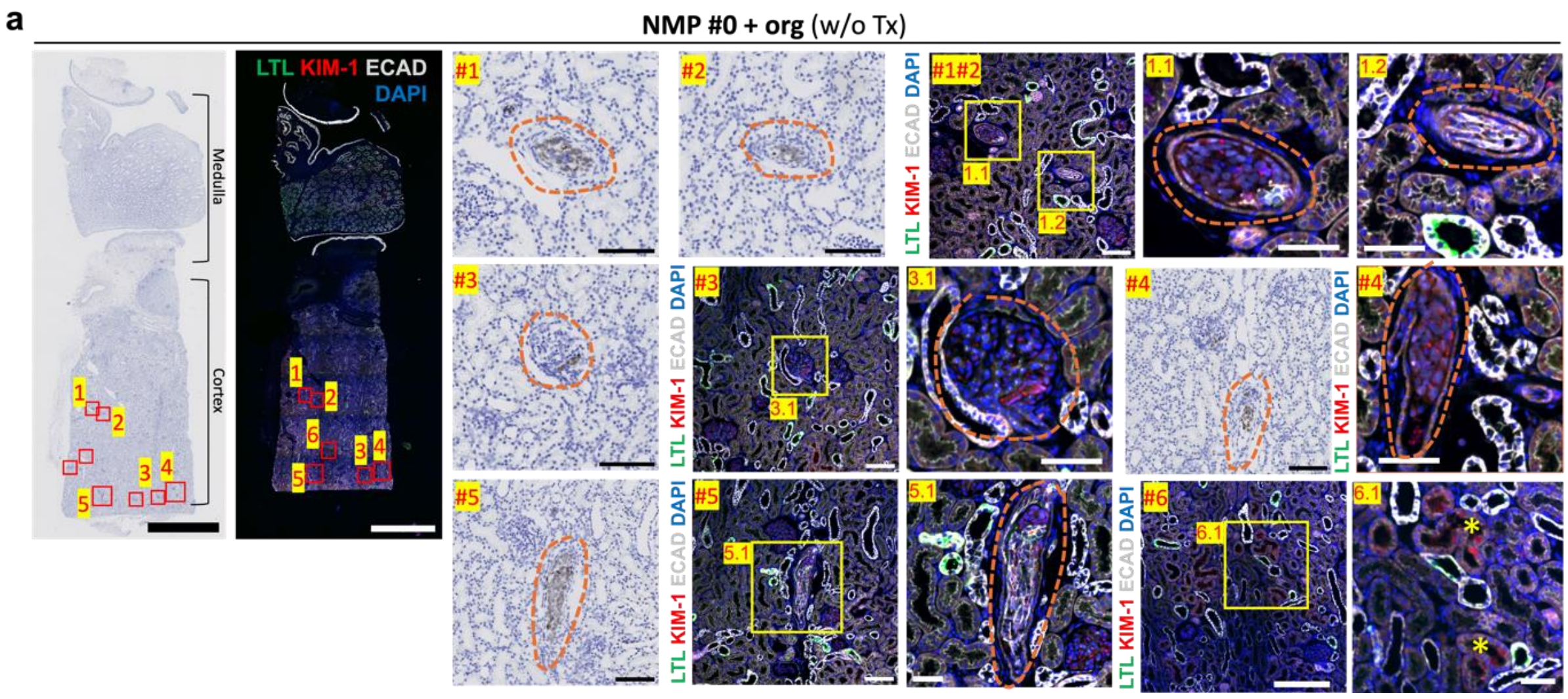

(Annex) Figure 4. ***a***, *Detection of E-Cadherin (ECAD; white), Lotus Tetraglobus Lectin (LTL; green) and KIM-1 (red) in consecutive paraffin sections positive for the detection of Alu repeat sequences of human DNA (red boxes highlighted with dashed numbers). Images correspond to kidneys after infusion of intact kidney organoids during ex vivo NMP. Scale bars, 2 mm, 100 µm (magnified views), 50 µm (closer magnified views). Dashed orange lines indicate areas containing cells of human origin. Yellow asterisks indicate KIM-1 positive tubuli.*

2.2. Panels **g-i** that are shown in ***(Annex) Figure 5*** correspond to the new data included in **Supplementary Fig. 8g-i of the manuscript**:

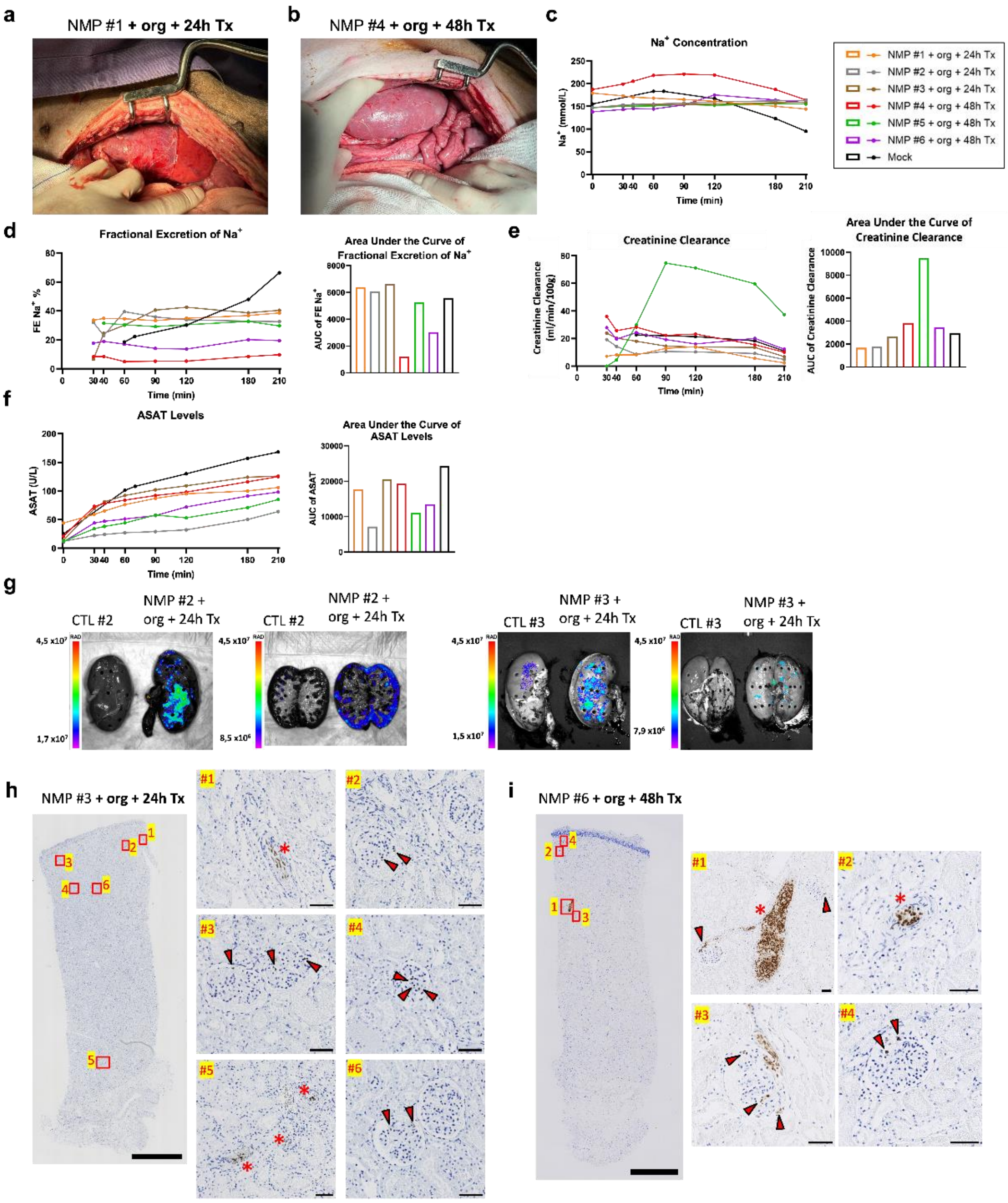

(Annex) Figure 5. ***In vivo engraftment of porcine kidneys treated with organoids. a,*** *Photograph of the engrafted kidney 24h after reimplantation in pig (NMP #2 + org + 24h Tx).* ***b,*** *Photograph of the engrafted kidney 48h after reimplantation in pig (NMP #2 + org + 48h Tx).* ***c,*** *Sodium concentration (mmol/L).* ***d,*** *FE of sodium (FENa+) (%) area-under-the curve (AUC).* ***e,*** *Creatinine clearance (mL/min/100g) and AUC.* ***f,*** *Aspartate aminotransferase (ASAT) levels (U/L) and AUC. In* ***c-f,*** *n= 6 kidneys were infused with organoids (NMP #1, #2, #3 + org + 24h Tx and NMP #4, #5, #6 + org + 48h Tx) and n= 1 kidney was not infused with organoids (Mock) during NMP.* ***g,*** *IVIS fluorescent images of kidneys infused with kidney organoids during ex vivo NMP and transplanted into porcine recipients for 24 h. n= 2 (NMP #2, #3 + org + 24h Tx). Non-perfused contralateral kidneys are also shown (CTL). Whole and coronal organ views are shown.* ***h,i,*** *Detection of Alu repeat sequences of human DNA by in situ hybridization in formalin fixed paraffin sections of porcine renal cortex from engrafted kidneys 24h (h) and 48h (i) post-engraftment. Red arrowheads indicate Alu+ cells in glomerular structures. Red asterisks indicate Alu+ cells in vascular structures. Scale bars, 2mm, 50µm (magnified views).*

2.3. Panels **a-c** that are shown in ***(Annex) Figure 6*** correspond to the new data included in **Supplementary Fig. 9a-c of the manuscript:**

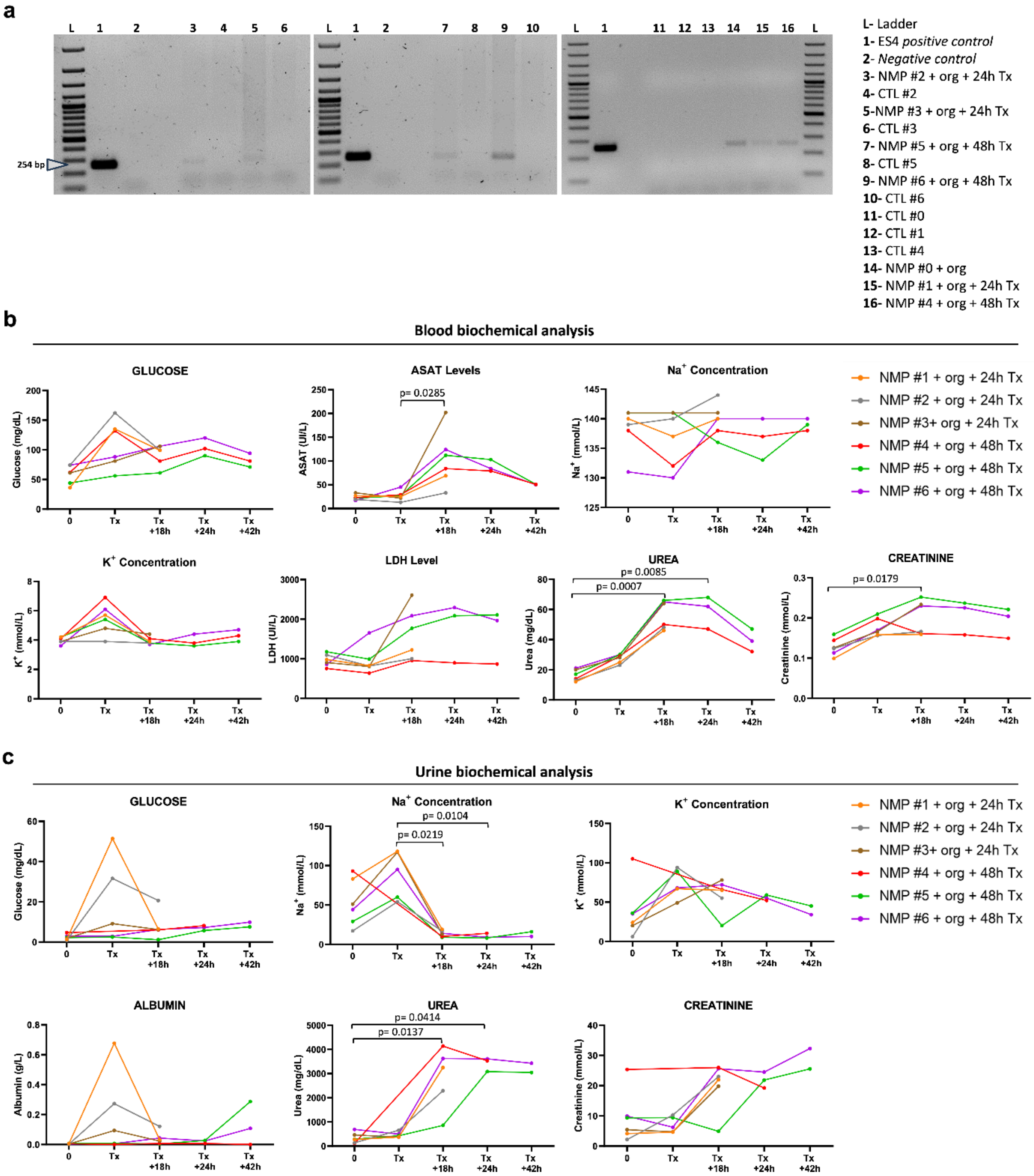

(Annex) Figure 6. ***Human cell detection and biochemical analysis in reimplanted animals. a,*** *Electrophoresed PCR products showing band sized 254bp for the detection of SRY (Y chromosome) in biopsy samples from organoid-infused kidneys before and after in vivo engraftment compared to the correspondent contralateral kidney biopsy samples.* ***b,*** *Blood and* ***c,*** *urine biochemical analysis performed in animals before (t0, basal) and after transplantation (Tx). n= 3 animals reimplanted with organoid-treated kidneys for 24h (NMP #1, #2, #3 + org + 24h Tx) and n= 3 animals reimplanted with organoid-treated kidneys for 48h (NMP #4, #5, #6 + org + 48h Tx). Kruskal-Wallis (two-sided) followed by Dunn's multiple comparisons test. p values for statistically significant comparisons are shown.*

2.4. Panels **a-c** that are shown in ***(Annex) Figure 7*** correspond to the new data included in **Supplementary Fig. 10a-d of the manuscript**:

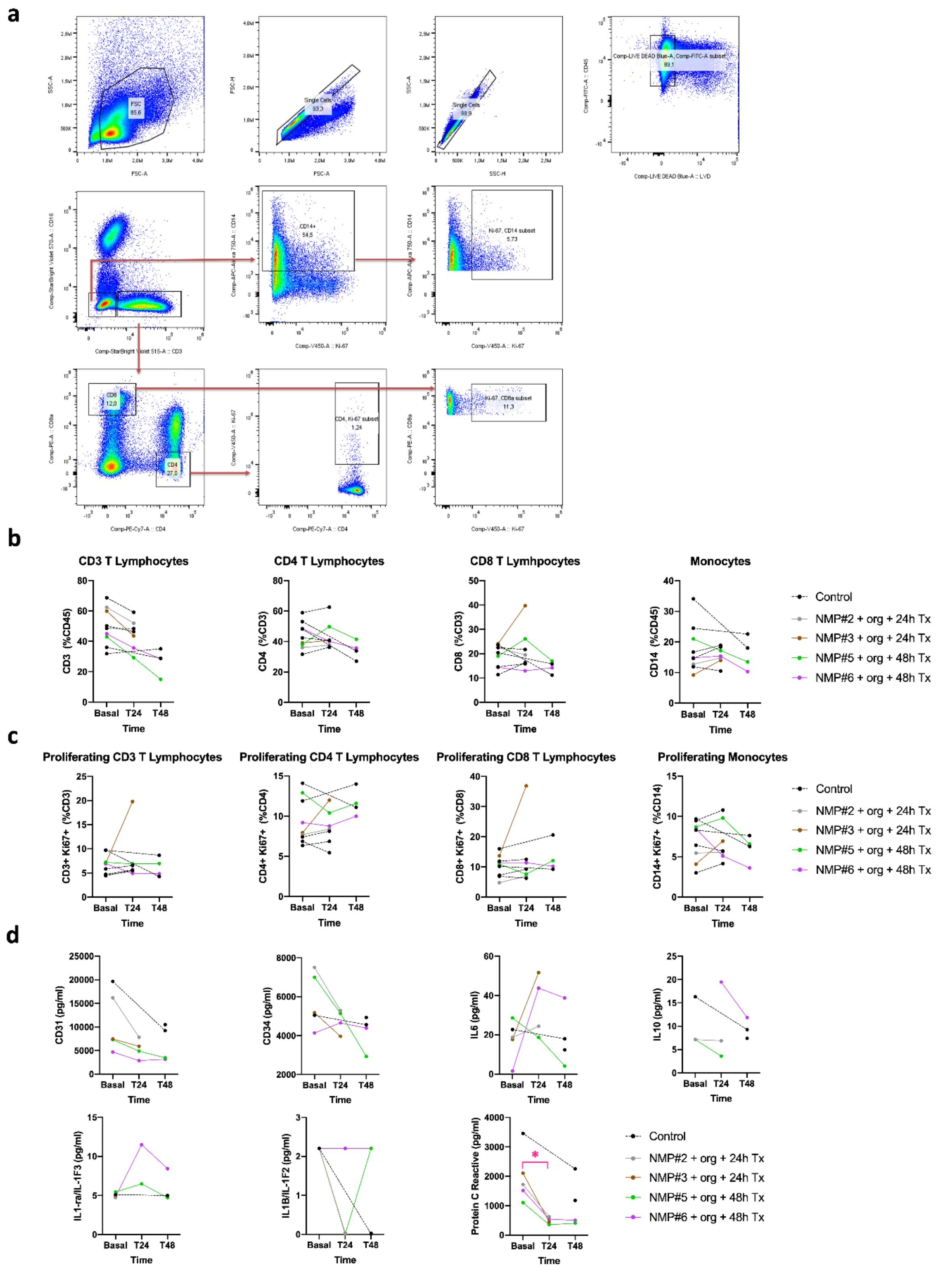

(Annex) Figure 7. ***Systemic inmune response after in vivo engraftment of porcine kidneys infused with human kidney organoids. a****, Flow cytometry gating strategy for analyzing peripheral blood mononuclear cells of pigs at different timepoints after the perfusion and transplantation (Tx) were analyzed by flow cytometry.* ***b****, Levels of total CD3 lymphocytes, CD4, CD8 or monocytes as percentage of total CD45 cells were analyzed at times 0, 24 and 48 hours.* ***c****, Proliferating levels of CD3 lymphocytes, CD4, CD8 or monocytes by the expression of Ki-67 at times 0, 24 and 48 hours. Each line represents a single pig.* ***d****, Serum levels of CD31, CD34, IL6, IL10, IL1-ra, IL1B and C reactive protein measured*

by MILLIPLEX®. Mann-Whitney test was used to compared between the 2 experimental groups at 0, 24 and 48 hours, no statistical differences were found. Paired Wilcoxon matched-pairs signed rank test was used to analyze changes inside the same group at 0, 24 and 48 hours. P-values less than 0.05 were considered statistically significant.

2.4. Data shown in New **Supplementary Table 1 and Supplementary Table 6 in the manuscript.**

3. Summary of all the NMP and in vivo experiments conducted in the manuscript

We have made use of a total of 16 animals for the realization of all the experiments included in the manuscript:
Infusion of disaggregated cells from organoids during NMP (n=2)

Infusion of intact organoids during NMP (n=5)

Infusion of perfusate during NMP (n=2)

Infusion of intact organoids during NMP and auto-TX for 24 h (n=3)

Infusion of intact organoids during NMP and auto-TX for 48 h (n=3)

Infusion of perfusate during NMP and auto-TX with nephrectomy for 24 h (n=1)

In ***(Annex) Table 1*** we summarize the experiments included in the manuscript. We provide information about the realization of the experiments in this revision process as well as the associated Figures within the manuscript showing the related data.

(Annex) Table 1. *List of experiments conducted within the lifetime of the project. CTL: control; N/A: not applicable.*

ANIMAL NAME	INTERNAL SAMPLE NAME	EXPERIMENTAL CONDITION	SAMPLE CODING (before Tx)	SAMPLE CODING (after Tx)	INCLUDED IN THE FIRST SUBMISSION	INCLUDED IN THE SECOND and THIRD SUBMISSION	FIGURES CONTATINING THIS EXPERIMENT
ANIMAL A	E250	Perfused with single cells	Disaggregated 1	N/A	YES	YES	**Suppl. Fig. 6**
ANIMAL B	E256	Perfused with single cells	Disaggregated 2	N/A	YES	YES	**Suppl. Fig. 6**
ANIMAL C	E251	PERFUSED with intact organoids low dose	Low dose	N/A	YES	YES	**Suppl. Fig.5**
ANIMAL D	E257	PERFUSED with intact organoids low dose	Low dose	N/A	YES	YES	**Supp. Fig. 5**
ANIMAL E	E999	PERFUSED with intact organoids low dose	Low dose	N/A	YES	YES	**Suppl. Fig. 5**

ANIMAL F	E998	PERFUSED with intact organoids high dose	High dose	N/A	YES	YES	**Suppl. Fig. 5**
ANIMAL G	E996	Perfused with Perfusate alone	Mock	N/A	YES	YES	**Suppl. Fig. 5**
ANIMAL H	E997	Perfused with Perfusate alone	Mock	N/A	YES	YES	**Suppl. Fig. 5**
ANIMAL 1	Kidney 1	**PERFUSED without organoids and TX with Nephrectomy**	MOCK +24H Tx	MOCK +24H Tx	NO	YES	**Fig.3, Suppl. Fig 8**
ANIMAL 1	Kidney 2	**CTRL of Kidney 1**		MOCK Ctl 4H	NO	YES	**Supp.Figs. 8, 9**
ANIMAL 2	Kidney 3	**PERFUSED with intact organoids and Tx 48 H**	NMP#4+org	NMP#4+org+48h Tx	NO	YES	**Fig. 3, 4 Suppl. Figs. 8, 9**
ANIMAL 2	Kidney 4	**CTRL of Kidney 3**	N/A	CTL#4	NO	YES	**Figs.3,4 Suppl.Figs.8, 9**
ANIMAL 3	Kidney 5	**PERFUSED with intact organoids and Tx 24 H**	NMP#1+org	NMP#1+org+24h Tx	NO	YES	**Figs. 3,4 Suppl. Figs. 8, 9**
ANIMAL 3	Kidney 6	**CTRL of Kidney 3**	N/A	CTL#1	NO	YES	**Figs. 3,4 Suppl. Fig. 9**
ANIMAL 5	Kidney 9	**PERFUSED with intact organoids**	NMP#0+org	N/A	NO	YES	**Suppl. Figs. 7, 9**
ANIMAL 5	Kidney 10	**CTRL of Kidney 9**	CTL#0	N/A	NO	YES	**Suppl. Figs. 7, 9**
**ANIMAL 6**	**Kidney 11**	**PERFUSED with intact organoids and Tx 48 H**	**NMP#5+org**	**NMP#5+org+48h Tx**	NO	YES	**Figs. 3, 4 Suppl. Figs. 8,9,10**
**ANIMAL 6**	**Kidney 12**	**CTRL of Kidney 11**	**N/A**	**CTL#5**	NO	YES	**Fig. 4, Suppl. Fig. 9**
**ANIMAL 7**	**Kidney 13**	**PERFUSED with intact organoids and Tx 48 H**	**NMP#6+org**	**NMP#6+org+48h Tx**	NO	YES	**Figs. 3, 4 Suppl. Figs. 8, 9, 10**
**ANIMAL 7**	**Kidney 14**	**CTRL of Kidney 13**	**N/A**	**CTL#6**	NO	YES	**Fig. 4 Suppl. Fig. 9**
**ANIMAL 8**	**Kidney 15**	**PERFUSED with intact organoids and Tx 24 H**	**NMP#2+org**	**NMP#2+org+24h Tx**	NO	YES	**Fig. 4 Suppl. Figs. 8, 9, 10**
**ANIMAL 8**	**Kidney 16**	**CTRL of Kidney 15**	**N/A**	**CTL#2**	NO	YES	**Fig. 4 Suppl. Figs. 8,9**
**ANIMAL 9**	**Kidney 17**	**PERFUSED with intact organoids and Tx 24 H**	**NMP#3+org**	**NMP#3+org+24h Tx**	NO	YES	**Figs. 3, 4 Suppl. Figs. 8, 9, 10**

ANIMAL 9	Kidney 18	CTRL of Kidney 17	N/A	CTL#3	NO	YES	**Suppl. Figs. 8,9**

4. Qualitative assessment of potential tissue damage in biopsies from NMP and in vivo experiments by hematoxylin-eosin staining and pathological evaluation

To date, there is no consensus on rather basic aspects of machine perfusion for kidneys such as perfusate composition or pressure settings. Similarly, recent studies have evaluated the amount of endothelial injury during NMP in porcine kidneys (PMID: 37456589) and whether different pressures and hematocrits affect this injury (PMID: 33979315; PMID: 24811916; PMID: 16673356). Interestingly, previous studies have compared structural cell injury in isolated porcine kidneys during NMP for seven hours with autologous, allogeneic porcine, or human red blood cells (RBC) observing glomerular and tubular damage in all experimental groups with signs of pathological hyperfiltration and microvascular injury in the human RBC group (PMID: 32155167). Here we have performed a qualitative evaluation of damage in our experimental set up through hematoxylin eosin staining and pathology assessment (shown ***in Annex-Table 2***). The biopsies were examined, and a damage scoring system was developed, based on previously reported scoring systems (PMID: 30951018) and validated by an experienced renal pathologist (University of Leuven; participating as co-authors in the revised manuscript). The sections were scored on a scale of 0–3 for the severity of acute tubular injury (ATI), tubular necrosis, tubulitis, interstitial inflammation, Glomerulitis (g), Peritubular capillaritis (ptc), Endarteritis (v), interstitial fibrosis and tubular atrophy (IFTA).

Specimen	ATI	Severity of ATI	Tubular necrosis	Tubulitis	Interstitial inflammation	Type of inflammatory cells	Glomerulitis	Peritubular capillaritis	Endarteritis	IFTA
MOCK CTL 4h U_5_HE	1	1	0	0	0	mononuclear cell infiltrates	0	0	0	5%
CTL#0 U 20 - 2023-12-04	1	1	0	0	0	mononuclear cell infiltrates	0	0	0	0
CTL#0 U_5_HE - 2024-05-04	1	1	0	0	0	nil	0	0	0	0
CTL#1 U 20 - 2023-12-04	1	1	0	0	0	nil	0	0	0	0
CTL#1 U_5_HE - 2024-05-04	1	1	0	0	1	mononuclear cell infiltrates	0	0	0	0
CTL#2 C(1)_10_HE - 2024-06-20	1	2	0	0	0	nil	0	0	0	0
CTL#3 C(1)_10_HE - 2024-06-20	1	1	0	0	0	nil	0	0	0	0
CTL#4 U 20 - 2023-12-04	1	2	0	0	0	nil	0	0	0	0
CTL#4 U_5_HE - 2024-05-04	1	1	0	0	0	nil	0	0	0	0
CTL#5 C (1)_HE - 2024-05-17	1	1	0	0	0	nil	0	0	0	0
CTL#5 C (2)_HE - 2024-05-17	1	1	0	0	0	mononuclear cell infiltrates	0	0	0	0
CTL#5 L (1)_HE - 2024-05-17	1	1	0	0	0	nil	0	0	0	0
CTL#5 L (2)_HE - 2024-05-17	1	1	0	0	0	nil	0	0	0	0
CTL#5 U (1)_HE - 2024-05-17	1	1	0	0	0	nil	0	0	0	0
CTL#5 U (2)_HE - 2024-05-17	1	1	0	0	0	mononuclear cell infiltrates	0	0	0	0
CTL#6 C (1)_HE - 2024-05-17	1	1	0	0	0	nil	0	0	0	0
CTL#6 C (2)_HE - 2024-05-17	1	1	0	0	0	nil	0	0	0	0
CTL#6 L (1)_HE - 2024-05-17	1	1	0	0	0	nil	0	0	0	0
CTL#6 L (2)_HE - 2024-05-17	1	1	0	0	0	nil	0	0	0	0
CTL#6 U (1)_HE - 2024-05-17	1	1	0	0	0	nil	0	0	0	0
CTL#6 U (2)_HE - 2024-05-17	1	1	0	0	0	nil	0	0	0	0
MOCK +24 H tx U_5_HE	1	3	0	0	0	mononuclear cell infiltrates	0	0	0	0
NMP#0+org C 20 - 2023-12-04	1	1	0	0	0	nil	0	0	0	0
NMP#0+org L 5 - 2023-12-04	1	2	0	0	1	mononuclear cell infiltrates	0	0	0	0
NMP#0+org L 20 - 2023-12-04	1	2	0	0	0	mononuclear cell infiltrates	0	0	0	0
NMP#0+org U 20 - 2023-12-04	1	1	0	0	0	nil	0	0	?1	0
NMP#0+org U_5_HE - 2024-05-04	1	1	0	0	0	nil	0	0	0	0
NMP#1+org+24h Tx C 5 - 2023-12-04	1	2	1	0	1	mononuclear cell infiltrates	0	0	0	0
NMP#1+org+24h Tx C 20 -2023-12-04	1	2	1	0	1	mononuclear cell infiltrates	0	0	0	0
NMP#1+org+24h Tx L 5 - 2023-12-04	1	3	1	0	1	mixed - L, P, N	0	0	0	0
NMP#1+org+24h Tx L 20 - 2023-12-04	1	3	1	0	1	mixed - L, P, N	0	0	0	0
NMP#1+org+24h Tx U 5 - 2023-12-05	1	2	1	0	1	mixed - L, P, N	0	0	0	0
NMP#1+org+24h TxU_5_HE - 2024-05-04	1	2	1	0	1	mixed - L, P, N	0	0	0	0
NMP#2+org+24h Tx C (2)_10_HE - 2024-06-20	1	2	0	0	0	nil	0	0	0	0
NMP#2+org+24h Tx C (1)_10_HE - 2024-06-20	1	2	0	0	0	nil	0	0	0	0
NMP#3+org+24h TxC(1)_10_HE - 2024-06-20	1	3	1	0	0	nil	0	0	0	0
NMP#3+org+24h Tx C(2)_10_HE - 2024-06-20	1	3	1	0	0	nil	0	0	0	0
NMP#4+org+48h Tx C 5 - 2023-12-05	0	0	0	0	0	nil	0	0	0	0
NMP#4+org+48h Tx L 5 - 2023-12-05	1	1	1	0	0	mononuclear cell infiltrates	0	0	0	0
NMP#4+org+48h Tx L 20 - 2023-12-05	1	1	1	0	0	mononuclear cell infiltrates	0	0	0	0
NMP#4+org+48h Tx U 5 - 2023-12-05	1	1	1	0	0	mononuclear cell infiltrates	0	0	0	0
NMP#4+org+48h Tx U 20 - 2023-12-05	1	1	1	0	0	mononuclear cell infiltrates	0	0	0	0
NMP#4+org+48h Tx U_5_HE - 2024-05-04	1	1	1	0	0	mononuclear cell infiltrates	0	0	0	0
NMP#5+org+48h Tx C (1)_HE - 20-05-17	1	3	1	0	2	Mixed - L, P, E	0	0	0	0
NMP#5+org+48h Tx C (2)_HE - 20-05-17	1	3	1	0	1	mononuclear cell infiltrates	0	0	0	0
NMP#5+org+48h Tx L (1)_HE - 2024-05-17	1	3	1	0	1	mononuclear cell infiltrates	0	0	0	0
NMP#5+org+48h Tx L (2)_HE - 2024-05-17	1	3	1	0	1	mononuclear cell infiltrates	0	0	0	0
NMP#5+org+48h Tx U (1)_HE - 2024-05-17	1	3	1	0	1	mononuclear cell infiltrates	0	0	0	0
NMP#5+org+48h Tx U (2)_HE - 2024-05-17	1	3	1	0	1	mononuclear cell infiltrates	0	0	0	0
NMP#6+org+48h Tx C (1)_HE - 2024-05-17	1	3	1	0	0	mononuclear cell infiltrates	0	0	0	0
NMP#6+org+48h Tx C (2)_HE - 2024-05-17	1	3	1	0	0	mononuclear cell infiltrates	0	0	0	0
NMP#6+org+48h Tx L (1)_HE - 2024-05-17	1	3	1	0	0	mononuclear cell infiltrates	0	0	0	0
NMP#6+org+48h TxL (2)_HE - 2024-05-17	1	3	1	0	0	mononuclear cell infiltrates	0	0	0	0
NMP#6+org+48h Tx U (1)_HE - 2024-05-17	1	3	1	0	0	mononuclear cell infiltrates	0	0	0	0
NMP#6+org+48h Tx U (2)_HE - 2024-05-17	1	3	1	0	0	mononuclear cell infiltrates	0	0	0	0

(Annex) Table 2*. Qualitative evaluation of damage in all the samples of the study through hematoxylin eosin (H&E) staining and pathology assessment. Formalin fixed paraffin embedded biopsies of the upper (U), lateral (L) and central (C) renal cortex were stained with HE to assess changes in tissue morphology. The sections were scored on a scale of 0–. ATI: acute tubular injury; IFTA: interstitial fibrosis and tubular atrophy (IFTA). ATI severity was also scored on a scale of 0-3. One specimen undergoing NMP in the absence of organoids and further autotransplantation with nephrectomy during 24 h (MOCK+24 h Tx) was included in the study. CTL refers to control lateral specimens. NMP#0+org refers to one specimen undergoing NMP for 3 hours after organoid infusion. NMP#1-3+org+24h Tx refers to specimens (#1, #2 and #3) undergoing NMP for 3 hours after organoid infusion and further autotransplantation for 24h. NMP#4-6+org+24h Tx refers to specimens (#4, #5 and #6) undergoing NMP for 3 hours after organoid infusion and further autotransplantation for 48h.*

Our results show that all the analyzed samples, including control lateral kidneys, exhibit ATI. No signs of tubular necrosis, tubulitis, interstitial inflammation, Glomerulitis (g), Peritubular capillaritis (ptc), or endarteritis were found. We would like to highlight that the level of severity of ATI ranges from 1 to 3 in all the experimental conditions. Importantly, we encountered that NMP+org conditions and NMP+org+Tx conditions led to a low (score 1) to severe (score 3) extent of ATI whereas a kidney specimen undergoing NMP in the absence of organoids and Tx with nephrectomy for 24h led to a severity of ATI with score 3. This observation suggests that NMP alone, in the absence of organoids, together with the surgery procedure, already induces a certain degree of acute damage in the tubuli and that the level of damage is comparable to that found in NMP+org+Tx conditions.

Here we attach a link to all the images from which this analysis was done (https://tinyurl.com/yc6pzd2m). Images can be visualized with the NDP.view2 Image viewing free software (https://www.hamamatsu.com/jp/en/product/life-science-and-medical-systems/digital-slide-scanner/U12388-01.html) or ImageJ2 (version 2.3.0) software (https://imagej.net/software/fiji/). Similarly, a link to ***Table 2*** presented can be also found here https://tinyurl.com/yc6pzd2m. In case the editorial board and experts consider that this information is of interest we could include these results in the manuscript.

Point-by-point response to the Reviewers' Comments:

Reviewer #1 (R#1)

R#1: *Thank again for the meaningful edits to the manuscript. The authors clearly put a lot of efforts into further improving the study.*

Authors (A): We thank the reviewer for the very positive evaluation of our work and his/her insightful comments during the revision process, which clearly allowed us to improve our first submission resulting in the incorporation of new data and supportive information that now are incorporated as main figures (**Fig. 3** and **Fig. 4** of the manuscript) and supplementary figures (**Supplementary Fig. 7 to Supplementary Fig. 10** of the manuscript) in the final version of our work.

Some minor remarks:

1) In the revised version of the abstract, the authors stat that the organoid transfer into kidneys is done "with the aim to improve kidney function during organ failure". This is a legit aim, but none of the data supports this claim. I would suggest to remove this part of the sentence and any such speculations throughout the manuscript.

A: We appreciate the reviewer's comment and agree to remove the phrase 'with the aim of improving kidney function during organ failure' from the abstract and throughout the manuscript where it appears, as well as similar speculative statements.

2) In the chapter demonstrating organoid engraftment, the authors mention that they are using slaughterhouse pigs for these experiments. In the next chapter, they describe that they are reimplanting the kidneys into the donor animals. I assume this is a different model. Please clarify.

A: We thank the reviewer for his/her comment. We have now further described in the main text the use of viable porcine kidneys from slaughterhouse pigs to assess kidney organoid and kidney organoid-derived cell engraftment during ex vivo normothermic machine perfusion (results shown in **Supplementary Fig. 5 and Supplementary Fig. 6 in the manuscript**), as well as the use of laboratory pigs (Hybrid Landrace/Large White crossbreed) as porcine model for in vivo transplantation experiments (results shown in **Fig. 3, Fig.4, and Supplementary Fig. 7 to Supplementary Fig. 10 in the manuscript**).
This is also described in the Methods section ('Study design' subsection).

3) Terms such as "in vivo engrafted kidneys" is confusing. Maybe use retransplanted or transplanted instead.

A: We thank the reviewer for his/her suggestion. To avoid confusion, we have now changed the term 'in vivo engrafted kidneys' to 'transplanted kidneys' in the manuscript.

4) The authors state that "These results (of the transplant experiments) suggest that graft viability was mostly preserved during the study period evaluated here, overall showing the feasibility and safety of our approach." I agree with safety and feasibility with some reservation. SFig. 9. Fig. 9 does not show a control, so strong conclusions are difficult. Also, the second, native kidney seems to be in place, but urea, crea and tissue injury markers increase during the early phase.

What is shown is that despite of the interventions, the kidneys look macroscopically viable. However, a histological readout is missing. ***I would suggest to add a histological readout showing and grading the degree of damage to support the conclusion.***

A: We greatly appreciate the reviewer's insightful comment.

In the previous rebuttal letter, we presented histological data to qualitatively evaluate potential damage in our experimental set up through hematoxylin and eosin (HE) staining and pathology assessment. This was summarized in a Table (**Table 1 in this rebuttal letter**) as well as all the HE images used for this analysis were included in a link (https://tinyurl.com/yc6pzd2m).

We understand that the way of sharing this data could be confusing and we apologize for this. Now, considering the expert comment, we have prepared two additional figures showing representative HE images of two different areas of all kidney biopsy sections that were evaluated (**Figure 1** and **Figure 2 in this rebuttal letter**), together with the table summarizing the pathology assessment (**Table 1 in this rebuttal letter**). Our results show that all the analyzed samples, including contralateral kidneys, exhibit some degree of ATI (**Figure 1** and **Figure 2,** and **Table 1 in this rebuttal letter**). In contrast, no signs of tubular necrosis, tubulitis, glomerulitis (g), peritubular capillaritis (ptc), or endarteritis were observed. The severity of ATI ranges from 1 to 3 in all the experimental conditions. This might suggest that NMP, together with the surgical procedure, can induce a certain degree of acute damage in the tubules, as previously reported (PMID: **32155167**). It is important to note that these results align with our previous observations regarding the presence of few tubular structures expressing the marker of tubular injury, the kidney injury molecule-1 (KIM-1), in kidneys after NMP (**Supplementary Fig. 7 in the manuscript**) and *in vivo* transplantation (**Fig. 3 in the manuscript**).

Since this pathology analysis is primarily qualitative, we have not included it in the manuscript. However, we would be delighted to incorporate this data in the manuscript in case the expert considers it appropriate.

Future studies increasing the sample size as well as including spatial transcriptomics analysis, among others, could provide more insights into this important question, enhancing our understanding on the extent and impact of acute damage during NMP and over longer post-transplantation periods.

On the other hand, we have now replaced the term 'safety' to 'viability' in the current version of the manuscript to avoid potential confusion. We would like to thank again the expert for all his/her valuable inputs that have been essential to improve our manuscript.

Specimen	ATI	Severity of ATI	Tubular necrosis	Tubulitis	Interstitial inflammation	Type of inflammatory cells	Glomerulitis	Peritubular capillaritis	Endarteritis	IFTA
MOCK CTL 4h U_5_HE	1	1	0	0	0	mononuclear cell infiltrates	0	0	0	5%
CTL#0 U 20 - 2023-12-04	1	1	0	0	0	mononuclear cell infiltrates	0	0	0	0
CTL#0 U_5_HE - 2024-05-04	1	1	0	0	0	nil	0	0	0	0
CTL#1 U 20 - 2023-12-04	1	1	0	0	0	nil	0	0	0	0
CTL#1 U_5_HE - 2024-05-04	1	1	0	0	1	mononuclear cell infiltrates	0	0	0	0
CTL#2 C(1)_10_HE - 2024-06-20	1	2	0	0	0	nil	0	0	0	0
CTL#3 C(1)_10_HE - 2024-06-20	1	1	0	0	0	nil	0	0	0	0
CTL#4 U 20 - 2023-12-04	1	2	0	0	0	nil	0	0	0	0
CTL#4 U_5_HE - 2024-05-04	1	1	0	0	0	nil	0	0	0	0
CTL#5 C (1)_HE - 2024-05-17	1	1	0	0	0	nil	0	0	0	0
CTL#5 C (2)_HE - 2024-05-17	1	1	0	0	0	mononuclear cell infiltrates	0	0	0	0
CTL#5 L (1)_HE - 2024-05-17	1	1	0	0	0	nil	0	0	0	0
CTL#5 L (2)_HE - 2024-05-17	1	1	0	0	0	nil	0	0	0	0
CTL#5 U (1)_HE - 2024-05-17	1	1	0	0	0	nil	0	0	0	0
CTL#5 U (2)_HE - 2024-05-17	1	1	0	0	0	mononuclear cell infiltrates	0	0	0	0
CTL#6 C (1)_HE - 2024-05-17	1	1	0	0	0	nil	0	0	0	0
CTL#6 C (2)_HE - 2024-05-17	1	1	0	0	0	nil	0	0	0	0
CTL#6 L (1)_HE - 2024-05-17	1	1	0	0	0	nil	0	0	0	0
CTL#6 L (2)_HE - 2024-05-17	1	1	0	0	0	nil	0	0	0	0
CTL#6 U (1)_HE - 2024-05-17	1	1	0	0	0	nil	0	0	0	0
CTL#6 U (2)_HE - 2024-05-17	1	1	0	0	0	nil	0	0	0	0
**MOCK +24 H tx U_5_HE**	1	3	0	0	0	mononuclear cell infiltrates	0	0	0	0
**NMP#0+org C 20 - 2023-12-04**	1	1	0	0	0	nil	0	0	0	0
**NMP#0+org L 5 - 2023-12-04**	1	2	0	0	1	mononuclear cell infiltrates	0	0	0	0
**NMP#0+org L 20 - 2023-12-04**	1	2	0	0	0	mononuclear cell infiltrates	0	0	0	0
**NMP#0+org U 20 - 2023-12-04**	1	1	0	0	0	nil	0	0	?1	0
**NMP#0+org U_5_HE - 2024-05-04**	1	1	0	0	0	nil	0	0	0	0
**NMP#1+org+24h Tx C 5 - 2023-12-04**	1	2	1	0	1	mononuclear cell infiltrates	0	0	0	0
**NMP#1+org+24h Tx C 20 -2023-12-04**	1	2	1	0	1	mononuclear cell infiltrates	0	0	0	0
**NMP#1+org+24h Tx L 5 - 2023-12-04**	1	3	1	0	1	mixed - L, P, N	0	0	0	0
**NMP#1+org+24h Tx L 20 - 2023-12-04**	1	3	1	0	1	mixed - L, P, N	0	0	0	0
**NMP#1+org+24h Tx U 5 - 2023-12-05**	1	2	1	0	1	mixed - L, P, N	0	0	0	0
**NMP#1+org+24h TxU_5_HE - 2024-05-04**	1	2	1	0	1	mixed - L, P, N	0	0	0	0
**NMP#2+org+24h Tx C (2)_10_HE - 2024-06-20**	1	2	0	0	0	nil	0	0	0	0
**NMP#2+org+24h Tx C (1)_10_HE - 2024-06-20**	1	2	0	0	0	nil	0	0	0	0
**NMP#3+org+24h TxC(1)_10_HE - 2024-06-20**	1	3	1	0	0	nil	0	0	0	0
**NMP#3+org+24h Tx C(2)_10_HE - 2024-06-20**	1	3	1	0	0	nil	0	0	0	0
**NMP#4+org+48h Tx C 5 - 2023-12-05**	0	0	0	0	0	nil	0	0	0	0
**NMP#4+org+48h Tx L 5 - 2023-12-05**	1	1	1	0	0	mononuclear cell infiltrates	0	0	0	0
**NMP#4+org+48h Tx L 20 - 2023-12-05**	1	1	1	0	0	mononuclear cell infiltrates	0	0	0	0
**NMP#4+org+48h Tx U 5 - 2023-12-05**	1	1	1	0	0	mononuclear cell infiltrates	0	0	0	0
**NMP#4+org+48h Tx U 20 - 2023-12-05**	1	1	1	0	0	mononuclear cell infiltrates	0	0	0	0
**NMP#4+org+48h Tx U_5_HE - 2024-05-04**	1	1	1	0	0	mononuclear cell infiltrates	0	0	0	0
**NMP#5+org+48h Tx C (1)_HE - 20-05-17**	1	3	1	0	2	Mixed - L, P, E	0	0	0	0
**NMP#5+org+48h Tx C (2)_HE - 20-05-17**	1	3	1	0	1	mononuclear cell infiltrates	0	0	0	0
**NMP#5+org+48h Tx L (1)_HE - 2024-05-17**	1	3	1	0	1	mononuclear cell infiltrates	0	0	0	0
**NMP#5+org+48h Tx L (2)_HE - 2024-05-17**	1	3	1	0	1	mononuclear cell infiltrates	0	0	0	0
**NMP#5+org+48h Tx U (1)_HE - 2024-05-17**	1	3	1	0	1	mononuclear cell infiltrates	0	0	0	0
**NMP#5+org+48h Tx U (2)_HE - 2024-05-17**	1	3	1	0	1	mononuclear cell infiltrates	0	0	0	0
**NMP#6+org+48h Tx C (1)_HE - 2024-05-17**	1	3	1	0	0	mononuclear cell infiltrates	0	0	0	0
**NMP#6+org+48h Tx C (2)_HE - 2024-05-17**	1	3	1	0	0	mononuclear cell infiltrates	0	0	0	0
**NMP#6+org+48h Tx L (1)_HE - 2024-05-17**	1	3	1	0	0	mononuclear cell infiltrates	0	0	0	0
**NMP#6+org+48h TxL (2)_HE - 2024-05-17**	1	3	1	0	0	mononuclear cell infiltrates	0	0	0	0
**NMP#6+org+48h Tx U (1)_HE - 2024-05-17**	1	3	1	0	0	mononuclear cell infiltrates	0	0	0	0
**NMP#6+org+48h Tx U (2)_HE - 2024-05-17**	1	3	1	0	0	mononuclear cell infiltrates	0	0	0	0

Table 1. *Qualitative evaluation of damage in all the samples of the study through hematoxylin eosin (H&E) staining and pathology assessment. Formalin fixed paraffin embedded biopsies of the upper (U), lateral (L) and central (C) renal cortex were stained with HE to assess changes in tissue morphology. The sections were scored on a scale of 0–. ATI: acute tubular injury; IFTA: interstitial fibrosis and tubular atrophy. ATI severity was also scored on a scale of 0-3. One specimen undergoing NMP in the absence of organoids and further autotransplantation with nephrectomy during 24 h (MOCK+24 h Tx) was included in the study. CTL refers to control lateral specimens. NMP#0+org refers to one specimen undergoing NMP for 3 hours after organoid infusion. NMP#1-3+org+24h Tx refers to specimens (#1, #2 and #3) undergoing NMP for 3 hours after organoid infusion and further autotransplantation for 24h. NMP#4-6+org+24h Tx refers to specimens (#4, #5 and #6) undergoing NMP for 3 hours after organoid infusion and further autotransplantation for 48h.*

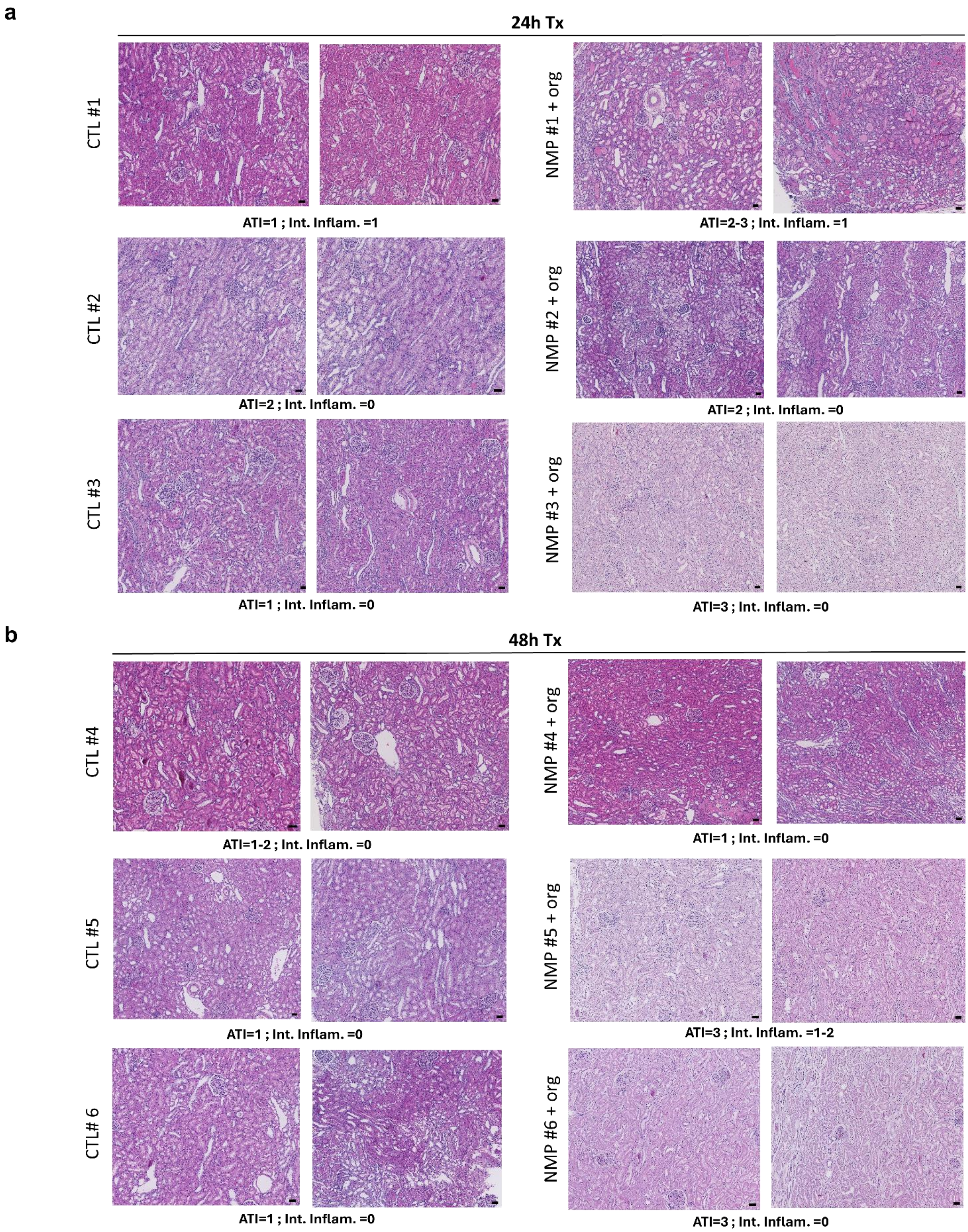

Figure 1. ***Histological analysis of kidneys after in vivo transplantation. a.*** *Representative hematoxylin and eosin images of stained paraffin sections from* ***a.*** *kidneys infused with kidney organoids during ex vivo NMP following reimplantation in pigs for 24h. n= 3 (NMP #1, #2, #3 + org + 24h Tx).* ***b.*** *kidneys infused with kidney organoids during ex vivo NMP following reimplantation in pigs for 48h. n= 3 (NMP #4, #5, #6 + org + 48h Tx). Correspondent non-perfused contralateral kidneys are also shown (CTL). Severity of acute tubular*

injury (ATI) is scored on a scale of 0-3. Interstitial inflammation (Int. Inflam.) is indicated with 1 (presence) or 0 (absence). Scale bars, 50µm.

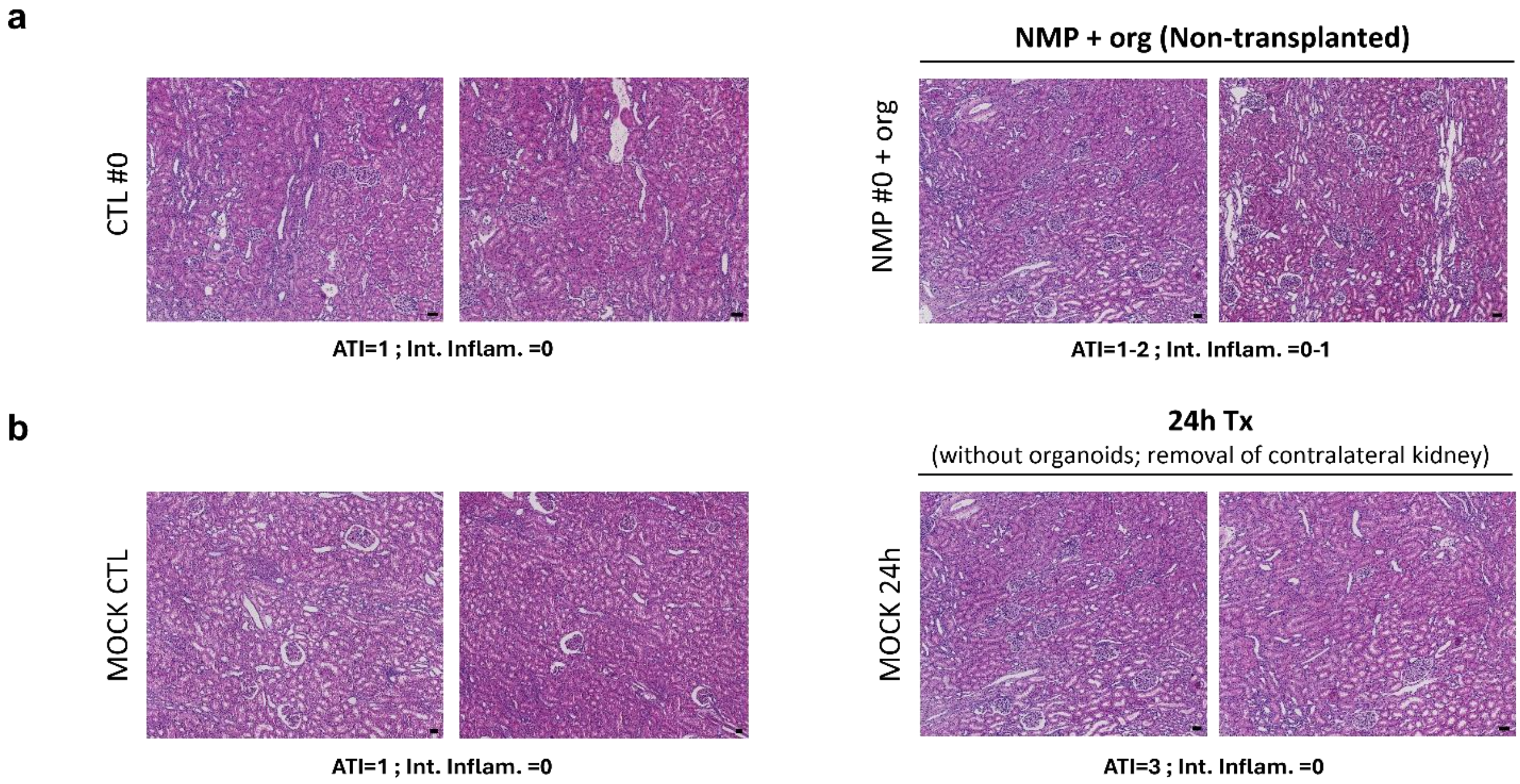

Figure 2. ***Histological evaluation of kidneys a.*** *after infusion of organoids during ex vivo NMP. n= 1 (NMP #0).* ***b.*** *without infusion of organoids during NMP and after in vivo transplantation for 24h with nephrectomy of the contralateral kidney. n= 1 (MOCK 24h). Correspondent non-perfused contralateral kidneys are also shown (CTL). Severity of acute tubular injury (ATI) is scored on a scale of 0-3. Interstitial inflammation (Int. Inflam.) is indicated with 1 (presence) or 0 (absence). Scale bars, 50µm.*

5) Rebuttal letter Fig 1a displays the results of 1 kidney transplanted without organoids. In this procedure, the contralateral kidney was removed. In the intervention group it was

A: We thank the reviewer for their comment. We would also like to apologize beforehand as the question appears incomplete in the email that we got from the Editorial.
In the previous rebuttal letter, we presented blood urea and creatinine levels comparing organoid-treated kidneys (n=6) with mock-treated kidney [without organoids; with contralateral nephrectomy, (n=1)] during the first 18 hours of postoperative follow-up (now ***Figure 3*** **in this rebuttal letter**). In this regard, we agree that a more appropriate comparison would involve using the same surgical procedure in all experimental groups. At the same time, this first study also highlights that early changes in blood and urea parameters may be influenced by various factors related to the surgical procedure (such as ischemia-reperfusion injury, surgical injury, and hemodynamic effects) as well as the physiological response of the organism during the initial hours of postoperative recovery, as previously reported (PMID: **35641829**).

Furthermore, we would like to emphasize that future studies examining the impact of organoid treatment on kidney function after transplantation should include contralateral kidney nephrectomy or utilize kidney injury models. To facilitate a more thorough discussion of the findings presented here, we have added a section in the manuscript titled 'Limitations of the Study,' where we address this and other study limitations.

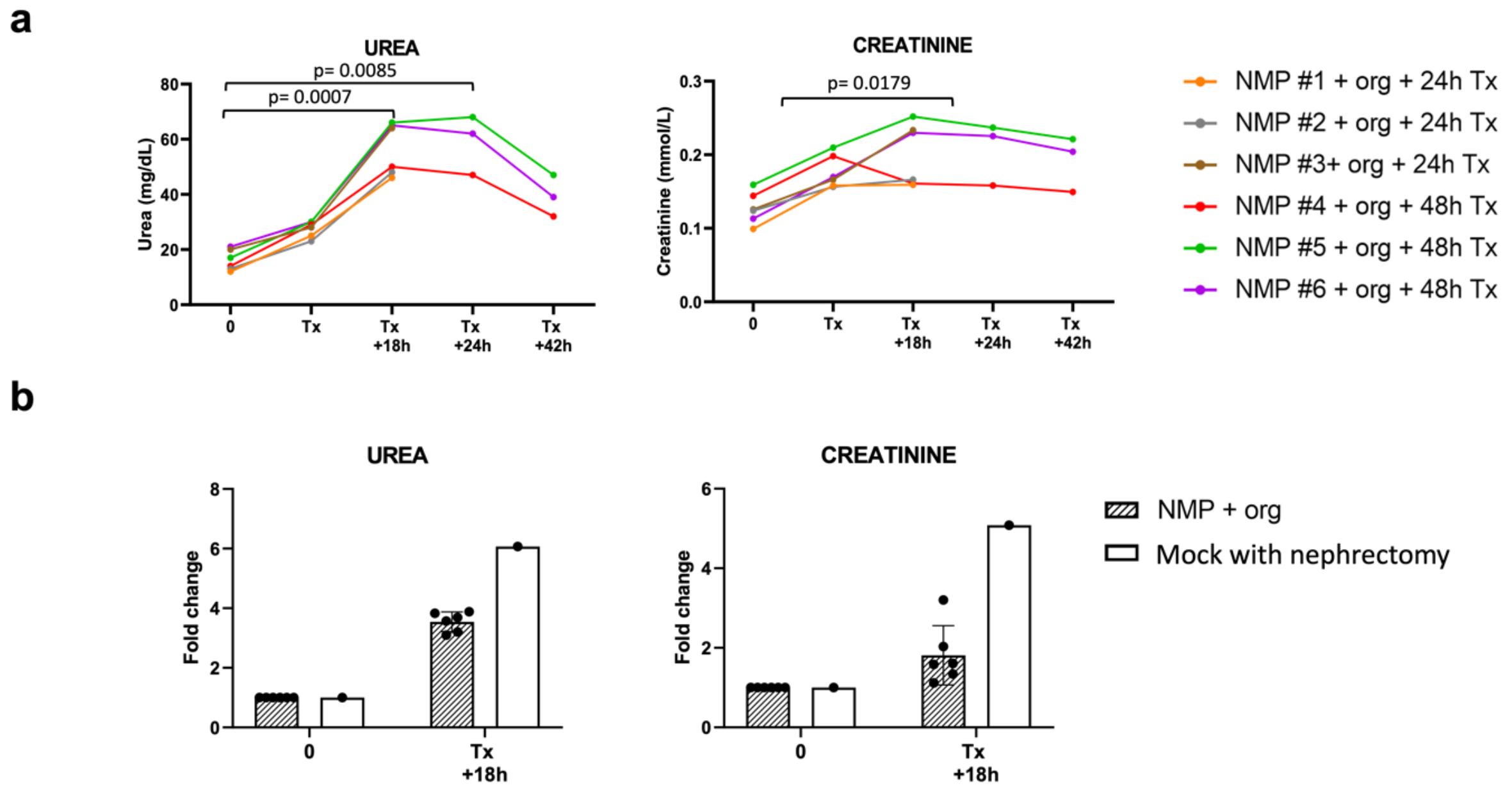

Figure 3*. a)** *Blood urea and creatinine analysis performed in recipient animals before (t0, basal) and after transplantation (Tx). Data are mean ± s.d. n= 3 animals transplanted with organoid-treated kidneys for 24h (NMP #1, #2, #3 + org + 24h Tx) and n= 3 animals transplanted with organoid-treated kidneys for 48h (NMP #4, #5, #6 + org + 48h Tx). Kruskal-Wallis (two-sided) followed by Dunn's multiple comparisons test. p values for statistically significant comparisons are shown.* ***b) *Blood urea and creatinine levels in recipient animals at Tx+18h of postoperative follow up relative to basal values (t0). Data are mean ± s.d. n= 3 animals transplanted with organoid-treated kidneys for 24h (NMP #1, #2, #3 + org + 24h Tx) compared to n= 1 animal transplanted with a kidney without organoids (Mock kidney with nephrectomy of the contralateral kidney).*
